# Spectroscopy and Theory of Acetylene Coupling Reactions in Ti^+^(C_2_H_2_)_*n*_ Complexes

**DOI:** 10.1021/acs.jpca.5c05395

**Published:** 2025-09-09

**Authors:** Anna G. Poncelet, John R. C. Blais, Richard B. Odonkor, Michael A. Duncan

**Affiliations:** Department of Chemistry, University of Georgia, Athens, Georgia 30602, United States

## Abstract

Ti^+^(C_2_H_2_)_*n*_ complexes produced by laser vaporization in a supersonic expansion are investigated with mass spectrometry, infrared laser photodissociation spectroscopy, and UV laser photodissociation. The mass distributions of the cluster ions produced are found to vary significantly with the sample rod mounting configuration in the source. For infrared spectroscopy experiments, the so-called “offset” rod mounting produces colder conditions than the “cutaway” configuration, which allows tagging the ions with one or more argon atoms for the *n* = 1 and 2 complexes. Infrared photodissociation spectra for these ions allow the identification of cation-π complexes (*n* = 1, 2) and reaction products from acetylene coupling (*n* = 2). A TiC_4_ metallacycle ion is identified by experiment and theory as the dominant reaction product. Larger complexes could not be tagged with argon under our conditions and therefore infrared spectra could not be measured. Under warmer expansion conditions with the cutaway rod configuration, prominent Ti^+^(C_2_H_2_)_3_ and Ti^+^(C_2_H_2_)_6_ ions are formed. UV photodissociation patterns for these ions are found to be almost identical to those for the corresponding Ti^+^(C_6_H_6_) and Ti^+^(C_6_H_6_)_2_ ions, suggesting that acetylene cyclization reactions have produced benzene and di-benzene complexes. Reaction path computations for both the *n* = 2 and 3 complexes investigate the energetics of the cyclization reactions in these systems.

## Graphical abstract

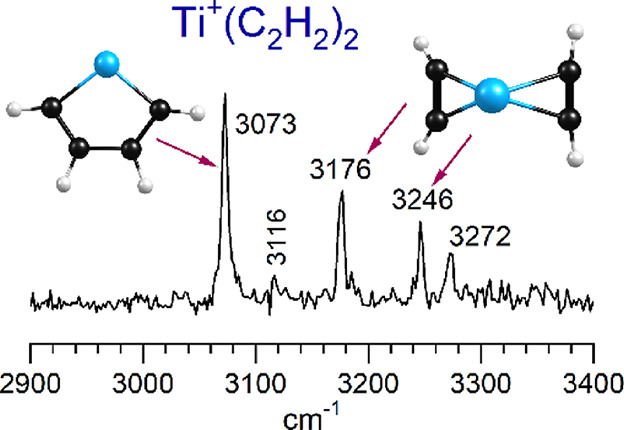

## Introduction

The cycloaddition reaction to form benzene from acetylene is well-known and has been studied extensively.^1–4^ Because of the strong bonds in acetylene which must be broken, this reaction involves significant activation barriers. Carefully chosen catalysts with high pressure and temperature reaction conditions are usually required.^1–4^ The intermediates in this chemistry and the reaction mechanisms have been investigated with gas phase ions and mass spectrometry on metal cation-acetylene complexes, complemented by computational studies.^5–25^ Our laboratory has focused on the spectroscopy of metal ion-acetylene complexes, using UV–visible or infrared laser photodissociation spectroscopy measurements.^26–42^ In complexes containing multiple acetylene molecules, where ligand-coupling reactions are possible, infrared spectroscopy makes it possible to identify reaction products versus the formation of “unreacted” cation-π complexes.^29–42^ Experiments to date and recent computational studies suggest that reactions in these systems should be more efficient for early transition metals. We therefore investigate the titanium-acetylene system in the present work.

Metal ion-acetylene complexes have been studied extensively in mass spectrometry. Early work investigated ion–molecule reactions and other studies used collision-induced dissociation measurements to investigate bond energies.^5,7–9,14–22^ Computational chemistry examined structures, thermochemistry, and the possibility of metal-catalyzed reactions.^6,10–13,23–25^ Alkaline earth metal ion complexes were found to have unpaired valence electrons and low energy electronic spectra, producing details on the structures of single-ligand complexes.^26–28^ More recently, photodissociation threshold spectroscopy and photofragment imaging were used to determine bond energies.^42^ Infrared photodissociation spectroscopy measurements by our group investigated multiple-ligand complexes for several transition metal ion complexes with acetylene.^29,30,32,33,35–42^ Charge-transfer interactions were found to cause vibrational bands in the C–H stretching region to shift to frequencies lower than those of acetylene. Computational studies predicted the infrared spectra for various metal complex structures and spin states. Recognizable band patterns were found for the products of ligand-coupling reactions, which could be clearly distinguished from those of unreacted complexes. Reactions for many metal ions (Ni^+^, Cu^+^, Ag^+^, Au^+^, Fe^+^, Pt^+^), were inhibited by energetic barriers, and cation-π complexes formed with individual acetylene ligands coordinating around the central metal ion.^29,30,32,33,35,38–41^ Additional ligands beyond the inner coordination formed solvation networks. In certain systems (V, Zn),^35,37^ ligand coupling reactions were detected via distinctive patterns in their infrared spectra. For vanadium ion-acetylene complexes, metallacycle intermediates were formed and eventually the metal ion-benzene complex.^35^ The chemistry of zinc ions was different, with end-to-end ligand coupling reactions forming polyacetylene structures.^37^

Titanium ions are recognized to be particularly reactive with small hydrocarbons, forming both ion–molecule complexes and insertion products.^43–46^ Indeed, reactions with methane or acetylene were employed to produce the famous titanium carbide “met-cars” and “nanocrystal” clusters studied by several research groups in the 1990s.^47–51^ However, the reaction conditions used to produce metal carbide clusters were quite different from those used to produce metal cation-acetylene complexes. It is therefore interesting to see if titanium ion-molecule complexes can be produced instead of carbides and to investigate the structures and possible reaction products that might form. As an additional consideration, Murakami et al. have recently performed a computational study of different metal ions and their ability to catalyze acetylene cyclization chemistry.^23–25^ Early transition metals were found to have lower activation barriers for this chemistry than later transition metals. We therefore investigate the titanium-acetylene system here with both infrared and UV-visible photodissociation experiments.

## Methods

Cation-molecular complexes of the form Ti^+^(C_2_H_2_)_*n*_ and Ti^+^(C_2_H_2_)_*n*_Ar_*m*_ were produced by laser vaporization^52^ of a rotating metal rod in a pulsed supersonic expansion containing about 1–3% acetylene in argon. “Offset” and “cutaway” configurations of the source were employed in different experiments.^52^ The ions were analyzed and selected for study with a reflectron time-of-flight mass spectrometer designed for photodissociation experiments.^53,54^ Mass selection was accomplished with pulsed deflection plates using the transit time through the first flight tube section of the instrument. Photodissociation was accomplished by laser excitation at the turning point in the reflectron field, and fragment mass analysis was determined by the flight time through the second flight tube section. Tunable infrared radiation for these experiments was provided by a Nd:YAG-pumped optical parametric oscillator/amplifier (OPO/OPA) laser system (LaserVision). Because the binding energies of acetylene ligands to titanium cations are generally greater than the infrared photon energy, ion-molecule complexes were “tagged” with argon atoms to enhance photodissociation yields. IR excitation of acetylene vibrations in the C–H stretching region leads to the elimination of argon from these complexes. In each case, the yield of the fragment ion mass was recorded versus the IR photon energy to obtain the infrared spectrum. Computational studies of complexes with or without argon were used to investigate the effects of argon tagging on the vibrational spectra.

UV–visible photodissociation experiments employed the same methods as the infrared experiments except that ions were studied without tagging. The 355 or 266 nm wavelengths from a Nd:YAG laser (Continuum Surelite) were used for ultraviolet excitation.

Computational studies on the titanium cation-acetylene complexes were carried out with the Gaussian16 program package,^55^ using density functional theory (DFT), the B3LYP functional, and the def2-TZVP basis set.^56^ Infrared frequencies from theory were scaled by a factor of 0.96 for comparison to experimentally measured spectra. Energetics were corrected for the harmonic, nonscaled zero point energies. To identify transition states, we used the Synchronous Transit-Guided Quasi-Newton (STQN) Method in Gaussian16 with the QST3 command. Transition states were confirmed with subsequent TS and IRC calculations.

## Results and Discussion

Laser vaporization of titanium with acetylene produces fascinating mass spectra that vary with the vaporization laser intensity and nozzle conditions. Two examples of such mass spectra are presented in Figure 1. These mass spectra are affected by the isotopes of titanium (46–8.3; 47–7.4; 48–73.7%) and small amounts of oxides from residual oxide on the metal rod surface. Some of the mass assignments indicated are not unique, i.e., TiO^+^(C_3_H_6_O) vs TiO_3_^+^(C_2_H_2_), and because of the limited mass resolution these cannot be resolved. However, because of the subsequent mass selection these assignments do not affect any of the results described here. In both source configurations, argon interacts with metal vapor and acetylene in the throat of the supersonic expansion where collisional cooling is taking place. The gases are heated by the vaporization laser and the hot metal vapor it produces as well as the exothermicity of reactions (if any), and cooled by the collisions in the supersonic expansion. The two different source configurations differ in the position of the ablation laser focus and in how the gas flows through this region. Another variable is the laser pulse energy, which varies in different experiments as needed to produce ions (gas flow and laser pulse energy affect the plasma temperature, which affects ion-electron recombination).^52^ It is unfortunately not possible in either configuration to determine the exact temperature. However, both of these configurations have produced low rotational temperatures of 10–30K for other nonreacting ions in previous work.^36^ The presence of argon tagging is evidence that similar low temperatures have been achieved.

**Figure 1:**
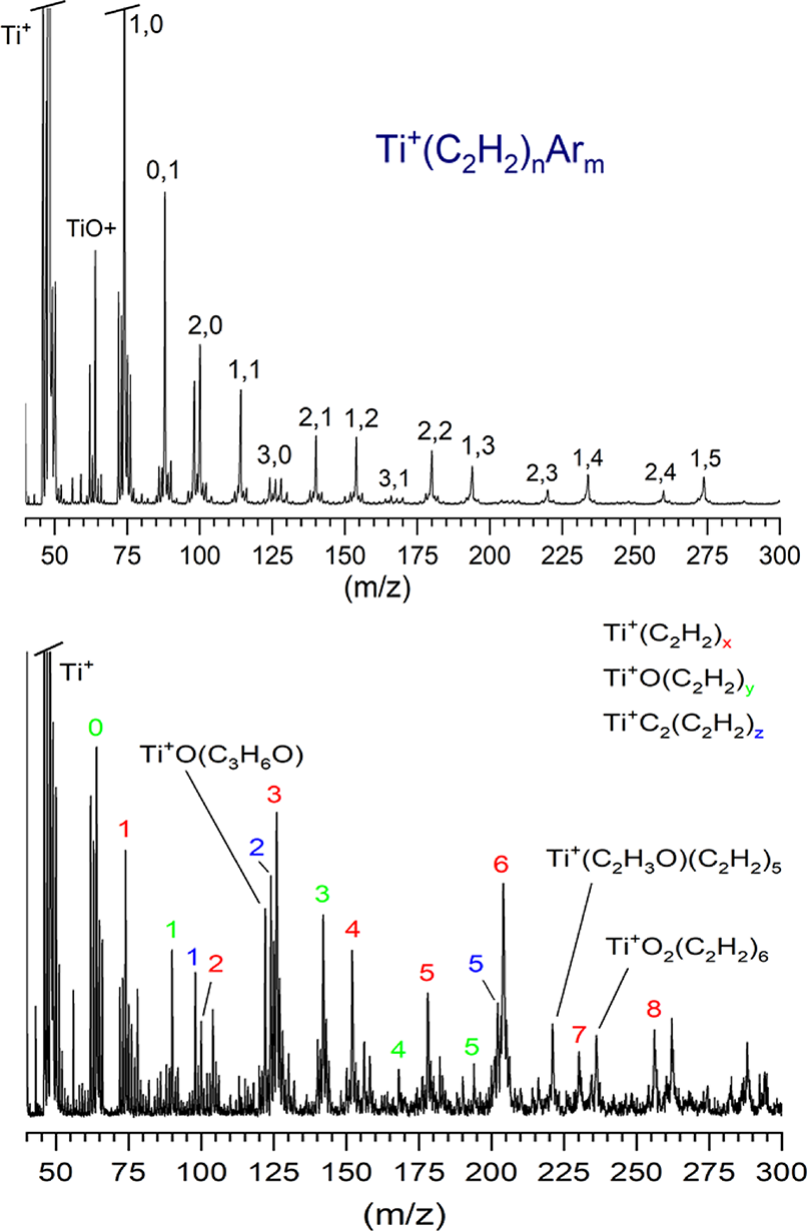
Mass spectra for laser vaporization of titanium with acetylene under different conditions. The upper trace shows the spectrum measured from an offset configuration for mounting the metal rod, whereas the lower trace shows the spectrum measured from a cutaway configuration for the rod holder.

The upper trace of Figure 1 shows the mass spectrum produced from the so-called “offset” source,^52^ in which the titanium metal rod is mounted off to the side from the nozzle jet expansion. Laser vaporized material is ejected from this rod into the gas flow where it is entrained. Metal vapor from this configuration is cooled more effectively and the acetylene in the gas flow is not heated significantly by the laser plasma. Lower ablation laser pulse energies (3–5 mJ/pulse) were used in this configuration. The lower trace of Figure 1 shows the mass spectrum produced from the so-called “cutaway” source,^52^ in which the titanium rod is mounted in a block with the gas flowing directly over its surface. In this configuration, the acetylene gas is heated more by the laser ablation plasma. Higher laser pulse energies were required (20 mJ/pulse) to produce ions. The offset source configuration produces colder ions and is more effective for argon tagging, as shown in the ion masses including argon. The cutaway source produces warmer ions and does not produce tagged ions efficiently. However, its warmer conditions may promote reactions that have activation barriers. The mass spectrum from the cutaway source has more ions of the form TiC_2_^+^(C_2_H_2_)_*n*_, and the Ti^+^(C_2_H_2_)_3_ and Ti^+^(C_2_H_2_)_6_ masses, which could conceivably correspond to benzene or di-benzene reaction products, are much more prominent.

### Infrared Photodissociation Spectra

We first investigate the Ti^+^(C_2_H_2_)_*n*_ complexes with infrared photodissociation spectroscopy. For these measurements, the corresponding argon-tagged ions are mass selected and the fragment ion corresponding to argon elimination is recorded as a function of the infrared wavelength. Figure 2 shows the infrared spectrum measured in this way for the Ti^+^(C_2_H_2_)­Ar_2_ ion in the mass channel corresponding to the loss of one argon. The Ti^+^(C_2_H_2_)Ar ion did not photodissociate efficiently and therefore we tagged this complex with two argon atoms. The computed argon binding energy for the singly tagged complex (7.4 kcal/mol = 2588 cm^–1^; see Table S4) is lower than C–H stretch energies, but these complexes did not dissociate with IR excitation. Either the DFT computed bond energies are unreliable (likely) or the rate of intramolecular vibrational energy transfer (IVR) from the C–H stretch that is excited to the argon stretch is low (also possible). The spectrum shown for the doubly tagged complex in the figure has two bands at 3051 and 3076 cm^–1^. These resonances appear at lower frequencies than the antisymmetric and symmetric stretches of acetylene, which occur at 3289 and 3374 cm^–1^ respectively^57^ (positions indicated with dashed vertical lines). This kind of shift to lower frequencies, i.e., a “red” shift, has been observed for all previous examples of metal ion-acetylene complexes. Consistent with the well-known Dewar-Chatt-Duncanson model of π bonding,^58–61^ this red shift is attributed to a charge-transfer interaction that removes electron density from the C–C and C–H bonds, lowering the vibrational frequencies.

**Figure 2:**
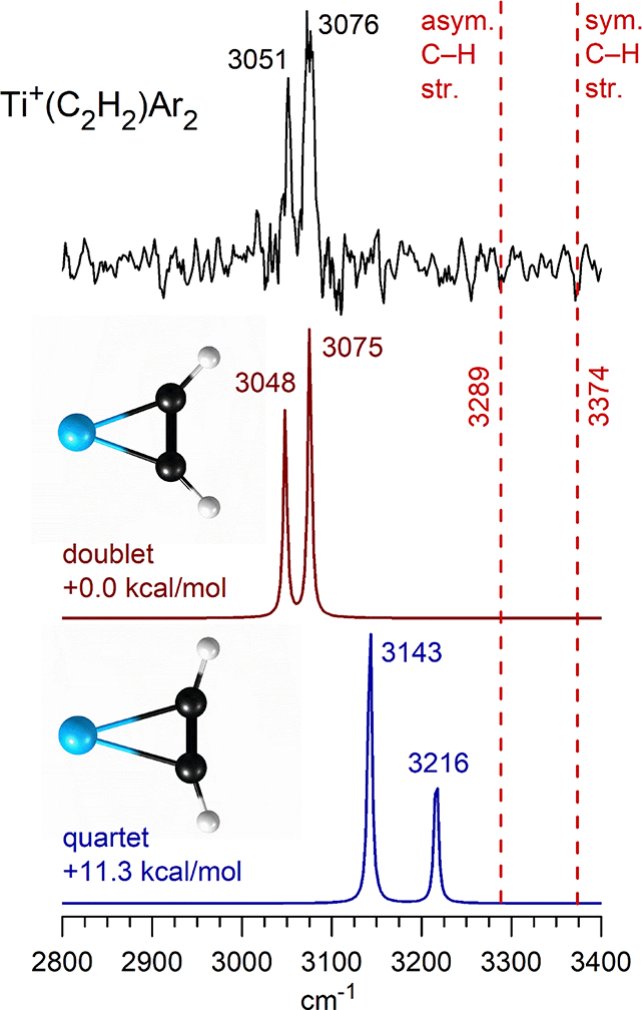
Infrared photodissociation spectrum measured for the Ti^+^(C_2_H_2_)­Ar_2_ ion compared to the spectra predicted by theory for the doublet versus quartet electronic states for this ion.

We use theory at the DFT/B3LYP level to investigate the structures and infrared spectra of the various Ti^+^(C_2_H_2_)_*n*_ complexes, including complexes tagged with argon and those without tagging. We investigated the doublet and quartet spin states for each of these systems. The full details of the computations are presented in the Supporting Information file. The structures identified for the *n* = 1–3 complexes are shown in Figure 3. Table 1 presents a summary of the energetics for the *n* = 1–3 complexes and the individual acetylene molecule and titanium ion composing these.

**Figure 3:**
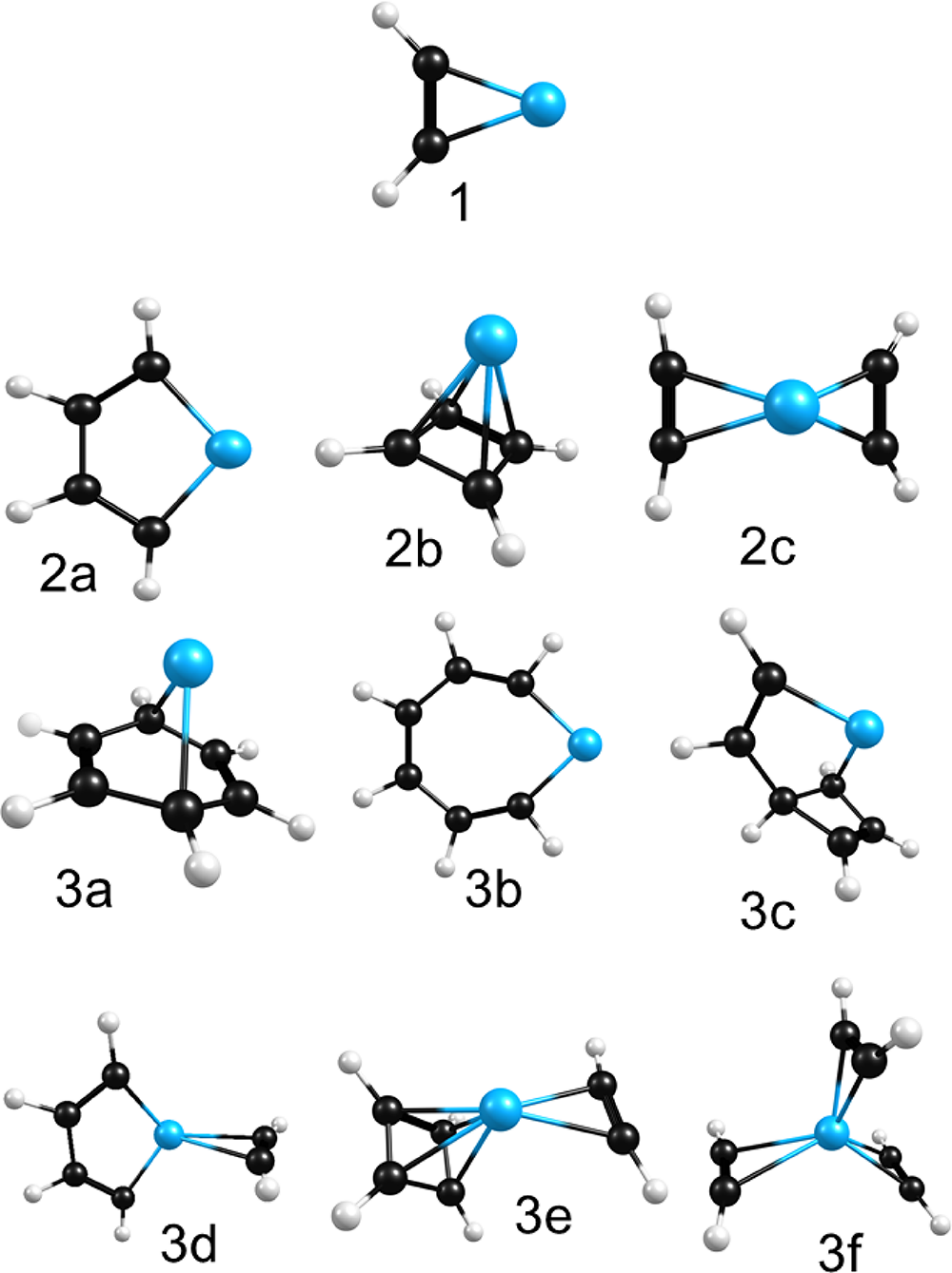
Structures for the Ti^+^(C_2_H_2_)_*n*_ (*n* = 1–3) complexes identified in the computational studies for this system.

**Table 1:** Energetics of Ti^+^(C_2_H_2_)_*n*_ Complexes from Computational Studies^a^

*n*	2S + 1	isomer	*E* (Hartree)	relative energy (kcal/mol)	BDE acetylene elimination (kcal/mol)	BDE benzene elimination (kcal/mol)
0	2		–849.105520	12.7		
0	4		–849.125763	0.0		
1	2	1a	–926.538288	0.0	59.7	
1	4	1a	–926.520344	11.3	35.7	
2	2	2a	–1003.949568	0.0	46.2	
2	4	2a	–1003.905443	27.7	29.8	
2	2	2b	–1003.939542	6.3	39.9	
2	4	2b	–1003.918417	19.5	37.9	
2	2	2c	–1003.92452	15.7	30.5	
2	4	2c	–1003.898632	32.0	25.5	
3	2	3a	–1081.438582	9.3	110.7	60.2^b^
3	4	3a	–1081.453355	0.0	136.2	
3	2	3b	–1081.357344	60.2	59.7	
3	4	3b	–1081.318071	84.9	51.3	
3	2	3c	–1081.342759	69.4	50.6	
3	4	3c	–1081.302305	94.8	41.4	
3	2	3d	–1081.336558	73.3	30.9	
3	4	3d	–1081.288714	103.3	28.6	
3	2	3e	–1081.318760	84.5	35.5	
3	4	3e	–1081.302158	94.9	41.3	
3	2	3f	–1081.302218	94.8	25.1	
3	4	3f	–1081.280336	108.6	27.6	

a Bond dissociation energies (BDE) are indicated for the elimination of either acetylene or benzene.

b Experimental value: 61.9 kcal/mol.^17^

Theory finds a cation-π structure for the Ti^+^(C_2_H_2_) complex, with the titanium ion located symmetrically over the midpoint of the C–C bond and 1.82 Å away from this. The doublet is predicted to be the electronic ground state, lying 11.3 kcal/mol lower in energy than the quartet. The C–C bond in the complex (1.32 Å) is elongated with respect to that of free acetylene (computed at 1.21 Å). This elongation is greater than that for other metal ion-acetylene complexes (e.g., 1.23 Å for Co^+^(C_2_H_2_)).^29–42^ The hydrogens in the acetylene structure are bent significantly away from the linear structure of acetylene, with a C–C–H angle of 43°. This is also a greater distortion than that found for other metal ion-acetylene complexes (e.g., 16.2° for Co^+^(C_2_H_2_)).^29–42^

As shown in Figure 2, the doublet state reproduces the vibrational spectrum nicely, with a two-band pattern and the higher frequency band having a greater intensity just like that in the experiment. The quartet has a very different pattern, with two bands lying at higher frequencies and more widely spaced. Consistent with the computed energetics, the doublet is confirmed to be the ground state. We studied the effects of argon bonding to the Ti^+^(C_2_H_2_) ion in different isomeric structures, and found that no significant shifts are caused on these vibrations (see Supporting Information Figures S18–S21). This is somewhat surprising considering the strong attachment of argon to this complex (bond energy 7.4 kcal/mol), but the most stable structure has the argon attached to the metal ion remote from the C–H stretches. In free acetylene, the symmetric stretch is IR-inactive and only the antisymmetric stretch is detected in the spectrum.^57^ In metal-ion complexes, the acetylene is bent and the symmetric stretch becomes IR-active, although usually with weaker intensity than the antisymmetric stretch. The pattern seen here, with the higher intensity band at higher frequency, has only been seen before for the V^+^(C_2_H_2_) complex, which is the system found to be most reactive for cyclization chemistry.^35^ In metal ion-acetylene complexes studied previously, the red shift usually places the C–H stretches in the 3150–3250 cm^–1^ region.^31,36^ Again, the red shift is greater here and more like that in the corresponding vanadium complex, where the bands were at 3067 and 3097 cm^–1^. The titanium complex is therefore an outlier like vanadium, with a more distorted acetylene structure and an infrared spectrum shifted more to the red than those of other metals. According to the Dewar-Chatt-Duncanson model,^58–61^ the red shift of the C–H stretches and the strength of the resulting bonds are associated with electron withdrawal from the ligand to the metal. Early transition metals like vanadium and titanium have fewer *d* electrons and more unoccupied *d* orbitals, which facilitates the transfer of electron density from the acetylene to the metal.

Figure 4 shows the infrared spectrum measured for the Ti^+^(C_2_H_2_)_2_Ar_2_ ion compared to the predictions of theory for the tag-free ion. We ignore the argon here since our computations showed that it had no significant effect on band positions (see Supporting Information Figures S34–S45). The infrared spectrum has four main bands at 3073, 3176, 3246, and 3272 cm^–1^ and a hint of another feature at 3116 cm^–1^. Theory finds three main isomers for the structure of this ion (Figure 3), whose predicted spectra are also shown in the figure. In each case the doublet lies lower in energy than the quartet. The MC_4_ metallacycle (isomer 2a) is the most stable structure, followed by the metal-cyclobutadiene π complex (isomer 2b) and the diacetylene π complex (isomer 2c), respectively. The latter is an unreacted structure, whereas the first two result from acetylene cyclization reactions. The infrared spectrum has prominent bands matching those predicted for isomers 2a and 2c, and a weak feature matching the most intense band predicted for isomer 2b. The band at 3073 cm^–1^ is the most intense feature in the experimental spectrum, and it clearly matches the single feature predicted at 3060 cm^–1^ for isomer 2a. This indicates that acetylene cyclization chemistry has occurred. The band at 3176 and 3246 cm^–1^ in the experimental spectrum match those predicted at 3168 and 3250 cm^–1^ for isomer 2c. These correspond to complexes which have added acetylene to the metal ion but have not gone on to react any further through cyclization chemistry. Finally, the weak feature at 3116 cm^–1^ provides a hint that some ions have formed the isomer 2b structure. The band at 3272 cm^–1^ does not match the positions of any of the fundamental vibrations predicted for these isomers. However, it can be tentatively assigned as a combination band with the 3073 cm^–1^ feature. A metal-C_4_ stretch is predicted to have a harmonic fundamental at 250 cm^–1^, and this vibration could easily couple with the hydrogen stretch vibration.

**Figure 4:**
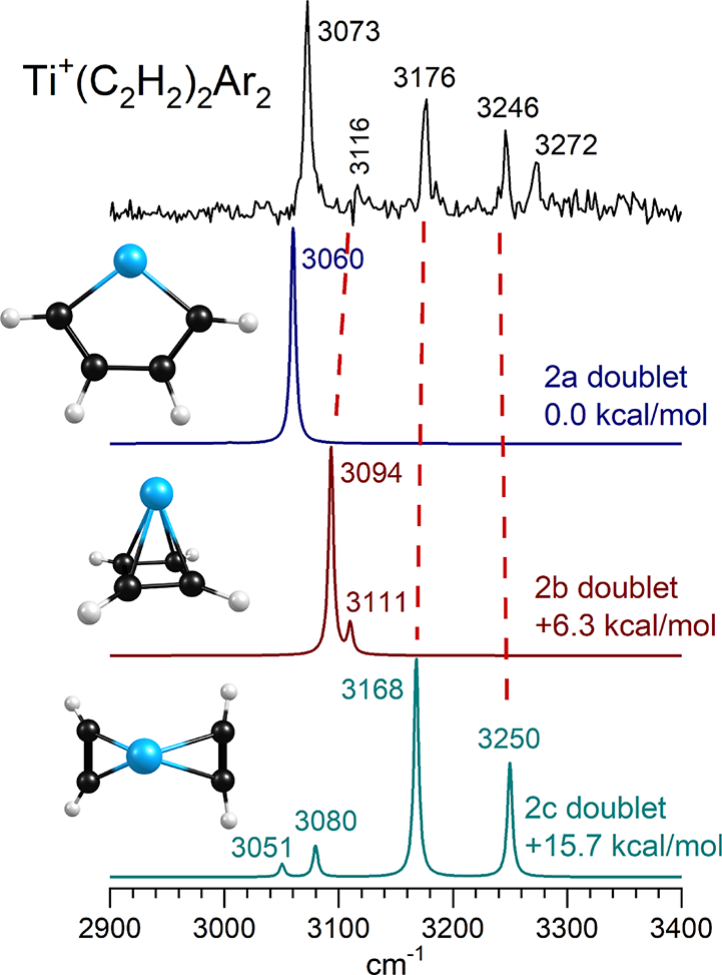
Infrared photodissociation spectrum measured for the Ti^+^(C_2_H_2_)_2_Ar_2_ ion compared to the spectra predicted by theory for different isomeric structures for this ion.

To further investigate the cyclization chemistry which apparently occurs for this system, we have computed the energetics of the reaction coordinate, which is shown in Figure 5. We begin at the left of the figure with the unreacted *n* = 2 isomer (isomer 2c). This makes sense because the initial encounter of an *n* = 1 complex with an acetylene molecule should produce such an unreacted structure. The energetics in the figure are relative to isomer 2a in its doublet electronic state. As shown, the doublet electronic surface is lower in energy than the quartet for all structures and transition states. According to theory, there is only a small barrier (1.1 kcal/mol) for the reaction from isomer 2c to produce isomer 2a. It therefore is understandable how the experiment could produce isomer 2a, as shown in Figure 3. Isomer 2b, which has the metal-cyclobutadienyl structure, lies only 6.3 kcal/mol above isomer 2a, but it lies behind a significant barrier of 32.1 kcal/mol. This explains why isomer 2b is not produced efficiently. It is in fact remarkable that there seems to be some slight evidence for a vibrational band corresponding to this isomer. However, a note of caution is in order here. DFT has known problems with transition state energies, and therefore the energetics along the reaction path here are not likely to be highly accurate.

**Figure 5:**
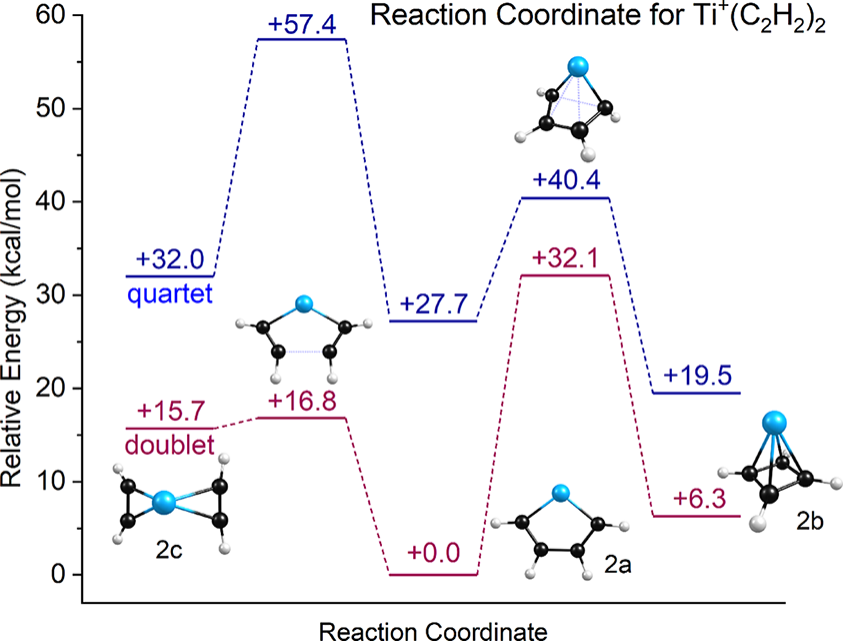
Reaction coordinate predicted by theory for the Ti^+^(C_2_H_2_)_2_ ion. Energetics are relative to the most stable metallacycle structure of isomer 2a.

A metallacycle structure like isomer 2a has been reported previously by Armentrout and co-workers from the reaction of tantalum ions with ethylene.^62^ A similar structure was also reported in our previous work on vanadium ion reactions with acetylene.^35^ This was confirmed to be the first intermediate structure along the way to the eventual production of the metal ion-benzene complex. In the present system, this initial acetylene-coupling reaction product is formed efficiently because the activation barrier to its formation from the unreacted isomer 2c (1.1 kcal/mol) is much lower than it was for other metal ion-acetylene complexes.

The signal levels for the Ti^+^(C_2_H_2_)_3_ complex and its corresponding tagged ion are extremely small under conditions used to produce the IR spectra for the n = 1 and 2 ions (see upper mass spectrum in Figure 1). Therefore, we were unable to obtain IR spectra for these ions. However, as shown in the lower mass spectrum of Figure 1, warmer ion production conditions with the different sample rod mounting configuration produce quite prominent mass peaks corresponding to Ti^+^(C_2_H_2_)_3_ and Ti^+^(C_2_H_2_)_6_, which are of course the same masses as Ti^+^(C_6_H_6_) and Ti^+^(C_6_H_6_)_2_. This suggests that these more energetic conditions may have allowed the production of the benzene and dibenzene complexes. It is not possible to confirm this without infrared spectra, but another approach is to investigate the UV photodissociation patterns of these ions and to compare them to the corresponding masses produced using benzene in the expansion gas mixture instead of acetylene.

### UV Photodissociation Measurements

Figure 6 shows the photodissociation mass spectra of Ti^+^(C_2_H_2_)_3_ compared to that for the corresponding Ti^+^(C_6_H_6_) ion at 266 nm. This photodissociation mass spectrum was accumulated in a difference mode of operation, in which the selected parent ion intensity without laser excitation is subtracted from that with laser excitation. The depletion of the parent ion is then represented by a negative-going signal and the fragment ions produced from it give positive mass peaks. As indicated, each of these ions produce a somewhat similar pattern of fragment ions. Although the relative intensities of the fragments are somewhat different, the fragments produced are essentially the same. These include the Ti^+^ atomic metal ion, the Ti^+^(C_2_H_2_) metal-acetylene ion, and the Ti^+^(C_2_H_2_)_2_ ion. Both complexes seem to eliminate a sequence of acetylene molecules. Additionally, both of these ions produce the Ti^+^(C_2_)­(C_2_H_2_) fragment, presumably via the loss of H_2_ in addition to an acetylene. This sort of reaction is not surprising, since titanium reactions with acetylene under other conditions produces larger metal carbide clusters.^47–51^ The relative intensity differences in these compared spectra could easily be from different internal temperatures in the ion production process. Therefore, these data are consistent with a Ti^+^(C_2_H_2_)_3_ ion having the same structure as Ti^+^(C_6_H_6_). A similar result is found for the larger ions, where we compare the fragmentation of Ti^+^(C_2_H_2_)_6_ and Ti^+^(C_6_H_6_)_2_ in Figure 7. Again, the fragmentation patterns - in this case the elimination of the mass of either three acetylenes or benzene - are the same. Neither ion has fragments corresponding to intermediate losses of acetylene. This is consistent with these ions having the same dibenzene structure, suggesting again that acetylene has undergone cyclization chemistry. In both of these comparisons, we have studied the fragmentation processes at different wavelengths (532, 355, and 266 nm and laser powers; see Supporting Information Figures S3 and S4). In each case the fragmentation is essentially the same. These results indicate that titanium likely does react with acetylene to form these benzene and dibenzene complexes.

**Figure 6:**
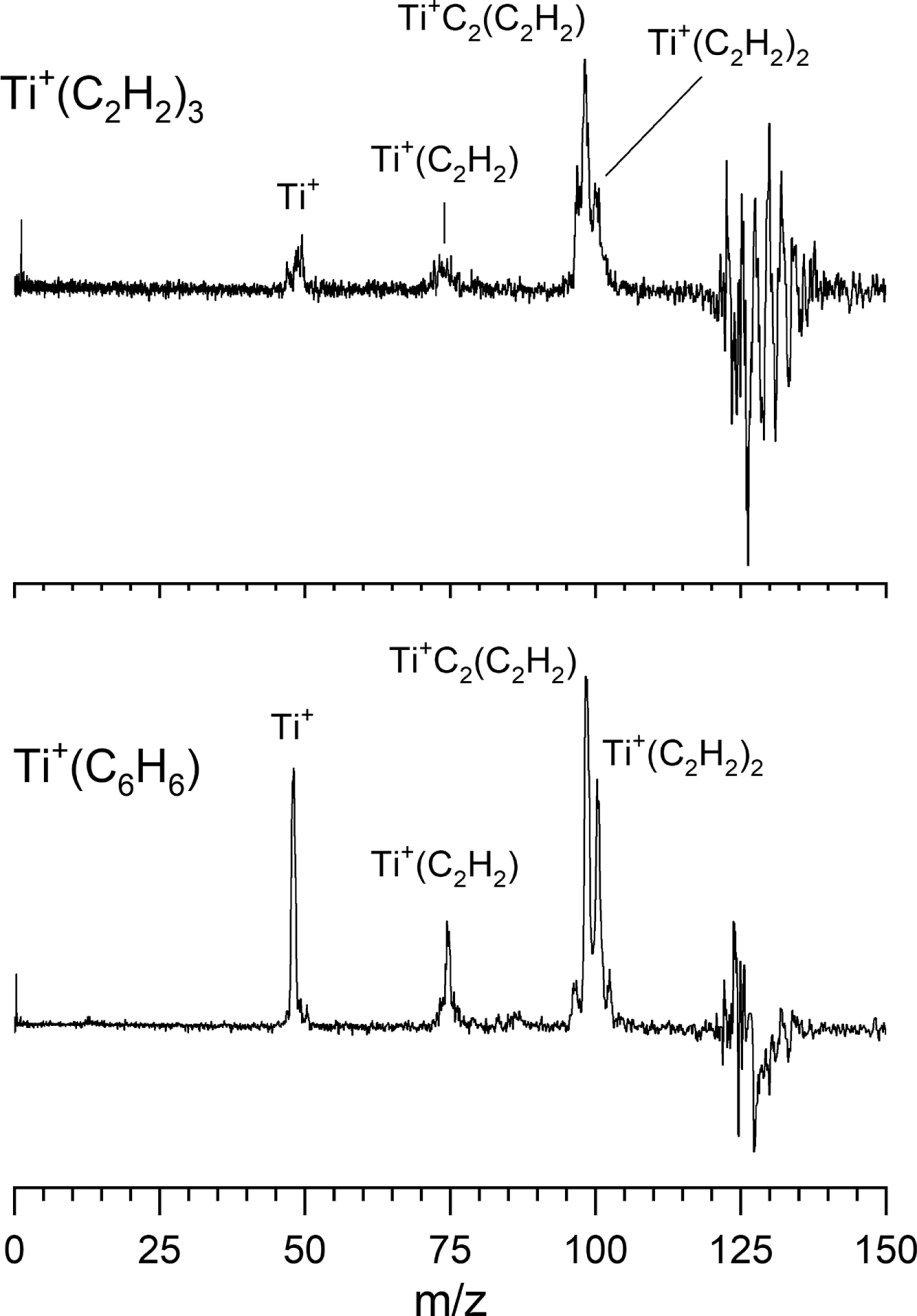
Photodissociation mass spectrum of the Ti^+^(C_2_H_2_)_3_ versus that for Ti^+^(C_6_H_6_) at 266 nm.

**Figure 7:**
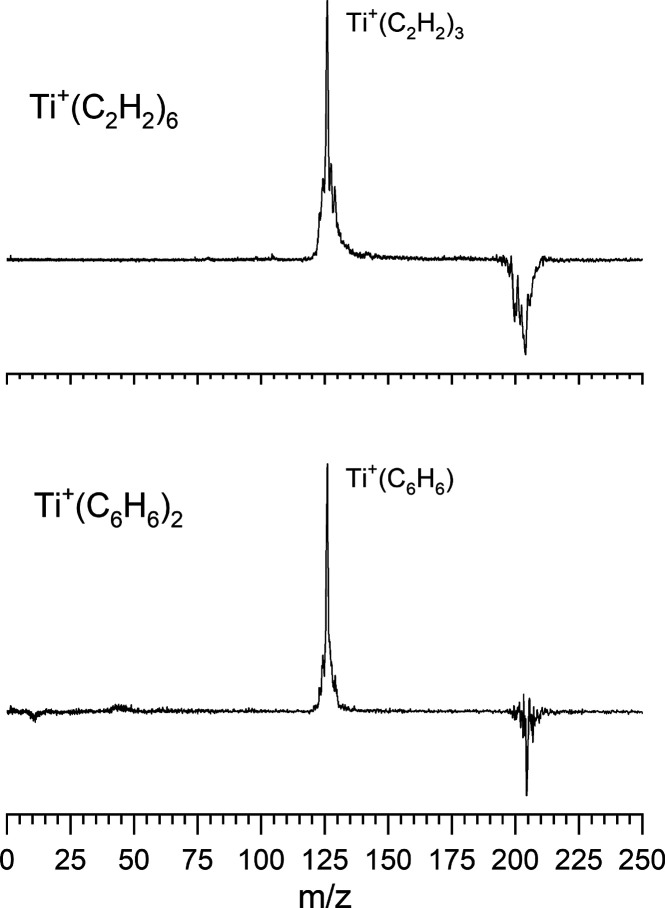
Photodissociation mass spectrum of the Ti^+^(C_2_H_2_)_6_ versus that for Ti^+^(C_6_H_6_)_2_ at 355 nm.

To investigate the energetics of this proposed reaction, we have computed the structures and transition states along the reaction path. The isomers of the *n* = 3 complex are presented in Figure 3. These include the most stable Ti^+^ (benzene) structure (isomer 3a), the MC_6_ metallacycle (isomer 3b), the MC_4_ metallacycle + acetylene (isomer 3d), two isomers with cyclobutadiene rings (3c and 3e), as well as the unreacted three-acetylene structure (isomer 3f). All of these except the 3a benzene complex are more stable in doublet spin states; the metal ion-benzene complex has a quartet ground state. The reaction coordinate resulting from these computations is presented in Figure 8. We begin at the left of the figure with the unreacted *n* = 3 isomer (isomer 3f). This makes sense because the initial encounter of an unreacted *n* = 2 complex with an acetylene molecule should produce such an unreacted structure. The energetics in the figure are relative to isomer 3a in its quartet electronic state. As shown, there is a relatively low barrier of 5.3 kcal/mol for the reaction of isomer 3f to form isomer 3d on the doublet potential surface, which is the first step in the process leading eventually to the metal-benzene complex. Other transition states along this doublet path are lower in energy than isomer 3f, explaining how it is possible for this cyclization reaction to take place. Instead of isomer 2c the reaction might begin with isomer 2a, which is the most stable *n* = 2 species and the one already documented to form. The addition of acetylene to this produces isomer 3d and its conversion to the benzene complex requires passage over a barrier of 8.7 kcal/mol. Regardless of the starting structure, it is easy to see how these reactions would be sensitive to the temperature of the ions. The formation of the benzene complex in this way is quite exothermic, explaining how this product could be difficult to cool and tag. However, theory indicates that the Ti^+^(C_6_H_6_) ion is most stable as a quartet spin state, so a curve crossing would be required for the system to find its way to the most stable benzene complex. It is also conceivable that the reaction produces the benzene product initially in its excited doublet state. Nothing in our experiment allows us to distinguish between the two spin states for Ti^+^(C_6_H_6_).

**Figure 8:**
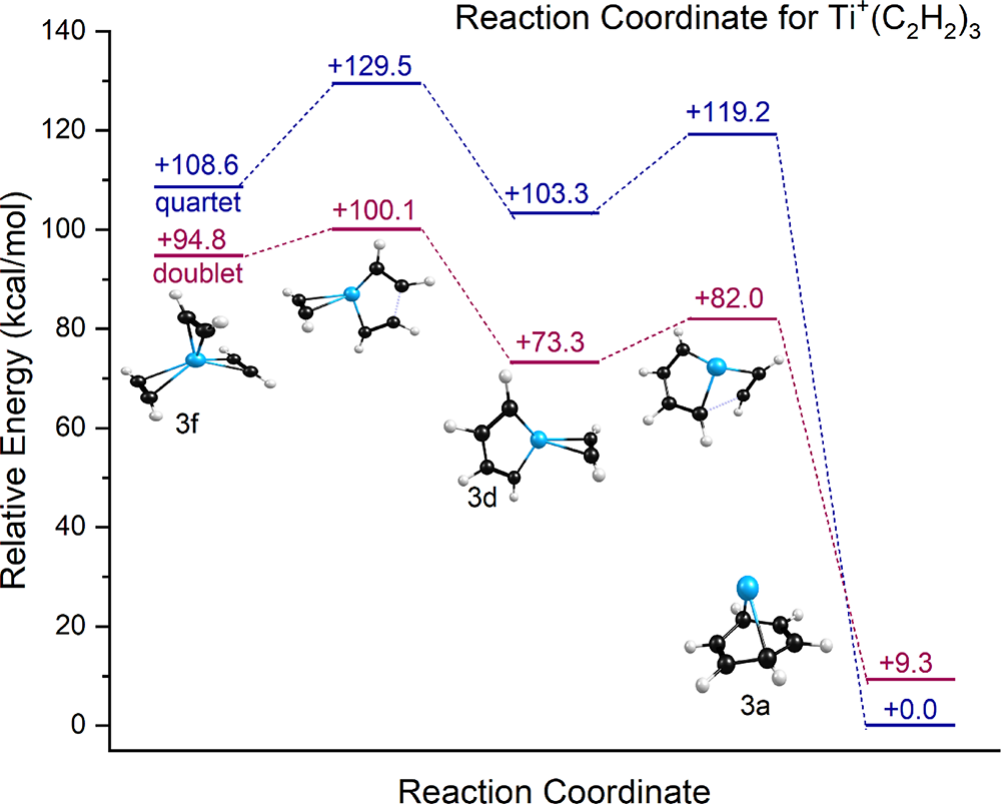
Reaction coordinate predicted by theory for the Ti^+^(C_2_H_2_)_3_ ion.

Confirmation of the reaction that forms the metal ion-benzene complex might be possible in the future if these ions are produced in a cryogenic ion trap where they can be cooled more effectively after reaction for tagging and infrared spectroscopy. The present vaporization source and jet expansion has the disadvantage that reactions and cooling are happening at the same time and place. Infrared multiphoton dissociation would be possible at higher laser powers. Another option would be to use electronic photodissociation spectroscopy, as our group has described for other metal ion-acetylene^26–28^ and -benzene^63,64^ complexes. Metal ion-benzene complexes could be identified because they have characteristic HOMO–LUMO transitions on the benzene molecule as well as charge-transfer resonances that lead to the elimination of the benzene cation.

## Conclusions

Ti^+^(C_2_H_2_)_*n*_ complexes were studied with infrared spectroscopy, UV photodissociation and computational chemistry. The *n* = 1 species forms a cation-π structure in which acetylene is strongly distorted from its isolated-molecule structure. Its C–H stretches are shifted to lower frequencies than any metal cation-acetylene complex yet studied. The *n* = 2 species reacts to form a MC_4_ metallacycle structure, with additional evidence for unreacted diacetylene cation-π complexes. Larger complexes could not be tagged with argon and so infrared photodissociation spectra could not be measured. However, UV photodissociation patterns for Ti^+^(C_2_H_2_)_3_ and Ti^+^(C_2_H_2_)_6_ ions are essentially the same as those for Ti^+^(C_6_H_6_) and Ti^+^(C_6_H_6_)_2_, suggesting that cycloaddition reactions to form benzene and dibenzene complexes did indeed occur. Reaction coordinate calculations are consistent with lower barriers for these acetylene coupling reactions for the titanium ion compared to other transition metal ions studied previously, consistent with the spectroscopy.

Titanium ions are found to have chemistry similar to that of vanadium ions with respect to acetylene cyclization reactions. Vibrational band shifts are greater than those of other transition metal ion complexes with acetylene, and reaction barriers are found by theory to be lower. Both may be influenced by the lower numbers of *d* electrons and the corresponding unoccupied orbitals available to accept ligand electron density. Theory by other groups has also suggested lower activation energies for acetylene cyclization for early transition metal ions.^25^ Future experiments will investigate other early transition metal ion-acetylene complexes to see if this suggested trend is valid.

## Supplementary Material



## Notes

Full citation for ref 55 and the details of the DFT computations done, including the structures and energetics for each of the ions considered (PDF)
