# Supplementary material for: Spectroscopy and Theory of Acetylene Coupling Reactions in Ti+(C2H2) n Complexes
Source: J Phys Chem A. 2025 Sep 9;129(37):8613–21. doi: 10.1021/acs.jpca.5c05395 (PMC12451657; doi:10.1021/acs.jpca.5c05395)
Supplement: Supplementary file 1 [file jp5c05395_si_001.pdf]

Supporting Information

Spectroscopy and Theory of Acetylene Coupling Reactions in $Ti^+(C_2H_2)_n$ Complexes

Anna G. Poncelet,[1] John R. C. Blais,[1] Richard B. Odonkor,[1] Michael A. Duncan*[1]

[1]Department of Chemistry, University of Georgia, Athens, GA 30602

*Email: maduncan@uga.edu

Table of Contents

Gaussian16 full reference	S3
Figure S1: Mass spectrum of $Ti^+(C_2H_2)_n$	S4
Figure S2: Mass spectrum of $Ti^+(C_6H_6)_n$	S5
Figure S3: Photodissociation mass spectrum of $Ti^+(C_2H_2)_6$	S6
Figure S4: Photodissociation mass spectrum of $Ti^+(C_6H_6)_2$	S7
**(C_2H_2)**	
Table S1: energy of acetylene.	S8
Figure S5. Strtucture of acetylene	S9
**Ti^+**	
Table S2: relative energies of titanium cation spin states.	S10
**$Ti^+(C_2H_2)$, $Ti^+(C_2H_2)Ar$, & $Ti^+(C_2H_2)Ar_2$**	
Tables S3 – S5: relative energies	S11 – S13
Figures S6 – S17: *n* = 1 structures from theory	S14 – S25
Figures S18 – S21: simulated spectra	S26 – S29
**$Ti^+(C_2H_2)_2$, $Ti^+(C_2H_2)_2Ar_2$, & $Ti^+(C_2H_2)_2Ar_2$**	
Tables S6 – S8: relative energies	S30 – S32
Figures S22–S33: *n* = 2 structures	S33 – S44
Tables S9 – S35: cartesian coordinates & unscaled vibrational frequencies	S45 – S71
Figures S34 – S45: simulated spectra for tag-free and argon tagged isomers	S72 – S83
Figure S46: *n* = 2 reaction coordinate	S84
**$Ti^+(C_2H_2)_3$**	
Tables S36: relative energies	S85
Figure S47: structures of *n* = 3 isomers	S85
Tables S37 – S48: cartesian coordinates & unscaled vibrational frequencies	S86 – S97
Figures S48–S51: structures of *n* = 3 transition states	S98- S101
Figure S52 – S55: simulated spectra for *n* = 3 isomers	S102 – S105
Figure S56: *n* = 3 reaction coordinate	S106
**$Ti^+(C_2H_2)_4$**	
Table S49: relative energies	S107
Figure S57 : structures of *n* = 4 isomers	S108
Tables S50 – S70: cartesian coordinates & unscaled vibrational frequencies	S109 – S129
**$Ti^+(C_2H_2)_5$**	
Table S71: relative energies	S130
Figure S58: structures of *n* = 5 isomers	S131
Tables S72 – S114: cartesian coordinates & unscaled vibrational frequencies	S132 – S174

Full citation for reference 55:

Frisch, M. J.; Trucks, G. W.; Schlegel, H. B.; Scuseria, G. E.; Robb, M. A.; Cheeseman, J. R.; Scalmani, G.; Barone, V.; Petersson, G. A.; Nakatsuji, H.; Li, X.; Caricato, M.; Marenich, A. V.; Bloino, J.; Janesko, B. G.; Gomperts, R.; Mennucci, B.; Hratchian, H. P.; Ortiz, J. V.; Izmaylov, A. F.; Sonnenberg, J. L.; Williams-Young, D.; Ding, F.; Lipparini, F.; Egidi, F.; Goings, J.; Peng, B.; Petrone, A.; Henderson, T.; Ranasinghe, D.; Zakrzewski, V. G.; Gao, J.; Rega, N.; Zheng, G.; Liang, W.; Hada, M.; Ehara, M.; Toyota, K.; Fukuda, R.; Hasegawa, J.; Ishida, M.; Nakajima, T.; Honda, Y.; Kitao, O.; Nakai, H.; Vreven, T.; Throssell, K.; Montgomery, J. A., Jr.; Peralta, J. E.; Ogliaro, F.; Bearpark, M. J.; Heyd, J. J.; Brothers, E. N.; Kudin, K. N.; Staroverov, V. N.; Keith, T. A.; Kobayashi, R.; Normand, J.; Raghavachari, K.; Rendell, A. P.; Burant, J. C.; Iyengar, S. S.; Tomasi, J.; Cossi, M.; Millam, J. M.; Klene, M.; Adamo, C.; Cammi, R.; Ochterski, J. W.; Martin, R. L.; Morokuma, K.; Farkas, O.; Foresman, J. B.; Fox, D. J. Gaussian 16 (Revision C.01), Gaussian, Inc., Wallingford CT, 2009.

All calculations were performed using DFT at the B3LYP/def2-TZVP level. The thresholds for energy and structure optimizations were set to “tight,” and all calculations used the default “ultrafine” integration grid. The structures presented were checked for electronic wavefunction stability with the “stable=opt” keyword. All structures except for the calculated transition states and second order saddle points are free of imaginary vibrational frequencies, and all electronic energies are zero-point vibrational energy (ZPVE) corrected.

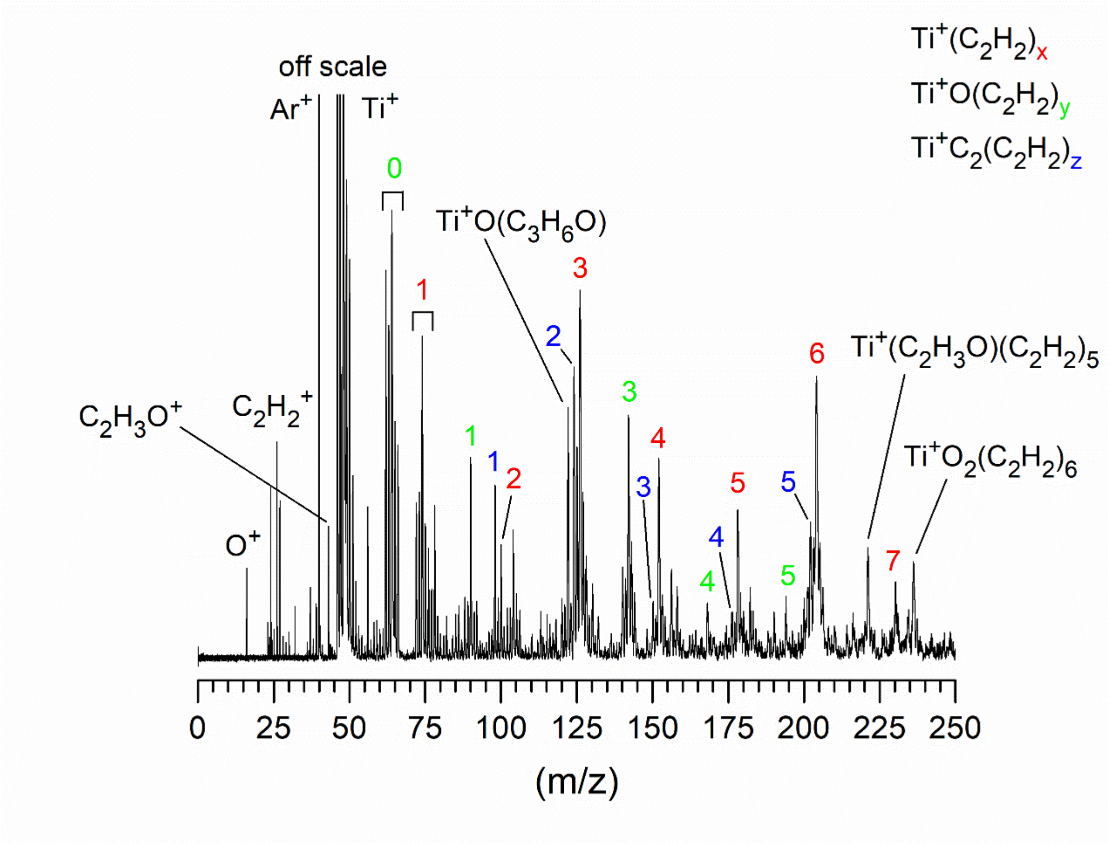

Figure S1. The mass spectrum of $Ti^{+}(C_2H_2)_n$ ions produced by laser vaporization of a titanium rod with a cutaway source configuration in a supersonic expansion of argon with 3% acetylene.

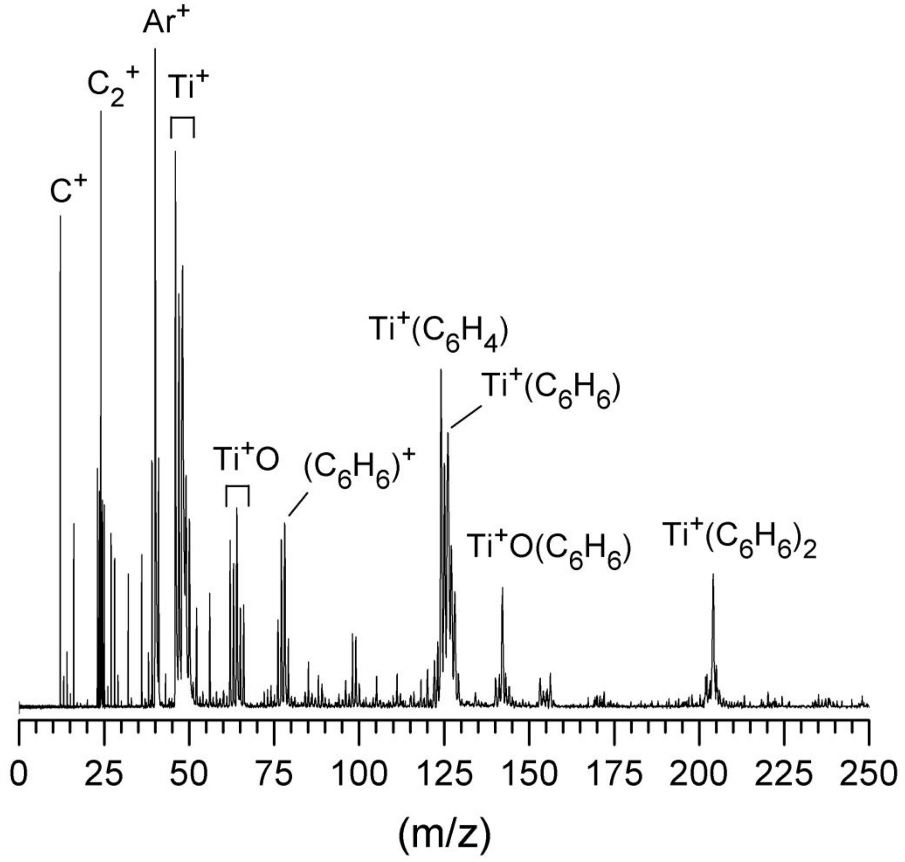

Figure S2. The mass spectrum of $Ti^+(C_6H_6)_n$ ions produced by laser vaporization of a titanium rod with a cutaway source configuration in a supersonic expansion of argon with 1mL of benzene injected in the expansion gas line.

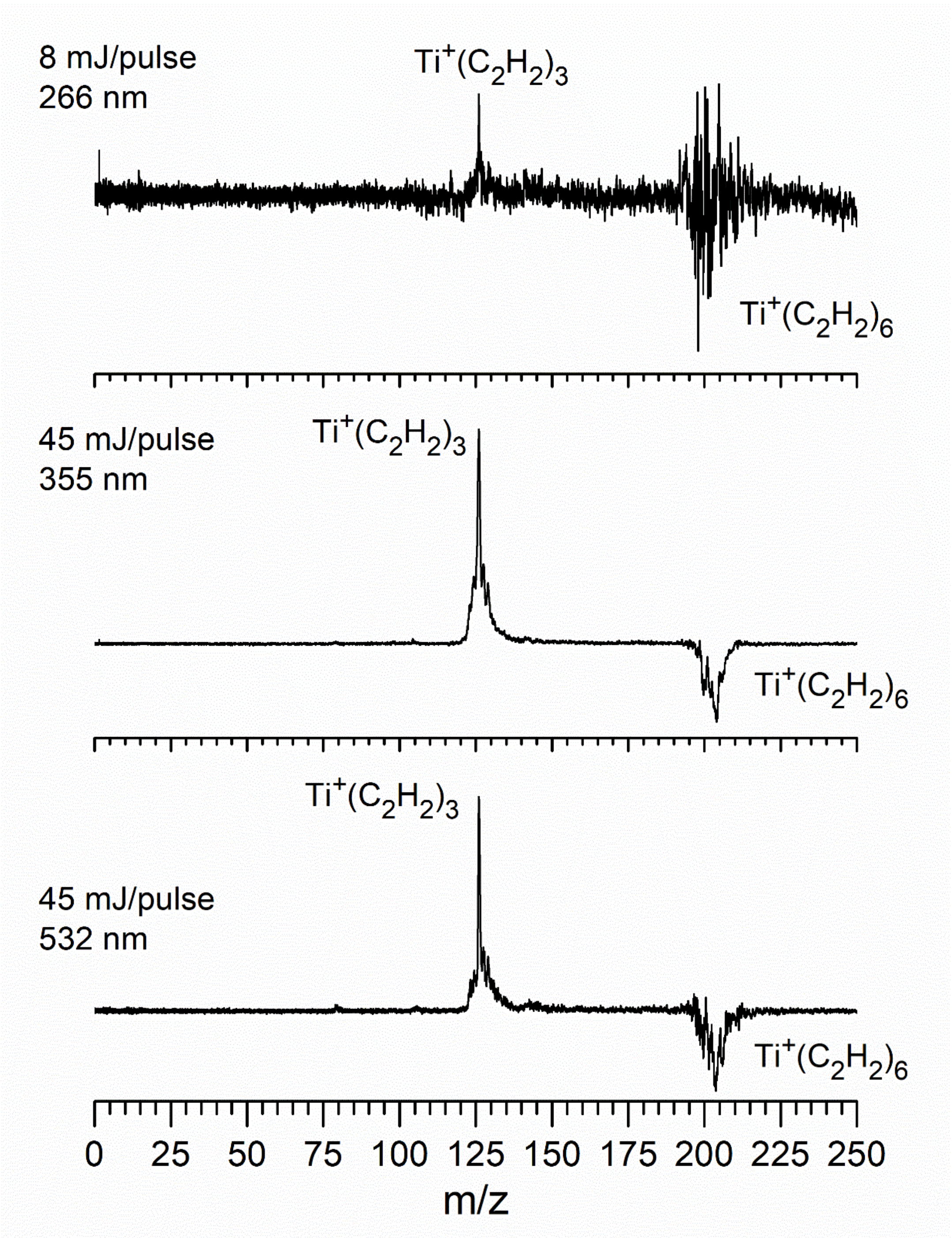

Figure S3. The difference mass spectrum of $Ti^{+}(C_2H_2)_6$ at various wavelengths.

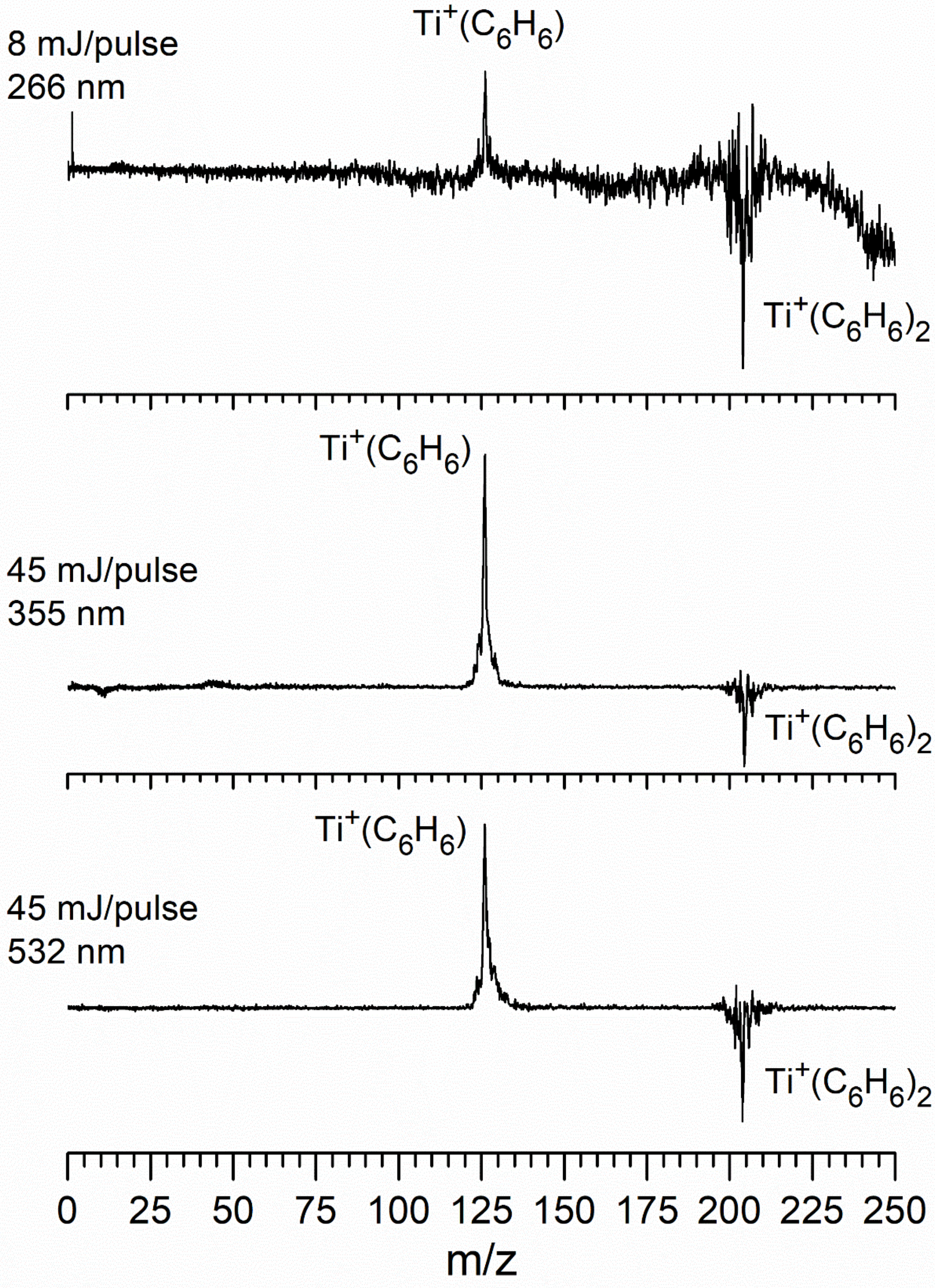

Figure S4. The difference mass spectrum of $Ti^+(C_6H_6)_2$ at various wavelengths.

Table S1. C_2H_2 calculated at the B3LYP/def2-TZVP level of theory using Gaussian16.

2s + 1	E (hartree)	Relative E (kcal/mol)
1	-77.337669	+0.0

Figure S5. The optimized geometry of C_2H_2 followed by its predicted frequencies (cm^{-1}) and IR intensities (km/mol).

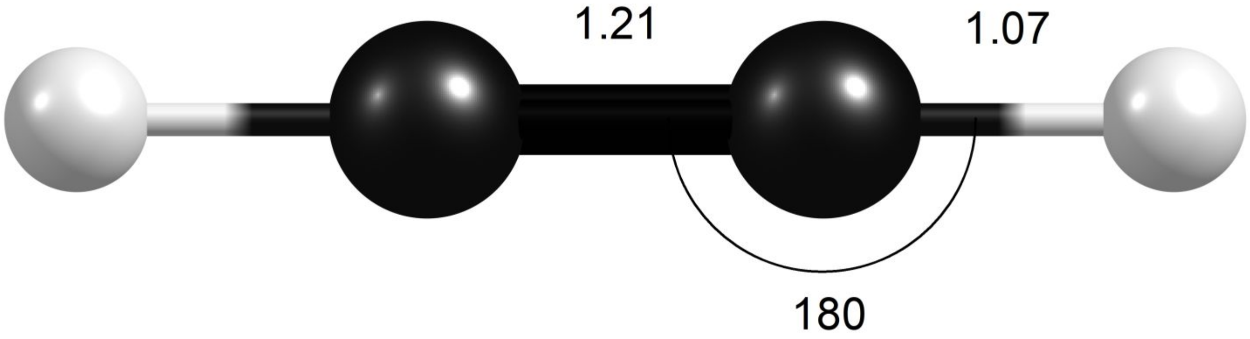

Frequency (cm^{-1})	Intensity (km/mol)
620.1749	0
620.1749	0
764.5617	108.0068
764.5617	108.0068
2072.432	0
3417.5782	90.6379
3515.7371	0

Table S2. Ti^+ electronic energy calculated at the B3LYP/def2-TZVP level.

2s + 1	E (hartree)	Relative E (kcal/mol)	Experimental*
2	-849.105520	+12.7	+13.2
4	-849.125763	0.0	0.0

*Kramida, A.; Ralchenko, Y.; Reader, J.; NIST ASD Team, NIST Atomic Spectra Database (version 5.10). NIST Standard Reference Database Number 78; National Institute of Standards and Technology: Gaithersburg, MD, 2022, Available: https://physics. nist.gov/asd.

Table S3. $Ti^{+}(C_2H_2)$ electronic energy calculated at the B3LYP/def2-TZVP level.

2s + 1	E (hartree)	Relative E (kcal/mol)
2	-926.538288	0.0
4	-926.520344	+11.3

Table S4. $Ti^{+}(C_2H_2)Ar$ electronic energy calculated at the B3LYP/def2-TZVP level.

Isomer	2s + 1	E (hartree)	Relative E (kcal/mol)	Ar BE (kcal/mol)
1i	2	-1454.100058	0.0	7.4
1ii	2	-1454.08873	+7.1	0.3
1i	4	-1454.081424	+11.7	7.0
1ii	4	-1454.071133	+18.2	0.5

Table S5. $Ti^{+}(C_2H_2)Ar_2$ electronic energy calculated at the B3LYP/def2-TZVP level.

Isomer	2s + 1	E (hartree)	Relative E (kcal/mol)	Ar BE (kcal/mol)
1a	2	-1981.661197	0.0	7.0
1b	2	-1981.650426	+6.8	0.3
1c	2	-1981.639124	+13.9	0.3
1a	4	-1981.637123	+15.1	3.6
1b	4	-1981.632130	+18.2	0.5
1c	4	-1981.621944	+24.6	0.5

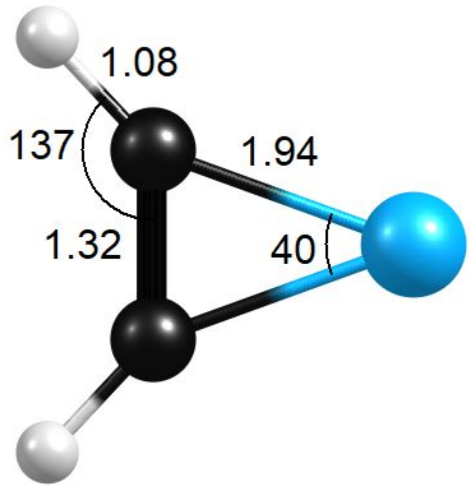

Figure S6. The optimized geometry of doublet $Ti^+(C_2H_2)$ followed by its predicted frequencies (cm^{-1}) and IR intensities (km/mol).

Frequency (cm^{-1})	Intensity (km/mol)
582.0022	54.3315
612.5667	24.9341
705.3148	114.2712
791.2185	2.1319
856.0682	0
1031.4231	97.4008
1494.5062	0.2908
3175.1136	18.4156
3203.4932	26.5971

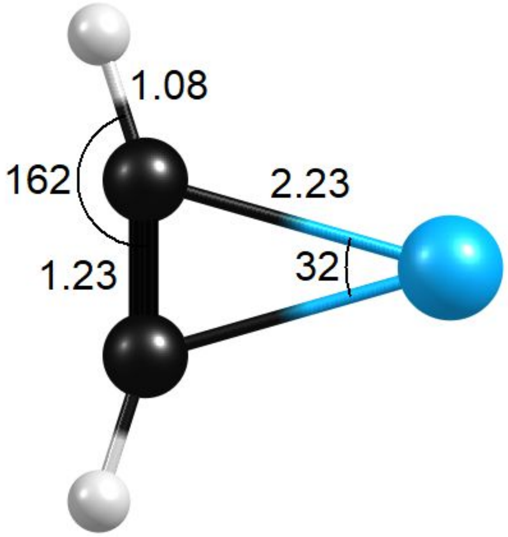

Figure S7. The optimized geometry of quartet $Ti^{+}(C_2H_2)$ followed by its predicted frequencies (cm^{-1}) and IR intensities (km/mol).

Frequency (cm^{-1})	Intensity (km/mol)
345.9313	1.0203
367.074	0.1138
649.2488	0
657.3582	72.4648
688.6122	15.0153
727.6006	119.2353
1857.4796	54.3444
3273.5062	164.1599
3350.4594	78.4153

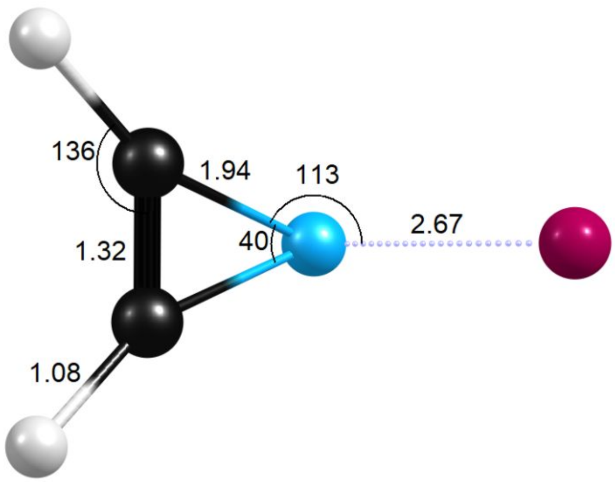

Figure S8. The optimized geometry of isomer 1i-doublet $Ti^{+}(C_2H_2)Ar$ followed by its predicted frequencies (cm^{-1}) and IR intensities (km/mol).

Frequency (cm^{-1})	Intensity (km/mol)
55.9707	4.3293
77.5948	0.8688
157.4869	22.1356
583.1169	53.7546
612.692	25.325
702.5139	118.1588
791.2012	1.5809
891.9894	0.3732
1036.8447	88.2422
1493.5238	0.2822
3174.1356	13.2569
3202.0497	22.1752

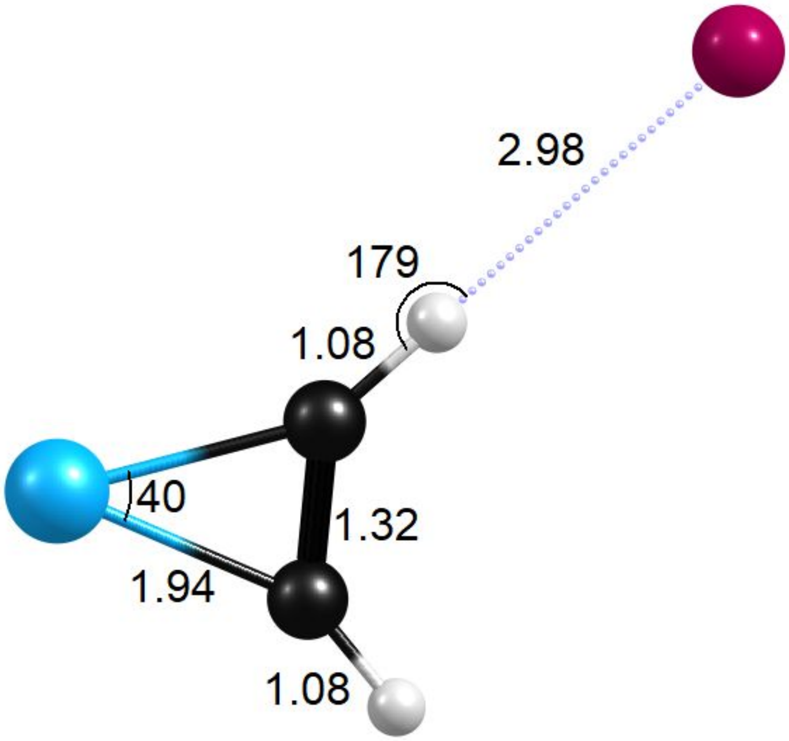

Figure S9. The optimized geometry of isomer 1ii-doublet $Ti^{+}(C_2H_2)Ar$ followed by its predicted frequencies (cm^{-1}) and IR intensities (km/mol).

Frequency (cm^{-1})	Intensity (km/mol)
14.4081	8.3758
17.4308	1.9009
29.4628	3.6303
583.0681	49.9558
612.5873	23.8454
708.8948	104.0622
793.4948	1.2445
856.0912	0.0515
1032.1814	98.6404
1493.8061	0.8067
3173.022	33.8549
3201.6989	38.7599

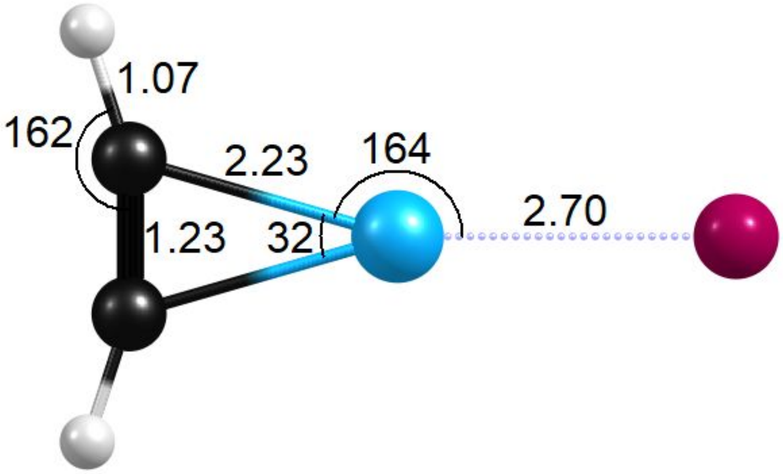

Figure S10. The optimized geometry of isomer 1i-quartet $Ti^{+}(C_2H_2)Ar$ followed by its predicted frequencies (cm^{-1}) and IR intensities (km/mol).

Frequency (cm^{-1})	Intensity (km/mol)
19.0409	3.4915
49.0085	3.1133
133.222	10.1961
351.3372	0.5804
376.4118	0.0009
652.8552	0
654.9955	68.4671
690.8077	16.8184
726.2244	109.9252
1858.5646	63.0388
3275.101	149.9254
3351.4816	77.8669

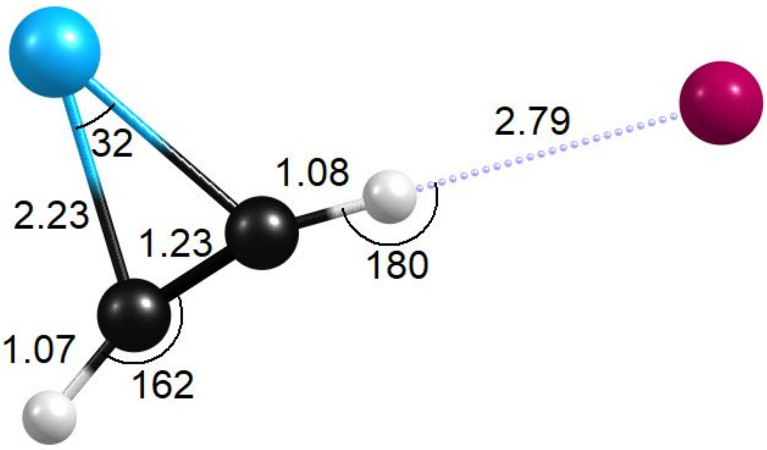

Figure S11. The optimized geometry of isomer 1ii-quartet $Ti^+(C_2H_2)Ar$ followed by its predicted frequencies (cm^{-1}) and IR intensities (km/mol).

Frequency (cm^{-1})	Intensity (km/mol)
14.8054	5.69
42.8231	0.2874
44.0168	2.4143
344.6699	1.1463
368.2553	0.0064
655.6106	3.0029
661.2035	71.3156
695.7339	23.0119
737.485	100.0492
1858.9182	48.6347
3265.9585	250.6038
3345.7539	96.6083

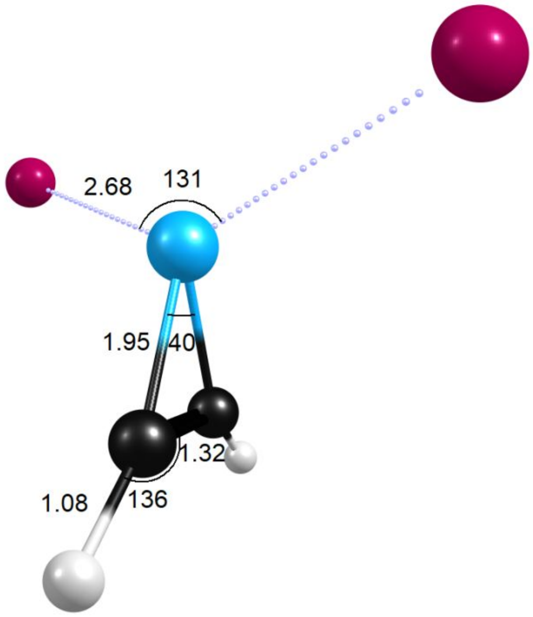

Figure S12. The optimized geometry of argon tagged isomer 1a-doublet $Ti^+(C_2H_2)Ar_2$ followed by its predicted frequencies (cm^{-1}) and IR intensities (km/mol).

Frequency (cm^{-1})	Intensity (km/mol)
31.4428	0.9534
61.8201	5.7107
64.1909	1.5608
86.6958	0
121.0134	3.7045
182.3135	33.2573
583.8601	54.5416
612.6399	25.3889
699.8755	122.2895
791.1205	0.3822
902.203	0
1042.4119	85.9332
1492.5226	0.2453
3172.5189	8.857
3200.0843	17.903

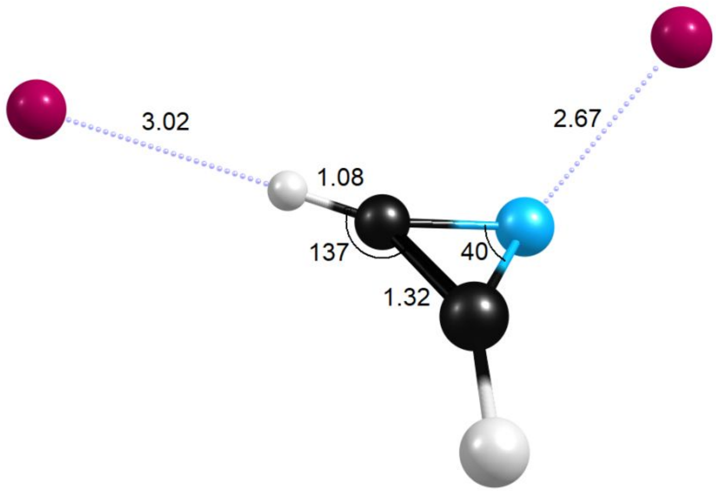

Figure S13. The optimized geometry of argon tagged isomer 1b-doublet $Ti^{+}(C_2H_2)Ar_2$ followed by its predicted frequencies (cm^{-1}) and IR intensities (km/mol).

Frequency (cm^{-1})	Intensity (km/mol)
8.179	0.0841
15.4968	7.1344
26.7441	2.4131
56.8972	4.6457
80.8385	0.872
157.2168	22.4397
584.0798	49.8317
612.6975	24.3623
705.7305	108.5325
793.2149	1.0826
894.6532	0.4133
1037.5396	89.1708
1492.9534	0.4114
3172.7531	24.41
3200.7688	33.0152

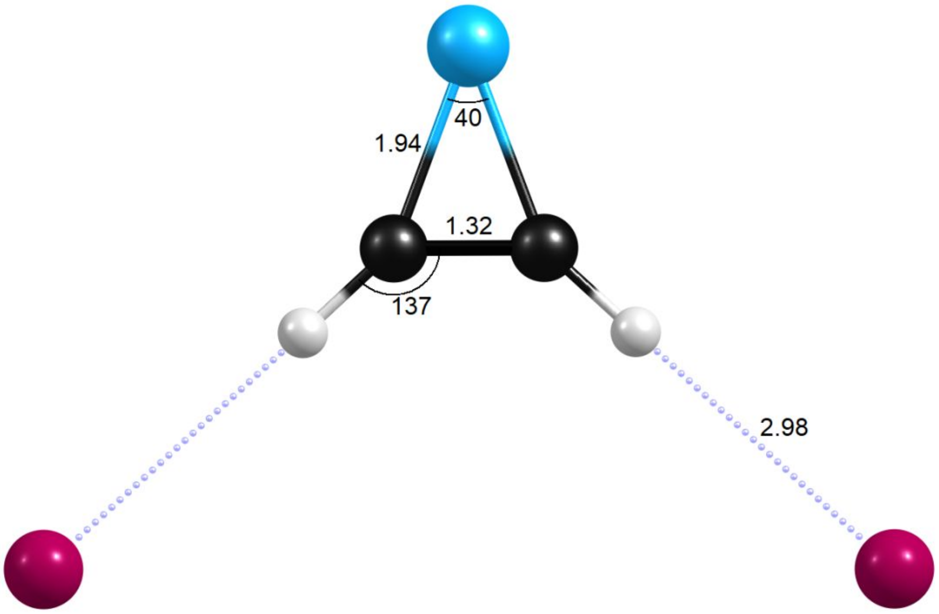

Figure S14. The optimized geometry of argon tagged isomer 1c-doublet $Ti^{+}(C_2H_2)Ar_2$ followed by its predicted frequencies (cm^{-1}) and IR intensities (km/mol).

Frequency (cm^{-1})	Intensity (km/mol)
4.5288	2.0215
18.7909	12.3284
19.2787	6.9451
28.193	5.7729
31.2812	0.5028
31.7815	0.0005
584.2108	45.5827
612.6105	22.8268
712.9143	94.1138
795.9081	0.5318
860.9081	0
1033.0628	99.1045
1493.1286	1.2783
3171.2631	45.38
3199.8524	53.4601

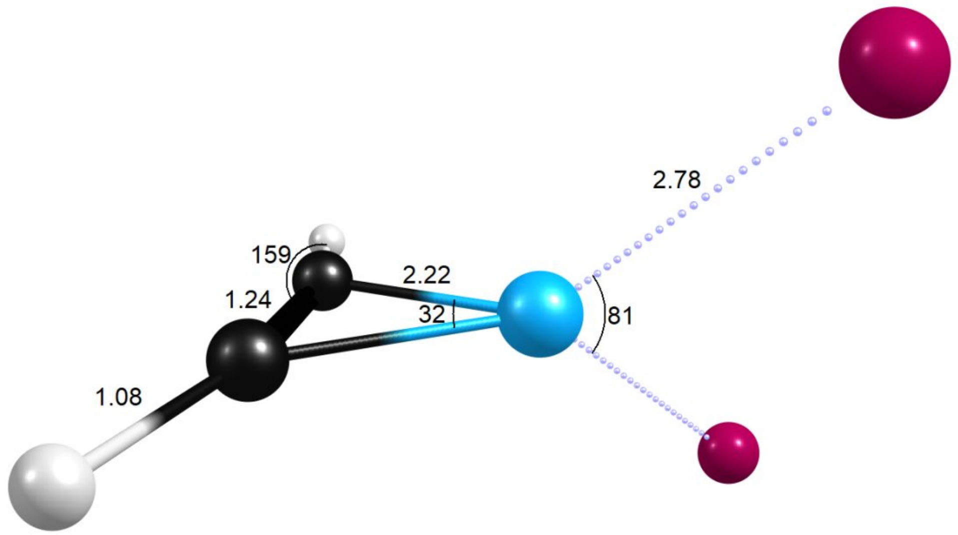

Figure S15. The optimized geometry of argon tagged isomer 1a-quartet $Ti^+(C_2H_2)Ar_2$ followed by its predicted frequencies (cm^{-1}) and IR intensities (km/mol).

Frequency (cm^{-1})	Intensity (km/mol)
52.1646	0.07
57.7864	1.4423
68.0529	0
70.6028	2.0242
112.9208	10.8217
119.2657	13.0143
377.4247	0.0007
385.9804	0.0756
656.4592	80.929
662.9248	0
682.1268	20.5609
716.0542	93.5131
1828.8041	71.7478
3261.5761	115.1307
3333.0669	67.9915

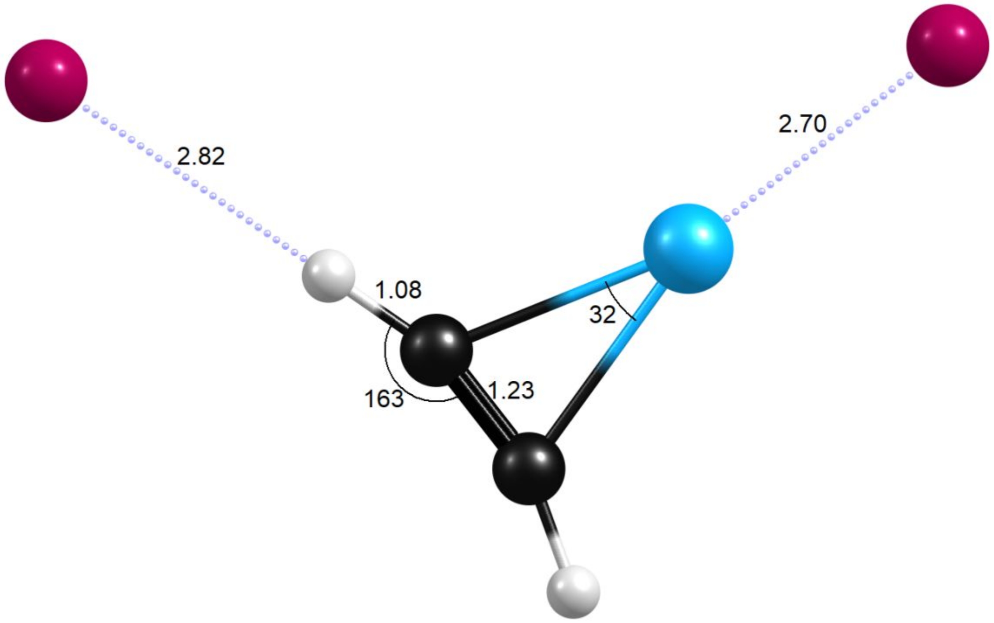

Figure S16. The optimized geometry of argon tagged isomer 1b-quartet $Ti^{+}(C_2H_2)Ar_2$ followed by its predicted frequencies (cm^{-1}) and IR intensities (km/mol).

Frequency (cm^{-1})	Intensity (km/mol)
8.5617	0.9555
19.8374	3.4679
37.4474	5.9404
41.7277	0.3038
54.3131	0.9053
132.9334	10.1718
349.9677	0.6748
377.2785	0.0803
658.6219	68.1663
659.102	2.7892
696.8755	23.7588
735.6216	92.4976
1859.8431	56.8559
3268.9442	225.4001
3347.5373	96.093

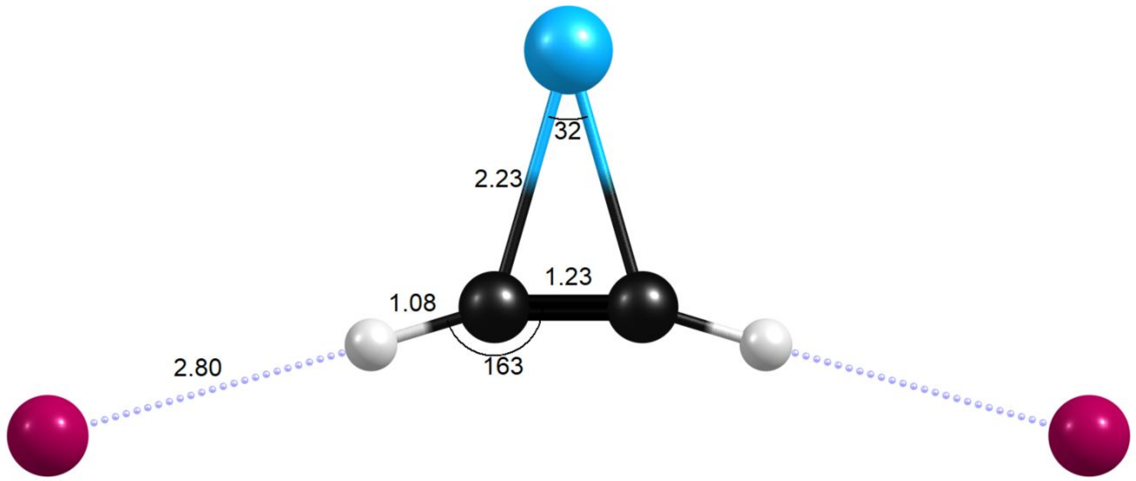

Figure S17. The optimized geometry of argon tagged isomer 1c-quartet $Ti^+(C_2H_2)Ar_2$ followed by its predicted frequencies (cm^{-1}) and IR intensities (km/mol).

Frequency (cm^{-1})	Intensity (km/mol)
6.6256	4.6183
16.1494	0.4388
17.2945	7.9271
31.8742	1.7048
53.7661	2.8107
59.4752	0
343.387	1.2441
369.3035	0.0638
664.4614	0
666.933	76.2519
700.3968	26.4746
745.2265	87.5366
1860.4354	42.819
3260.4664	330.1388
3340.167	118.4524

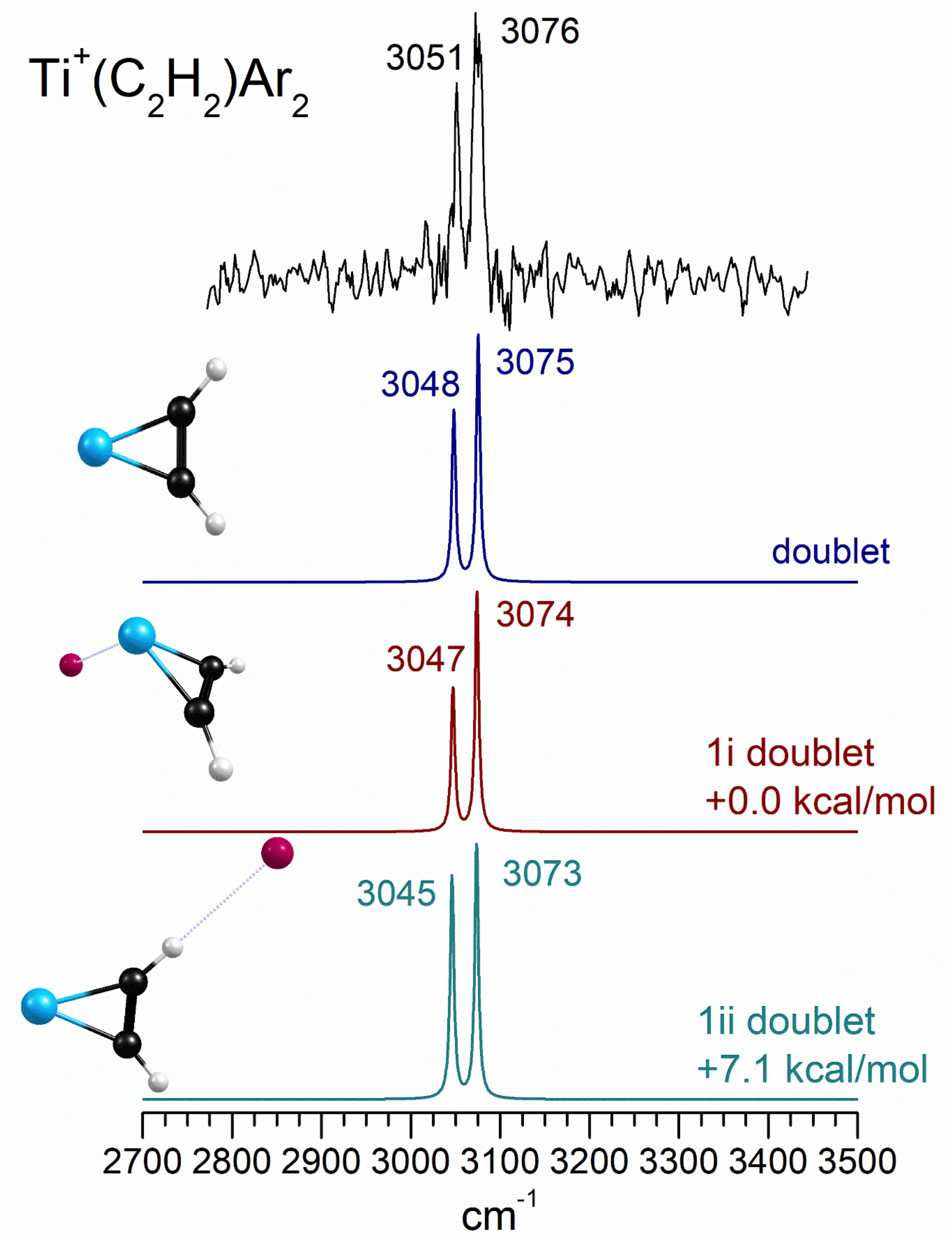

Figure S18. The experimental spectrum measured for $Ti^+(C_2H_2)Ar_2$ with simulated spectra for the doublet and singly-tagged doublet isomers.

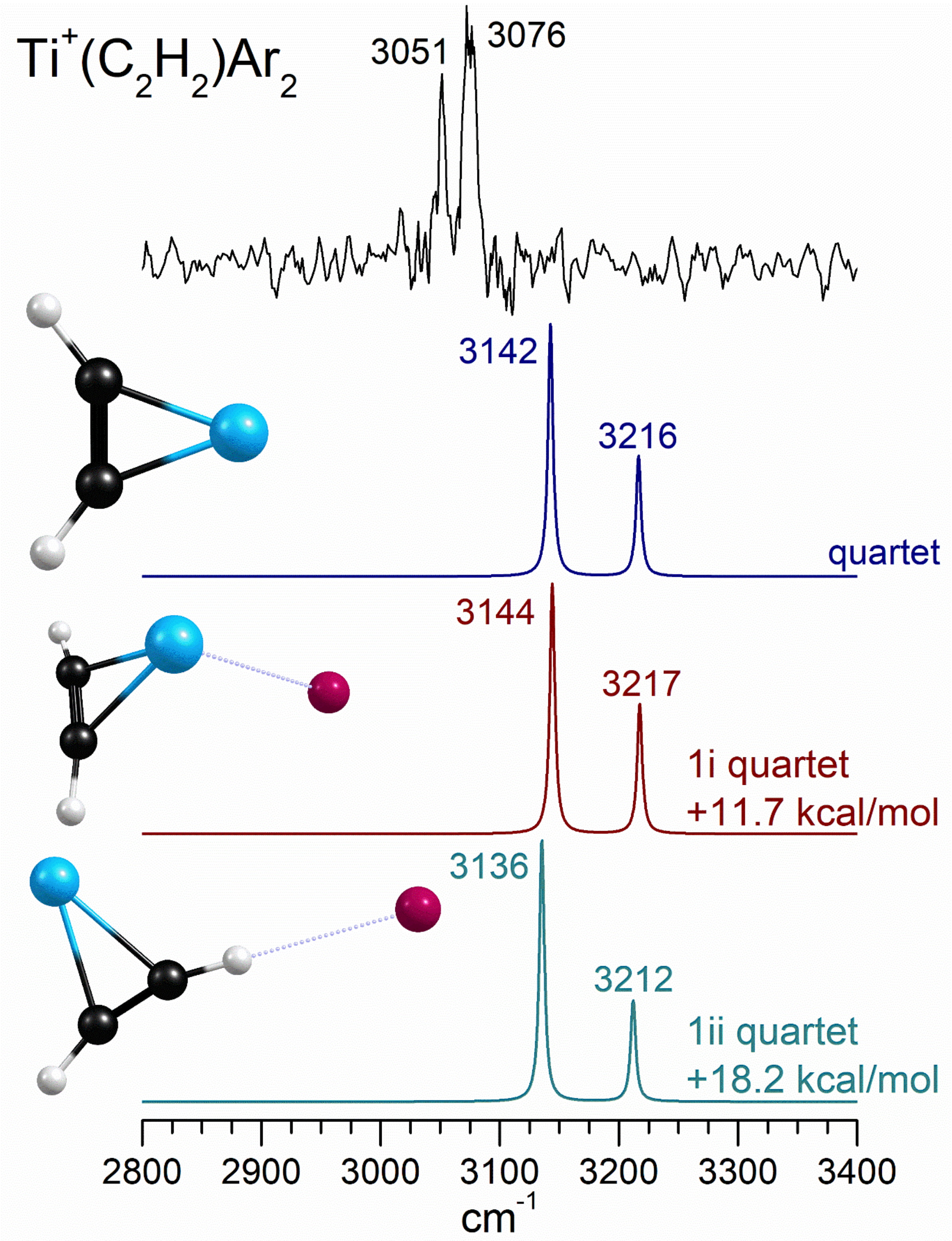

Figure S19. The experimental spectrum measured for $Ti^{+}(C_2H_2)Ar_2$ with simulated spectra for the quartet and singly-tagged quartet isomers.

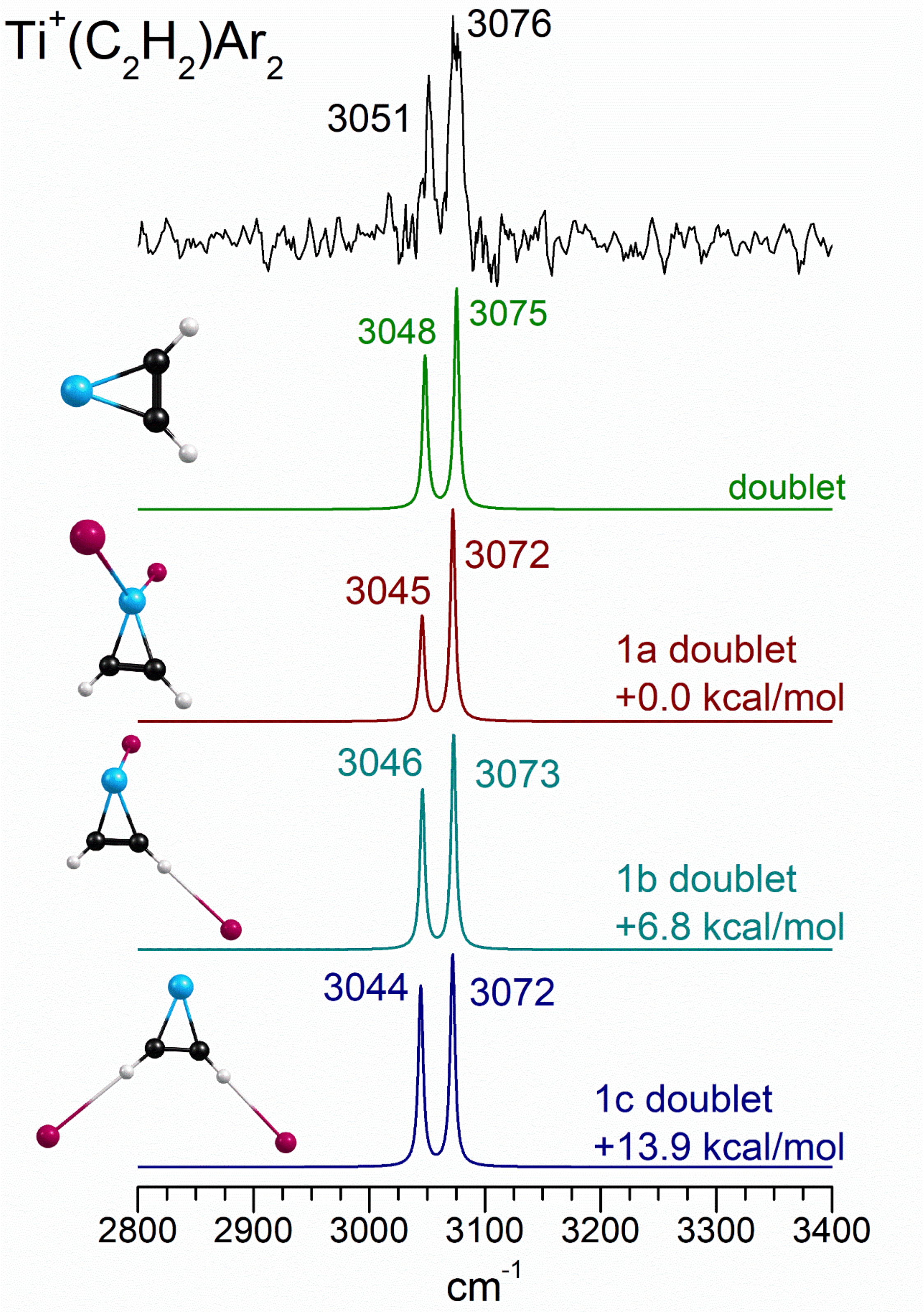

Figure S20. The experimental spectrum measured for $Ti^+(C_2H_2)Ar_2$ with simulated spectra for the doublet and doubly-tagged doublet isomers.

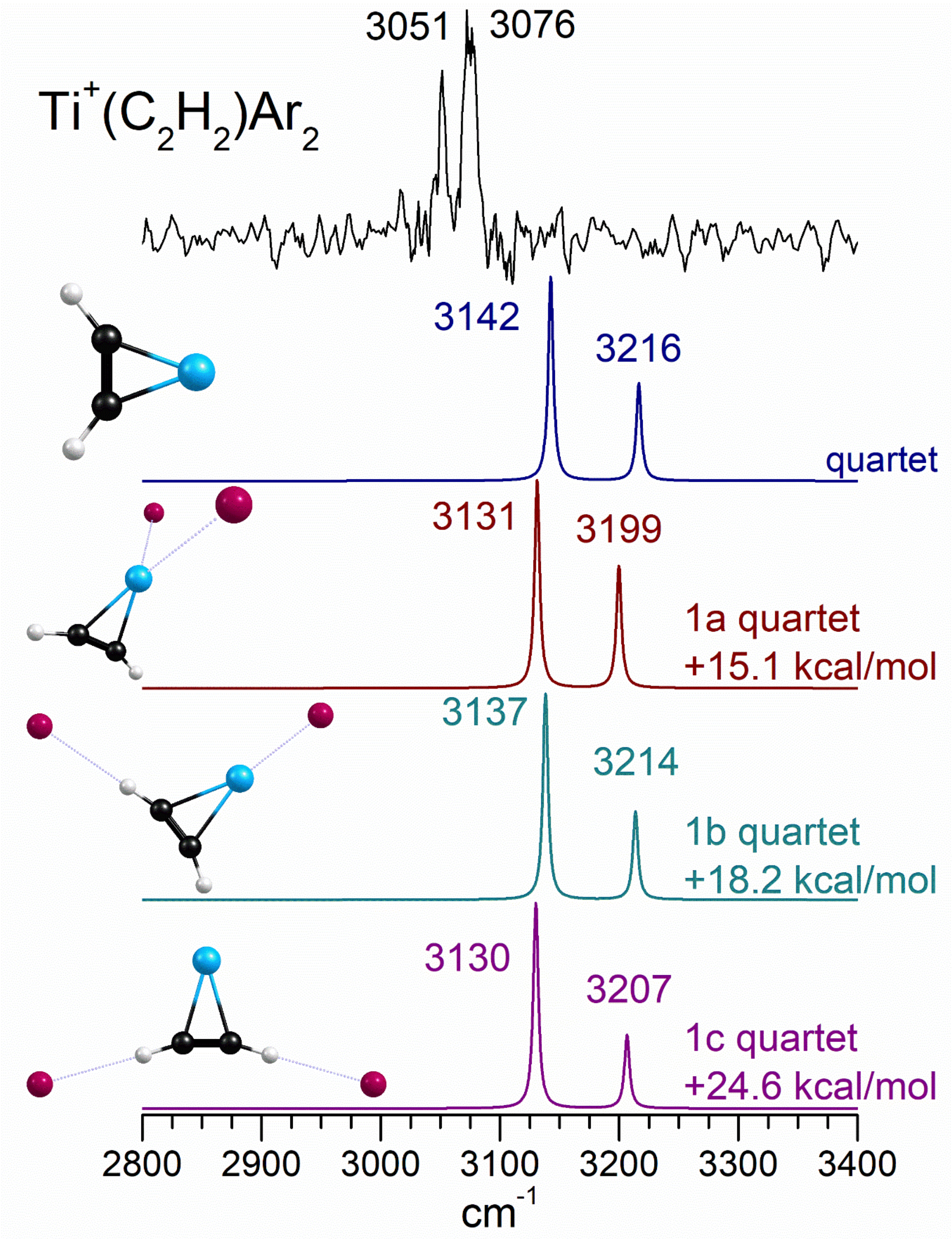

Figure S21. The experimental spectrum measured for $Ti^+(C_2H_2)Ar_2$ with simulated spectra for the quartet and doubly-tagged quartet isomers.

Table S6. $Ti^+(C_2H_2)_2$ electronic energy calculated at the B3LYP/def2-TZVP level.

Isomer	2s + 1	E (hartree)	Relative E (kcal/mol)
2a	2	-1003.949568	0.0
2b	2	-1003.939542	+6.3
2c	2	-1003.92452	+15.7
2a	4	-1003.905443	+27.7
2b	4	-1003.918417	+19.5
2c	4	-1003.898632	+32.0
TS1	2	-1003.922778	+16.8
SOSP	2	-1003.836059	+71.22
TS2	2	-1003.898427	+32.1
TS1	4	-1003.858049	+57.4
SOSP	4	-1003.821222	+80.5
TS2	4	-1003.885135	+40.4

Table S7. $Ti^+(C_2H_2)_2Ar$ electronic energy calculated at the B3LYP/def2-TZVP level.

Isomer	2s + 1	E (hartree)	Relative E (kcal/mol)
2ai	2	-1531.511552	0.0
2aii	2	-1531.500109	+7.2
2ai	4	-1531.465388	+29.0
2aii	4	-1531.455904	+34.9
2aiii	4	-1531.455869	+34.9
2bi	2	-1531.500865	0.0
2bi	4	-1531.478965	+13.7
2bii	4	-1531.468896	+20.1
2ci	2	-1531.485335	0.0
2cii	2	-1531.474875	+6.6
2ci	4	-1531.459662	+16.1
2cii	4	-1531.449456	+22.5

Table S8. $Ti^{+}(C_2H_2)_2Ar_2$ electronic energy calculated at the B3LYP/def2-TZVP level.

Isomer	2s + 1	E (hartree)	Relative E (kcal/mol)
2aI	2	-2059.070589	0.0
2aII	2	-2059.062055	+5.4
2aIII	2	-2059.050445	+12.6
2aI	4	-2059.022225	+30.3
2aII	4	-2059.015807	+34.4
2aIII	4	-2059.006329	+40.3
2bI	2	-2059.058331	0.0
2bI	4	-2059.036619	+13.6
2bII	4	-2059.029345	+18.2
2bIII	4	-2059.019302	+24.5
2cI	2	-2059.039131	0.0
2cII	2	-2059.035656	+2.2
2cIII	2	-2059.02582	+8.4
2cI	4	-2059.011414	+17.4
2cIII	4	-2059.000266	+24.4

Figure S22. The optimized geometry of isomer 2a-doublet $Ti^{+}(C_2H_2)_2$ followed by its predicted frequencies (cm^{-1}) and IR intensities (km/mol).

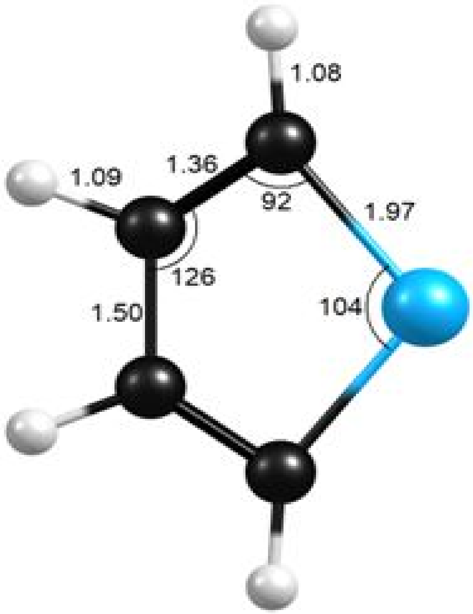

Frequency (cm^{-1})	Intensity (km/mol)
104.646	19.3957
247.724	0
250.5437	9.5586
469.5287	11.5138
626.8079	134.1309
689.0309	11.1599
752.3027	83.9582
843.1683	0
863.2902	6.9282
981.1716	0.0006
1026.8603	0
1097.1001	32.6062
1116.5687	6.9184
1302.2865	40.4386
1340.0929	28.9782
1439.8107	6.2693
1568.3579	1.737
3117.4222	0.0183
3130.3942	0.1068
3187.5327	1.9163
3187.6203	24.6431

Figure S23. The optimized geometry of isomer 2b-doublet $Ti^{+}(C_2H_2)_2$ followed by its predicted frequencies (cm^{-1}) and IR intensities (km/mol).

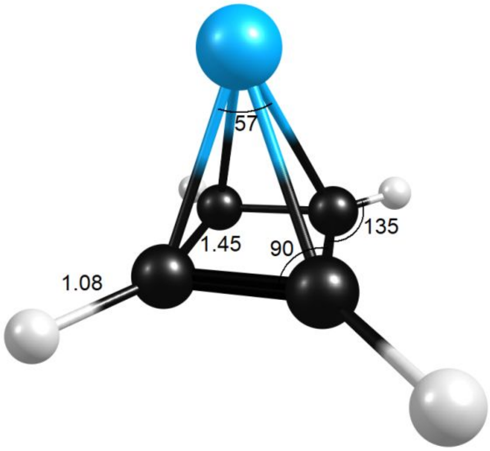

Frequency (cm^{-1})	Intensity (km/mol)
277.9838	2.8276
277.9843	2.8276
466.4769	0.3245
656.536	0
662.7495	29.2504
662.7501	29.2504
801.6731	0
812.8902	102.4746
936.7843	28.3527
936.7847	28.3525
951.0179	0
952.7061	0
1185.04	0
1195.6976	0
1250.0981	0.0627
1327.8192	3.2351
1327.8197	3.2351
3205.6382	0
3222.2585	14.1139
3222.2595	14.1139
3239.7024	4.0976

Figure S24. The optimized geometry of isomer 2c-doublet $Ti^{+}(C_2H_2)_2$ followed by its predicted frequencies (cm^{-1}) and IR intensities (km/mol).

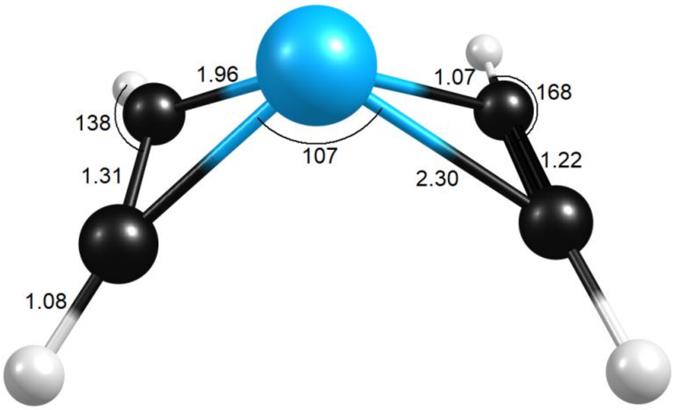

Frequency (cm^{-1})	Intensity (km/mol)
122.3796	2.8814
131.5875	0.1667
170.0818	0.1866
271.8573	7.6893
346.43	5.0925
582.3887	17.0221
583.044	2.2708
629.3763	8.2865
668.6871	39.0705
692.7087	43.0717
709.1662	57.0987
753.6762	118.9925
790.463	8.4818
894.3997	0.0026
1013.4288	89.8685
1532.1885	20.3144
1918.6005	44.9285
3177.6581	10.4419
3208.1062	25.2292
3299.9052	176.1161
3385.2079	92.3733

Figure S25. The optimized geometry of isomer 2a-quartet $Ti^{+}(C_2H_2)_2$ followed by its predicted frequencies (cm^{-1}) and IR intensities (km/mol).

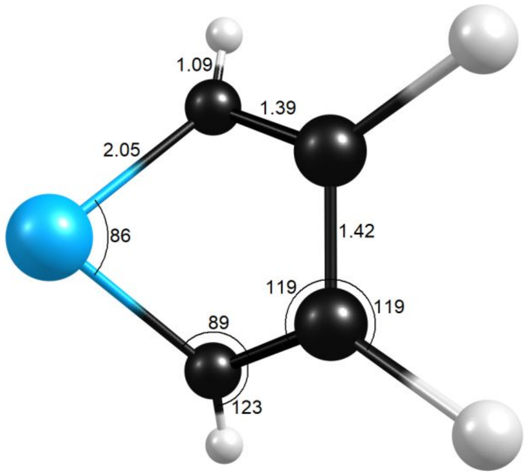

Frequency (cm^{-1})	Intensity (km/mol)
204.2461	3.8119
269.4216	3.8039
298.8852	34.4745
433.887	0.5063
531.3195	16.3169
650.909	25.3092
664.1478	88.3574
724.0493	25.8
909.8665	37.6801
910.9524	71.5898
962.6902	20.3444
1059.6057	3.6415
1075.1608	16.8728
1155.3026	56.1747
1175.979	12.1598
1428.7297	15.2292
1441.5534	12.6254
3105.6496	0.6906
3117.7882	0.2082
3170.1593	15.6923
3170.2985	3.1138

Figure S26. The optimized geometry of isomer 2b-quartet $Ti^+(C_2H_2)_2$ followed by its predicted frequencies (cm^{-1}) and IR intensities (km/mol).

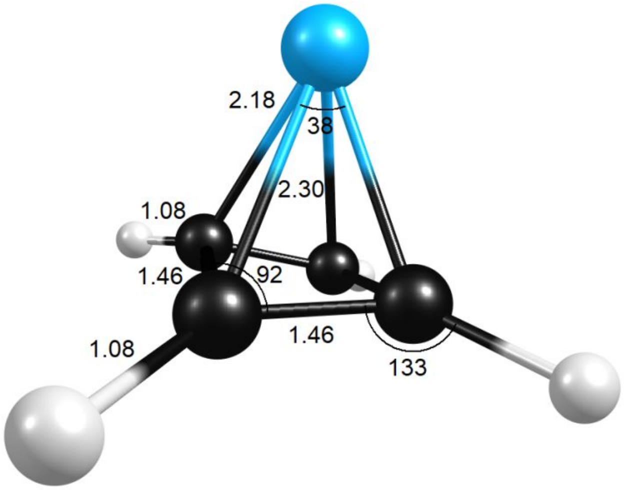

Frequency (cm^{-1})	Intensity (km/mol)
285.9602	8.702
359.614	1.4669
415.8806	4.6259
570.9414	0
581.6894	22.3486
675.4004	96.1063
753.6638	12.2053
762.44	27.4927
853.7467	1.2064
901.0307	3.8958
945.7459	35.7159
952.8314	12.9528
1055.6696	0
1187.995	0
1226.6268	1.7549
1282.0128	28.0015
1341.1345	1.2251
3212.3139	0.8703
3217.6707	14.1183
3244.2991	25.5931
3250.7182	4.973

Figure S27. The optimized geometry of isomer 2c-quartet $Ti^{+}(C_2H_2)_2$ followed by its predicted frequencies (cm^{-1}) and IR intensities (km/mol).

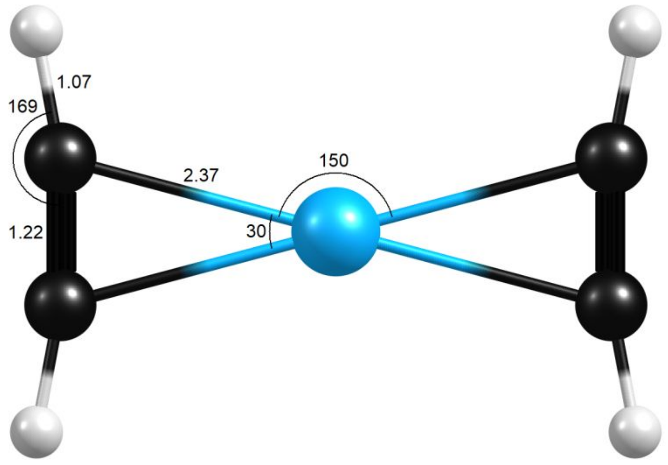

Frequency (cm^{-1})	Intensity (km/mol)
17.0475	0.1432
81.4341	0.9785
141.408	0
175.439	76.899
249.9884	0
291.4358	0
294.4755	0.1603
625.6857	0
626.3561	0
640.157	0
647.8358	55.8803
682.0212	25.877
750.4802	0
752.8123	174.9843
769.4529	0
1939.6525	150.5642
1959.3925	0
3319.874	0
3320.5376	391.1645
3404.1244	257.7634
3412.2901	0

Figure S28. The optimized geometry of the TS1 doublet $Ti^+(C_2H_2)_2$ followed by its predicted frequencies (cm^{-1}) and IR intensities (km/mol).

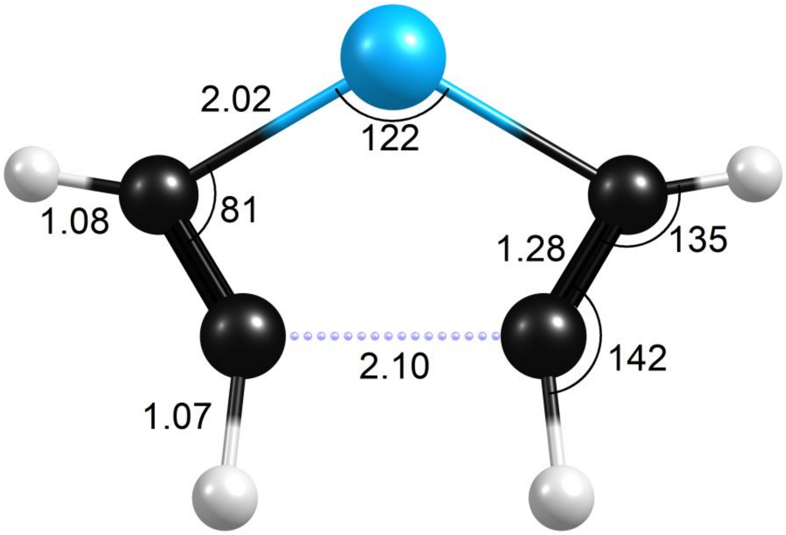

Frequency (cm^{-1})	Intensity (km/mol)
-278.6576	5.1277
151.007	5.0178
156.5412	0.0263
396.0718	24.2674
506.2204	18.0657
592.3909	8.954
647.0191	84.7077
661.6222	151.9042
732.057	8.4383
803.0084	100.1079
833.9313	0.9418
851.3362	6.4512
882.4576	6.0445
1061.6781	1.2563
1082.4393	38.0505
1613.0931	8.5557
1671.5251	55.2836
3223.5553	65.4675
3224.3228	46.9744
3295.7862	10.7941
3301.9203	20.3992

Figure S29. The optimized geometry of the SOSP doublet $Ti^{+}(C_2H_2)_2$ followed by its predicted frequencies (cm^{-1}) and IR intensities (km/mol).

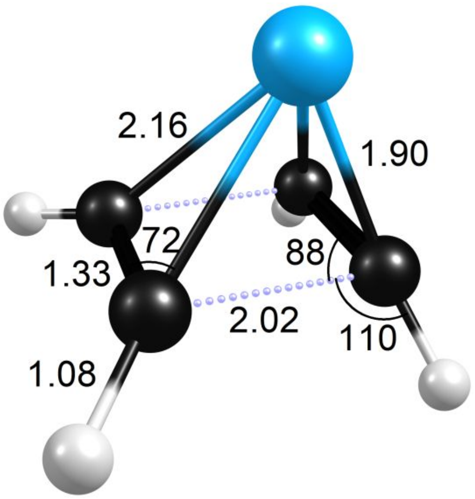

Frequency (cm^{-1})	Intensity (km/mol)
-748.657	21.6481
-428.7728	0.607
150.2939	2.9764
442.5573	19.7807
473.8667	16.986
570.8831	21.2774
633.3721	47.815
633.7125	56.0541
758.1178	39.7074
857.993	0.2253
868.7852	61.9175
910.7858	4.2225
928.6389	1.0392
1029.3028	18.902
1082.7001	70.964
1120.9118	12.3202
1501.2966	6.2669
3160.9326	8.2307
3185.8431	14.6002
3191.1426	9.7522
3199.6843	18.7784

Figure S30. The optimized geometry of the TS2 doublet $Ti^+(C_2H_2)_2$ followed by its predicted frequencies (cm^{-1}) and IR intensities (km/mol).

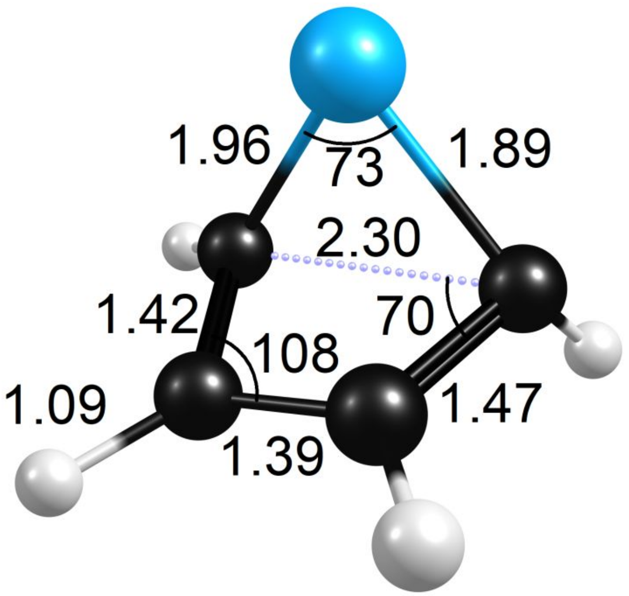

Frequency (cm^{-1})	Intensity (km/mol)
-313.0913	2.0207
274.5175	5.2858
329.4273	1.1325
539.5166	11.6658
597.2398	42.9896
672.2742	24.2325
718.9225	51.1677
771.4188	19.8318
814.2822	38.0503
917.6905	66.3392
956.8343	21.2521
1018.5729	1.3937
1044.0327	22.2425
1088.4057	5.6478
1156.8996	33.787
1358.3269	13.419
1425.4878	3.211
3133.5264	0.4398
3161.9621	13.1666
3167.9946	22.1804
3179.1326	3.7119

Figure S31. The optimized geometry of the TS1 quartet $Ti^+(C_2H_2)_2$ followed by its predicted frequencies (cm^{-1}) and IR intensities (km/mol).

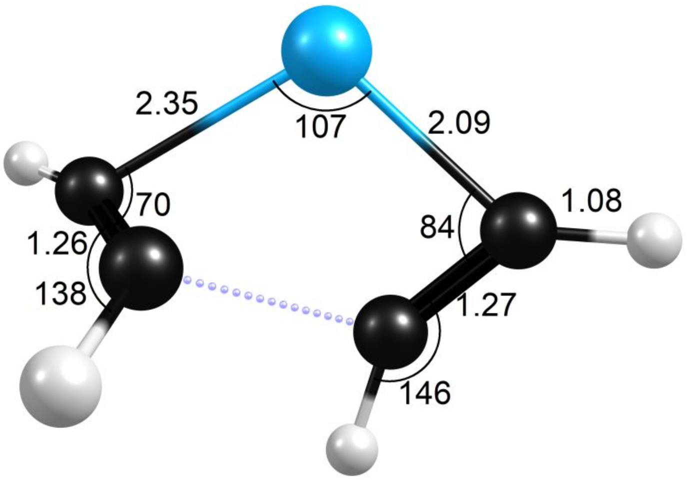

Frequency (cm^{-1})	Intensity (km/mol)
-507.6078	109.7088
87.8781	3.8656
179.5084	5.4679
272.6228	15.5757
337.113	15.105
390.4034	2.29
551.338	33.6451
581.0548	6.3369
655.8897	78.5566
691.102	90.7611
708.669	54.8489
752.213	39.0465
794.31	16.9737
908.735	31.5684
1062.1747	2.737
1605.5708	53.5288
1725.9126	40.5803
3169.4845	38.6223
3206.2483	60.8489
3256.9832	56.7633
3316.1841	95.5119

Figure S32. The optimized geometry of the SOSP quartet $Ti^{+}(C_2H_2)_2$ followed by its predicted frequencies (cm^{-1}) and IR intensities (km/mol).

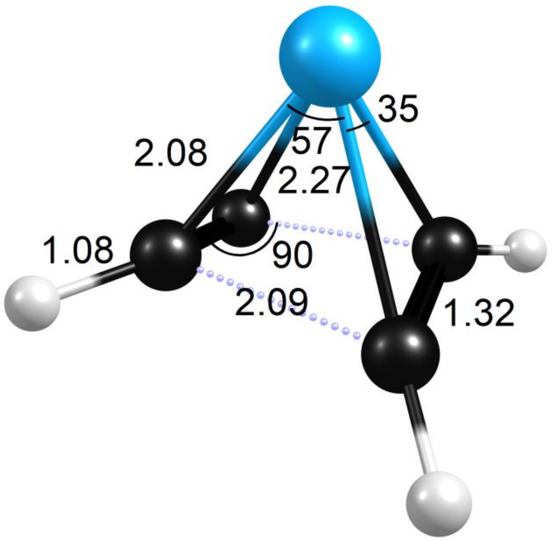

Frequency (cm^{-1})	Intensity (km/mol)
-634.0834	24.3185
-447.7507	21.3149
274.8718	5.9789
298.2734	2.1188
468.0058	6.1786
468.0847	8.3828
518.8105	52.4674
616.9752	26.1151
669.2986	10.4512
737.5496	48.0075
784.2378	21.6241
861.7434	8.9987
908.6573	0.083
945.0012	13.5488
992.5612	125.7555
1449.4435	28.9029
1456.2295	0.034
3168.7963	0.3869
3169.619	22.79
3243.7355	13.601
3244.5181	46.0555

Figure S33. The optimized geometry of the TS2 quartet $Ti^{+}(C_2H_2)_2$ followed by its predicted frequencies (cm^{-1}) and IR intensities (km/mol).

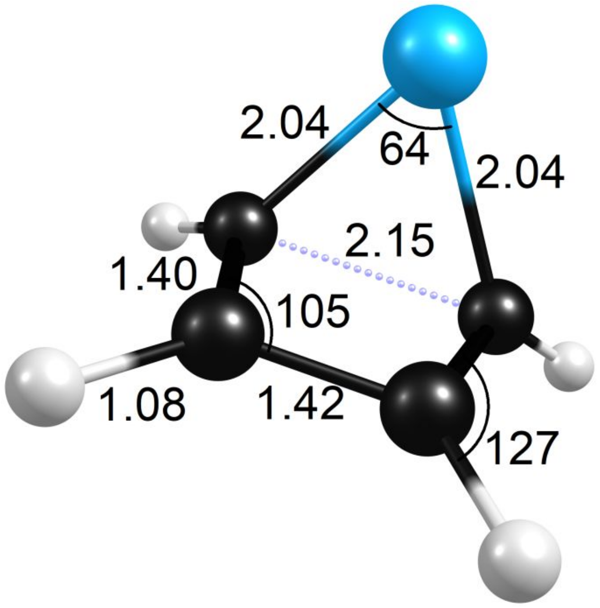

Frequency (cm^{-1})	Intensity (km/mol)
-565.7313	1.1247
235.0134	2.9996
320.8166	0.9069
400.0291	5.1307
574.7793	10.5753
583.384	8.6526
685.4517	58.2086
768.0848	31.2414
835.1135	22.6286
878.2123	4.5182
979.3116	60.6084
998.7591	0.3429
1056.7116	2.9694
1118.5335	50.6319
1236.9948	17.2251
1371.444	9.643
1410.8469	2.0387
3159.0964	6.2077
3165.2483	12.5133
3176.9337	15.5177
3187.828	3.2818

Table S9. Cartesian coordinates for the optimized geometry of isomer 2ai-doublet $Ti^{+}(C_2H_2)_2Ar$ followed by its predicted frequencies (cm^{-1}) and IR intensities (km/mol).

Z	x	y	z
22	-0.036594000	0.000002000	0.368501000
6	1.120375000	1.557692000	0.002063000
6	2.177089000	0.750800000	-0.260515000
6	2.177084000	-0.750805000	-0.260514000
6	1.120367000	-1.557694000	0.002065000
1	1.197355000	2.637312000	-0.032887000
1	3.152345000	1.164635000	-0.507159000
1	3.152336000	-1.164646000	-0.507166000
1	1.197345000	-2.637313000	-0.032884000
18	-2.636878000	0.000000000	-0.218084000

Frequency (cm^{-1})	Intensity (km/mol)
40.4592	1.1694
45.653	1.0457
94.6982	11.2347
139.5632	13.7818
244.6563	0.0141
271.7952	21.6983
471.5061	10.318
628.8842	120.2604
684.739	18.9712
751.9926	76.4203
842.7426	0.0326
854.8966	8.0415
984.0503	0.335
1023.8666	0.1301
1095.4969	32.8124
1116.2875	6.2575
1302.9421	38.1953
1342.8768	26.538
1438.0481	8.8092
1571.3028	1.4063
3116.183	0.0716
3129.6309	0.0039
3188.7433	2.3527
3189.0246	18.2823

Table S10. Cartesian coordinates for the optimized geometry of isomer 2aii-doublet $Ti^{+}(C_2H_2)_2Ar$ followed by its predicted frequencies (cm^{-1}) and IR intensities (km/mol).

Z	x	y	z
22	-2.428818000	-0.000002000	0.000083000
6	-1.211384000	-1.550320000	-0.000045000
6	-0.116784000	-0.749046000	-0.000141000
6	-0.116789000	0.749050000	-0.000141000
6	-1.211391000	1.550320000	-0.000046000
1	-1.138438000	-2.630977000	-0.000059000
1	0.887455000	-1.167143000	-0.000230000
1	0.887448000	1.167152000	-0.000228000
1	-1.138448000	2.630977000	-0.000057000
18	3.881893000	0.000000000	0.000055000

Frequency (cm^{-1})	Intensity (km/mol)
14.0583	1.993
15.5775	0.2142
27.0345	3.961
105.106	17.612
247.4037	0
252.1732	9.2566
470.1808	11.9291
628.0823	127.73
689.1295	10.6972
751.9858	81.9916
843.5706	0
862.3327	6.9639
983.1971	0.0861
1026.7862	0
1096.9669	26.792
1116.3869	7.1083
1301.176	43.6132
1339.8139	43.356
1439.2314	4.7273
1568.8974	1.5125
3119.4202	0.1137
3131.1387	1.3905
3187.584	2.2156
3187.6925	23.6789

Table S11. Cartesian coordinates for the optimized geometry of isomer 2bi-doublet $Ti^{+}(C_2H_2)_2Ar$ followed by its predicted frequencies (cm^{-1}) and IR intensities (km/mol).

Z	x	y	z
6	-1.839984000	1.022040000	-0.078005000
6	-1.840017000	0.077988000	1.022019000
6	-1.839983000	-1.022039000	0.077969000
6	-1.839957000	-0.077985000	-1.022057000
1	-1.925473000	-0.159909000	-2.095699000
1	-1.925532000	2.095679000	-0.159930000
1	-1.925582000	0.159912000	2.095657000
1	-1.925556000	-2.095676000	0.159890000
22	0.066653000	-0.000002000	0.000031000
18	2.799745000	0.000001000	-0.000008000

Frequency (cm^{-1})	Intensity (km/mol)
41.0379	10.1842
41.0382	10.1844
127.627	8.927
292.9193	4.3234
292.9237	4.3221
469.5216	0.2155
653.2262	28.0134
653.2272	28.007
659.8856	0
798.4177	0
807.8228	104.0041
936.1551	29.5571
936.1579	29.5552
951.2664	0
952.8783	0
1184.584	0
1196.4407	0
1250.6065	0.7129
1328.6495	1.6322
1328.6527	1.6321
3206.0494	0
3222.6827	11.013
3222.6848	11.0138
3240.3297	5.1545

Table S12. Cartesian coordinates for the optimized geometry of isomer 2ci-doublet $Ti^{+}(C_2H_2)_2Ar$ followed by its predicted frequencies (cm^{-1}) and IR intensities (km/mol).

Z	x	y	z
22	-0.240245000	-0.030332000	-0.000002000
6	-0.782260000	-1.801258000	-0.656247000
6	-0.782259000	-1.801255000	0.656251000
1	-1.000824000	-2.495334000	-1.458311000
1	-1.000829000	-2.495328000	1.458317000
6	-1.893581000	1.426515000	-0.611051000
6	-1.893577000	1.426517000	0.611052000
1	-2.088717000	1.577991000	-1.655287000
1	-2.088699000	1.578004000	1.655289000
18	2.420807000	0.388826000	0.000000000

Frequency (cm^{-1})	Intensity (km/mol)
43.8249	1.6286
61.5828	2.9239
103.1842	1.228
136.0398	0.0437
155.7203	12.6201
175.5761	0.3332
283.9613	10.9081
353.385	5.6252
580.8172	14.7306
582.9652	2.4572
629.6363	12.1909
665.2205	37.0046
685.4733	17.6241
702.5616	86.9375
748.2067	120.3117
788.6175	4.8556
897.367	0.0023
1016.1334	84.8896
1532.8454	21.1032
1909.3504	58.9274
3176.6212	7.6473
3206.9633	20.71
3299.1238	161.2372
3382.5844	97.5899

Table S13. Cartesian coordinates for the optimized geometry of isomer 2cii-doublet $Ti^+(C_2H_2)_2Ar$ followed by its predicted frequencies (cm^{-1}) and IR intensities (km/mol).

Z	x	y	z
22	1.633767000	-0.401769000	-0.569587000
6	-0.191623000	-0.666222000	0.098515000
6	0.620795000	-1.505653000	0.696844000
1	-1.244723000	-0.413079000	0.112692000
1	0.564469000	-2.284473000	1.447294000
6	2.288321000	1.683907000	0.126352000
6	3.045242000	0.903830000	0.682094000
1	1.737838000	2.530680000	-0.236371000
1	3.793766000	0.411952000	1.273185000
18	-4.187257000	0.338815000	0.017294000

Frequency (cm^{-1})	Intensity (km/mol)
9.4175	1.5261
13.3249	3.2072
26.0625	2.351
122.7532	3.0643
132.0731	0.1706
171.5082	0.2141
272.0677	8.1976
347.2685	4.9094
582.3136	3.0142
583.2147	13.5505
629.4208	8.3611
668.4899	38.5512
694.2183	28.961
709.6623	64.3854
753.1732	119.4728
791.9161	9.3281
896.3074	0.0636
1013.3575	92.0392
1532.3599	19.0246
1917.5908	46.6556
3176.7362	20.3123
3207.2704	36.8778
3299.7804	174.5462
3384.9004	93.123

Table S14. Cartesian coordinates for the optimized geometry of isomer 2ai-quartet $Ti^{+}(C_2H_2)_2Ar$ followed by its predicted frequencies (cm^{-1}) and IR intensities (km/mol).

Z	x	y	z
22	0.054164000	-0.001000000	-0.366558000
6	-1.178421000	1.390158000	0.490170000
6	-2.275616000	0.708736000	-0.042987000
6	-2.276837000	-0.706614000	-0.042691000
6	-1.180785000	-1.389718000	0.490674000
1	-1.237129000	2.405015000	0.869745000
1	-3.114390000	1.237087000	-0.494677000
1	-3.116565000	-1.233693000	-0.494091000
1	-1.241269000	-2.404384000	0.870486000
18	2.721539000	0.000145000	0.107879000

Frequency (cm^{-1})	Intensity (km/mol)
11.1952	1.1613
53.176	1.3685
121.7982	8.6188
222.6883	9.3548
271.3008	4.8506
313.6827	27.999
439.8816	0.1887
536.9128	18.5775
642.6161	21.3899
663.0779	73.7335
723.4886	23.4278
901.7035	36.7129
909.7106	59.0573
966.964	20.2755
1058.2987	3.3397
1075.8694	14.1958
1149.6699	55.2338
1172.0943	11.4917
1430.3327	14.4779
1436.3353	12.2342
3106.0246	0.3825
3118.4537	0.0008
3169.4749	11.5152
3169.6727	3.0732

Table S15. Cartesian coordinates for the optimized geometry of isomer 2aii-quartet $Ti^{+}(C_2H_2)_2Ar$ followed by its predicted frequencies (cm^{-1}) and IR intensities (km/mol).

Z	x	y	z
22	-2.245628000	-0.559548000	-0.409807000
6	-0.751510000	-0.983948000	0.928429000
6	-0.023741000	0.087633000	0.412352000
6	-0.700939000	1.287855000	0.072164000
6	-2.080634000	1.370895000	0.256779000
1	-0.312903000	-1.763199000	1.543178000
1	1.048848000	0.019028000	0.233454000
1	-0.128963000	2.110710000	-0.355353000
1	-2.609530000	2.310869000	0.376420000
18	4.041517000	-0.052331000	-0.155572000

Frequency (cm^{-1})	Intensity (km/mol)
7.5359	0.1557
11.5234	4.3451
27.8045	1.989
206.2588	3.3362
269.4524	3.759
299.348	34.8957
434.0221	0.6683
531.6604	15.6788
650.7479	25.5098
664.5927	84.6366
723.8588	27.5493
910.8704	40.1167
912.0633	69.0269
963.1279	18.6206
1059.967	3.6563
1075.546	18.9703
1155.9049	53.1716
1176.7816	12.1357
1428.9241	16.1764
1442.0227	11.8531
3106.0592	3.6929
3117.897	3.1596
3169.9946	10.746
3170.4738	8.1348

Table S16. Cartesian coordinates for the optimized geometry of isomer 2aiii-quartet $Ti^+(C_2H_2)_2Ar$ followed by its predicted frequencies (cm^{-1}) and IR intensities (km/mol).

Z	x	y	z
22	-1.613351000	-1.064475000	-0.176778000
6	0.012494000	0.167968000	0.026625000
6	-0.761497000	1.221669000	-0.457625000
6	-2.115222000	1.336886000	-0.046517000
6	-2.646181000	0.390989000	0.829789000
1	1.096056000	0.194990000	0.080345000
1	-0.363938000	1.944696000	-1.169215000
1	-2.720002000	2.148406000	-0.449869000
1	-3.505286000	0.584084000	1.464023000
18	4.113852000	-0.008822000	0.102789000

Frequency (cm^{-1})	Intensity (km/mol)
7.4815	2.1823
12.4777	1.1393
26.0239	3.105
205.0779	3.8644
269.5652	4.1021
298.6667	36.9618
434.056	0.772
532.031	15.1505
653.0473	29.8819
666.6621	80.512
724.774	25.3762
910.0231	57.9483
911.5824	54.6084
963.1644	20.1814
1059.8744	5.2001
1075.1316	15.824
1154.9318	57.8482
1176.0768	12.1816
1428.6944	14.6585
1441.5753	13.1811
3105.3393	0.4798
3117.5179	0.211
3169.8794	30.5846
3170.0249	5.1502

Table S17. Cartesian coordinates for the optimized geometry of isomer 2bi-quartet $Ti^{+}(C_2H_2)_2Ar$ followed by its predicted frequencies (cm^{-1}) and IR intensities (km/mol).

Z	x	y	z
6	-1.696668000	-0.743263000	0.870829000
6	-1.763351000	-0.885599000	-0.575780000
6	-2.141868000	0.518600000	-0.610618000
6	-1.766565000	0.707447000	0.782044000
1	-1.966077000	1.483432000	1.503154000
1	-1.723770000	-1.444405000	1.693352000
1	-1.959990000	-1.720978000	-1.228038000
1	-2.634435000	1.142345000	-1.343544000
22	0.104025000	0.185466000	-0.214860000
18	2.789246000	-0.062431000	0.072397000

Frequency (cm^{-1})	Intensity (km/mol)
20.5458	4.638
31.5792	4.0166
125.8216	6.3462
296.3211	9.1631
359.7578	0.9581
418.5881	7.9314
576.8045	0.0327
581.0931	22.3485
674.2124	89.109
753.7652	9.8094
762.4851	24.9365
850.8727	0.7154
902.4112	3.7397
946.8391	35.3809
953.5106	12.4746
1056.3164	0.0005
1188.9394	0
1230.7126	3.0807
1283.1554	27.4735
1344.2968	0.8952
3213.0714	0.8438
3218.486	11.4338
3245.4867	23.4685
3251.9472	5.8261

Table S18. Cartesian coordinates for the optimized geometry of isomer 2bii-quartet $Ti^{+}(C_2H_2)_2Ar$ followed by its predicted frequencies (cm^{-1}) and IR intensities (km/mol).

Z	x	y	z
6	1.992868000	1.273228000	-0.000196000
6	1.120010000	0.762963000	1.047069000
6	0.131384000	0.548181000	0.000186000
6	1.119638000	0.762811000	-1.047078000
1	1.045270000	0.953416000	-2.105592000
1	2.870563000	1.904754000	-0.000397000
1	1.046017000	0.953724000	2.105582000
1	-0.942125000	0.417662000	0.000384000
22	1.812172000	-1.016822000	0.000003000
18	-3.892828000	-0.107920000	0.000004000

Frequency (cm^{-1})	Intensity (km/mol)
8.7584	5.9286
13.8704	0.0389
29.1871	1.3329
286.3366	8.5371
360.0316	1.2699
416.2674	4.8584
571.8368	0.0025
582.9397	19.2728
675.4053	95.3819
756.9044	13.9251
763.5472	23.9523
854.6676	1.4434
901.1305	3.0303
947.061	32.1887
952.8966	13.0644
1056.8343	0.0416
1188.8743	0.0373
1226.6449	1.7333
1282.1016	30.1294
1341.779	0.8
3211.8336	4.1049
3217.1785	24.7433
3244.1838	25.0593
3250.4913	5.5944

Table S19. Cartesian coordinates for the optimized geometry of isomer 2ci-quartet $Ti^+(C_2H_2)_2Ar$ followed by its predicted frequencies (cm^{-1}) and IR intensities (km/mol).

Z	x	y	z
22	-0.415690000	0.048958000	-0.141788000
6	-1.295941000	-2.025133000	-0.398789000
6	-1.768810000	-1.556827000	0.641347000
1	-1.106033000	-2.723883000	-1.193447000
1	-2.353475000	-1.473211000	1.539494000
6	-0.161491000	2.302373000	0.150328000
6	-1.343424000	2.091491000	-0.133275000
1	0.737717000	2.835903000	0.396769000
1	-2.382226000	2.274829000	-0.339650000
18	2.314845000	-0.381229000	0.064361000

Frequency (cm^{-1})	Intensity (km/mol)
38.1607	0.0829
49.4099	0.8819
65.6634	0.636
77.3028	0.3053
123.3482	10.5197
126.7472	0.1419
306.0638	0.4346
346.065	5.83
374.5132	11.1788
400.2491	5.5013
641.6919	35.4943
649.888	62.9052
658.7172	63.1736
669.6369	8.6337
673.156	1.6029
714.6604	40.8876
720.6374	122.6885
725.1412	49.6681
1842.4919	160.646
1855.0777	26.7354
3268.9576	110.842
3273.7263	104.2776
3341.5457	128.8809
3348.2864	50.0354

Table S20. Cartesian coordinates for the optimized geometry of isomer 2cii-quartet $Ti^{+}(C_2H_2)_2Ar$ followed by its predicted frequencies (cm^{-1}) and IR intensities (km/mol).

Z	x	y	z
22	1.384120000	-0.245488000	-0.004265000
6	-0.645146000	-1.481869000	-0.000302000
6	0.230588000	-2.323553000	0.004123000
1	-1.533401000	-0.881097000	-0.003627000
1	0.856997000	-3.192521000	0.008492000
6	2.529417000	1.816816000	0.001136000
6	3.404646000	0.972313000	0.005707000
1	1.915988000	2.695352000	-0.002096000
1	4.304421000	0.390483000	0.010351000
18	-3.839538000	0.693682000	0.000929000

Frequency (cm^{-1})	Intensity (km/mol)
9.8378	1.4323
17.8491	0.1914
31.7219	0.002
42.0164	4.1312
83.3136	0.4818
144.5754	0.0745
178.5788	76.1338
250.6428	0.3644
290.0322	0.2681
299.133	0.0981
626.95	0.0059
634.0386	1.7692
640.1677	15.7195
654.7738	40.9707
684.8776	31.6609
749.4843	74.4567
762.9058	84.3284
773.8041	0.2486
1937.9341	140.9232
1959.9337	4.5003
3312.702	259.1532
3318.6468	220.3237
3400.6832	272.0463
3409.3642	1.2611

Table S21. Cartesian coordinates for the optimized geometry of isomer 2aI-doublet $Ti^{+}(C_2H_2)_2Ar_2$ followed by its predicted frequencies (cm^{-1}) and IR intensities (km/mol).

Z	x	y	z
22	0.223618000	-0.000002000	0.000029000
6	1.445718000	-0.000255000	-1.558387000
6	2.534816000	-0.000115000	-0.750809000
6	2.534857000	0.000117000	0.750738000
6	1.445805000	0.000232000	1.558377000
1	1.538428000	-0.000421000	-2.637507000
1	3.541029000	-0.000168000	-1.164516000
1	3.541094000	0.000201000	1.164389000
1	1.538577000	0.000405000	2.637492000
18	-1.745724000	-1.853389000	0.000123000
18	-1.745714000	1.853397000	-0.000123000

Frequency (cm^{-1})	Intensity (km/mol)
42.5037	0.0002
50.087	0.2753
56.4936	0.8089
100.8448	2.5609
122.8718	4.5417
152.4454	26.5501
243.4183	0
271.7448	25.3377
475.5575	9.0692
630.9694	111.2972
682.9184	22.8133
751.9003	69.3422
842.4387	0
855.2948	7.8707
989.6055	1.255
1025.1456	0
1099.0626	33.4772
1118.2362	5.3329
1305.0799	34.7358
1344.7297	28.5164
1437.7617	11.5331
1570.4205	1.3733
3112.8221	0.2626
3126.5923	0.4125
3185.4375	2.0285
3185.8246	10.9833

Table S22. Cartesian coordinates for the optimized geometry of isomer 2aII-doublet $Ti^{+}(C_2H_2)_2Ar_2$ followed by its predicted frequencies (cm^{-1}) and IR intensities (km/mol).

Z	x	y	z
22	-1.382927000	-0.000005000	0.427884000
6	-0.187813000	1.558716000	0.228945000
6	0.894865000	0.750969000	0.120396000
6	0.894859000	-0.750995000	0.120387000
6	-0.187825000	-1.558735000	0.228929000
1	-0.106086000	2.638271000	0.204922000
1	1.896438000	1.161993000	0.015662000
1	1.896428000	-1.162026000	0.015645000
1	-0.106106000	-2.638290000	0.204895000
18	-3.877637000	0.000015000	-0.512930000
18	4.897593000	0.000010000	-0.267431000

Frequency (cm^{-1})	Intensity (km/mol)
7.8766	0.3344
11.7717	0.2652
24.788	2.3117
42.3662	1.6399
46.4878	0.9375
95.2888	10.1973
139.5907	13.8835
244.4487	0.0127
273.2367	21.4177
472.1632	10.6754
630.1092	114.3846
684.8295	18.5078
751.6501	74.6181
843.0852	0.0348
853.9558	8.0592
986.0072	0.531
1023.904	0.1316
1095.369	27.2055
1116.0714	6.4882
1301.9089	41.0192
1342.6088	40.1397
1437.523	7.2642
1571.7875	1.2031
3118.2407	0.2047
3130.4456	0.7941
3188.7863	2.6937
3189.0873	17.545

Table S23. Cartesian coordinates for the optimized geometry of isomer 2aIII-doublet $Ti^{+}(C_2H_2)_2Ar_2$ followed by its predicted frequencies (cm^{-1}) and IR intensities (km/mol).

Z	x	y	z
22	3.064119000	-0.000123000	0.000215000
6	1.846578000	-1.549993000	0.000490000
6	0.751693000	-0.748893000	-0.000083000
6	0.751893000	0.749267000	-0.000669000
6	1.846993000	1.550074000	-0.000746000
1	1.773023000	-2.630616000	0.000888000
1	-0.251563000	-1.169491000	-0.000153000
1	-0.251251000	1.170132000	-0.001043000
1	1.773729000	2.630717000	-0.001194000
18	-2.823228000	-2.753000000	-0.000130000
18	-2.823299000	2.752958000	0.000287000

Frequency (cm^{-1})	Intensity (km/mol)
4.9984	0.7876
5.1582	0.6043
10.733	0
18.8481	2.4222
24.3432	1.8569
28.0794	5.2514
105.2966	16.2904
250.287	0
252.5408	8.388
470.3092	10.2301
630.4379	120.8893
689.2757	12.1007
751.6605	88.6331
844.892	0
862.2972	5.7388
985.0776	0.2777
1031.5243	0
1097.2431	41.3412
1116.4119	6.5302
1303.8835	36.7492
1341.7747	27.0876
1439.1901	7.2527
1568.5742	2.0948
3119.0482	1.3641
3131.3951	5.743
3187.4115	2.6553
3187.5243	24.5943

Table S24. Cartesian coordinates for the optimized geometry of isomer 2bI-doublet $Ti^+(C_2H_2)_2Ar_2$ followed by its predicted frequencies (cm^{-1}) and IR intensities (km/mol).

Z	x	y	z
6	-0.725843000	1.900154000	-0.723330000
6	0.725497000	1.900280000	-0.723332000
6	0.725494000	1.900327000	0.723301000
6	-0.725845000	1.900194000	0.723296000
1	-1.486971000	1.988756000	1.484101000
1	-1.486966000	1.988679000	-1.484142000
1	1.486602000	1.988988000	-1.484139000
1	1.486609000	1.989026000	1.484101000
22	0.000015000	-0.010566000	0.000033000
18	2.322407000	-1.481184000	-0.000007000
18	-2.322153000	-1.481523000	-0.000007000

Frequency (cm^{-1})	Intensity (km/mol)
26.8166	0.4844
41.6947	4.1093
44.2799	0
50.2448	7.8405
109.692	4.647
144.6543	24.2266
269.4788	1.2102
312.857	3.1379
468.1087	0.0835
649.8368	36.998
654.2564	22.9514
664.1385	0
798.3523	0
807.5754	111.4506
934.4824	29.3025
939.6509	31.2047
951.6827	0
957.9555	0.1156
1184.4491	0
1201.979	0.4954
1254.6125	0.6059
1332.1517	1.6614
1335.034	0.6792
3209.0667	0
3225.5236	7.8208
3225.7517	8.9246
3243.413	5.2419

Table S25. Cartesian coordinates for the optimized geometry of isomer 2cI-doublet $Ti^+(C_2H_2)_2Ar_2$ followed by its predicted frequencies (cm^{-1}) and IR intensities (km/mol).

Z	x	y	z
22	-0.492651000	-0.272909000	0.007458000
6	-1.345246000	-1.292464000	1.460001000
6	-1.133535000	-0.062294000	1.858548000
1	-1.773201000	-2.201959000	1.862946000
1	-1.300366000	0.541734000	2.741239000
6	-2.071843000	-0.153368000	-1.614473000
6	-1.863884000	1.009963000	-1.296261000
1	-2.447192000	-1.059417000	-2.050455000
1	-1.873983000	2.077432000	-1.194771000
18	1.909867000	-1.519281000	-0.458799000
18	1.241250000	2.054569000	0.238247000

Frequency (cm^{-1})	Intensity (km/mol)
33.4242	0.5689
47.9242	0.6548
65.3784	2.2925
68.6776	1.1376
83.2183	10.0176
100.1701	2.8987
136.9763	0.8751
150.9585	9.1593
176.3631	0.3846
291.2785	10.4212
358.5005	3.9906
577.2076	15.8535
588.9297	4.9661
631.902	18.7408
665.3607	39.5502
681.1006	15.385
705.2195	76.1506
742.7785	118.5791
794.4199	5.6662
902.1811	0.0203
1019.9264	80.2673
1545.1367	20.332
1900.77	59.3811
3177.7793	5.9933
3209.0842	15.4959
3299.7246	142.9798
3382.1506	86.9576

Table S26. Cartesian coordinates for the optimized geometry of isomer 2cII-doublet $Ti^+(C_2H_2)_2Ar_2$ followed by its predicted frequencies (cm^{-1}) and IR intensities (km/mol).

Z	x	y	z
22	-1.154660000	0.294017000	0.111943000
6	-0.036656000	0.376843000	1.725592000
6	0.725625000	0.193553000	0.673139000
1	0.086695000	0.491273000	2.795405000
1	1.780537000	0.083484000	0.453745000
6	-2.073998000	2.369641000	-0.154555000
6	-1.362866000	2.198850000	-1.133868000
1	-2.732854000	2.745869000	0.604274000
1	-0.806956000	2.283219000	-2.047821000
18	-2.316256000	-2.099684000	-0.313638000
18	4.736393000	-0.283957000	-0.293595000

Frequency (cm^{-1})	Intensity (km/mol)
6.7176	0.0928
8.6751	1.3135
23.789	1.3062
44.3875	1.8371
62.2751	3.038
103.2711	1.1364
136.4284	0.0455
155.8278	12.8464
176.9339	0.3695
284.1991	11.5684
354.1657	5.4436
581.3833	12.0554
582.3871	2.8608
629.7503	12.2821
665.1513	36.4638
685.9165	11.7074
703.9524	86.4745
747.7742	120.6632
789.9733	6.0019
899.0955	0.0564
1015.9674	86.9505
1533.0888	19.8373
1908.3617	60.6681
3176.0705	15.267
3206.4476	30.7434
3298.9347	159.7734
3382.2222	98.0642

Table S27. Cartesian coordinates for the optimized geometry of isomer 2cIII-doublet $Ti^+(C_2H_2)_2Ar_2$ followed by its predicted frequencies (cm^{-1}) and IR intensities (km/mol).

Z	x	y	z
22	-0.164968000	0.717726000	-0.957477000
6	0.419085000	-1.144940000	-0.763155000
6	1.422120000	-0.340219000	-0.499046000
1	0.228383000	-2.210663000	-0.790251000
1	2.461268000	-0.417551000	-0.203253000
6	-1.712519000	1.103285000	0.694656000
6	-0.776982000	1.845788000	0.945876000
1	-2.620254000	0.529849000	0.661860000
1	-0.080811000	2.551438000	1.356617000
18	-4.988025000	-0.891281000	0.423812000
18	5.406386000	-0.499081000	0.563385000

Frequency (cm^{-1})	Intensity (km/mol)
5.1988	2.7738
6.8504	0.5661
11.6589	0.2475
22.6987	6.7367
27.0398	1.1426
45.7308	3.6708
122.0281	3.184
137.5264	0.361
171.4137	0.3342
272.6541	7.4751
347.9814	6.099
583.2497	2.4076
583.9065	16.4437
636.356	8.1844
674.8634	40.4144
697.207	50.3014
715.4606	52.114
762.6463	99.544
792.6114	7.4903
896.841	0.055
1014.2931	91.9686
1531.557	18.7181
1918.9584	40.0126
3176.6393	19.4678
3207.0861	35.9418
3290.5435	267.7568
3379.1376	107.5593

Table S28. Cartesian coordinates for the optimized geometry of isomer 2aI-quartet $Ti^+(C_2H_2)_2Ar_2$ followed by its predicted frequencies (cm^{-1}) and IR intensities (km/mol).

Z	x	y	z
22	0.201103000	-0.117595000	0.019766000
6	1.636673000	-1.269190000	-0.906678000
6	2.520777000	-0.197036000	-0.761806000
6	2.525182000	0.537637000	0.448372000
6	1.616369000	0.202129000	1.456417000
1	1.808599000	-2.103744000	-1.578082000
1	3.177199000	0.116900000	-1.572974000
1	3.212629000	1.376431000	0.549764000
1	1.779364000	0.468249000	2.495970000
18	-2.029362000	-1.685018000	0.182966000
18	-1.537085000	2.078796000	-0.280041000

Frequency (cm^{-1})	Intensity (km/mol)
22.4366	0.8486
41.3095	1.3343
42.5201	1.7398
59.9289	0.4733
108.5804	6.9576
122.5811	8.0858
232.2548	6.534
273.8585	4.9713
316.3654	29.4728
436.9736	0.8715
536.9085	23.1485
642.28	23.3364
662.8985	66.009
724.7559	22.9289
898.6637	56.3687
909.2263	44.7731
967.8124	20.0316
1055.7417	4.994
1078.2025	12.3851
1144.1972	50.9432
1178.9341	14.0262
1430.8781	15.8465
1435.8552	10.6207
3103.1501	0.2349
3116.5514	0.2419
3165.6333	3.8445
3171.9524	6.7801

Table S29. Cartesian coordinates for the optimized geometry of isomer 2aII-quartet $Ti^{+}(C_2H_2)_2Ar_2$ followed by its predicted frequencies (cm^{-1}) and IR intensities (km/mol).

Z	x	y	z
22	1.300804000	0.045674000	-0.300868000
6	0.787721000	1.966341000	0.182101000
6	-0.554062000	1.628620000	-0.013076000
6	-1.021683000	0.367765000	0.428459000
6	-0.130874000	-0.509321000	1.052582000
1	1.140418000	2.991598000	0.226129000
1	-1.243236000	2.294245000	-0.531404000
1	-2.057935000	0.091851000	0.236627000
1	-0.446489000	-1.290503000	1.736363000
18	3.854230000	-0.836167000	-0.082936000
18	-4.992955000	-0.597858000	-0.192009000

Frequency (cm^{-1})	Intensity (km/mol)
4.7295	1.8462
8.3911	2.2992
12.3133	0.6264
25.1531	1.9535
54.0125	1.2091
121.9204	9.0909
224.0587	8.8411
271.3745	4.7957
314.0474	28.4295
440.0373	0.2939
537.1791	17.9088
642.5415	21.5827
663.4435	70.6309
723.3208	24.9898
902.7882	36.152
910.5455	59.2134
967.2618	18.9897
1058.5112	3.364
1076.2242	16.1074
1150.2395	52.5472
1172.8643	11.3962
1430.4745	15.4344
1436.8495	11.4059
3106.6359	2.7197
3118.7927	1.7387
3169.2946	8.6432
3169.7815	6.1174

Table S30. Cartesian coordinates for the optimized geometry of isomer 2aIII-quartet $Ti^{+}(C_2H_2)_2Ar_2$ followed by its predicted frequencies (cm^{-1}) and IR intensities (km/mol).

Z	x	y	z
22	-0.340118000	2.062066000	-0.376518000
6	-1.210386000	0.271571000	0.109840000
6	0.028080000	-0.344395000	-0.065764000
6	1.202968000	0.311463000	0.386416000
6	1.095744000	1.560546000	0.996979000
1	-2.149409000	-0.271367000	0.144080000
1	0.123561000	-1.305606000	-0.569830000
1	2.168967000	-0.163554000	0.218179000
1	1.841271000	1.956406000	1.678906000
18	-4.887471000	-1.562596000	0.034833000
18	4.820792000	-1.569428000	-0.132209000

Frequency (cm^{-1})	Intensity (km/mol)
3.102	2.292
7.0031	1.6924
11.5921	2.8777
13.9693	2.1434
24.1185	1.6638
29.205	3.0283
207.1114	3.3958
269.7168	3.8299
299.0998	37.5876
434.2455	0.9444
532.3753	14.5827
652.8034	29.6892
667.1975	77.2605
724.5743	27.0687
910.7982	56.1872
913.1673	55.7613
963.4825	18.8297
1060.3409	5.1401
1075.4495	18.0804
1155.5758	55.4864
1176.6979	11.6667
1428.9247	15.5436
1441.9874	12.5097
3105.8011	3.0214
3117.7024	3.4364
3169.822	29.3247
3170.2216	5.7611

Table S31. Cartesian coordinates for the optimized geometry of isomer 2bI-quartet $Ti^+(C_2H_2)_2Ar_2$ followed by its predicted frequencies (cm^{-1}) and IR intensities (km/mol).

Z	x	y	z
6	-2.249772000	-0.798192000	0.599370000
6	-2.091616000	-0.629949000	-0.837032000
6	-2.249781000	0.798128000	-0.599357000
6	-2.091591000	0.629886000	0.837043000
1	-2.306711000	1.263862000	1.681809000
1	-2.524583000	-1.633392000	1.228329000
1	-2.306736000	-1.263926000	-1.681796000
1	-2.524603000	1.633329000	-1.228311000
22	-0.170766000	-0.000033000	-0.000010000
18	1.819965000	-1.906369000	0.012025000
18	1.819816000	1.906460000	-0.012022000

Frequency (cm^{-1})	Intensity (km/mol)
29.3924	0.1068
42.5455	0.4527
45.3239	1.6709
59.408	1.8282
117.0682	6.6933
124.4022	9.1358
297.8631	9.488
361.2081	1.5673
413.3764	10.3433
574.9364	10.5841
586.2289	14.4355
665.05	86.4331
745.0589	5.099
760.0851	31.0763
845.5074	1.7053
899.3975	2.9429
945.4494	34.4217
952.3025	13.9457
1055.4196	0.0004
1187.678	0.0002
1228.6116	1.6993
1281.7221	27.6886
1344.1347	0.7402
3212.8454	0.6774
3218.3468	6.4655
3245.0784	16.5724
3251.5202	4.9404

Table S32. Cartesian coordinates for the optimized geometry of isomer 2bII-quartet $Ti^+(C_2H_2)_2Ar_2$ followed by its predicted frequencies (cm^{-1}) and IR intensities (km/mol).

Z	x	y	z
6	-0.127414000	2.003157000	-0.802213000
6	0.898165000	2.312894000	0.182624000
6	0.069391000	1.598049000	1.142372000
6	-0.765498000	1.045718000	0.087064000
1	-1.725288000	0.554998000	0.082136000
1	-0.390014000	2.420123000	-1.764522000
1	1.614782000	3.112637000	0.276837000
1	0.012288000	1.591205000	2.221985000
22	1.216690000	0.205615000	-0.281163000
18	-4.332956000	-1.069522000	0.000298000
18	2.848133000	-1.928334000	0.094705000

Frequency (cm^{-1})	Intensity (km/mol)
4.6566	0.6332
11.7901	0.98
21.4151	5.8859
25.6386	0.1414
31.3439	4.0365
125.6151	6.2069
296.5904	10.12
360.0319	0.9582
418.9088	8.2457
574.8438	0.0458
581.1318	22.2456
676.2565	93.7181
753.8743	9.7312
763.5775	22.2565
851.5563	0.4291
903.4601	2.9216
946.7519	33.8994
953.4176	11.5479
1057.172	0.0742
1189.883	0.0669
1230.7294	3.0539
1284.1628	24.7783
1344.2844	1.1564
3213.0378	0.8841
3218.3933	11.2043
3245.446	34.6392
3251.9158	8.4063

Table S33. Cartesian coordinates for the optimized geometry of isomer 2bIII-quartet $Ti^{+}(C_2H_2)_2Ar_2$ followed by its predicted frequencies (cm^{-1}) and IR intensities (km/mol).

Z	x	y	z
6	-0.617393000	1.854237000	1.241911000
6	0.825161000	1.934762000	1.060020000
6	0.748787000	0.574920000	0.544227000
6	-0.701666000	0.639903000	0.444502000
1	-1.468928000	-0.105331000	0.308952000
1	-1.326209000	2.401671000	1.847772000
1	1.601990000	2.493765000	1.556414000
1	1.472660000	-0.218308000	0.416783000
22	0.011571000	2.158598000	-0.945829000
18	-3.640220000	-2.214236000	-0.074620000
18	3.525587000	-2.345980000	-0.095694000

Frequency (cm^{-1})	Intensity (km/mol)
3.7427	1.2016
8.916	5.9005
10.01	5.5377
18.4578	0.2208
26.1368	1.2484
28.9699	1.2344
286.7043	9.4498
360.3625	1.2856
416.68	5.153
569.8204	0.0087
582.9839	19.2074
678.4475	97.7724
757.0862	13.3597
764.866	21.4677
855.5947	0.9883
902.2519	2.2537
947.0006	30.9561
952.968	11.7509
1057.7858	0.1332
1189.9065	0.11
1226.6149	1.6884
1283.1845	27.1144
1341.7603	1.0385
3211.7788	3.9445
3217.0938	24.2303
3243.9723	37.8728
3250.3134	8.3302

Table S34. Cartesian coordinates for the optimized geometry of isomer 2cI-quartet $Ti^{+}(C_2H_2)_2Ar_2$ followed by its predicted frequencies (cm^{-1}) and IR intensities (km/mol).

Z	x	y	z
22	0.557834000	-0.084758000	0.000008000
6	2.018109000	-0.041834000	-1.685562000
6	1.020571000	-0.653585000	-2.096523000
1	3.013575000	0.363812000	-1.687628000
1	0.422969000	-1.208151000	-2.799063000
6	2.018045000	-0.041530000	1.685629000
6	1.020446000	-0.653102000	2.096706000
1	3.013542000	0.364040000	1.687622000
1	0.422769000	-1.207446000	2.799356000
18	-1.117851000	2.103726000	-0.000208000
18	-1.971496000	-1.443018000	0.000098000

Frequency (cm^{-1})	Intensity (km/mol)
12.959	0.0006
41.7677	0.2172
51.7176	0.4308
56.6114	0.2918
66.1791	0.6854
75.1821	0.1137
90.0643	6.481
126.9732	10.1074
153.2049	0.7212
357.207	1.6244
358.5013	0.0014
410.5044	4.8985
437.0884	9.975
626.4149	9.4959
658.4955	83.3424
671.4743	120.8613
680.4423	0.043
686.1366	25.8094
701.2086	14.8718
717.7329	139.5244
747.4106	7.1781
1813.0726	162.1049
1831.453	3.1097
3256.3343	30.5341
3256.8581	125.2146
3326.3222	101.5127
3328.6232	23.1572

Table S35. Cartesian coordinates for the optimized geometry of isomer 2cIII-quartet $Ti^+(C_2H_2)_2Ar_2$ followed by its predicted frequencies (cm^{-1}) and IR intensities (km/mol).

Z	x	y	z
22	0.000212000	1.839678000	0.000011000
6	-2.285818000	2.454135000	-0.000085000
6	-2.289229000	1.238663000	0.000012000
1	-2.482886000	3.507154000	-0.000174000
1	-2.483256000	0.183968000	0.000089000
6	2.286364000	2.453683000	-0.000005000
6	2.289533000	1.238211000	0.000097000
1	2.483643000	3.506663000	-0.000096000
1	2.483349000	0.183476000	0.000188000
18	3.030570000	-2.560375000	-0.000035000
18	-3.031159000	-2.559754000	0.000014000

Frequency (cm^{-1})	Intensity (km/mol)
5.9923	0.0949
10.735	0
11.875	1.9632
18.8109	0.2063
39.2164	7.4091
44.4154	0.7765
44.8075	0.1081
85.0644	0.2213
147.5929	0
180.4071	76.9638
250.999	0.001
294.4388	0.054
297.8476	0.4978
634.6226	3.5837
635.5846	0
646.8395	0.0633
654.4433	57.9804
690.0773	36.2898
759.8737	0
761.8071	143.0646
776.0887	0.1746
1939.0265	137.0321
1958.1481	2.1535
3311.0354	11.6549
3312.1294	548.9679
3397.6258	293.2149
3406.6542	0.9575

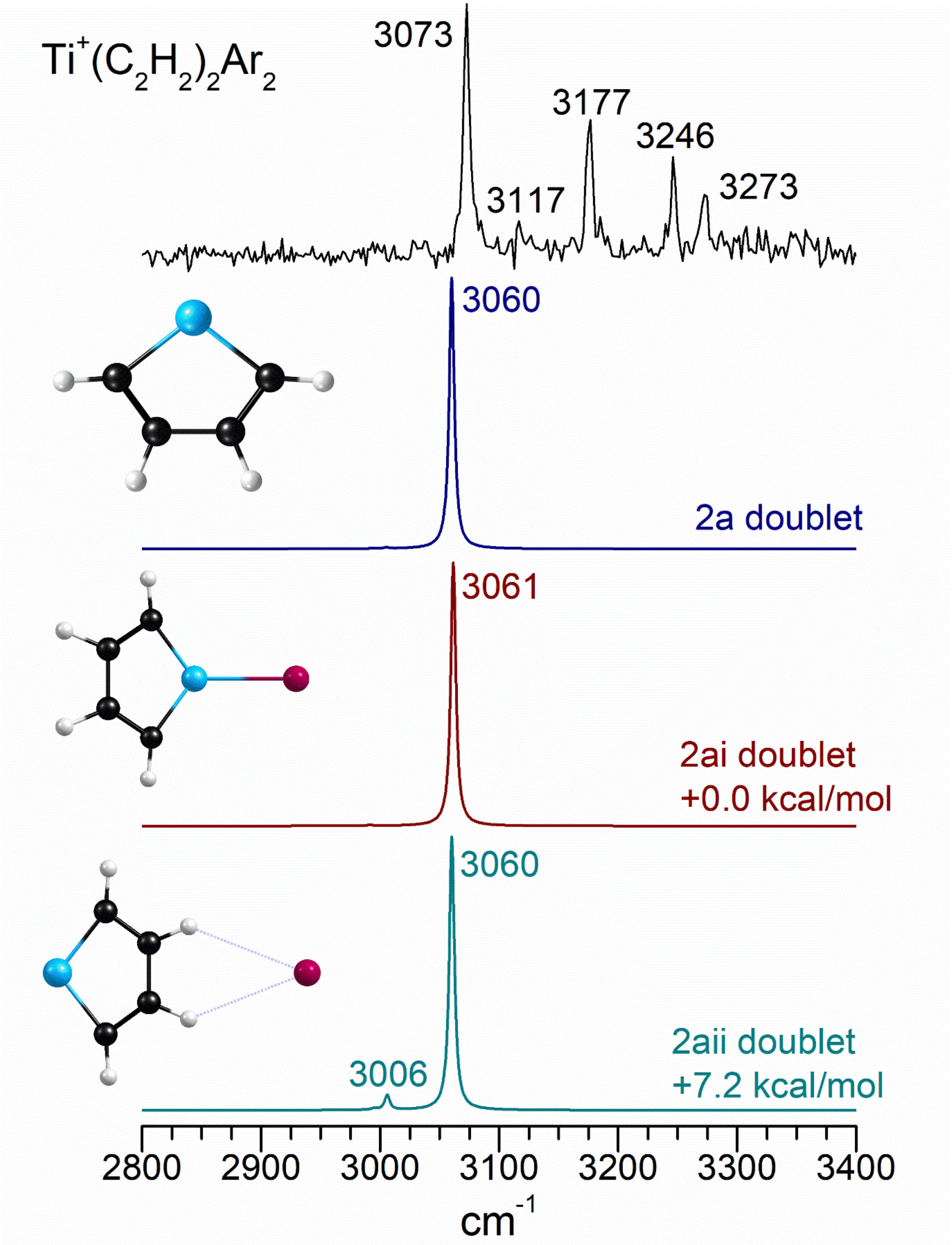

Figure S34. The experimental spectrum measured for $Ti^{+}(C_2H_2)_2Ar_2$ with simulated spectra for the 2a-doublet and singly-tagged doublet isomers.

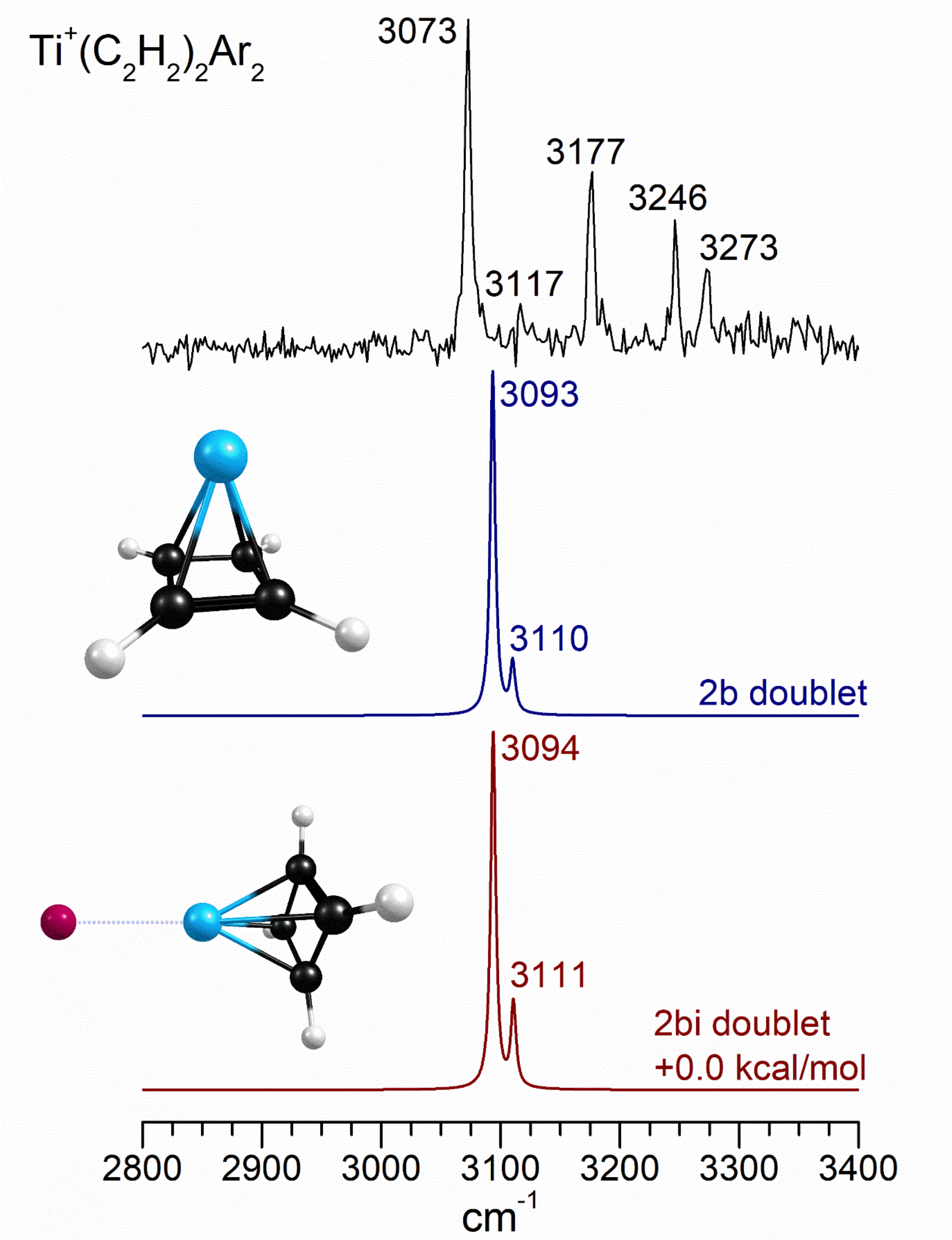

Figure S35. The experimental spectrum measured for $Ti^{+}(C_2H_2)_2Ar_2$ with simulated spectra for the 2b-doublet and singly-tagged doublet isomers.

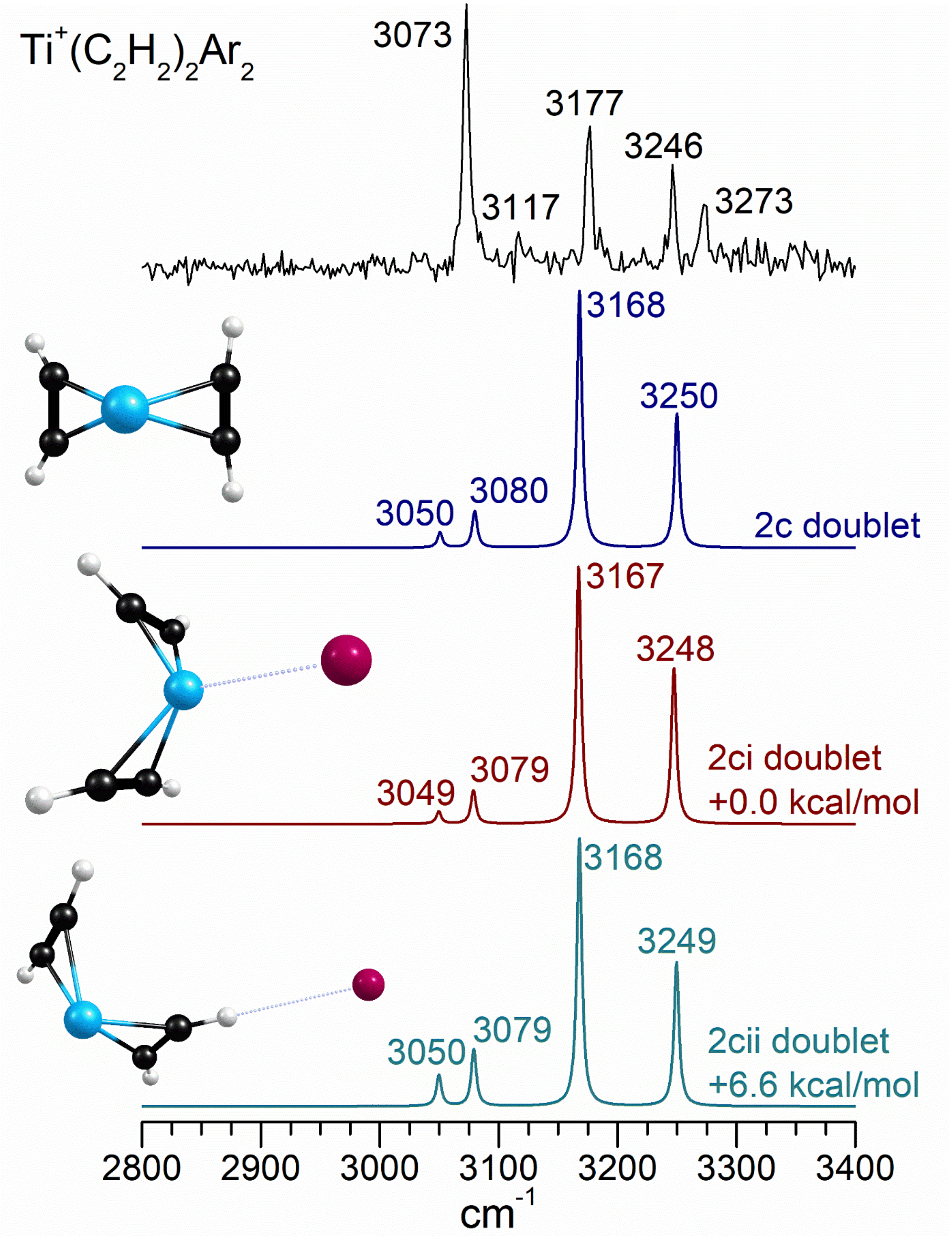

Figure S36. The experimental spectrum measured for $Ti^{+}(C_2H_2)_2Ar_2$ with simulated spectra for the 2c-doublet and singly-tagged doublet isomers.

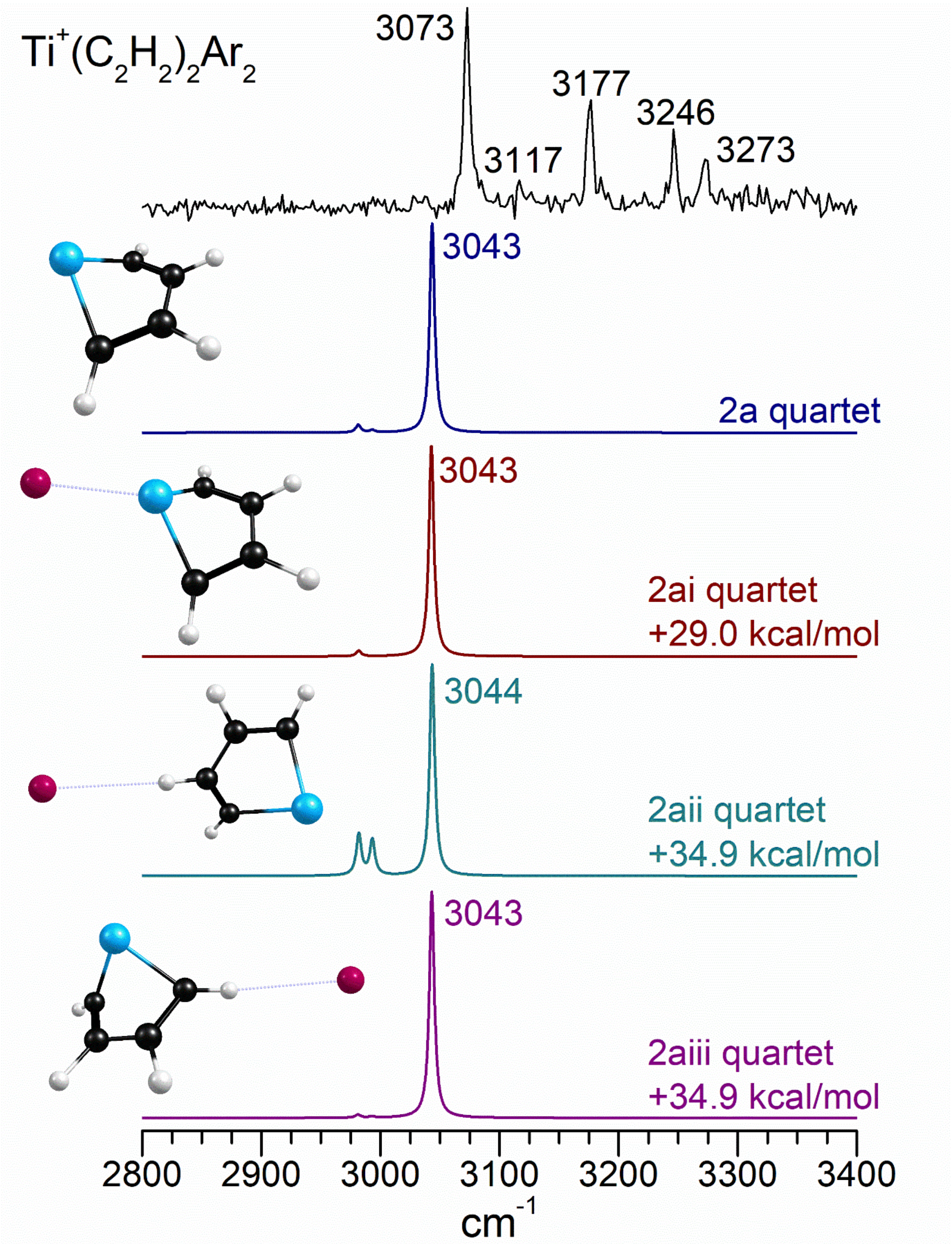

Figure S37. The experimental spectrum measured for $Ti^{+}(C_2H_2)_2Ar_2$ with simulated spectra for the 2a-quartet and singly-tagged quartet isomers.

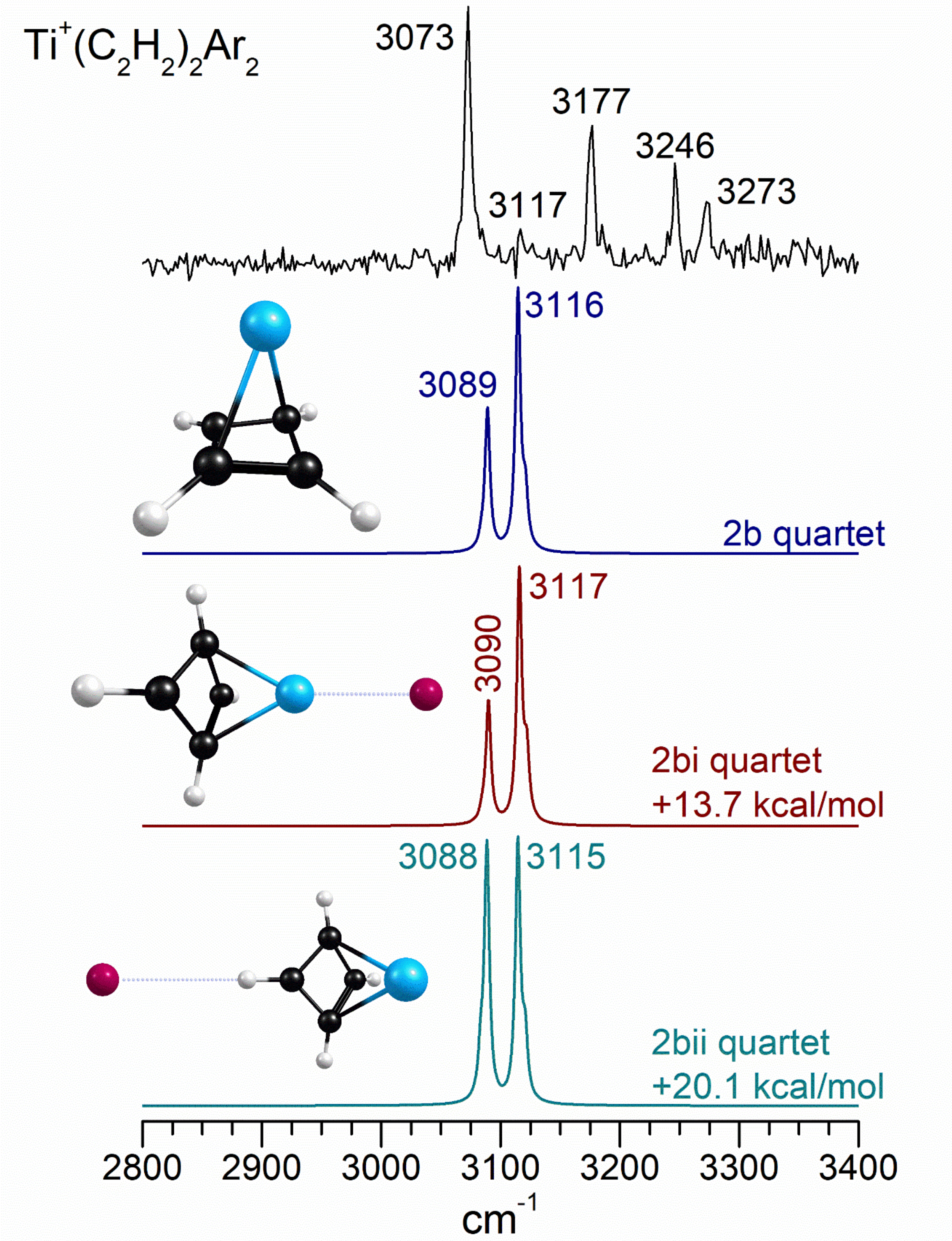

Figure S38. The experimental spectrum measured for $Ti^{+}(C_2H_2)_2Ar_2$ with simulated spectra for the 2b-quartet and singly-tagged quartet isomers.

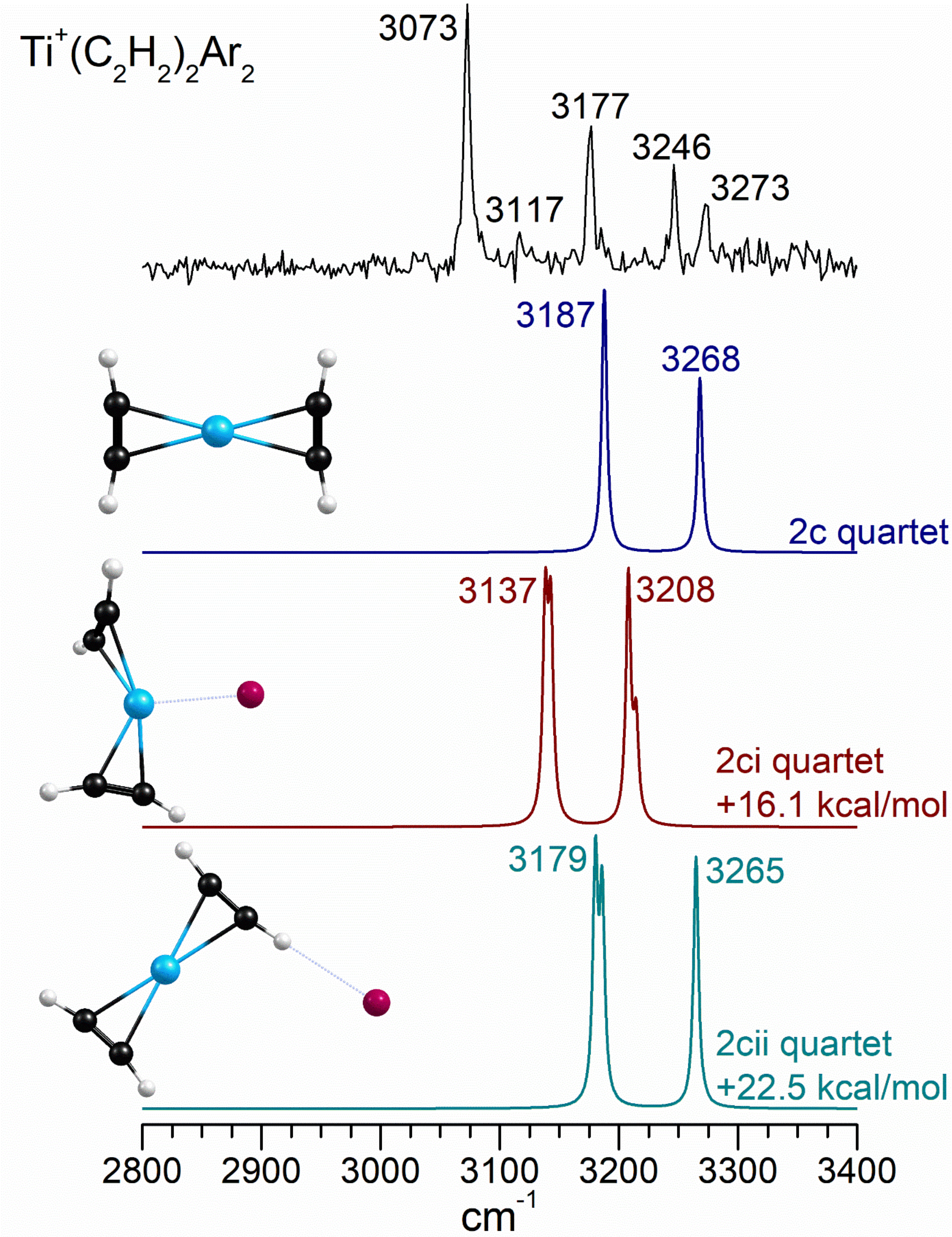

Figure S39. The experimental spectrum measured for $Ti^{+}(C_2H_2)_2Ar_2$ with simulated spectra for the 2c-quartet and singly-tagged quartet isomers.

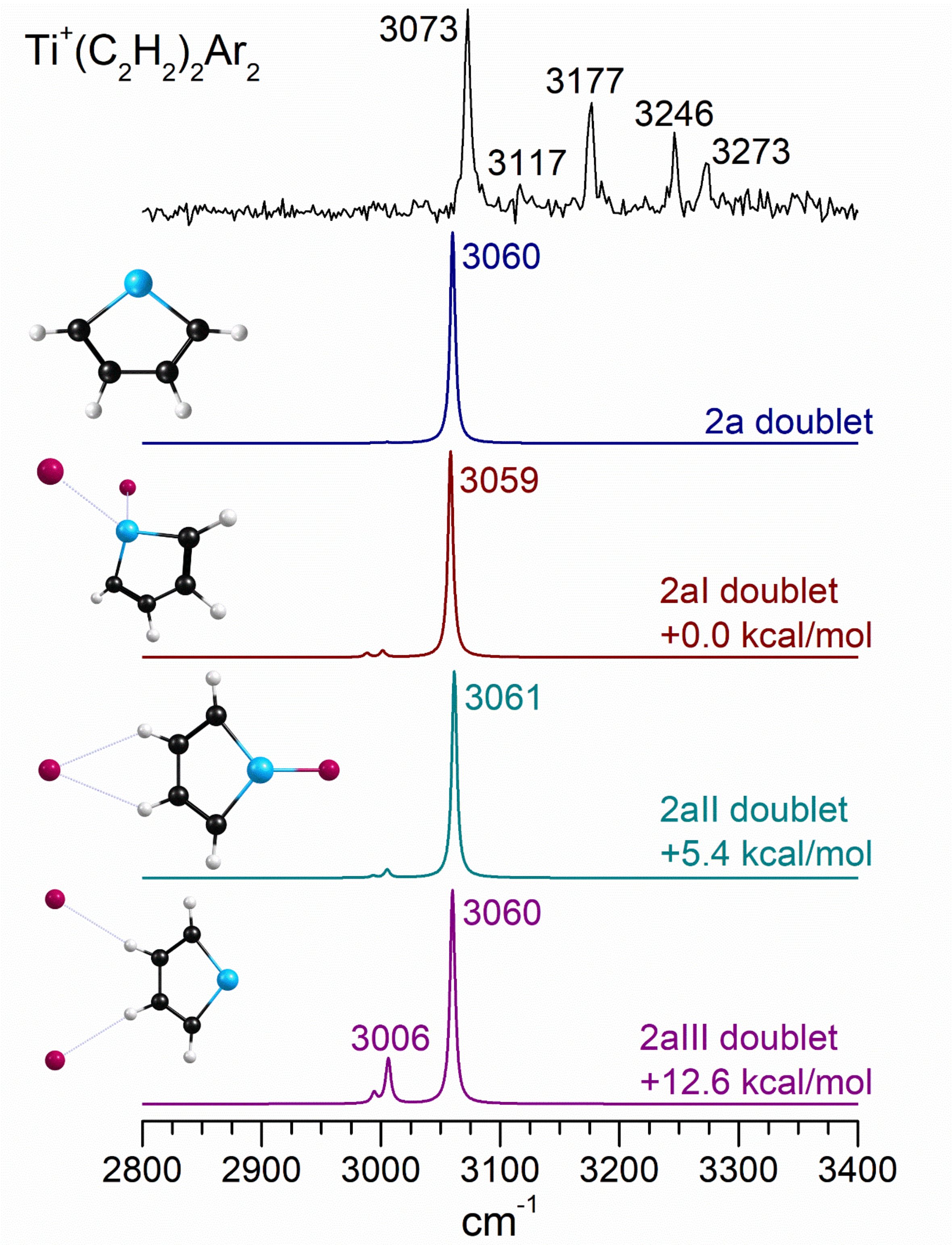

Figure S40. The experimental spectrum measured for $Ti^{+}(C_2H_2)_2Ar_2$ with simulated spectra for the 2a-doublet and doubly-tagged doublet isomers.

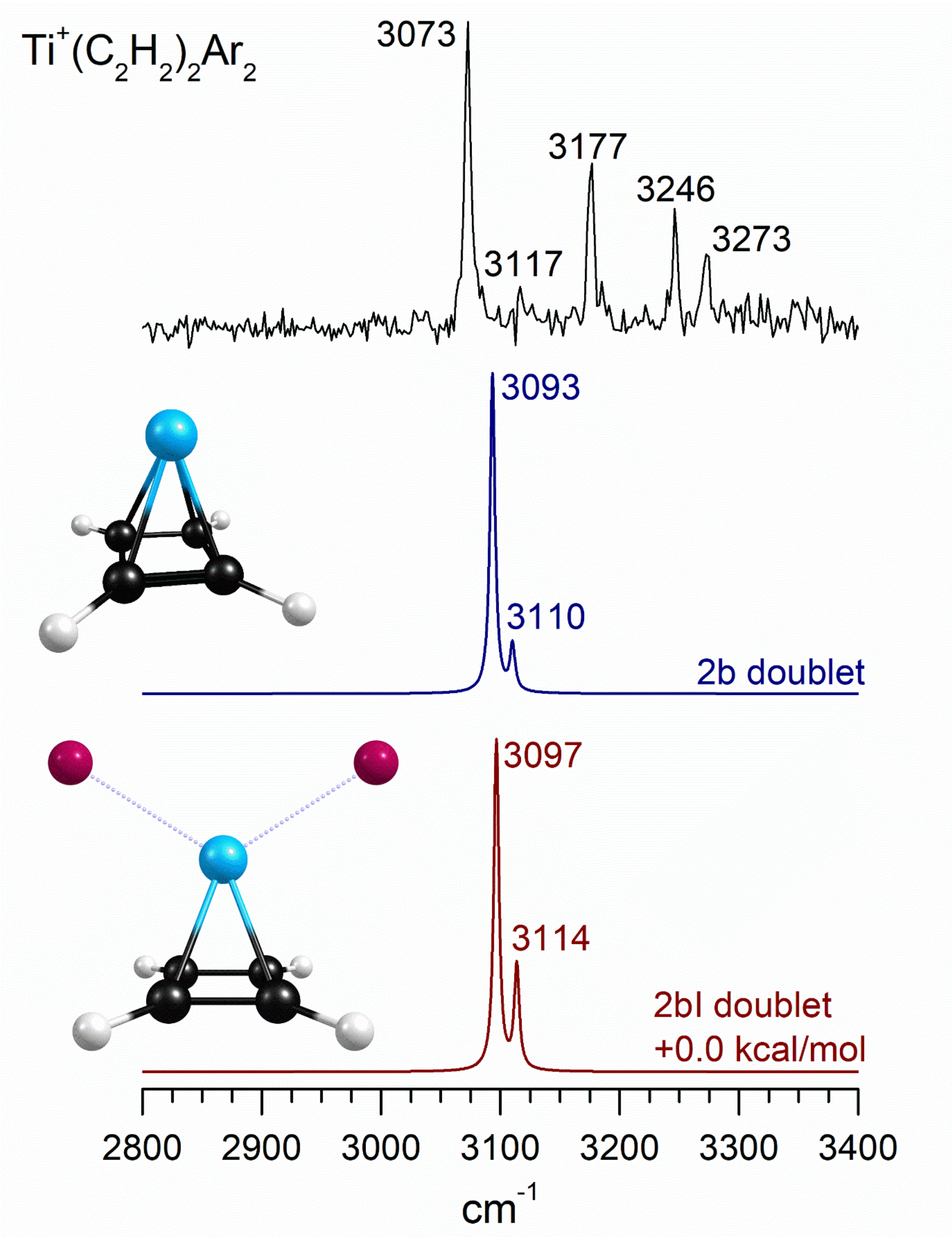

Figure S41. The experimental spectrum measured for $Ti^{+}(C_2H_2)_2Ar_2$ with simulated spectra for the 2b-doublet and doubly-tagged doublet isomers.

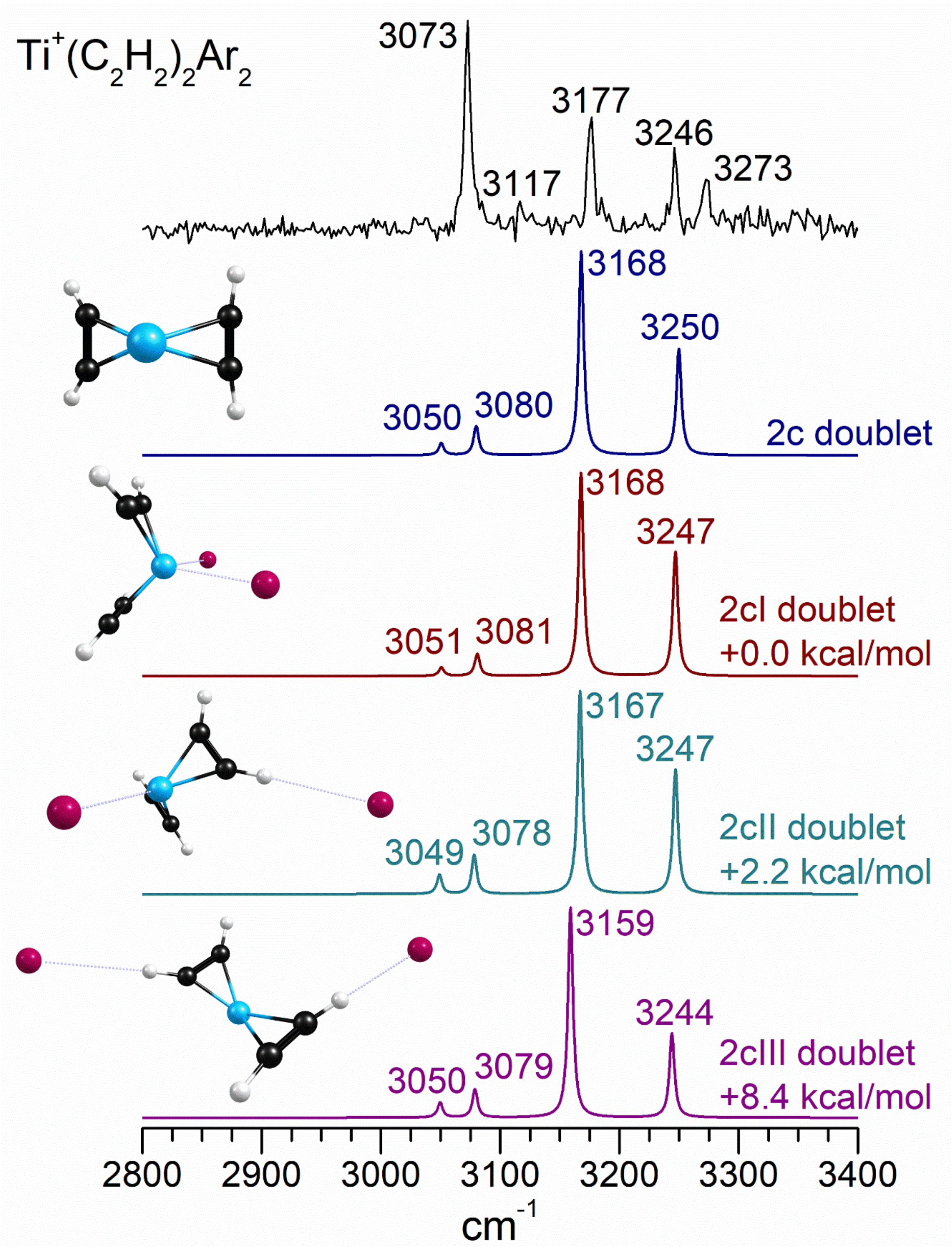

Figure S42. The experimental spectrum measured for $Ti^{+}(C_2H_2)_2Ar_2$ with simulated spectra for the 2c-doublet and doubly-tagged doublet isomers.

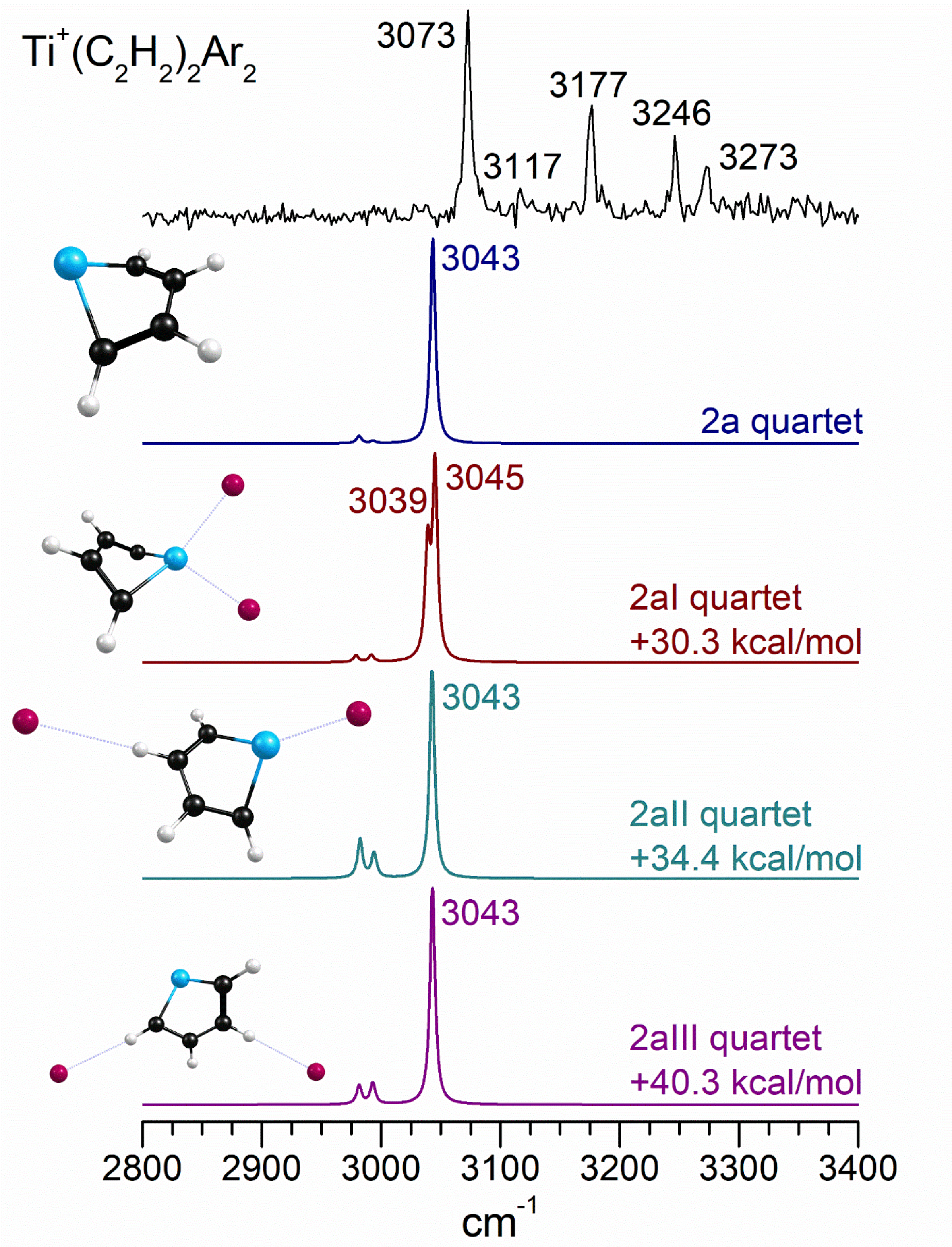

Figure S43. The experimental spectrum measured for $Ti^{+}(C_2H_2)_2Ar_2$ with simulated spectra for the 2a-quartet and doubly-tagged quartet isomers.

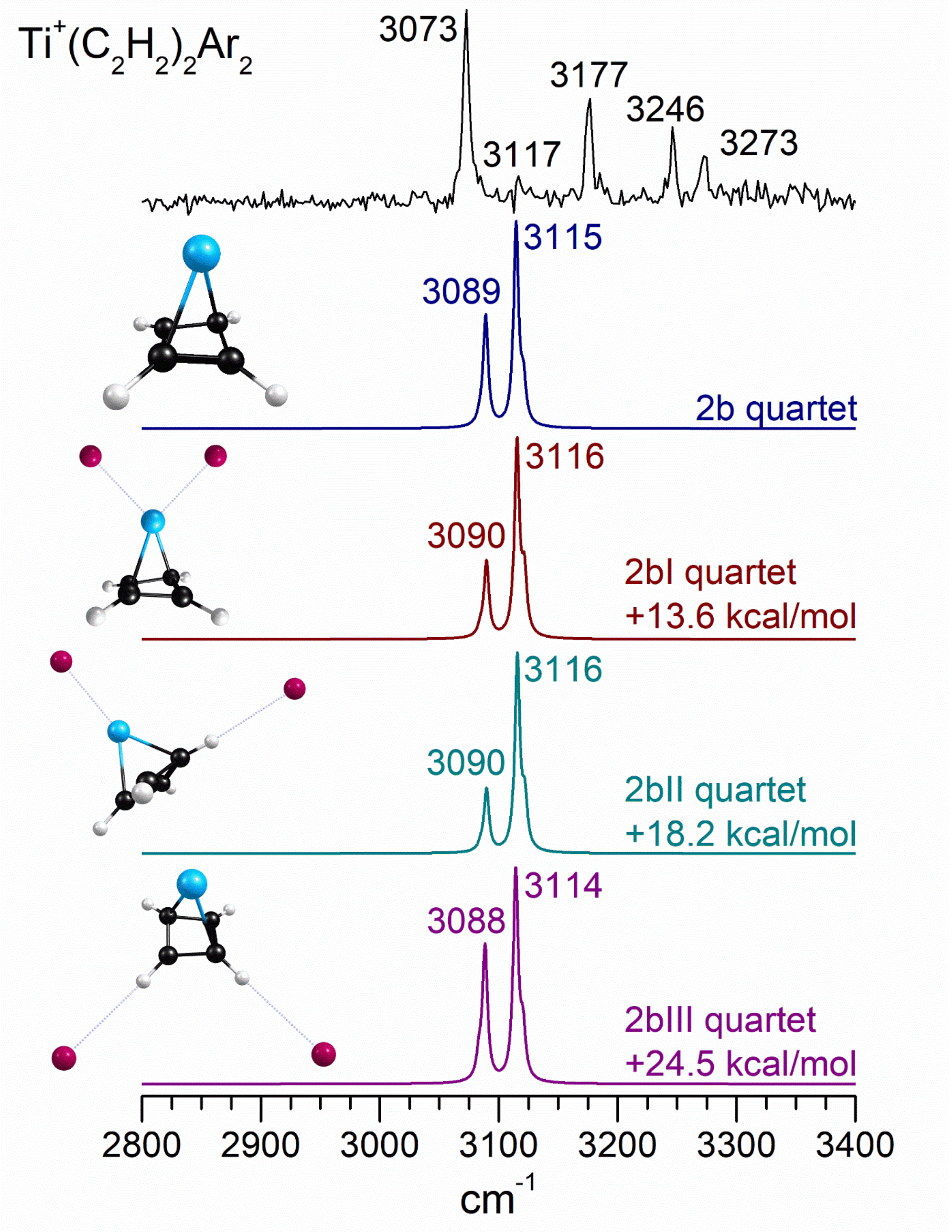

Figure S44. The experimental spectrum measured for $Ti^+(C_2H_2)_2Ar_2$ with simulated spectra for the 2b-quartet and doubly-tagged quartet isomers.

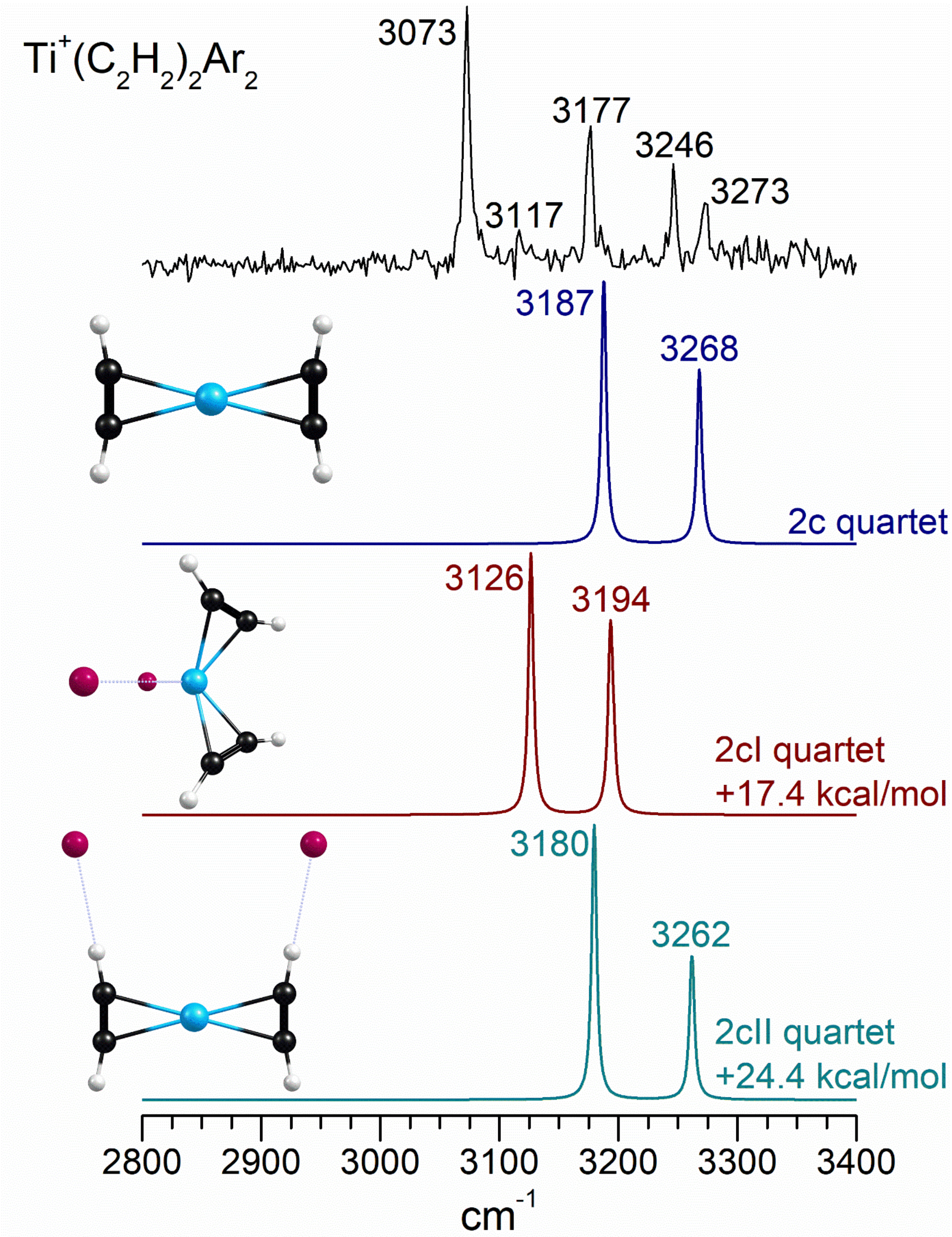

Figure S45. The experimental spectrum measured for $Ti^+(C_2H_2)_2Ar_2$ with simulated spectra for the 2c-quartet and doubly-tagged quartet isomers.

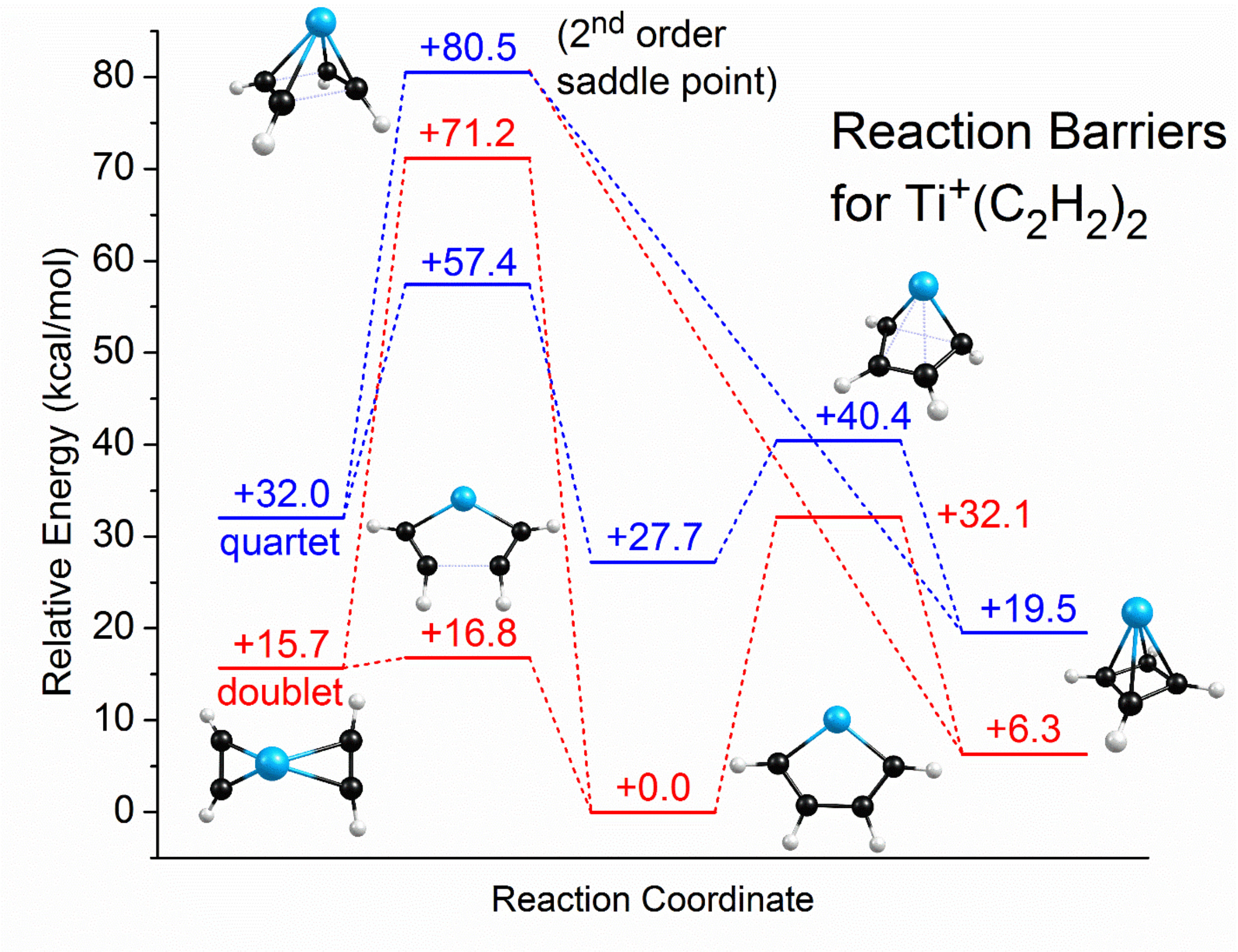

Figure S46. Reaction coordinate for the $Ti^{+}(C_2H_2)_2$ complex including SOSPs.

Table S36. $Ti^+(C_2H_2)_3$ electronic energy calculated at the B3LYP/def2-TZVP level.

Isomer	2s + 1	E (hartree)	Relative E (kcal/mol)
3a	2	-1081.438582	+9.3
3b	2	-1081.357344	+60.2
3c	2	-1081.342759	+69.4
3d	2	-1081.336558	+73.3
3e	2	-1081.318760	+84.5
3f	2	-1081.302218	+94.8
3a	4	-1081.453355	0.0
3b	4	-1081.318071	+84.9
3c	4	-1081.302305	+94.8
3d	4	-1081.288714	+103.3
3e	4	-1081.302158	+94.9
3f	4	-1081.280336	+108.6

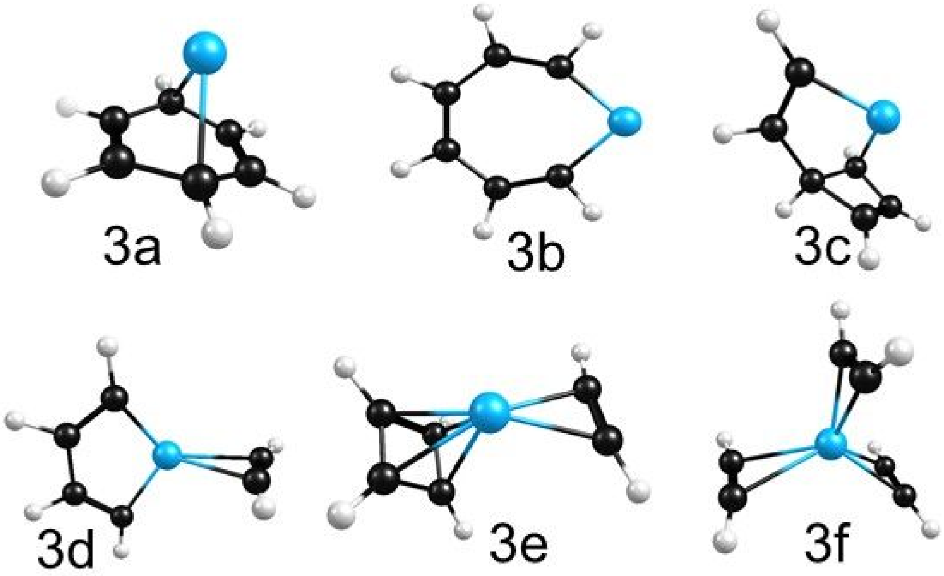

Figure S47. Isomers of $Ti^+(C_2H_2)_3$.

Table S37. Cartesian coordinates for the optimized geometry of isomer 3a-doublet $Ti^+(C_2H_2)_3$ followed by its predicted frequencies (cm^{-1}) and IR intensities (km/mol).

Z	x	y	z
22	0.000000000	0.000000000	1.176395000
6	1.228781000	-0.690004000	-0.679600000
6	0.000000000	-1.417255000	-0.457891000
6	1.228781000	0.690005000	-0.679600000
1	0.000000000	-2.497272000	-0.478769000
1	2.159805000	1.232891000	-0.779515000
6	-1.228781000	-0.690005000	-0.679600000
6	0.000000000	1.417255000	-0.457891000
1	-2.159805000	-1.232891000	-0.779515000
1	0.000000000	2.497272000	-0.478769000
6	-1.228781000	0.690004000	-0.679600000
1	-2.159805000	1.232890000	-0.779515000
1	2.159805000	-1.232890000	-0.779515000

Frequency (cm^{-1})	Intensity (km/mol)	Frequency (cm^{-1})	Intensity (km/mol)
282.9949	0	1035.5314	0.0149
301.017	0.0009	1088.7161	0
355.6891	2.0339	1168.2346	0.0123
359.5806	3.5148	1186.0225	0.3658
408.0657	0.2103	1310.7744	48.6513
598.3543	0.0397	1353.6572	0
614.4022	0	1410.2994	0
640.6109	9.3482	1442.4527	9.0093
802.095	94.9408	1499.6402	0.1675
832.7862	26.6378	1580.2786	15.5224
880.779	0.5337	3187.5375	0.4202
899.2084	6.7702	3188.5286	0
955.2274	0.5428	3197.9458	0.0963
962.0391	16.0959	3200.8928	2.9064
976.4739	0	3211.6051	6.7494
1007.3172	2.7485	3214.0173	0.7171
1018.5479	0.297		

Table S38. Cartesian coordinates for the optimized geometry of isomer 3b-doublet $Ti^+(C_2H_2)_3$ followed by its predicted frequencies (cm^{-1}) and IR intensities (km/mol).

Z	x	y	z
22	0.000000000	0.000000000	1.747000000
6	0.000000000	1.604205000	-0.973307000
6	0.000000000	0.688183000	-2.052541000
6	0.000000000	-0.688183000	-2.052541000
6	0.000000000	1.322284000	0.363707000
6	0.000000000	-1.604205000	-0.973307000
6	0.000000000	-1.322284000	0.363707000
1	0.000000000	1.149312000	-3.033474000
1	0.000000000	-2.651387000	-1.267424000
1	0.000000000	2.200223000	1.056745000
1	0.000000000	2.651387000	-1.267424000
1	0.000000000	-1.149312000	-3.033474000
1	0.000000000	-2.200223000	1.056745000

Frequency (cm^{-1})	Intensity (km/mol)	Frequency (cm^{-1})	Intensity (km/mol)
107.2335	6.6508	1045.7613	0
213.2385	0	1124.2454	7.6219
253.4026	1.3029	1200.9656	1.6721
281.027	14.9358	1302.1547	0.071
329.1083	19.2976	1346.2454	25.654
422.1701	8.6226	1365.7951	9.0978
441.6376	23.6944	1461.4617	6.2948
522.6945	0	1500.4304	7.53
565.567	135.1079	1560.7941	4.5368
633.9511	3.1173	1617.0544	0.5455
685.0145	0	2816.4257	29.8149
816.9999	0.6167	2817.0039	7.5312
836.4348	1.2366	3125.6227	0.8011
896.1276	8.5	3126.2628	0.0171
961.9788	0	3166.6613	0.1182
1005.925	0.2887	3181.9879	0.8583
1016.6022	0.0115		

Table S39. Cartesian coordinates for the optimized geometry of isomer 3c-doublet $Ti^{+}(C_2H_2)_3$ followed by its predicted frequencies (cm^{-1}) and IR intensities (km/mol).

Z	x	y	z
22	-0.306596000	-1.000484000	-0.000012000
6	1.069697000	0.337162000	1.035041000
6	0.242206000	1.165010000	0.000014000
1	0.578208000	2.197158000	0.000036000
1	1.178855000	0.438582000	2.104999000
6	-2.027281000	-0.002692000	-0.000003000
6	-1.320252000	1.134416000	-0.000011000
1	-1.775958000	2.121727000	-0.000040000
1	-3.109477000	-0.029252000	-0.000026000
6	1.932407000	-0.069590000	0.000016000
6	1.069720000	0.337192000	-1.035015000
1	1.178896000	0.438636000	-2.104969000
1	2.895592000	-0.565197000	0.000019000

Frequency (cm^{-1})	Intensity (km/mol)	Frequency (cm^{-1})	Intensity (km/mol)
88.3659	14.6172	985.9385	18.5322
180.1164	0.1111	1010.8295	0.1444
248.8868	17.5737	1075.6131	18.9788
368.558	0.2895	1160.6225	9.2456
380.689	5.7271	1196.6902	4.8416
428.446	22.8862	1232.5854	0.6584
590.5702	11.6217	1298.4396	33.475
654.7984	3.0887	1390.4386	5.0169
697.0051	43.4239	1391.0253	0.5624
719.0303	92.0719	1549.7585	10.7734
786.5574	15.4874	3122.4141	1.5775
847.1842	49.1615	3139.0317	10.7676
901.7322	0.0024	3192.7757	0.6328
916.79	13.2928	3193.4237	16.9165
943.112	75.0735	3220.6148	15.9738
956.068	28.37	3226.4695	4.9949
980.5078	0.0216		

Table S40. Cartesian coordinates for the optimized geometry of isomer 3d-doublet $Ti^{+}(C_2H_2)_3$ followed by its predicted frequencies (cm^{-1}) and IR intensities (km/mol).

Z	x	y	z
22	-0.315125000	0.000000000	-0.407172000
6	0.836971000	1.555905000	0.009903000
6	1.911693000	0.751694000	0.199643000
1	2.899607000	1.166654000	0.387761000
1	0.905057000	2.634040000	0.081751000
6	0.836973000	-1.555905000	0.009896000
6	1.911695000	-0.751693000	0.199639000
1	2.899609000	-1.166652000	0.387754000
1	0.905061000	-2.634040000	0.081739000
6	-2.155085000	0.000002000	0.951171000
6	-2.585995000	-0.000003000	-0.192872000
1	-3.224825000	-0.000003000	-1.056371000
1	-1.989280000	0.000002000	2.010869000

Frequency (cm^{-1})	Intensity (km/mol)	Frequency (cm^{-1})	Intensity (km/mol)
63.2645	0.276	852.1212	7.1164
109.3288	0.6382	993.672	2.9573
122.4465	13.8093	1024.4572	0.8732
130.4558	0.1983	1093.3939	21.4816
244.1896	18.1874	1114.1177	5.192
249.7603	0.6368	1300.2099	33.651
321.6503	1.6055	1341.5596	21.4239
350.8854	3.3794	1437.3553	16.5142
480.3194	6.1482	1568.1185	2.0587
636.806	53.2103	1909.4632	53.6557
647.1592	2.8517	3114.1872	0.1496
660.5874	82.2062	3127.6737	0.1249
679.2679	56.7729	3191.8436	2.4916
725.2273	1.3147	3192.2488	12.5503
735.9878	11.845	3294.903	172.8614
769.9014	115.8442	3381.5595	105.7613
844.8194	0.1849		

Table S41. Cartesian coordinates for the optimized geometry of isomer 3e-doublet $Ti^+(C_2H_2)_3$ followed by its predicted frequencies (cm^{-1}) and IR intensities (km/mol).

Z	x	y	z
22	-0.283183000	-0.000018000	-0.586411000
6	1.016257000	0.729170000	0.947550000
6	1.016257000	-0.729113000	0.947594000
6	1.821802000	-0.709560000	-0.268208000
6	1.821801000	0.709545000	-0.268251000
1	0.752447000	1.481427000	1.674434000
1	0.752447000	-1.481325000	1.674523000
1	2.251688000	-1.469574000	-0.905419000
1	2.251686000	1.469520000	-0.905509000
6	-2.386119000	-0.609814000	0.216312000
6	-2.386119000	0.609827000	0.216273000
1	-2.600754000	1.656797000	0.307648000
1	-2.600758000	-1.656778000	0.307742000

Frequency (cm^{-1})	Intensity (km/mol)	Frequency (cm^{-1})	Intensity (km/mol)
76.3724	4.3076	923.9943	17.1751
90.1708	1.6915	930.1476	7.7871
117.568	0.1994	956.7998	5.3647
227.5623	31.9664	971.0123	18.7298
245.742	13.056	1176.0286	5.5585
287.868	0.333	1186.4896	0.0288
324.3887	4.845	1250.8737	4.1507
459.3442	6.5568	1325.9761	5.438
618.0322	5.8547	1370.3045	3.9754
648.6928	9.1193	1920.023	99.041
656.4683	6.9668	3204.0712	0.0937
660.4475	3.0069	3222.1967	10.623
690.0538	34.2774	3231.5045	8.2118
693.5837	24.3488	3247.5499	7.1971
748.5367	97.8894	3304.7206	165.4171
810.6133	85.0598	3389.0803	140.1169
825.9365	1.8535		

Table S42. Cartesian coordinates for the optimized geometry of isomer 3f-doublet $Ti^{+}(C_2H_2)_3$ followed by its predicted frequencies (cm^{-1}) and IR intensities (km/mol).

Z	x	y	z
1	-0.000038000	2.616291000	-1.458489000
6	0.000006000	1.891364000	0.653943000
6	-0.000019000	1.891358000	-0.653960000
1	0.000021000	2.616304000	1.458466000
1	2.222775000	-1.065577000	1.664701000
6	2.069055000	-1.007899000	-0.606158000
6	2.069054000	-1.007949000	0.606171000
1	2.222743000	-1.065401000	-1.664700000
1	-2.222770000	-1.065602000	-1.664691000
6	-2.069049000	-1.007963000	-0.606162000
6	-2.069050000	-1.007902000	0.606167000
1	-2.222735000	-1.065400000	1.664709000
22	0.000001000	0.023788000	0.000000000

Frequency (cm^{-1})	Intensity (km/mol)	Frequency (cm^{-1})	Intensity (km/mol)
76.5201	0.0224	703.716	38.8061
86.4162	0	740.3074	10.3114
99.8505	9.8384	753.9721	44.9703
111.563	0.8036	765.8511	150.4202
132.7462	0.0787	801.1261	1.4289
178.2429	0	887.0875	0
224.7707	15.4009	1003.565	82.275
237.3952	2.0959	1548.5904	35.6488
288.5226	0	1959.3626	66.7972
328.724	13.0665	1976.2511	11.7591
559.2597	0.0668	3173.5564	4.4716
579.2431	9.8413	3204.8694	19.3058
612.8382	0	3324.1543	0
617.9239	1.0537	3325.5434	349.6202
654.0892	0	3411.6848	179.2972
673.2715	40.1776	3418.8854	31.966
681.002	53.728		

Table S43. Cartesian coordinates for the optimized geometry of isomer 3a-quartet $Ti^+(C_2H_2)_3$ followed by its predicted frequencies (cm^{-1}) and IR intensities (km/mol).

Z	x	y	z
22	0.000000000	-0.000001000	1.238613000
6	-0.705643000	-1.221325000	-0.647456000
6	-1.410536000	0.000441000	-0.647457000
6	0.704881000	-1.221765000	-0.647456000
1	-2.492089000	0.000778000	-0.656880000
1	1.245355000	-2.158594000	-0.656831000
6	-0.704880000	1.221766000	-0.647454000
6	1.410537000	-0.000439000	-0.647456000
1	-1.245355000	2.158595000	-0.656828000
1	2.492089000	-0.000777000	-0.656879000
6	0.705643000	1.221326000	-0.647454000
1	1.246702000	2.157817000	-0.656828000
1	-1.246702000	-2.157816000	-0.656832000

Frequency (cm^{-1})	Intensity (km/mol)
291.6935	0.126
291.7063	0.1262
310.2101	0.0813
418.9681	0
418.9755	0
617.2888	0
617.2903	0
652.7411	0
771.9921	96.4404
889.6584	4.3119
889.6592	4.3074
968.8134	0
968.8222	0
978.0151	0.7083
985.5608	0
1026.8004	7.1192
1026.8151	7.1215

Frequency (cm^{-1})	Intensity (km/mol)
1035.0821	0
1171.4987	0
1171.5193	0
1178.0754	0
1330.339	0
1373.4446	0
1481.0299	10.8378
1481.0364	10.8362
1532.3912	0
1532.3989	0
3191.9933	0
3197.4476	0
3197.4602	0
3207.5064	5.3689
3207.5189	5.3696
3213.6135	0.3476

Table S44. Cartesian coordinates for the optimized geometry of isomer 3b-quartet $Ti^{+}(C_2H_2)_3$ followed by its predicted frequencies (cm^{-1}) and IR intensities (km/mol).

Z	x	y	z
22	0.000000000	0.000000000	1.657903000
6	0.000000000	1.729895000	-0.964839000
6	0.000000000	0.714835000	-1.924876000
6	0.000000000	-0.714835000	-1.924876000
6	0.000000000	1.652613000	0.424602000
6	0.000000000	-1.729895000	-0.964839000
6	0.000000000	-1.652613000	0.424602000
1	0.000000000	1.102459000	-2.938518000
1	0.000000000	-2.724154000	-1.405705000
1	0.000000000	2.640301000	0.897960000
1	0.000000000	2.724154000	-1.405705000
1	0.000000000	-1.102459000	-2.938518000
1	0.000000000	-2.640301000	0.897960000

Frequency (cm^{-1})	Intensity (km/mol)	Frequency (cm^{-1})	Intensity (km/mol)
98.8643	0	1059.5341	3.0667
111.5344	6.1903	1160.1305	20.9539
187.6996	3.4473	1253.5718	10.3006
277.4699	4.111	1303.3554	1.7843
321.9528	3.7321	1327.6415	38.103
435.4596	0.241	1384.7865	16.3978
477.1866	0	1471.0526	64.8475
499.5288	24.8997	1472.448	84.5685
591.8654	143.6575	1530.8236	0.8924
596.5936	3.5907	1547.2085	11.3725
618.6555	0	3042.7012	0.4621
807.0886	0.2387	3043.6507	10.3676
825.69	1.019	3120.7625	2.5033
834.1882	0.5737	3121.9211	0.3074
942.6636	0	3151.2408	0.4125
1022.5708	1.2872	3168.2282	0.4002
1033.7186	0		

Table S45. Cartesian coordinates for the optimized geometry of isomer 3c-quartet $Ti^+(C_2H_2)_3$ followed by its predicted frequencies (cm^{-1}) and IR intensities (km/mol).

Z	x	y	z
22	-0.615251000	-1.160774000	-0.055352000
6	1.065876000	0.312719000	1.057670000
6	0.614079000	1.309007000	-0.022278000
1	1.185978000	2.239795000	-0.007615000
1	1.085969000	0.366702000	2.137153000
6	-1.745934000	0.558244000	0.154382000
6	-0.884295000	1.554219000	-0.107513000
1	-1.194784000	2.563424000	-0.373148000
1	-2.814794000	0.757457000	0.147442000
6	1.653927000	-0.543220000	0.096419000
6	1.222543000	0.266328000	-0.979306000
1	1.406444000	0.269804000	-2.045050000
1	2.309539000	-1.403939000	0.162725000

Frequency (cm^{-1})	Intensity (km/mol)	Frequency (cm^{-1})	Intensity (km/mol)
52.4492	11.2116	1004.4775	3.2143
179.8195	2.0208	1104.3027	7.8256
234.1363	25.6003	1135.4739	5.6285
266.8108	1.8835	1165.1624	11.8186
347.226	9.9724	1208.1459	0.2643
394.4872	8.6101	1302.2895	1.4238
544.0735	30.1388	1306.681	19.1189
635.4685	4.6302	1315.8196	15.0234
704.2799	26.5758	1393.7247	7.8432
716.2302	26.491	1560.9395	3.6467
751.9551	17.6184	3064.7855	0.1173
874.9149	15.6488	3110.5309	3.7107
895.2966	5.1117	3137.6912	0.6813
919.134	23.9671	3179.4043	1.3854
932.8763	3.6014	3217.2625	9.9789
956.6489	0.1013	3224.5741	6.8886
978.3481	22.1195		

Table S46. Cartesian coordinates for the optimized geometry of isomer 3d-quartet $Ti^{+}(C_2H_2)_3$ followed by its predicted frequencies (cm^{-1}) and IR intensities (km/mol).

Z	x	y	z
22	-0.339293000	0.000003000	-0.420229000
6	0.883714000	1.385471000	0.455312000
6	2.003977000	0.705945000	-0.041014000
1	2.854521000	1.237096000	-0.465794000
1	0.927840000	2.398580000	0.840836000
6	0.883707000	-1.385473000	0.455308000
6	2.003973000	-0.705952000	-0.041016000
1	2.854515000	-1.237106000	-0.465797000
1	0.927826000	-2.398582000	0.840832000
6	-2.270624000	-0.000004000	0.824597000
6	-2.623739000	0.000000000	-0.346384000
1	-3.225070000	0.000004000	-1.236138000
1	-2.161234000	0.000004000	1.890279000

Frequency (cm^{-1})	Intensity (km/mol)	Frequency (cm^{-1})	Intensity (km/mol)
88.9124	0.8363	892.0097	31.244
97.5626	0.4567	909.7793	51.2641
99.7479	0.0583	969.1132	9.7036
193.8866	3.1578	1056.6051	2.6279
261.8769	3.3445	1075.7229	4.0099
312.3469	5.0023	1142.8673	47.3286
334.0096	24.4517	1164.7656	12.1691
354.5898	7.6528	1427.876	23.4096
449.8477	0.5173	1428.7119	14.688
549.2948	4.5475	1905.0154	82.8135
650.6085	14.3057	3109.5886	0.5172
652.6938	10.5227	3122.1594	0.008
659.7442	7.2985	3172.2575	8.43
665.3694	96.1413	3172.4219	3.6504
696.8778	32.8244	3299.4037	153.3053
723.8019	0.2181	3388.9103	109.883
759.2578	83.3078		

Table S47. Cartesian coordinates for the optimized geometry of isomer 3e-quartet $Ti^{+}(C_2H_2)_3$ followed by its predicted frequencies (cm^{-1}) and IR intensities (km/mol).

Z	x	y	z
22	-0.381982000	0.490340000	0.066396000
6	1.517794000	-0.570430000	0.902208000
6	1.047633000	-1.111010000	-0.368808000
6	1.645327000	0.061856000	-0.991649000
6	1.827740000	0.707070000	0.296691000
1	1.687497000	-1.000128000	1.879438000
1	0.862317000	-2.113412000	-0.718052000
1	1.956202000	0.298440000	-1.999537000
1	2.385830000	1.570692000	0.622296000
6	-2.237506000	-0.852187000	0.055623000
6	-2.627903000	0.307108000	-0.091153000
1	-3.318670000	1.122025000	-0.218097000
1	-2.208086000	-1.919534000	0.155763000

Frequency (cm^{-1})	Intensity (km/mol)	Frequency (cm^{-1})	Intensity (km/mol)
49.2148	0.7149	857.7661	1.7248
88.4445	1.2884	902.3261	2.5615
102.895	0.4058	947.4358	30.1901
272.6356	6.3268	955.8247	16.2671
312.3692	13.0024	1059.1535	0.0532
360.0154	5.9873	1189.0526	0.0066
375.1563	7.0102	1236.004	3.2031
430.4868	2.2966	1287.0243	26.9194
560.0465	17.2072	1349.0124	1.3106
617.9248	4.095	1857.2205	142.0172
638.114	25.8071	3215.3681	0.3916
659.1291	70.0265	3221.7963	10.2342
665.8787	16.2243	3243.5594	21.5139
690.2896	63.4969	3254.4471	8.7486
730.4734	75.6741	3273.9113	115.6234
757.4539	12.5968	3359.3201	101.0002
767.5098	35.3932		

Table S48. Cartesian coordinates for the optimized geometry of isomer 3f-quartet $Ti^{+}(C_2H_2)_3$ followed by its predicted frequencies (cm^{-1}) and IR intensities (km/mol).

Z	x	y	z
1	2.528506000	1.304677000	-1.306167000
6	1.732294000	0.893906000	0.778411000
6	1.983412000	1.023439000	-0.422527000
1	1.819364000	0.938873000	1.847064000
1	-0.096669000	-2.045020000	1.847081000
6	-0.105332000	-2.229392000	-0.422510000
6	-0.092024000	-1.947145000	0.778427000
1	-0.134267000	-2.842076000	-1.306152000
1	-2.394211000	1.537256000	-1.306188000
6	-1.878072000	1.205889000	-0.422538000
6	-1.640289000	1.053275000	0.778403000
1	-1.722732000	1.106272000	1.847055000
22	0.000003000	0.000009000	-0.364940000

Frequency (cm^{-1})	Intensity (km/mol)	Frequency (cm^{-1})	Intensity (km/mol)
75.9316	0.7484	668.1134	18.2557
75.976	0.7486	668.1142	18.2152
118.0863	0.1157	674.1279	153.9219
118.1264	0.1167	709.0805	0.0002
122.0217	0	726.619	127.5346
139.6906	0.127	726.6525	127.5233
317.9817	0.3344	754.8321	0.1214
348.1314	6.5732	1840.3179	149.631
348.1354	6.5701	1840.3297	149.6552
375.0535	6.3687	1865.6418	1.447
375.0588	6.3698	3270.4095	31.4139
432.8485	21.0092	3270.4144	31.4243
633.1976	0.1012	3271.4444	245.2182
633.2092	0.0992	3348.701	108.138
647.5061	42.788	3348.7107	108.1406
647.5273	42.8234	3352.1977	18.6713
655.4217	0		

Figure S48. The optimized geometry of the TS1 doublet $Ti^{+}(C_2H_2)_3$ followed by its predicted frequencies (cm^{-1}) and IR intensities (km/mol).

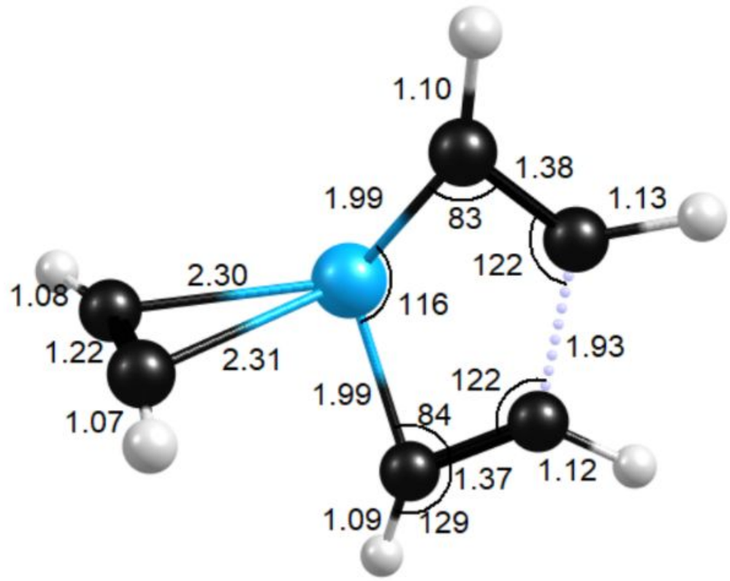

Frequency (cm^{-1})	Intensity (km/mol)	Frequency (cm^{-1})	Intensity (km/mol)
-254.07312	0.604	808.701792	2.6916
134.13168	1.1641	906.29088	32.1709
140.099712	0.2883	952.427712	4.7362
171.0912	0.5501	964.260768	4.4368
257.3064	10.1322	986.949216	5.1597
311.80752	2.0279	1149.810432	7.9302
338.771712	8.8704	1185.111264	17.7006
379.543584	0.7645	1204.374144	14.4695
445.492704	11.0516	1314.427488	5.2736
455.091552	0.036	1831.027392	47.2663
639.470592	7.7967	2653.74528	6.9888
644.322144	6.2776	2714.287968	8.6427
664.652256	36.1804	2948.774688	22.8659
699.568128	29.9004	2970.225504	25.9275
717.539328	13.5309	3129.247776	168.6376
745.95456	101.5519	3228.395232	84.5317
749.855616	105.9144		

Figure S49. The optimized geometry of the TS1 quartet $Ti^{+}(C_2H_2)_3$ followed by its predicted frequencies (cm^{-1}) and IR intensities (km/mol).

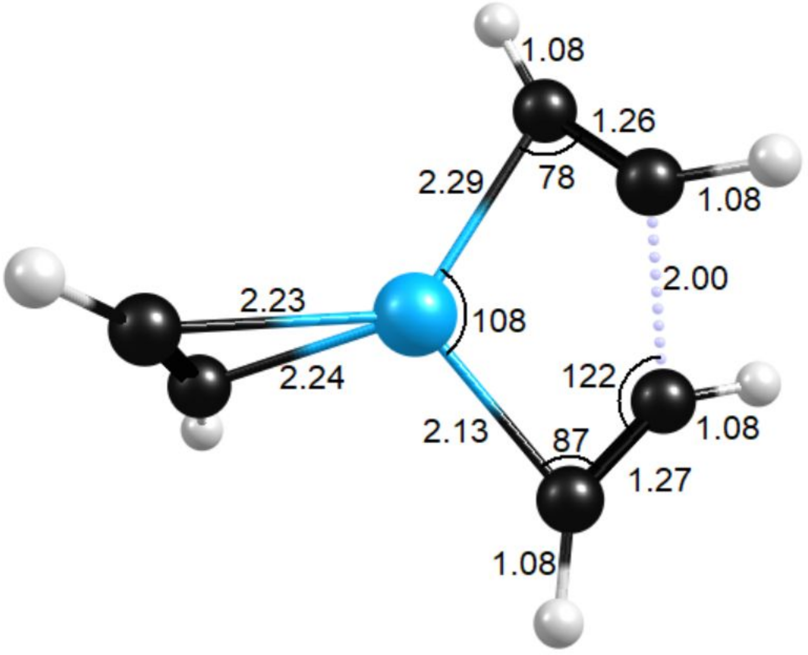

Frequency (cm^{-1})	Intensity (km/mol)	Frequency (cm^{-1})	Intensity (km/mol)
-466.448736	114.7228	664.9632	41.6781
60.260832	0.3418	683.284416	60.0522
64.387872	0.9391	692.134176	104.3168
98.441952	6.7335	720.089472	39.9515
121.167648	1.6379	750.708096	10.188
173.120448	2.2774	895.569312	40.5325
234.616416	21.9381	1020.469248	6.0783
295.607232	12.6762	1583.874048	57.7401
366.27696	2.2247	1696.089792	24.4353
389.214624	0.8665	1774.53312	96.7543
418.000224	4.402	3064.184064	37.8839
519.51552	13.3874	3084.591744	55.855
588.440352	71.4282	3134.792544	66.6436
620.726208	17.2389	3137.71488	113.0188
643.712256	32.4926	3169.161504	82.5151
644.744832	55.9046	3209.32992	94.9436
653.170848	51.2032		

Figure S50. The optimized geometry of the TS2 doublet $Ti^{+}(C_2H_2)_3$ followed by its predicted frequencies (cm^{-1}) and IR intensities (km/mol).

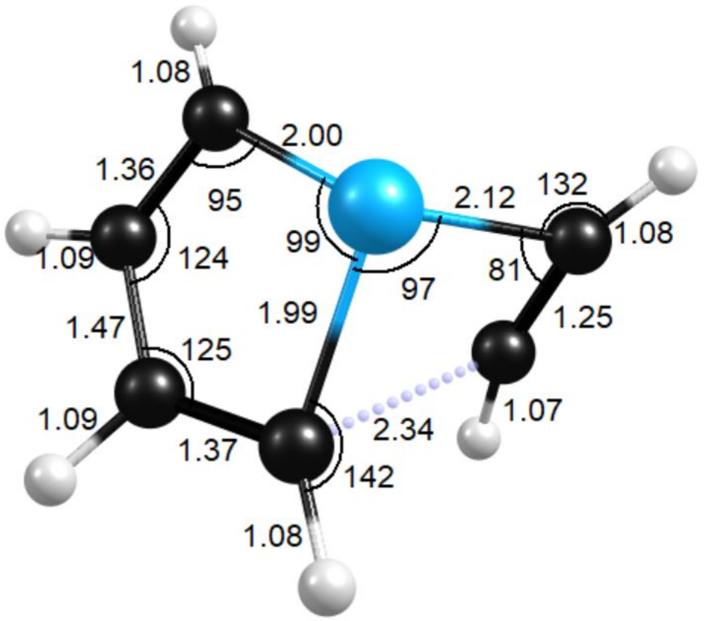

Frequency (cm^{-1})	Intensity (km/mol)	Frequency (cm^{-1})	Intensity (km/mol)
-271.672224	32.6756	861.811776	6.5271
99.75792	6.4215	922.643136	7.156
137.2224	3.7926	963.402336	2.6043
154.920288	2.161	1054.322496	16.2526
243.243936	14.2088	1064.665728	4.6111
302.247552	21.5223	1218.152832	27.4224
340.5768	8.0589	1262.73888	21.9767
458.716608	6.1863	1349.523936	3.4272
496.930944	7.3906	1474.890816	4.0917
590.93808	65.3999	1701.024768	77.6952
595.191552	17.7607	2996.395008	0.6653
642.976704	130.6058	3009.71568	1.9085
663.214752	46.0518	3056.265888	7.0566
719.844576	36.0455	3064.854816	6.4627
723.100608	15.2851	3114.968448	113.5391
781.199808	5.4448	3207.808512	58.7645
846.2136	15.8476		

Figure S51. The optimized geometry of the TS2 quartet $Ti^{+}(C_2H_2)_3$ followed by its predicted frequencies (cm^{-1}) and IR intensities (km/mol).

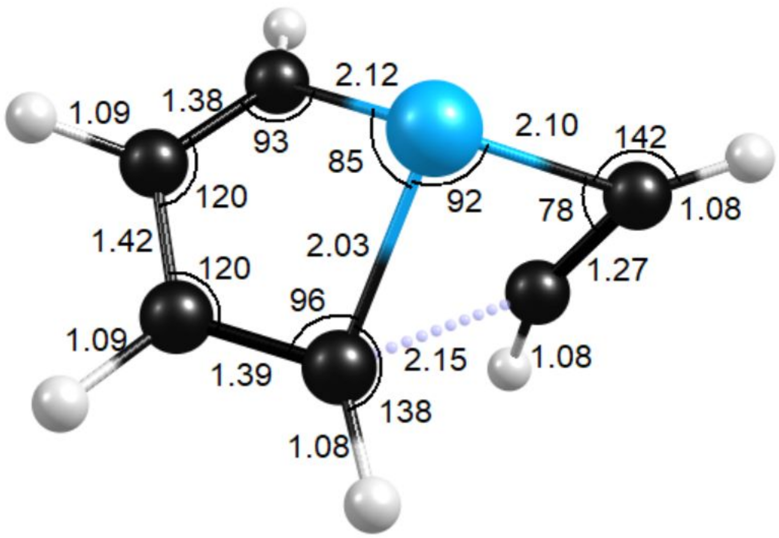

Frequency (cm^{-1})	Intensity (km/mol)	Frequency (cm^{-1})	Intensity (km/mol)
-311.106144	24.0937	864.367776	9.4163
76.236288	1.4243	928.83504	7.2892
125.654784	0.5384	933.733152	4.3039
150.666528	2.0026	986.145408	4.9705
296.899776	1.6266	1071.294624	7.2966
321.132	32.6219	1090.766112	45.9583
364.979808	1.4643	1208.064	4.8688
404.412576	16.2269	1347.525792	55.6477
511.489728	13.5636	1396.732896	4.6502
525.768288	7.6618	1603.869504	71.6134
590.298432	28.3655	2966.4	2.1798
640.720032	52.0148	3012.589056	0.339
663.941856	47.5029	3052.061664	1.5571
702.65328	28.8255	3062.349888	29.4073
752.049888	59.8213	3080.111232	56.3094
767.869152	74.657	3140.44608	32.7565
793.503648	67.424		

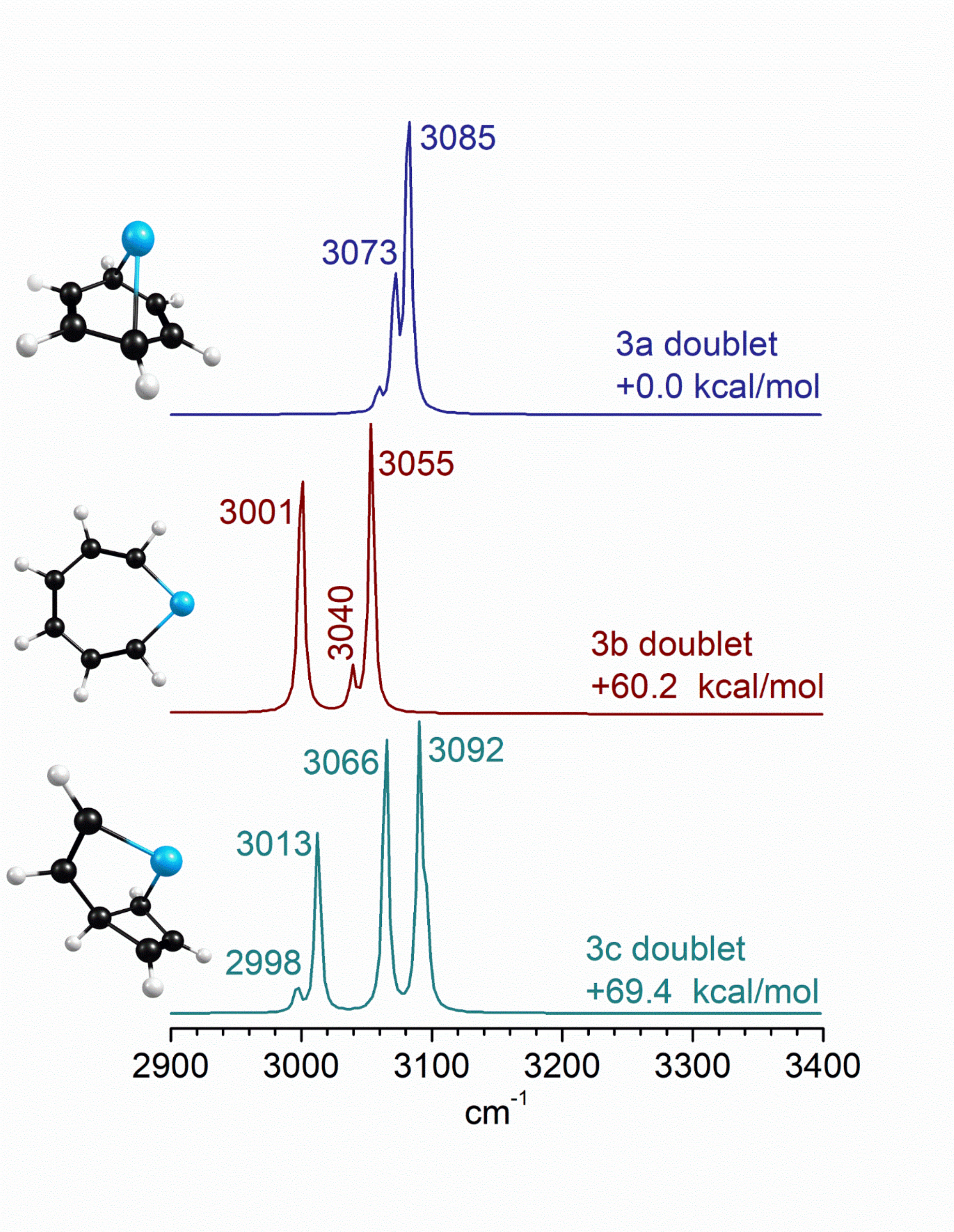

Figure S52. Predicted infrared spectra for $Ti^{+}(C_2H_2)_3$ isomers.

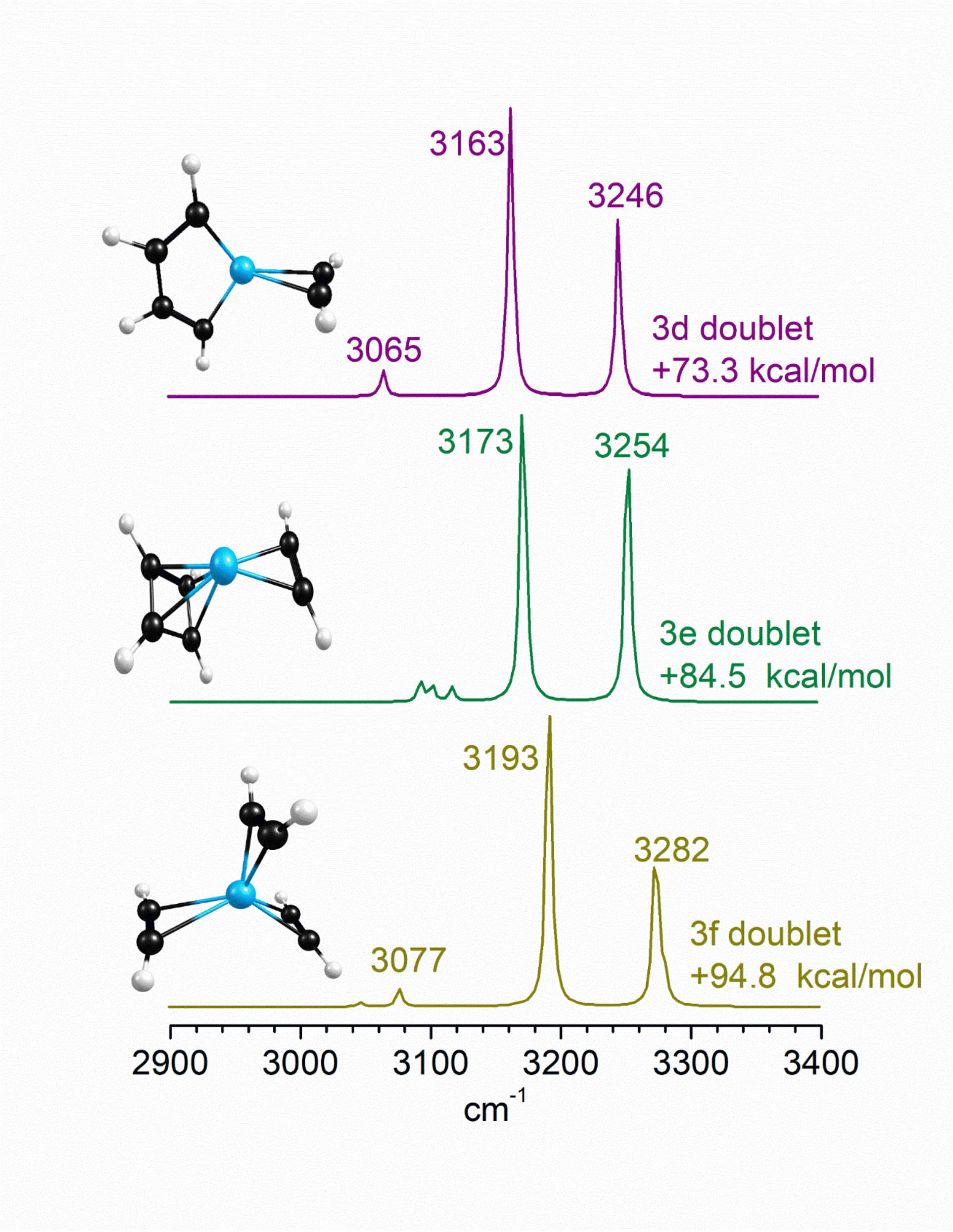

Figure S53. Predicted infrared spectra for $Ti^{+}(C_2H_2)_3$ isomers.

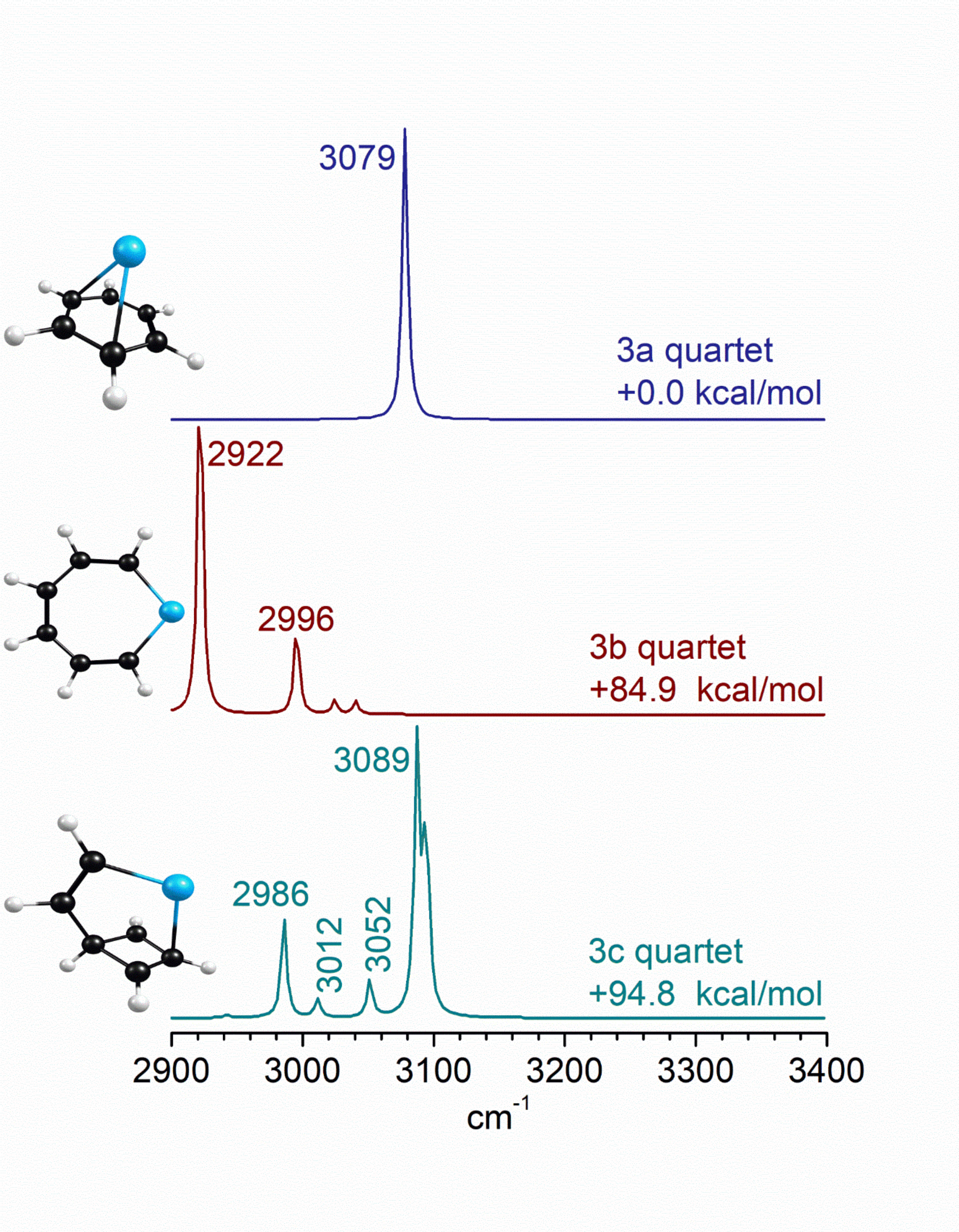

Figure S54. Predicted infrared spectra for $Ti^+(C_2H_2)_3$ isomers.

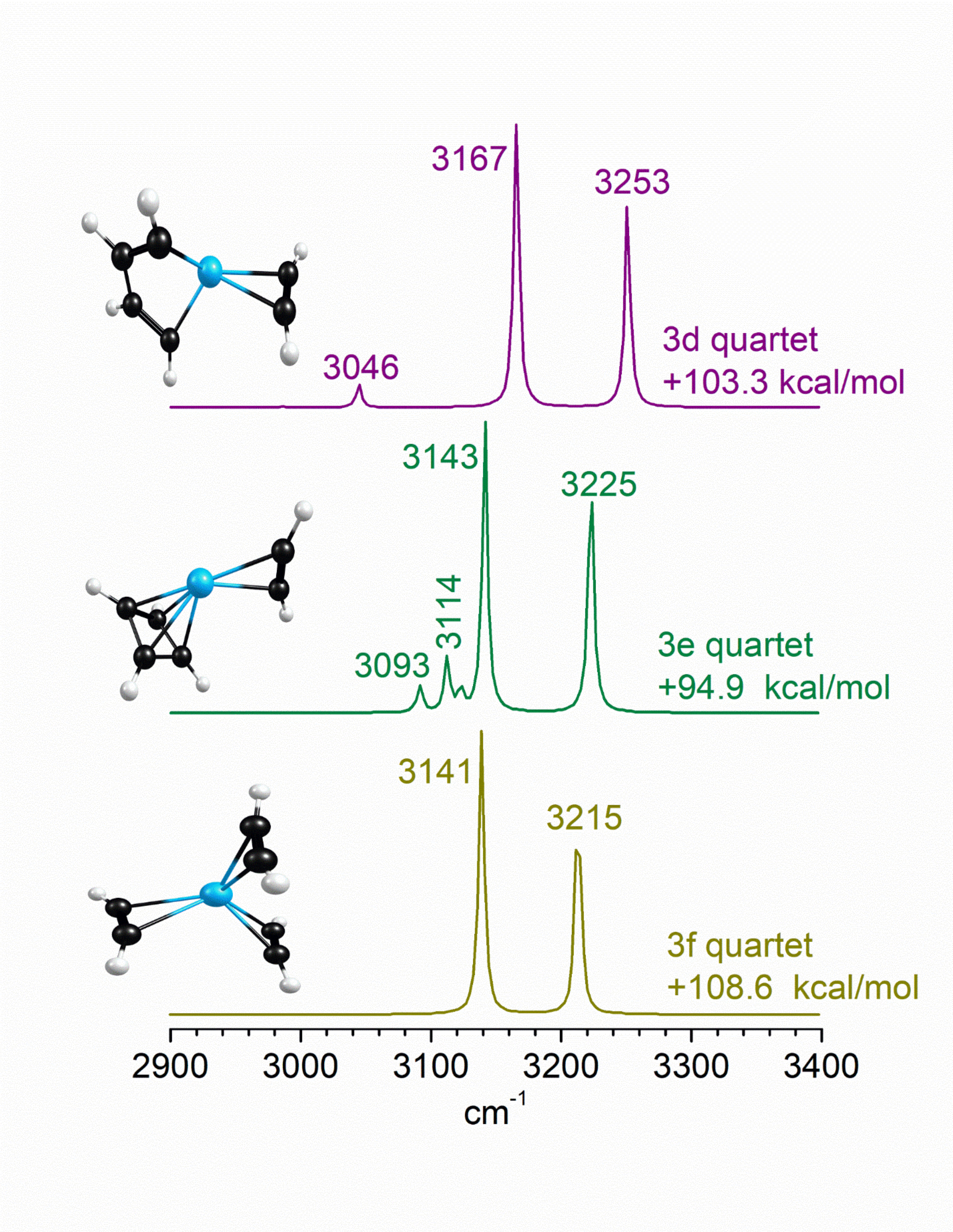

Figure S55. Predicted infrared spectra for $Ti^+(C_2H_2)_3$ isomers.

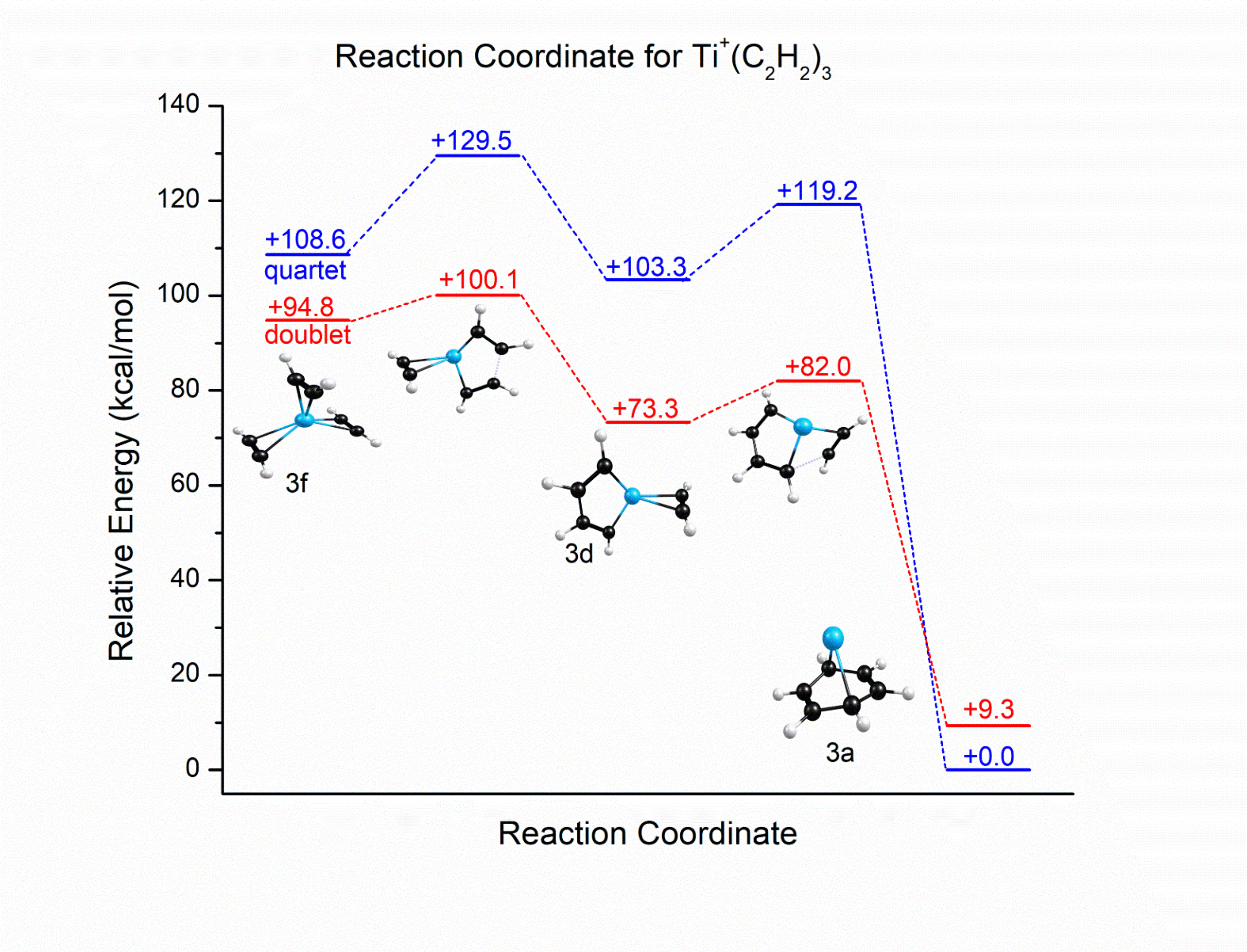

Figure S56. Reaction coordinate for the $Ti^{+}(C_2H_2)_3$ complex

Table S49. n = 4 energy table and structures

Isomer	2s + 1	E (hartree)	Relative E (kcal/mol)
4a	2	-1158.869238	0.0
4b	2	-1158.848575	+13.0
4c	2	-1158.785913	+52.3
4d	2	-1158.773801	+59.9
4e	2	-1158.738975	+81.7
4f	2	-1158.728868	+88.0
4g	2	-1158.728100	+88.9
4h	2	-1158.717138	+95.4
4i	2	-1158.708675	+100.8
4j	2	-1158.690344	+112.3
4a	4	-1158.802506	+41.9
4b	4	-1158.833523	+22.4
4c	4	-1158.779466	+56.3
4d	4	-1158.730719	+87.0
4e	4	-1158.698062	+107.4
4f	4	-1158.684073	+116.2
4g	4	-1158.678969	+119.4
4h	4	-1158.660818	+130.8
4i	4	-1158.65322	+135.6
4j	4	-1158.673643	+122.7
4k	4	-1158.641085	+143.2

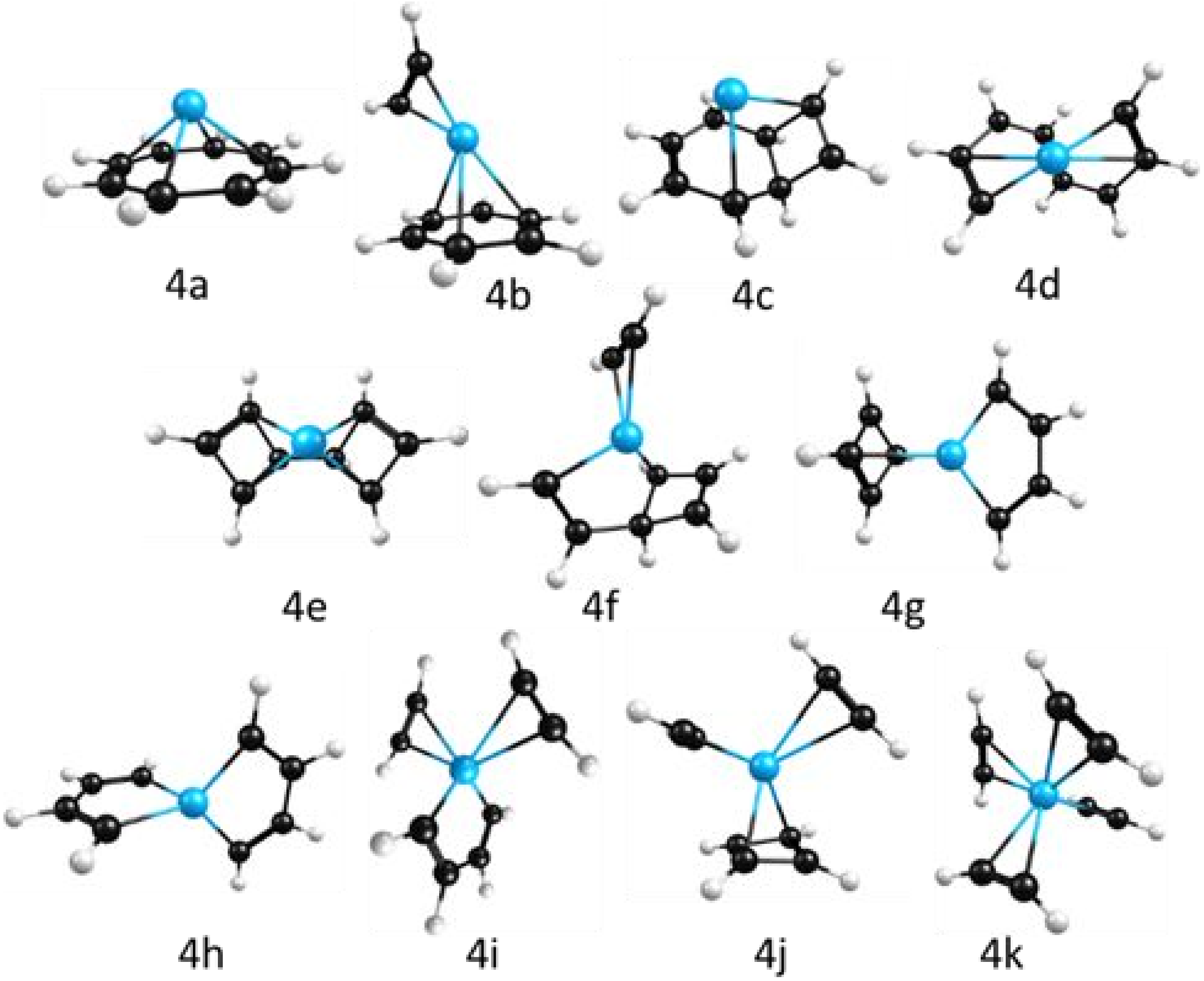

Figure S57. Isomers of $Ti^{+}(C_2H_2)_4$.

Table S50. Cartesian coordinates for the optimized geometry of isomer 4a-doublet $Ti^+(C_2H_2)_4$ followed by its predicted frequencies (cm^{-1}) and IR intensities (km/mol).

Z	x	y	z
6	1.225506000	-1.373583000	-0.400774000
6	1.837836000	-0.104707000	-0.400771000
6	1.373585000	1.225505000	-0.400773000
6	-1.837835000	0.104707000	-0.400773000
6	-1.225505000	1.373583000	-0.400775000
6	0.104707000	1.837833000	-0.400780000
1	-1.937194000	2.171267000	-0.225367000
1	1.937195000	-2.171267000	-0.225369000
1	2.905122000	-0.165512000	-0.225361000
1	2.171266000	1.937196000	-0.225367000
1	-2.905121000	0.165512000	-0.225360000
1	0.165512000	2.905120000	-0.225378000
22	-0.000001000	0.000000000	0.956371000
1	-0.165512000	-2.905120000	-0.225377000
6	-0.104707000	-1.837832000	-0.400780000
6	-1.373584000	-1.225505000	-0.400775000
1	-2.171265000	-1.937197000	-0.225367000

Frequency (cm^{-1})	Intensity (km/mol)	Frequency (cm^{-1})	Intensity (km/mol)
237.6916	0	1009.7405	0
237.7287	0	1219.4031	0
333.9404	4.179	1219.4157	0
334.9067	2.0669	1359.2886	0
334.9086	2.0669	1364.2291	0
393.7632	0	1364.2304	0
393.7784	0	1435.6662	0
494.9352	0	1484.6564	1.0986
494.9361	0	1484.6567	1.0986
591.5438	0	1522.0156	0
767.6405	0	1531.3664	0
767.6414	0	1531.3674	0
774.5921	57.2264	1547.226	0
787.4286	100.2295	1547.2376	0
840.4117	0.3727	3155.2286	0
840.4121	0.3728	3158.9571	0
926.5292	0	3158.9575	0
926.5404	0	3169.206	0
929.7218	8.1537	3169.2094	0
929.723	8.1538	3179.7445	4.2454
964.255	0	3179.7448	4.2454
981.8153	0	3184.6806	0.091
981.8156	0		

Table S51. Cartesian coordinates for the optimized geometry of isomer 4b-doublet $Ti^+(C_2H_2)_4$ followed by its predicted frequencies (cm^{-1}) and IR intensities (km/mol).

Z	x	y	z
6	1.695057000	-1.121727000	-0.203598000
6	1.719759000	-0.452140000	1.020100000
6	1.193592000	-0.473824000	-1.350181000
1	2.095344000	-0.953936000	1.902965000
1	1.172054000	-0.990336000	-2.300176000
6	1.235575000	0.867817000	1.116434000
6	0.783299000	0.867171000	-1.265342000
1	1.245511000	1.380457000	2.068877000
1	0.423112000	1.375797000	-2.149562000
6	0.805282000	1.539484000	-0.037630000
1	0.476003000	2.566973000	0.023395000
1	2.055347000	-2.140064000	-0.273447000
22	-0.714549000	-0.460512000	0.232613000
1	-2.780302000	1.675357000	-0.092961000
6	-2.309209000	0.707674000	0.020363000
1	-3.629110000	-1.069254000	-0.081632000
6	-2.679668000	-0.553411000	-0.002637000

Frequency (cm^{-1})	Intensity (km/mol)	Frequency (cm^{-1})	Intensity (km/mol)
30.0973	2.1214	1039.1721	16.8947
92.623	1.4821	1040.3535	35.8861
126.5064	1.1105	1045.9945	0.8463
204.0518	1.0547	1052.2425	1.7479
235.3184	1.3453	1184.5878	0.9925
249.5091	1.0106	1189.6886	0.1878
386.9964	2.8335	1199.945	3.1493
415.0221	0.7713	1335.8726	1.9026
563.3258	11.3681	1386.4925	0.0023
612.2128	1.7249	1498.3712	19.3049
613.0907	19.0001	1503.78	22.4405
616.301	6.4533	1524.1588	43.9887
689.6922	0.241	1566.6527	0.6221
704.0601	72.4066	1593.2999	10.9718
761.9835	37.6301	3172.8598	5.4196
797.5054	68.4352	3188.5433	0.1598
911.4773	0.2958	3198.1255	0.8106
914.5137	0.7712	3201.4821	1.6261
924.6758	0.8407	3204.4366	6.3231
991.2789	0.8143	3207.1808	6.0156
1005.9224	5.2469	3211.6927	7.9423
1022.0978	2.4809	3221.5632	3.0872
1026.1363	7.135		

Table S52. Cartesian coordinates for the optimized geometry of isomer 4c-doublet $Ti^+(C_2H_2)_4$ followed by its predicted frequencies (cm^{-1}) and IR intensities (km/mol).

Z	x	y	z
22	-0.256321000	0.651944000	0.958196000
6	1.961591000	-0.542627000	0.200352000
1	2.740639000	-1.056016000	0.748503000
6	1.681840000	0.779381000	0.016475000
1	2.231746000	1.682314000	0.272015000
6	-0.199276000	-1.544161000	0.233067000
6	-1.517734000	-1.085818000	0.129842000
6	0.877716000	-1.071930000	-0.712984000
1	-2.299387000	-1.576765000	0.696535000
1	1.156334000	-1.757558000	-1.516786000
6	-1.830174000	0.085939000	-0.588925000
6	0.610919000	0.423904000	-1.153913000
1	-2.856798000	0.428089000	-0.625090000
1	1.081592000	0.712492000	-2.092150000
6	-0.813216000	0.923758000	-1.086874000
1	-1.062985000	1.836103000	-1.612173000
1	0.017916000	-2.422089000	0.826584000

Frequency (cm^{-1})	Intensity (km/mol)	Frequency (cm^{-1})	Intensity (km/mol)
165.4445	1.7526	1042.125	2.881
264.5952	3.0997	1056.7501	11.0138
296.0519	0.6379	1110.5243	15.6062
344.19	9.9395	1157.1104	1.5883
371.5385	4.4024	1160.5575	4.5153
388.8044	1.6806	1214.2	8.3124
482.3447	0.0867	1232.4302	0.9525
529.2445	3.2738	1271.5589	21.9377
551.1243	4.8697	1289.0863	4.7785
606.2403	1.2303	1365.5956	7.7483
693.0141	3.288	1416.9147	5.2889
739.0934	8.5954	1494.2717	4.4227
782.0578	23.4625	1501.9389	4.3738
813.8965	6.8105	1511.2021	6.3237
830.8549	64.2002	3052.1728	6.7516
861.4815	9.7718	3106.2451	3.9271
893.0201	4.9433	3135.4777	1.2835
924.7566	5.0479	3176.1283	0.0924
953.9336	9.7268	3184.3472	0.8105
961.7924	1.5081	3192.7591	2.5765
974.5151	5.0214	3197.4503	1.4643
977.3899	8.2373	3210.2717	3.6513
1006.065	3.5561		

Table S53. Cartesian coordinates for the optimized geometry of isomer 4d-doublet $Ti^+(C_2H_2)_4$ followed by its predicted frequencies (cm^{-1}) and IR intensities (km/mol).

Z	x	y	z
6	-1.686853000	0.470097000	1.046053000
6	-2.320934000	-0.036686000	-0.036087000
6	-1.504067000	-0.744891000	-1.006017000
6	1.504069000	-0.744896000	1.006012000
6	0.267645000	-1.250707000	0.681538000
6	-0.267641000	-1.250700000	-0.681547000
1	-0.339388000	-1.695349000	1.461857000
1	-2.188071000	0.890371000	1.913658000
1	-3.383052000	0.079655000	-0.244618000
1	-1.863577000	-0.860949000	-2.021563000
1	1.863581000	-0.860962000	2.021557000
1	0.339395000	-1.695330000	-1.461870000
22	-0.000002000	0.996303000	0.000004000
1	2.188068000	0.890387000	-1.913652000
6	1.686851000	0.470107000	-1.046050000
6	2.320935000	-0.036682000	0.036086000
1	3.383054000	0.079654000	0.244615000

Frequency (cm^{-1})	Intensity (km/mol)	Frequency (cm^{-1})	Intensity (km/mol)
153.16	10.1961	1004.535	11.7369
155.8669	7.1918	1048.6588	1.3065
180.9186	0.3196	1059.9442	9.6328
226.3485	0.055	1073.9435	6.6209
302.0826	15.7044	1192.0422	1.5513
349.4132	0.0424	1219.4464	0.0576
389.0739	4.4692	1269.9337	56.8384
416.4803	1.9014	1280.2954	15.2103
478.1147	0.006	1379.9081	11.7286
506.9739	1.4073	1447.5314	1.6011
574.5732	36.1908	1472.7419	7.0185
584.2611	0.0919	1486.1061	15.5628
716.9373	31.4362	1557.3875	14.0287
737.5922	128.3967	1575.1052	0.0002
764.754	7.6985	3114.5157	0.0001
794.9194	0.4438	3114.8693	6.0617
831.2313	35.1095	3153.0359	13.1411
856.1272	5.0982	3153.2194	0.4255
919.0971	4.0473	3165.6854	1.5296
964.7121	0.8758	3171.0963	5.7848
974.3417	14.3196	3185.6985	2.4736
987.535	8.9302	3185.7228	0.0782
994.7481	1.9567		

Table S54. Cartesian coordinates for the optimized geometry of isomer 4e-doublet $Ti^+(C_2H_2)_4$ followed by its predicted frequencies (cm^{-1}) and IR intensities (km/mol).

Z	x	y	z
6	1.544256000	-0.190934000	-1.020791000
6	0.798814000	1.063072000	-0.406145000
6	1.506742000	0.635506000	0.894869000
6	2.331778000	-0.221342000	0.162245000
1	1.787783000	-0.424656000	-2.047433000
1	1.220525000	1.967580000	-0.835851000
1	1.595071000	1.104933000	1.862152000
1	3.243590000	-0.751106000	0.412955000
22	-0.025634000	-0.903094000	0.251867000
6	-0.770370000	1.085737000	-0.389374000
1	-1.171616000	2.027486000	-0.751053000
6	-1.549258000	-0.110067000	-1.032147000
6	-1.472081000	0.613768000	0.910739000
1	-1.551641000	1.068159000	1.886403000
1	-1.733905000	-0.361271000	-2.066336000
6	-2.323072000	-0.210144000	0.148952000
1	-3.226700000	-0.756641000	0.387997000

Frequency (cm^{-1})	Intensity (km/mol)	Frequency (cm^{-1})	Intensity (km/mol)
49.8851	0.9424	997.303	12.3542
147.7583	0.7251	1006.8951	1.0833
283.7708	3.7222	1017.0426	1.2133
287.6036	6.6123	1026.3475	1.7522
321.7532	0.8948	1119.9873	7.62
370.2673	0.3416	1142.8506	10.8436
406.6401	15.4293	1180.5849	5.5907
473.6808	4.1582	1196.0721	0.426
490.9767	7.6657	1198.3421	6.3332
662.7172	8.6135	1268.7276	1.3721
677.1233	37.6717	1366.2592	7.1952
728.1758	9.7034	1374.0193	1.6725
768.2164	7.1139	1390.4185	0.3999
777.5443	24.6254	1422.0718	2.3622
797.4933	32.2564	3125.8902	4.021
835.8063	57.097	3140.0644	22.5939
874.4613	6.7192	3184.1613	1.2351
915.2932	11.3583	3194.3595	0.7315
926.3462	11.232	3213.191	5.3078
938.0291	93.2085	3222.1415	9.3663
942.7828	46.2947	3233.2982	5.1992
957.1142	1.97	3237.5365	6.1762
972.36	10.9755		

Table S55. Cartesian coordinates for the optimized geometry of isomer 4f-doublet $Ti^{+}(C_2H_2)_4$ followed by its predicted frequencies (cm^{-1}) and IR intensities (km/mol).

Z	x	y	z
6	1.399697000	-0.994102000	-0.885378000
6	1.742297000	-0.031271000	0.276738000
6	0.743257000	-0.940642000	1.070271000
6	0.787181000	-1.875040000	-0.000637000
1	1.768627000	-1.067542000	-1.898499000
1	2.755583000	-0.195635000	0.631535000
1	0.598345000	-1.054590000	2.134712000
1	0.416499000	-2.886682000	-0.107352000
22	-0.466363000	0.187733000	-0.300422000
6	1.490257000	1.498080000	0.133668000
1	2.397289000	2.097476000	0.169246000
6	0.268546000	2.022560000	-0.028454000
1	0.079148000	3.085966000	-0.097354000
6	-2.653941000	-0.247499000	-0.439206000
6	-2.449527000	-0.054081000	0.758364000
1	-2.542724000	0.080641000	1.818464000
1	-3.179366000	-0.457809000	-1.353665000

Frequency (cm^{-1})	Intensity (km/mol)	Frequency (cm^{-1})	Intensity (km/mol)
67.9197	0.8456	959.8198	9.2748
98.3727	0.5675	965.0631	53.1689
114.8784	1.1893	992.0868	7.5679
123.9169	10.1893	995.3006	5.826
161.9112	0.067	1017.6815	0.7635
248.7535	16.4749	1078.4019	6.0916
314.7895	3.6242	1167.7542	6.1929
363.3411	4.7484	1199.7183	3.9671
371.0912	4.8977	1241.1692	2.361
403.6652	6.7894	1298.6711	23.0341
446.136	11.1681	1364.663	5.3225
600.606	17.6897	1438.0253	2.5013
644.6448	33.8053	1553.8815	4.5718
661.3163	14.636	1865.0082	100.4168
668.0259	40.4805	3117.7353	2.342
671.7245	7.8291	3132.8623	5.5162
706.2859	6.9574	3195.6773	8.9857
736.0344	68.8794	3196.7398	2.2442
741.5819	68.5344	3217.7076	7.5532
789.2171	13.4572	3225.4952	4.8918
848.3831	38.2442	3278.4662	132.288
917.664	11.6209	3363.6391	106.839
924.7257	16.4706		

Table S56. Cartesian coordinates for the optimized geometry of isomer 4g-doublet $Ti^+(C_2H_2)_4$ followed by its predicted frequencies (cm^{-1}) and IR intensities (km/mol).

Z	x	y	z
6	2.310603000	-0.757511000	0.121117000
6	2.310576000	0.757546000	0.121128000
6	1.243304000	-1.579648000	-0.011462000
1	3.313732000	1.153798000	0.259384000
1	1.339401000	-2.655747000	0.049970000
6	1.243254000	1.579654000	-0.011487000
1	1.339308000	2.655755000	0.049971000
1	3.313777000	-1.153729000	0.259331000
22	0.089315000	-0.000015000	-0.326120000
6	-1.978690000	1.005629000	0.199864000
6	-1.539398000	0.000026000	1.152607000
1	-1.380969000	0.000055000	2.219803000
1	-2.157836000	2.068924000	0.251589000
1	-2.522334000	-0.000060000	-1.829732000
6	-2.119656000	-0.000028000	-0.827919000
1	-2.157836000	-2.068919000	0.251702000
6	-1.978690000	-1.005627000	0.199921000

Frequency (cm^{-1})	Intensity (km/mol)	Frequency (cm^{-1})	Intensity (km/mol)
57.7453	0.0429	969.8928	18.9227
59.3307	0.2188	992.0156	0.8882
100.3114	10.8536	1015.5607	0.3296
110.5641	1.0512	1083.4469	0.0217
206.562	9.7821	1084.889	11.7936
239.4506	0.4926	1113.6471	3.9914
253.2162	2.0537	1194.5523	0.0124
324.0829	3.772	1257.6439	0.4997
438.5402	8.3102	1298.0353	18.9687
482.5845	3.7076	1300.8068	29.5028
586.1869	31.9939	1341.931	10.3408
637.0089	60.9423	1357.7068	2.065
672.1524	60.0481	1422.238	13.0788
709.2936	71.5516	1569.3969	3.5292
748.407	5.7467	3116.5137	0.1041
765.5603	14.9867	3130.7451	0.001
767.9786	50.8871	3197.2276	3.7819
773.4168	8.2388	3197.6144	10.1025
827.737	6.5418	3223.5694	3.7407
840.9471	0.0241	3236.9716	21.58
874.793	2.4756	3239.6241	15.7852
905.3896	5.2395	3254.0224	12.5866
953.9812	28.0958		

Table S57. Cartesian coordinates for the optimized geometry of isomer 4h-doublet $Ti^{+}(C_2H_2)_4$ followed by its predicted frequencies (cm^{-1}) and IR intensities (km/mol).

Z	x	y	z
22	-0.145815000	0.000002000	-0.201631000
6	1.674255000	0.000010000	-1.345485000
6	2.776330000	0.000002000	-0.496301000
1	3.797451000	0.000002000	-0.870906000
1	1.876679000	0.000016000	-2.415198000
6	2.561455000	-0.000009000	0.911223000
6	1.256530000	-0.000001000	1.392788000
1	1.114886000	0.000001000	2.471415000
1	3.422611000	-0.000022000	1.575720000
1	-1.470215000	2.616986000	-0.061111000
6	-1.399201000	1.536332000	-0.062115000
6	-2.493277000	0.745738000	0.106291000
1	-3.482507000	1.176538000	0.246892000
6	-2.493274000	-0.745745000	0.106283000
1	-3.482503000	-1.176550000	0.246879000
6	-1.399195000	-1.536333000	-0.062129000
1	-1.470206000	-2.616988000	-0.061134000

Frequency (cm^{-1})	Intensity (km/mol)	Frequency (cm^{-1})	Intensity (km/mol)
19.2214	0.4834	1017.2727	0.0013
54.2554	1.6584	1021.8556	0.0551
122.2666	0.0024	1095.1028	25.512
135.1788	10.7515	1112.23	14.7876
162.3769	0.0828	1114.6749	4.0458
195.8542	0.0862	1140.6011	3.2197
201.9016	2.9482	1296.9151	3.8684
276.4441	2.1288	1299.695	26.8418
278.1728	0.0294	1332.4626	108.4419
448.3187	1.5841	1345.329	2.2696
482.2244	1.5616	1428.8083	101.8595
548.1299	8.1243	1475.1704	70.4804
617.7547	84.6224	1505.4081	9.1857
618.9829	38.6706	1553.7859	5.7202
667.1234	22.5682	3113.4246	0.3229
726.7571	11.0175	3123.4665	1.0893
729.8582	3.121	3126.7415	0.9002
755.146	61.7096	3131.8441	1.5551
824.6067	0.0314	3143.5161	0.622
872.5417	11.0301	3151.1262	0.5724
951.0546	0.1917	3188.6049	0.9528
990.5085	0.7378	3188.6476	11.06
998.9369	0.0617		

Table S58. Cartesian coordinates for the optimized geometry of isomer 4i-doublet $Ti^{+}(C_2H_2)_4$ followed by its predicted frequencies (cm^{-1}) and IR intensities (km/mol).

Z	x	y	z
22	-0.179896000	0.000016000	0.000000000
6	1.072227000	-0.000027000	1.549806000
6	2.165360000	0.000008000	0.751909000
1	3.169958000	0.000003000	1.170552000
1	1.155512000	-0.000057000	2.629485000
6	2.165360000	0.000058000	-0.751908000
6	1.072228000	0.000100000	-1.549806000
1	1.155515000	0.000139000	-2.629485000
1	3.169959000	0.000057000	-1.170550000
6	-1.018173000	2.175084000	0.000120000
6	-2.062560000	1.555853000	-0.000114000
1	-3.080426000	1.233024000	-0.000335000
1	-0.208382000	2.876113000	0.000307000
1	-0.208139000	-2.876083000	-0.000319000
6	-1.017998000	-2.175132000	-0.000119000
6	-2.062438000	-1.555993000	0.000112000
1	-3.080329000	-1.233243000	0.000343000

Frequency (cm^{-1})	Intensity (km/mol)	Frequency (cm^{-1})	Intensity (km/mol)
39.6736	0	768.2184	161.8517
71.3072	0.6835	795.9877	1.0253
89.6285	0.0983	840.6947	0
102.4176	0	853.699	5.1533
122.0414	1.6873	999.4919	0.4514
138.8749	11.3729	1025.0607	0
141.323	0.2444	1095.5076	18.0057
194.8878	0.0088	1115.9894	4.1224
218.4459	11.6988	1304.7228	27.9833
255.0726	0	1345.4764	18.247
293.703	10.6579	1452.1208	19.8474
329.1267	2.3026	1575.1418	2.2168
336.377	0.3383	1954.761	33.1593
474.2453	5.7231	1969.7417	8.8853
625.418	0	3106.6878	0.5884
627.2427	58.7445	3120.823	2.6205
640.3275	0.2437	3186.9559	1.5467
645.7176	32.769	3187.2831	4.5585
656.6319	7.4456	3336.4488	119.9662
699.571	55.707	3340.0586	148.7466
706.7795	2.0387	3428.2454	83.6714
739.9951	23.4784	3434.4768	62.251
741.9139	0		

Table S59. Cartesian coordinates for the optimized geometry of isomer 4j-doublet $Ti^{+}(C_2H_2)_4$ followed by its predicted frequencies (cm^{-1}) and IR intensities (km/mol).

Z	x	y	z
6	-1.554924000	0.975136000	0.877414000
6	-1.513855000	1.330340000	-0.527656000
6	-1.648755000	-0.051045000	-0.926852000
6	-1.959352000	-0.334700000	0.500317000
1	-1.552547000	1.530907000	1.801846000
1	-1.494166000	2.269750000	-1.061903000
1	-1.966184000	-0.519155000	-1.843689000
1	-2.438827000	-1.154184000	1.012668000
22	0.303382000	-0.128547000	0.015223000
6	1.950276000	1.791140000	-0.058113000
6	2.622815000	0.807384000	0.132977000
1	3.286108000	-0.014287000	0.301631000
1	1.462801000	2.727257000	-0.224375000
1	0.414971000	-2.757450000	1.394537000
6	0.476376000	-2.000795000	0.622208000
6	0.725714000	-1.950548000	-0.667464000
1	1.023680000	-2.656286000	-1.432619000

Frequency (cm^{-1})	Intensity (km/mol)	Frequency (cm^{-1})	Intensity (km/mol)
23.3745	0.4791	794.2477	35.8055
63.6402	0.146	869.8065	4.4137
87.4609	1.2749	909.7224	1.0761
92.7929	0.2889	914.798	7.4872
121.2235	2.0169	948.7448	14.0453
143.1785	0.3159	970.7201	14.9419
224.1967	1.6352	1026.4178	69.151
239.6167	1.8104	1075.6002	1.5597
244.7019	10.0652	1194.932	0.0906
350.8226	1.041	1251.9319	3.8677
426.5751	1.4206	1300.3553	21.3296
477.5228	30.2026	1372.8701	3.8362
570.0615	6.2037	1517.4893	17.6713
585.6509	15.7326	2014.11	0.1335
635.5004	0.2946	3174.6124	4.166
666.7946	0.4001	3204.8667	7.8764
700.3408	11.5314	3217.7407	1.6673
732.7749	30.8598	3237.9688	7.6961
735.0836	56.7311	3250.1013	15.1355
751.9503	16.5445	3260.4383	9.8334
774.2496	28.5231	3355.0102	166.4372
777.651	72.2745	3451.6921	46.7533
793.5166	23.0699		

Table S60. Cartesian coordinates for the optimized geometry of isomer 4a-quartet $Ti^{+}(C_2H_2)_4$ followed by its predicted frequencies (cm^{-1}) and IR intensities (km/mol).

Z	x	y	z
6	-1.866677000	-0.000518000	-0.469909000
6	-1.311810000	-1.296576000	-0.411886000
6	0.000514000	-1.818300000	-0.485532000
6	1.311811000	1.296576000	-0.411886000
6	1.866678000	0.000518000	-0.469907000
6	1.312932000	-1.295491000	-0.411911000
1	2.950910000	0.001019000	-0.457248000
1	-2.950909000	-0.001019000	-0.457255000
1	-2.061568000	-2.074556000	-0.340921000
1	0.000998000	-2.901135000	-0.429654000
1	2.061569000	2.074556000	-0.340920000
1	2.063087000	-2.073096000	-0.341037000
22	-0.000001000	0.000000000	1.113117000
1	-2.063086000	2.073096000	-0.341037000
6	-1.312931000	1.295491000	-0.411910000
6	-0.000513000	1.818301000	-0.485532000
1	-0.000997000	2.901136000	-0.429655000

Frequency (cm^{-1})	Intensity (km/mol)	Frequency (cm^{-1})	Intensity (km/mol)
173.6889	0	985.2844	6.6142
201.7915	4.092	1001.7135	4.1173
216.7486	0.0856	1194.4141	0.0033
240.4487	1.7968	1198.9981	0
264.8843	17.474	1331.0661	33.526
300.8141	0.0035	1351.7357	27.6424
334.284	0.0015	1356.4054	0
383.0584	0.0638	1441.6436	0
439.2742	1.34	1475.4492	39.8262
445.469	0.2579	1477.8814	58.3916
578.8038	16.5054	1494.8492	9.1054
741.2075	103.5084	1503.7043	32.6003
749.8174	2.9514	1538.3101	0.0048
750.9829	0.6905	1543.1055	0
761.5527	0.082	3154.1423	0.0113
816.9467	0.7042	3156.1506	0.2022
820.6128	0.8684	3159.0285	0.0091
885.8969	0	3163.5643	0.1458
896.1373	0.3624	3173.0202	0
912.7732	0.0053	3178.8454	0.8138
962.844	1.0495	3178.9858	0.5143
973.4315	4.6578	3183.2135	0.1057
979.1802	1.5694		

Table S61. Cartesian coordinates for the optimized geometry of isomer 4b-quartet $Ti^{+}(C_2H_2)_4$ followed by its predicted frequencies (cm^{-1}) and IR intensities (km/mol).

Z	x	y	z
6	1.747014000	-1.079374000	0.000137000
6	1.479420000	-0.434377000	1.221924000
6	1.479541000	-0.434558000	-1.221774000
1	1.681139000	-0.936265000	2.158121000
1	1.681346000	-0.936589000	-2.157876000
6	0.971000000	0.881869000	1.220440000
6	0.971120000	0.881688000	-1.220539000
1	0.779976000	1.387258000	2.157001000
1	0.780188000	1.386936000	-2.157194000
6	0.743512000	1.544920000	-0.000110000
1	0.368985000	2.559347000	-0.000205000
1	2.135953000	-2.089337000	0.000231000
22	-0.580390000	-0.452771000	-0.000025000
1	-2.742693000	1.691151000	0.000037000
6	-2.631230000	0.625521000	-0.000010000
1	-3.376404000	-1.519468000	-0.000018000
6	-2.850363000	-0.582701000	0.000009000

Frequency (cm^{-1})	Intensity (km/mol)	Frequency (cm^{-1})	Intensity (km/mol)
20.4959	0.5682	996.3423	1.2051
95.2661	0.0189	1031.1092	5.2416
100.3492	0.4357	1032.6551	3.8714
236.0285	0.0706	1038.5313	0.1052
266.128	6.6858	1174.3379	0.062
288.6349	0.3684	1177.1334	0.0231
316.2281	40.4291	1180.7558	0.0069
393.2689	9.6135	1333.5058	0.0048
411.0437	0.0646	1376.052	0.0002
422.6872	0.6203	1485.8241	12.2634
614.1375	1.6021	1486.2586	12.0935
617.9147	0.9228	1540.3297	0.0079
633.1516	57.9359	1540.8017	0.0655
642.4428	13.0189	1878.3567	166.8627
661.6378	1.4521	3191.4742	0.5409
670.192	34.6202	3198.0853	0.285
719.8564	73.7352	3202.3042	0.0177
768.2309	80.2618	3208.9954	3.0984
888.8658	1.6667	3212.4193	6.314
895.291	0.3496	3216.929	1.5243
965.9731	0.0024	3289.8052	116.6793
982.252	3.5599	3374.2777	109.0354
985.1479	0.0765		

Table S62. Cartesian coordinates for the optimized geometry of isomer 4c-quartet $Ti^{+}(C_2H_2)_4$ followed by its predicted frequencies (cm^{-1}) and IR intensities (km/mol).

Z	x	y	z
22	-0.307791000	-0.000304000	1.281735000
6	1.782664000	-0.681787000	0.181734000
1	2.361729000	-1.426248000	0.715509000
6	1.782665000	0.681705000	0.182051000
1	2.361730000	1.425916000	0.716174000
6	-0.487587000	-1.443163000	-0.655846000
6	-1.646961000	-0.723150000	-0.507976000
6	0.827959000	-0.794325000	-1.009650000
1	-2.583759000	-1.240426000	-0.340588000
1	1.271288000	-1.238114000	-1.905089000
6	-1.646962000	0.723391000	-0.507640000
6	0.827959000	0.794800000	-1.009277000
1	-2.583761000	1.240587000	-0.340016000
1	1.271288000	1.239007000	-1.904508000
6	-0.487589000	1.443474000	-0.655175000
1	-0.519994000	2.526063000	-0.623909000
1	-0.519991000	-2.525766000	-0.625076000

Frequency (cm^{-1})	Intensity (km/mol)	Frequency (cm^{-1})	Intensity (km/mol)
184.2858	0	1044.2374	6.4402
207.753	5.0864	1094.4557	2.7225
237.0766	2.1734	1142.0997	23.2495
259.917	0.0029	1180.2457	0.1126
321.1411	0.2957	1208.9058	5.4581
416.8681	0.9953	1210.1885	4.3283
444.3031	7.3214	1284.0301	9.0389
541.8656	0.0186	1290.548	7.7371
552.9437	3.9685	1305.3837	9.5825
569.3423	0.0635	1386.6736	7.1898
734.2222	4.133	1437.3214	2.6014
762.5783	29.9305	1507.5466	34.7765
807.8385	0.3641	1520.1434	4.8494
826.9685	38.0095	1575.9301	0.0012
828.0256	2.2452	3041.168	0.7364
902.8362	6.9015	3046.8766	5.0047
933.6977	18.5877	3169.7835	0.0692
944.9079	8.7601	3171.374	0.2417
966.7252	2.9477	3173.575	0.0263
971.7246	1.1759	3183.0588	0.426
983.7281	0.1579	3191.1045	1.6635
983.7887	1.4604	3193.6175	2.4342
1002.6276	0.6618		

Table S63. Cartesian coordinates for the optimized geometry of isomer 4d-quartet $Ti^{+}(C_2H_2)_4$ followed by its predicted frequencies (cm^{-1}) and IR intensities (km/mol).

Z	x	y	z
6	0.880457000	-1.477976000	-0.606723000
6	2.062730000	-0.803143000	-0.353383000
6	2.121973000	0.512887000	0.138528000
6	-0.804914000	0.484718000	-1.195528000
6	-0.189107000	1.512864000	-0.338035000
6	1.090090000	1.503978000	0.191129000
1	-0.810152000	2.364019000	-0.082304000
1	0.847672000	-2.363512000	-1.233167000
1	3.016729000	-1.326019000	-0.428426000
1	3.081054000	0.842774000	0.519503000
1	-0.418557000	0.313864000	-2.194069000
1	1.353552000	2.386422000	0.763676000
22	-0.279365000	-0.486764000	0.785125000
1	-3.295203000	-0.223338000	1.001720000
6	-2.304003000	-0.084590000	0.586441000
6	-2.025688000	-0.089690000	-0.774650000
1	-2.618301000	-0.639685000	-1.506366000

Frequency (cm^{-1})	Intensity (km/mol)	Frequency (cm^{-1})	Intensity (km/mol)
62.6824	1.2942	1011.7961	16.2506
103.7397	5.7623	1017.393	0.1456
166.4415	4.0149	1050.7507	12.6986
235.8065	1.9587	1117.642	9.2696
275.6737	1.7505	1173.952	14.8581
295.2477	3.9591	1195.6963	39.978
350.4373	7.304	1218.7547	22.94
394.5905	5.2317	1276.9613	1.384
457.7441	1.1318	1358.3117	7.0215
491.3581	3.3238	1391.6254	15.4498
510.3375	3.3518	1413.0777	8.0296
622.7736	27.1855	1440.6192	4.8165
648.8162	80.8594	1487.7231	6.5616
679.2656	26.0381	1531.3931	2.3137
686.8533	16.3622	3092.6592	0.7621
743.3841	50.857	3093.5504	0.4527
805.2486	1.3043	3156.707	1.4786
853.9699	5.032	3164.435	2.7676
864.6826	16.3659	3166.3522	3.6554
908.7153	16.0599	3169.6113	2.2728
913.5166	21.6379	3182.5848	1.8799
957.1182	7.8422	3184.7793	6.5464
986.5892	0.7718		

Table S64. Cartesian coordinates for the optimized geometry of isomer 4e-quartet $Ti^{+}(C_2H_2)_4$ followed by its predicted frequencies (cm^{-1}) and IR intensities (km/mol).

Z	x	y	z
6	1.464985000	0.309781000	-1.042315000
6	0.693086000	1.180099000	-0.000005000
6	1.464991000	0.309804000	1.042310000
6	2.311992000	-0.134376000	0.000003000
1	1.631911000	0.463061000	-2.098206000
1	1.107147000	2.184003000	-0.000022000
1	1.631902000	0.463076000	2.098205000
1	3.272150000	-0.633567000	0.000006000
22	0.105080000	-1.055099000	0.000003000
6	-0.876042000	1.231341000	-0.000001000
1	-1.233684000	2.262888000	0.000000000
6	-1.598551000	0.329765000	-1.021049000
6	-1.598549000	0.329765000	1.021049000
1	-1.762503000	0.421241000	2.085619000
1	-1.762505000	0.421240000	-2.085619000
6	-2.226012000	-0.423376000	0.000001000
1	-3.011579000	-1.166591000	0.000001000

Frequency (cm^{-1})	Intensity (km/mol)	Frequency (cm^{-1})	Intensity (km/mol)
141.4416	0.0656	964.8407	0.2992
188.307	0.7046	1007.5035	0.0019
211.9257	12.4044	1025.965	6.1828
234.8935	4.0977	1135.9546	5.2201
334.0124	1.033	1142.3168	23.1736
337.0174	3.2851	1159.0353	1.235
368.6041	0.5206	1200.0039	2.4899
468.6621	0.8732	1209.2842	4.0389
480.4438	3.9702	1211.2821	0.5339
614.8593	18.8397	1308.1293	22.1611
672.1227	42.7712	1317.3608	0.0853
748.653	5.8201	1369.037	9.187
754.4258	2.3449	1373.2123	0.3566
779.4481	3.8571	1397.3636	9.0012
797.3735	17.3858	3064.1507	1.6803
852.1295	8.3937	3131.3424	5.6413
886.0482	3.1236	3199.0084	0.2828
905.1157	0.8144	3203.6045	0.9359
919.2764	10.9647	3219.1334	2.7009
921.1306	38.8292	3219.4912	9.7126
934.958	24.8229	3226.3206	3.3842
943.6205	37.8596	3228.5427	8.2583
959.8424	6.5957		

Table S65. Cartesian coordinates for the optimized geometry of isomer 4f-quartet $Ti^{+}(C_2H_2)_4$ followed by its predicted frequencies (cm^{-1}) and IR intensities (km/mol).

Z	x	y	z
6	1.474618000	-0.949421000	-0.981878000
6	1.973695000	-0.013611000	0.146175000
6	1.130774000	-0.942106000	1.037056000
6	0.771061000	-1.753370000	-0.057658000
1	1.744686000	-1.051761000	-2.023755000
1	3.046054000	-0.128316000	0.316822000
1	1.023563000	-1.024428000	2.109317000
1	0.247413000	-2.696271000	-0.146953000
22	-0.632245000	0.200496000	-0.303371000
6	1.595745000	1.469675000	0.119772000
1	2.429734000	2.148006000	-0.049558000
6	0.335427000	1.891898000	0.309044000
1	0.117268000	2.954310000	0.356491000
6	-2.857232000	0.137693000	-0.312548000
6	-2.532708000	-0.530446000	0.670732000
1	-2.581475000	-1.123089000	1.564051000
1	-3.466130000	0.648757000	-1.036417000

Frequency (cm^{-1})	Intensity (km/mol)	Frequency (cm^{-1})	Intensity (km/mol)
68.6062	1.5165	942.438	6.3593
91.5327	1.484	960.3975	16.6808
127.6919	0.9963	964.8446	7.296
145.3861	10.0563	996.2566	1.9287
161.2726	11.9982	1093.656	4.0527
193.269	18.3972	1120.0008	6.9672
254.9329	0.6812	1170.0395	8.3427
332.1261	8.1075	1205.286	0.5194
357.3842	1.6191	1282.7992	1.5255
389.7855	5.6584	1307.0522	19.7278
398.2795	2.5326	1316.3202	14.058
543.2214	19.4346	1402.1911	5.2765
631.162	34.3791	1548.5196	0.5095
646.9158	33.2698	1859.1627	109.0986
659.2649	4.9058	3067.7378	0.2954
679.9709	19.8848	3112.6535	2.7859
694.9158	22.2116	3156.901	0.5472
705.4292	58.5871	3199.1866	1.0481
715.7352	72.0382	3221.3035	9.5028
744.3955	17.8239	3229.9261	5.5624
866.0109	16.7117	3277.2199	115.7537
897.4836	10.1738	3355.7865	98.7843
915.1195	16.0649		

Table S66. Cartesian coordinates for the optimized geometry of isomer 4g-quartet $Ti^{+}(C_2H_2)_4$ followed by its predicted frequencies (cm^{-1}) and IR intensities (km/mol).

Z	x	y	z
6	2.456105000	-0.756112000	0.000058000
6	2.456314000	0.755775000	0.000082000
6	1.386672000	-1.585022000	-0.000361000
1	3.470199000	1.149324000	0.000464000
1	1.515621000	-2.659887000	-0.000511000
6	1.387109000	1.584980000	-0.000391000
1	1.516352000	2.659810000	-0.000488000
1	3.469881000	-1.149942000	0.000364000
22	0.179156000	0.000141000	0.000311000
6	-2.123812000	1.022749000	-0.000193000
6	-2.130815000	0.000084000	1.023974000
1	-2.270497000	0.000172000	2.094455000
1	-2.219193000	2.097716000	-0.000310000
1	-2.269524000	-0.000192000	-2.094727000
6	-2.130359000	-0.000094000	-1.024178000
1	-2.219046000	-2.097730000	0.000054000
6	-2.123749000	-1.022756000	-0.000015000

Frequency (cm^{-1})	Intensity (km/mol)	Frequency (cm^{-1})	Intensity (km/mol)
38.321	9.1785	985.3144	0.141
42.9545	0.3092	1015.5353	0
56.3218	0	1017.9639	0
68.2026	5.3274	1089.3844	6.8602
174.4475	0.2094	1118.8287	4.6245
187.1511	1.634	1214.5859	0
210.3999	0.6994	1256.4056	0.3206
236.884	0	1303.3907	25.4595
310.5157	16.5009	1309.1211	0
466.1732	2.2264	1317.1782	30.4889
471.2587	6.1344	1318.4748	29.4059
622.654	85.357	1346.5517	4.1984
646.0481	93.5948	1433.3052	8.1842
666.0507	64.6703	1574.0702	0.9643
739.4897	18.1833	3110.6685	0.4407
747.8571	7.7558	3125.811	1.1948
759.4653	34.5958	3187.685	4.9648
824.9257	0.3972	3187.9684	4.4292
832.4075	3.3885	3231.8821	0.4194
840.7289	0	3242.0433	27.608
908.0811	6.1058	3246.0825	30.27
916.7064	2.466	3260.0165	9.7452
948.9696	1.1261		

Table S67. Cartesian coordinates for the optimized geometry of isomer 4h-quartet $Ti^{+}(C_2H_2)_4$ followed by its predicted frequencies (cm^{-1}) and IR intensities (km/mol).

Z	x	y	z
22	0.095129000	-0.428278000	-0.046301000
6	1.828222000	-0.789306000	-1.165034000
6	2.433384000	0.367085000	-0.683084000
1	2.971592000	1.056471000	-1.332075000
1	2.160989000	-1.374654000	-2.015386000
6	2.274166000	0.664311000	0.690699000
6	1.436947000	-0.158851000	1.446903000
1	1.468361000	-0.178293000	2.531779000
1	2.762792000	1.541227000	1.110093000
1	-0.764790000	2.425950000	-0.303300000
6	-1.053183000	1.384470000	-0.174141000
6	-2.409750000	1.098914000	-0.241172000
1	-3.158922000	1.862953000	-0.434795000
6	-2.833733000	-0.246938000	-0.059940000
1	-3.878666000	-0.501776000	-0.230664000
6	-1.905936000	-1.198104000	0.343244000
1	-2.274893000	-2.139258000	0.748113000

Frequency (cm^{-1})	Intensity (km/mol)	Frequency (cm^{-1})	Intensity (km/mol)
34.7896	0.973	978.6878	17.9859
84.036	0.3573	1021.019	6.2573
96.007	0.3962	1047.7629	6.4883
123.0394	13.9111	1090.4054	5.6974
182.5603	1.5032	1103.7224	11.9405
193.3213	18.024	1132.9956	5.9557
242.111	8.5803	1143.397	54.7043
286.9739	6.1127	1205.7901	19.3927
344.8238	26.789	1231.0121	31.8992
432.4376	3.0961	1316.4132	4.0444
442.6314	5.1189	1422.9067	41.75
519.4877	7.2399	1442.1153	13.2081
535.7216	24.8569	1472.7938	15.6724
607.4105	59.1057	1482.3763	51.3909
628.2878	6.6418	3113.4499	0.2237
658.3218	41.9854	3114.6017	0.754
693.8846	14.0505	3126.4309	1.9663
732.9533	23.7072	3130.7751	0.3297
743.467	13.5555	3133.1185	1.0458
872.484	52.6396	3146.7553	1.016
914.5523	61.3703	3166.1406	5.0926
916.166	46.2377	3175.7951	19.3676
974.343	4.7608		

Table S68. Cartesian coordinates for the optimized geometry of isomer 4i-quartet $Ti^{+}(C_2H_2)_4$ followed by its predicted frequencies (cm^{-1}) and IR intensities (km/mol).

Z	x	y	z
22	-0.443198000	0.068536000	0.154452000
6	1.324350000	0.712975000	1.208470000
6	2.439531000	0.125740000	0.611626000
1	3.391947000	0.043010000	1.132583000
1	1.480109000	1.374169000	2.059857000
6	2.318279000	-0.346899000	-0.714126000
6	1.044481000	-0.374147000	-1.281505000
1	0.984990000	-0.698330000	-2.320773000
1	3.203783000	-0.688608000	-1.243207000
6	-1.062760000	2.187007000	-0.353010000
6	-2.065113000	1.474167000	-0.444072000
1	-3.082503000	1.192277000	-0.644192000
1	-0.402726000	3.031983000	-0.370771000
1	-0.538954000	-2.828142000	-0.897102000
6	-1.046894000	-2.231482000	-0.169813000
6	-1.752117000	-1.762200000	0.709372000
1	-2.484833000	-1.645127000	1.484009000

Frequency (cm^{-1})	Intensity (km/mol)	Frequency (cm^{-1})	Intensity (km/mol)
32.2471	3.2419	723.9124	27.9511
49.7211	0.4741	747.2883	54.97
79.1196	0.8944	751.156	97.8523
88.016	1.2436	901.0431	34.0872
105.1788	0.5367	964.2151	22.6596
113.5058	0.0402	1021.7154	1.6125
165.5981	7.7931	1093.8086	8.2453
236.8855	8.8286	1128.3677	6.6902
278.8062	3.4344	1188.7447	31.7153
318.1567	26.1264	1291.7884	11.2878
337.3708	2.4682	1456.5097	19.8726
351.2603	2.9027	1463.7212	68.0474
389.1947	6.446	1853.7763	89.1048
446.6529	0.5688	1915.5584	67.1079
506.3319	2.0355	3104.7716	1.9102
619.513	9.4954	3113.6352	0.8826
622.9269	11.5165	3129.649	0.17
646.768	30.3788	3148.7967	2.3256
659.4888	3.856	3281.6006	92.0539
679.1041	9.0222	3314.4827	124.7545
684.9499	19.1213	3361.4531	76.7337
703.3857	7.1213	3407.7969	81.8027
708.5669	31.4919		

Table S69. Cartesian coordinates for the optimized geometry of isomer 4j-quartet $Ti^{+}(C_2H_2)_4$ followed by its predicted frequencies (cm^{-1}) and IR intensities (km/mol).

Z	x	y	z
6	-1.712168000	1.038675000	-0.071490000
6	-1.854560000	-0.067719000	-0.999099000
6	-1.712174000	-1.038637000	0.071497000
6	-1.854540000	0.067761000	0.999109000
1	-1.936934000	2.089234000	-0.146894000
1	-2.100626000	-0.139551000	-2.048814000
1	-1.936980000	-2.089187000	0.146906000
1	-2.100584000	0.139595000	2.048829000
22	0.273230000	0.000008000	-0.000016000
6	1.110401000	2.210098000	0.138073000
6	2.049922000	1.539783000	-0.258865000
1	3.006631000	1.215752000	-0.611989000
1	0.464324000	2.999928000	0.455157000
1	3.006579000	-1.215836000	0.611980000
6	2.049862000	-1.539847000	0.258857000
6	1.110313000	-2.210144000	-0.138039000
1	0.464179000	-2.999944000	-0.455080000

Frequency (cm^{-1})	Intensity (km/mol)	Frequency (cm^{-1})	Intensity (km/mol)
61.2261	0.0966	757.0416	0.0453
78.6297	0.1947	758.0636	47.6902
95.8411	0.665	771.7562	39.635
96.3328	1.3672	864.8265	1.3945
123.2228	0.0291	903.369	2.5945
126.7101	2.0101	951.9166	23.6291
187.8157	21.2365	956.3951	12.0442
259.6866	3.615	1065.4216	0.2441
280.9885	7.5052	1192.9603	0.0087
336.8153	11.934	1245.8977	1.059
340.2638	17.2572	1291.3875	23.2891
357.4909	0.1959	1356.6136	1.8743
404.826	1.2141	1913.2883	82.4376
564.4376	17.424	1933.936	38.7845
618.657	12.9264	3219.1332	0.5037
636.5974	2.6106	3224.4941	7.2821
644.2273	2.62	3254.2785	5.0706
644.3835	3.9996	3260.3349	6.4652
657.1071	2.4533	3335.3319	71.2785
660.0721	1.8328	3336.3451	127.3377
714.8185	28.7213	3418.1445	123.9727
737.2421	1.6788	3420.3464	34.5293
751.6077	84.5541		

Table S70. Cartesian coordinates for the optimized geometry of isomer 4k-quartet $Ti^{+}(C_2H_2)_4$ followed by its predicted frequencies (cm^{-1}) and IR intensities (km/mol).

Z	x	y	z
6	-2.448732000	0.028820000	0.000023000
6	-2.197683000	-1.154011000	0.000024000
1	-2.169398000	-2.220122000	0.000066000
1	-2.834313000	1.024222000	-0.000019000
22	0.057718000	0.009319000	-0.000001000
6	1.053796000	1.124869000	-1.689780000
6	-0.157314000	1.239742000	-1.887450000
1	-1.073247000	1.512869000	-2.375117000
1	2.112169000	1.215883000	-1.839822000
6	-0.157322000	1.239914000	1.887341000
6	1.053789000	1.124998000	1.689698000
1	2.112160000	1.215990000	1.839764000
1	-1.073253000	1.513131000	2.374962000
6	0.804012000	-2.171301000	0.000082000
6	1.814476000	-1.470779000	0.000066000
1	2.842460000	-1.167611000	0.000063000
1	0.223486000	-3.072900000	0.000110000

Frequency (cm^{-1})	Intensity (km/mol)	Frequency (cm^{-1})	Intensity (km/mol)
50.2228	0.0001	669.5355	4.1066
59.9914	0.4382	676.3258	5.1836
81.5812	0.0191	676.7121	12.7245
98.6604	0.1736	697.443	0.8281
99.1477	0.3863	704.3885	0.2467
123.4327	0.2157	724.5356	118.3541
131.0327	0.5348	726.3317	70.5292
131.7609	1.2118	739.8439	9.3333
154.9828	0.0214	768.3346	95.7108
196.0827	0.3262	790.768	5.046
256.9343	9.8832	1853.7176	98.2018
293.9952	1.2503	1860.2281	97.4035
337.8426	4.1628	1882.5326	14.8254
348.1475	3.3007	1992.7388	5.9149
357.4289	2.0333	3286.7915	5.1081
362.9953	1.8103	3287.1513	125.1277
401.9904	19.978	3295.8746	67.5822
622.9536	1.5743	3359.5818	76.957
624.1469	1.4151	3360.9445	28.069
630.6929	0.2075	3368.3996	94.8075
641.8409	0.6559	3371.6698	82.6692
658.427	5.3825	3459.5167	43.2579
668.4423	80.3174		

Table S71. n = 5 isomers and energies.

Isomer	2s + 1	E (hartree)	Relative E (kcal/mol)
5a	2	-1236.259357	0.0
5b	2	-1236.249364	+6.3
5c	2	-1236.244657	+9.2
5d	2	-1236.235737	+14.8
5e	2	-1236.219334	+25.1
5f	2	-1236.215203	+27.7
5g	2	-1236.191043	+42.9
5h	2	-1236.150323	+68.4
5i	2	-1236.123232	+85.4
5j	2	-1236.123115	+85.5
5k	2	-1236.121304	+86.6
5l	2	-1236.11347	+91.5
5m	2	-1236.1133	+91.7
5n	2	-1236.112443	+92.2
5o	2	-1236.095408	+102.9
5p	2	-1236.058559	+126.0
5q	2	-1236.058106	+126.3
5r	2	-1236.057989	+126.4
5s	2	-1236.057646	+126.6
5t	2	-1236.049014	+132.0
5a	4	-1236.208715	+31.8
5b	4	-1236.226575	+20.6
5d	4	-1236.211796	+29.8
5e	4	-1236.197762	+38.7
5f	4	-1236.174225	+53.4
5g	4	-1236.151384	+67.8
5h	4	-1236.149883	+68.7
5i	4	-1236.083692	+110.2
5j	4	-1236.078005	+113.8
5k	4	-1236.063417	+123.0
5l	4	-1236.046198	+133.8
5m	4	-1236.062509	+123.5
5n	4	-1236.069175	+119.3
5o	4	-1236.156579	+64.5
5p	4	-1236.024812	+147.2
5q	4	-1235.994826	+166.0
5r	4	-1235.994563	+166.2
5s	4	-1235.994827	+166.0
5t	4	-1235.993432	+166.9
5u	4	-1236.153765	+66.3
5v	4	-1236.062994	+123.2
5w	4	-1236.059418	+125.5
5x	4	-1236.051622	+130.4

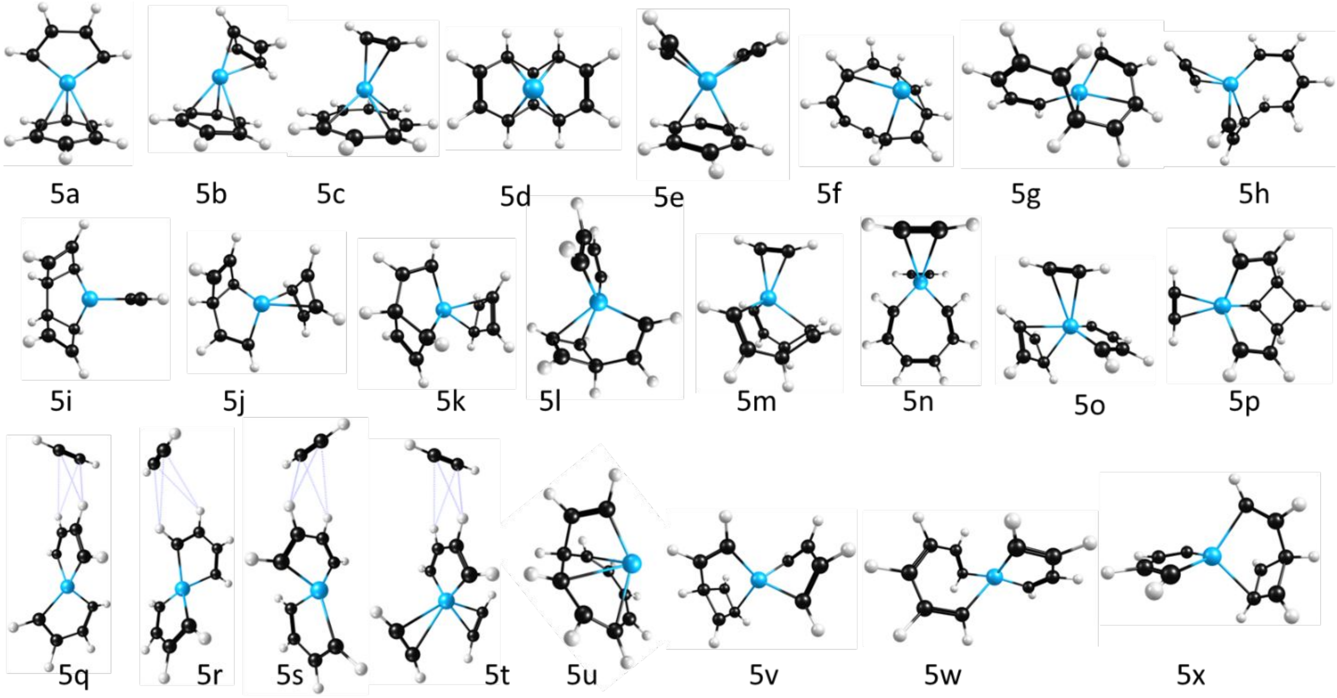

Figure S58. Isomers of $Ti^+(C_2H_2)_5$.

Table S72. Cartesian coordinates for the optimized geometry of isomer 5a-doublet $Ti^+(C_2H_2)_5$ followed by its predicted frequencies (cm^{-1}) and IR intensities (km/mol).

Z	x	y	z
22	-0.423424000	0.000001000	0.097217000
6	-1.606910000	1.588933000	-0.133027000
6	-2.656957000	0.757703000	0.063766000
1	-3.660562000	1.146832000	0.217112000
1	-1.748200000	2.660094000	-0.192138000
6	-2.656957000	-0.757702000	0.063769000
6	-1.606911000	-1.588931000	-0.133028000
1	-1.748197000	-2.660092000	-0.192136000
1	-3.660562000	-1.146831000	0.217119000
6	1.751393000	1.211242000	0.664735000
6	1.780082000	0.000000000	1.370313000
6	1.659473000	1.212108000	-0.730896000
1	1.848505000	0.000001000	2.449665000
1	1.614897000	2.149412000	-1.267917000
6	1.751389000	-1.211242000	0.664736000
6	1.591610000	-0.000002000	-1.432412000
1	1.776073000	-2.149418000	1.202800000
1	1.508312000	-0.000002000	-2.510518000
6	1.659470000	-1.212111000	-0.730895000
1	1.614886000	-2.149415000	-1.267914000
1	1.776078000	2.149417000	1.202800000

Frequency	Intensity	Frequency	Intensity	Frequency	Intensity
22.2697	0.081	824.3598	8.0199	1340.7499	3.9908
64.0458	0.4628	833.0737	0.5954	1341.8041	0.0156
91.9924	9.134	920.4559	0.0965	1389.7675	0.0005
96.5784	1.4315	928.0482	1.0694	1419.4698	16.2133
177.4016	4.7871	984.7702	11.9883	1506.2063	25.6837
202.67	1.9413	999.1658	0.0439	1507.6389	20.5797
215.9017	0.6772	1008.3764	3.0527	1565.1483	1.8741
239.8171	0.0094	1016.6054	2.6497	1592.9956	0.0143
340.9602	16.921	1029.9006	0.3687	1600.3889	6.5237
408.9533	0.0098	1031.4043	0.0568	3113.1186	0.1907
416.2427	0.2486	1047.8509	0.7335	3128.3844	0.3635
481.8528	3.9656	1052.376	0.3882	3195.3917	1.2895
611.7899	0.019	1052.5316	1.1772	3196.0359	1.5823
618.3717	1.4234	1088.8138	12.0337	3196.7884	0.0191
636.8288	68.0589	1113.9861	3.5291	3202.5604	0.7051
657.9075	28.8108	1192.7799	0.0231	3204.6133	0.4339
698.4598	0.5945	1196.7614	0.0116	3212.6204	7.7862
756.4562	22.1648	1204.9504	0.5664	3214.5017	6.4836
767.4571	86.1484	1295.3618	28.1742	3219.8621	1.942

Table S73. Cartesian coordinates for the optimized geometry of isomer 5b-doublet $Ti^{+}(C_2H_2)_5$ followed by its predicted frequencies (cm^{-1}) and IR intensities (km/mol).

Z	x	y	z
6	-1.408000000	0.865560000	1.220051000
6	-1.845762000	-0.462033000	1.217736000
6	-1.155463000	1.521011000	0.000139000
1	-2.027874000	-0.971902000	2.155153000
1	-0.825671000	2.549358000	0.000236000
6	-2.049367000	-1.140763000	-0.000110000
6	-1.407990000	0.865786000	-1.219895000
1	-2.403040000	-2.163235000	-0.000206000
1	-1.252635000	1.385189000	-2.155352000
6	-1.845751000	-0.461808000	-1.217829000
1	-2.027852000	-0.971505000	-2.155342000
1	-1.252653000	1.384791000	2.155605000
22	0.313910000	-0.501710000	-0.000041000
6	2.411447000	-0.907539000	-0.000080000
6	2.165622000	0.098581000	1.027889000
1	2.279585000	0.133792000	2.099702000
1	2.834490000	-1.901676000	-0.000166000
1	2.279570000	0.134156000	-2.099678000
6	2.165635000	0.098764000	-1.027868000
1	1.733679000	2.136341000	0.000187000
6	1.929027000	1.076159000	0.000094000

Frequency	Intensity	Frequency	Intensity	Frequency	Intensity
21.4434	1.0146	830.1142	26.3929	1250.6417	3.8498
66.5524	2.5842	906.4192	0.25	1336.8494	0.2437
124.5243	5.5093	910.7651	0.1053	1339.526	3.2933
198.6518	7.4266	916.9295	16.5048	1346.5826	0.4773
235.0684	0.3042	935.5756	19.0958	1383.4764	0.0031
237.6044	0.2066	954.8428	1.4206	1493.5667	16.3024
305.9715	10.9893	964.949	9.0241	1501.3605	17.3407
308.0238	10.6985	988.3587	4.5167	1556.2715	0.263
402.4699	0.1595	996.1526	1.7482	1583.6707	8.7835
419.8276	1.6483	1009.4329	0.3062	3189.6037	0.1972
466.5147	8.9832	1019.0962	0.146	3194.1584	1.3143
612.7273	0.3248	1034.0806	2.695	3204.0936	5.0935
613.936	0.0836	1044.7809	1.2228	3210.0877	1.6966
626.6342	16.8244	1048.9057	1.8485	3212.2603	4.3047
656.7165	4.3953	1166.7564	2.0437	3212.3052	5.879
660.6473	12.3215	1183.8993	0.0037	3225.4174	2.1365
687.7349	2.3718	1185.7555	0.1198	3234.0246	4.4135
763.8382	67.2633	1194.2976	1.4778	3239.0561	6.0782
787.1248	37.0402	1195.0775	0.3284	3256.2936	3.7954

Table S74. Cartesian coordinates for the optimized geometry of isomer 5c-doublet $Ti^+(C_2H_2)_5$ followed by its predicted frequencies (cm^{-1}) and IR intensities (km/mol).

Z	x	y	z
6	-0.345288000	-0.987032000	1.701182000
6	0.233361000	-1.819549000	0.722453000
6	0.247936000	-1.831846000	-0.684338000
6	-1.775192000	0.919452000	-0.728617000
6	-1.143010000	0.123408000	-1.706985000
6	-0.308928000	-1.015629000	-1.688845000
1	-1.230457000	0.548542000	-2.698703000
1	0.030603000	-1.198453000	2.694284000
1	0.935022000	-2.525693000	1.148865000
1	0.957756000	-2.545648000	-1.083665000
1	-2.240707000	1.799278000	-1.154256000
1	0.087690000	-1.243749000	-2.670130000
22	0.348438000	0.365225000	0.002466000
1	-1.291326000	0.590988000	2.665826000
6	-1.181282000	0.150672000	1.683026000
6	-1.791802000	0.930391000	0.678374000
1	-2.267410000	1.816440000	1.079223000
6	2.617253000	0.555632000	0.003812000
6	2.114750000	1.680576000	0.011209000
1	2.041584000	2.753746000	0.018310000
1	3.304819000	-0.266862000	-0.001637000

Frequency	Intensity	Frequency	Intensity	Frequency	Intensity
5.7777	0.0874	765.9368	97.632	1433.4932	0.0049
59.3788	2.0796	767.546	3.5996	1482.0879	1.8104
95.5798	0.1167	769.0006	0.0182	1482.6588	1.4334
230.765	3.0074	781.24	25.1654	1508.9737	0.197
235.111	0.0231	827.2441	1.5729	1524.1312	0.0061
239.3713	5.2743	828.0311	5.2158	1527.3062	0.0132
311.9528	1.1028	912.3379	0.1545	1544.2825	0.0737
348.2401	2.9739	913.1837	0.0233	1545.3702	0.0548
350.9444	4.0078	924.4671	7.5696	1857.5265	96.1451
385.6888	0.0067	932.168	6.9842	3159.8915	0.0214
389.1039	1.1572	967.6166	0	3165.4798	0.0379
418.0731	3.6899	976.2504	0.1154	3165.4923	0.0676
483.8874	0.0035	981.1395	0.1355	3175.0722	0.0539
501.5296	0.0086	1009.1985	0.0008	3175.2446	0.0009
589.0269	0.0081	1216.6081	0.0488	3184.5224	2.8219
643.0373	44.1831	1217.6041	0.0287	3186.6625	5.7551
667.915	14.1542	1355.6319	0.0006	3191.4502	1.7058
678.1695	11.8836	1360.2783	0.0083	3275.8563	114.9509
731.9335	59.3554	1364.344	0	3365.1989	98.3937

Table S75. Cartesian coordinates for the optimized geometry of isomer 5d-doublet $Ti^{+}(C_2H_2)_5$ followed by its predicted frequencies (cm^{-1}) and IR intensities (km/mol).

Z	x	y	z
1	-1.173272000	-2.515959000	0.386193000
6	-1.148773000	-1.436031000	0.380301000
6	-2.154902000	-0.710392000	-0.226144000
6	-2.154903000	0.710390000	-0.226141000
6	-1.148775000	1.436029000	0.380305000
6	0.000027000	0.807062000	1.176166000
6	0.000027000	-0.807065000	1.176165000
6	1.148833000	-1.436028000	0.380325000
6	2.154895000	-0.710399000	-0.226203000
6	2.154895000	0.710399000	-0.226203000
6	1.148832000	1.436027000	0.380325000
1	-2.917782000	-1.234201000	-0.788374000
1	-2.917784000	1.234200000	-0.788369000
1	-1.173274000	2.515957000	0.386199000
1	1.173326000	2.515956000	0.386207000
1	2.917753000	1.234205000	-0.788466000
1	2.917753000	-1.234205000	-0.788466000
1	1.173327000	-2.515957000	0.386204000
1	0.000015000	1.110245000	2.220946000
1	0.000012000	-1.110249000	2.220945000
22	-0.000047000	0.000003000	-0.938473000

Frequency	Intensity	Frequency	Intensity	Frequency	Intensity
206.9581	27.2122	886.4082	3.9848	1272.3415	14.2719
217.0625	0	894.3285	0	1368.9927	0
245.5318	0.022	922.5401	6.8392	1386.2494	2.2901
267.4563	3.4881	926.7468	11.6896	1416.7346	0.1017
298.8951	4.9906	933.7694	3.3588	1462.2382	2.6306
361.619	0.0106	955.5121	0	1490.6544	79.7776
364.6228	4.4979	976.5672	4.0795	1532.185	2.9359
475.2927	0	987.1118	0	1562.194	1.3608
488.8825	0.9856	999.6322	0.5365	1562.6022	0.0045
526.238	1.1382	1022.0621	2.3516	3083.791	1.2418
563.739	0	1041.9876	0.2983	3099.3922	2.2055
563.7679	3.0103	1085.5991	0.6702	3179.1512	0
583.22	0.3094	1122.4717	43.8714	3179.8502	0.0551
640.6596	1.5689	1166.9767	3.3575	3189.1191	0.2356
718.9995	53.7879	1174.7662	0	3190.1413	0.2568
777.9959	9.904	1192.6304	0	3207.1585	0
822.1985	0.7725	1200.719	12.6155	3208.4353	4.6437
823.5215	0	1203.5574	4.5343	3213.5124	2.1337
827.8106	76.7029	1242.8725	0.7227	3214.4298	0.23

Table S76. Cartesian coordinates for the optimized geometry of isomer 5e-doublet $Ti^{+}(C_2H_2)_5$ followed by its predicted frequencies (cm^{-1}) and IR intensities (km/mol).

Z	x	y	z
6	-1.563514000	-1.039000000	0.871427000
6	-1.656388000	-1.211197000	-0.519387000
6	-1.515086000	0.250072000	1.414510000
1	-1.709891000	-2.206630000	-0.937687000
1	-1.468807000	0.382112000	2.487305000
6	-1.660199000	-0.096489000	-1.360303000
6	-1.507008000	1.363216000	0.569593000
1	-1.721993000	-0.230511000	-2.432691000
1	-1.451957000	2.359465000	0.987466000
6	-1.566814000	1.193337000	-0.819397000
1	-1.562789000	2.056104000	-1.471711000
1	-1.554844000	-1.902168000	1.521544000
22	0.632867000	-0.106870000	-0.130285000
6	1.767327000	-1.669221000	-0.590694000
6	1.578014000	-1.642233000	0.706406000
1	1.867767000	-2.235197000	1.564425000
1	2.303172000	-2.303089000	-1.286155000
1	2.356332000	1.880722000	-1.556029000
6	2.045035000	1.658071000	-0.554721000
1	1.998105000	1.837440000	1.712498000
6	1.915604000	1.645592000	0.662117000

Frequency	Intensity	Frequency	Intensity	Frequency	Intensity
7.1942	0.2784	661.3248	6.3842	1203.0294	1.2975
66.142	0.9886	700.441	12.3263	1337.4352	3.5766
107.7679	0.6352	705.3195	38.2078	1389.6936	0.0025
128.8183	0.3161	729.2952	111.2756	1505.9726	21.0503
134.6528	0.2813	757.9946	66.3653	1510.1804	20.0478
154.5303	1.2828	791.1727	31.1343	1535.937	24.0555
166.6693	1.2868	909.7037	0.0812	1597.4415	0.6781
184.9601	0.5413	916.7825	0.5983	1606.5604	4.9096
208.3391	1.5376	920.4361	0.5011	1900.1123	67.4388
273.1104	6.7786	1000.0764	0.3269	3172.4992	1.45
343.5358	4.2579	1016.5304	5.9635	3191.7561	0.3561
410.1931	0.5348	1027.6469	5.9985	3197.1846	0.0875
414.2813	0.1661	1028.5189	25.4903	3202.8799	4.516
551.038	4.4009	1030.5179	16.4514	3203.8019	1.2875
584.1479	7.439	1047.107	0.4293	3211.771	4.019
615.8568	0.551	1048.7813	1.868	3211.9109	3.1975
616.5733	0.0279	1056.7202	1.9609	3221.5968	3.0346
625.6093	21.769	1190.407	0.3199	3310.6594	115.9031
654.8472	18.2984	1200.282	0.0308	3391.3131	79.3971

Table S77. Cartesian coordinates for the optimized geometry of isomer 5f-doublet $Ti^{+}(C_2H_2)_5$ followed by its predicted frequencies (cm^{-1}) and IR intensities (km/mol).

Z	x	y	z
6	1.407547000	1.543519000	-0.358249000
6	2.064103000	0.587929000	0.438903000
6	1.635587000	-0.619743000	1.072825000
6	0.548144000	-1.542550000	1.024357000
6	-0.637709000	-1.779827000	0.265319000
6	-1.714885000	-1.081101000	-0.397100000
6	-2.389293000	0.097281000	-0.160002000
6	-2.027204000	1.080900000	0.809518000
6	-0.808498000	1.644351000	0.790174000
6	0.007388000	1.645420000	-0.428690000
1	-0.414073000	2.199409000	-1.263981000
1	-0.449224000	2.228669000	1.632747000
1	-2.770010000	1.373480000	1.545988000
1	-3.355484000	0.184684000	-0.650531000
1	-2.217193000	-1.742752000	-1.097455000
1	-0.783130000	-2.850761000	0.162593000
1	0.882924000	-2.481894000	1.449067000
1	2.485384000	-1.111377000	1.530519000
1	3.144747000	0.671676000	0.450538000
1	2.041269000	2.124306000	-1.022282000
22	0.587441000	-0.456933000	-0.958161000

Frequency	Intensity	Frequency	Intensity	Frequency	Intensity
115.7018	0.2766	841.0646	11.6969	1419.631	2.7248
150.656	1.3305	848.5798	12.0722	1438.8288	7.7932
178.803	1.9993	860.2924	6.5103	1453.7366	1.7083
234.8979	2.3205	900.9185	0.9441	1476.7077	4.024
295.517	3.5154	918.0342	2.379	1494.0075	1.9376
309.4287	3.4193	938.8287	3.0159	1499.0994	0.3252
337.7183	2.0576	956.9603	0.9746	1530.2215	0.1559
343.568	11.0883	984.076	0.0885	1560.4232	5.9524
393.954	1.8244	995.3262	6.4166	1641.8718	3.7592
427.3535	0.6656	999.6975	0.1616	3119.4274	0.3963
438.9369	4.5745	1018.376	0.8034	3121.0064	0.7159
494.5337	3.9155	1059.6702	0.6037	3132.1106	0.3187
548.7563	1.6564	1124.117	1.3701	3138.9552	1.9896
635.5676	2.9753	1190.9563	6.8896	3141.5266	0.1748
688.3863	7.0877	1213.4663	2.6258	3144.7366	1.2668
734.7231	11.3511	1261.9208	1.7083	3149	0.0267
744.1739	42.6231	1327.9197	1.6754	3158.3453	0.5822
769.5883	89.7121	1361.1455	1.6631	3161.3822	0.5699
801.3886	20.4575	1407.7209	6.8893	3173.4917	0.5619

Table S78. Cartesian coordinates for the optimized geometry of isomer 5g-doublet $Ti^+(C_2H_2)_5$ followed by its predicted frequencies (cm^{-1}) and IR intensities (km/mol).

Z	x	y	z
6	2.683188000	-0.462716000	-0.392681000
6	2.632683000	0.751585000	0.351883000
6	1.511128000	1.490760000	0.634980000
6	-2.427233000	-0.202192000	0.533331000
6	-2.411004000	0.262722000	-0.849862000
6	-1.299926000	0.611002000	-1.587979000
1	-3.402485000	0.463296000	-1.251706000
1	3.667229000	-0.738138000	-0.757780000
1	3.607919000	1.134103000	0.647412000
1	1.679463000	2.393836000	1.222953000
1	-3.354917000	0.011177000	1.049395000
1	-1.379169000	0.922826000	-2.622142000
22	-0.349107000	0.737628000	0.084077000
1	1.889666000	-1.878475000	-1.719838000
6	1.641621000	-1.290664000	-0.839939000
6	-1.462962000	-0.866351000	1.311281000
1	-1.710584000	-0.950660000	2.363024000
1	-0.329078000	-2.012600000	-1.040100000
6	0.336278000	-1.475786000	-0.375610000
6	-0.130400000	-1.193164000	0.961442000
1	0.572082000	-1.324368000	1.778018000

Frequency	Intensity	Frequency	Intensity	Frequency	Intensity
91.7895	2.7575	791.7324	5.2401	1317.6337	16.8798
96.6606	0.9621	809.0364	20.7176	1361.5926	13.9471
116.0937	1.8519	818.9607	10.1124	1391.6925	2.8856
200.9822	1.612	873.0114	4.6693	1414.0836	14.6168
214.764	1.8178	897.886	1.0075	1451.1641	4.7361
242.1918	2.8095	915.311	1.327	1473.6412	6.4407
295.0184	2.0973	938.2412	3.3855	1506.7996	1.893
339.4695	0.4098	960.8659	2.1365	1514.7422	4.0705
370.4244	4.6078	978.1088	13.703	1527.6391	9.3912
408.5127	1.7665	998.4421	2.8177	3091.4135	2.5225
428.799	8.408	1006.8427	0.4025	3114.2214	1.2077
477.3032	3.6209	1010.7959	1.3341	3119.2856	1.9446
498.5579	5.4981	1076.3029	6.191	3137.6675	0.5481
524.5609	53.9298	1118.6349	9.7127	3147.4577	3.1009
596.7203	8.807	1170.5999	4.5676	3164.5804	0.2236
629.1199	55.0025	1207.5589	2.7005	3170.6203	1.6592
695.9896	9.4732	1250.9189	5.2489	3174.1872	1.7677
716.4731	40.5396	1270.5606	19.9737	3184.1483	3.0393
724.9171	33.5672	1283.526	4.6839	3192.2743	11.3989

Table S79. Cartesian coordinates for the optimized geometry of isomer 5h-doublet $Ti^+(C_2H_2)_5$ followed by its predicted frequencies (cm^{-1}) and IR intensities (km/mol).

Z	x	y	z
6	0.096602000	1.650504000	-0.967090000
6	0.571693000	2.172540000	0.319638000
6	0.589659000	1.342575000	1.391598000
6	-2.699215000	-0.352913000	0.329626000
6	-2.110533000	-1.646486000	0.082182000
6	-0.781306000	-1.793617000	-0.153081000
1	-2.773336000	-2.509475000	0.113598000
1	0.642561000	1.908564000	-1.869361000
1	0.985270000	3.180881000	0.332567000
1	0.950541000	1.688169000	2.354941000
1	-3.617552000	-0.295280000	0.902119000
1	-0.444558000	-2.771299000	-0.508535000
22	0.646833000	-0.337107000	0.242229000
1	-1.219849000	0.369920000	-2.023417000
6	-1.024517000	0.859795000	-1.072467000
6	-2.103787000	0.784460000	-0.086977000
1	-2.449731000	1.738306000	0.299647000
6	2.691290000	-0.683440000	-0.806084000
6	2.733152000	-1.292131000	0.252603000
1	3.020556000	-1.897980000	1.090405000
1	2.897535000	-0.243169000	-1.760673000

Frequency	Intensity	Frequency	Intensity	Frequency	Intensity
55.682	0.2659	654.6737	65.7015	1293.3023	22.6884
73.5501	0.4346	703.5381	49.9476	1324.1724	27.5626
96.4311	1.0983	728.2453	44.8071	1386.2698	10.41
105.3166	1.9134	738.884	50.6255	1445.8725	3.5379
131.9994	3.0818	761.1419	48.4187	1484.9392	8.2344
167.2785	2.6489	805.7447	21.3147	1520.578	64.0234
203.8236	9.0924	817.2152	11.0928	1553.8428	37.6295
256.2119	3.4325	854.5113	4.098	1621.3514	7.0013
281.819	8.6076	927.0894	2.0225	1912.7659	71.5285
288.2982	6.5641	963.2063	17.7779	3066.4396	1.4625
308.5132	5.9887	982.1201	1.6758	3096.8947	1.8029
338.6576	1.8595	995.7282	3.1525	3123.3413	1.6687
429.4399	9.5335	1004.4977	9.3835	3132.3561	1.2099
454.6223	4.4174	1013.7713	16.0605	3152.7809	1.8371
489.2386	26.2117	1050.5891	2.7218	3153.4693	1.3096
530.1403	18.258	1091.0724	11.5813	3162.0508	2.3047
598.0324	27.0916	1134.6508	0.8548	3183.4879	0.0554
642.2792	7.4075	1203.0485	1.4723	3305.0941	142.4933
650.2467	1.5327	1239.1075	1.8224	3390.2574	113.7445

Table S80. Cartesian coordinates for the optimized geometry of isomer 5i-doublet $Ti^{+}(C_2H_2)_5$ followed by its predicted frequencies (cm^{-1}) and IR intensities (km/mol).

Z	x	y	z
6	-0.283741000	-1.551536000	0.982159000
6	-1.556726000	-0.784367000	0.451295000
6	-1.209752000	-1.483117000	-0.869875000
6	-0.303246000	-2.313273000	-0.231010000
1	-0.017986000	-1.828577000	1.992185000
1	-2.448166000	-1.190302000	0.921784000
1	-1.688471000	-1.496472000	-1.837534000
1	0.235491000	-3.197829000	-0.546730000
22	0.494318000	0.000293000	-0.236921000
6	-1.557582000	0.782705000	0.451307000
1	-2.449459000	1.187659000	0.921815000
6	-0.285430000	1.551258000	0.982171000
6	-1.211387000	1.481853000	-0.869855000
1	-1.690125000	1.494692000	-1.837511000
1	-0.019969000	1.828579000	1.992197000
6	-0.305776000	2.312986000	-0.230992000
1	0.231996000	3.198130000	-0.546709000
6	2.555052000	0.001242000	0.622704000
6	2.653402000	0.001365000	-0.614050000
1	3.181355000	0.001652000	-1.553119000
1	2.821465000	0.001325000	1.662744000

Frequency	Intensity	Frequency	Intensity	Frequency	Intensity
68.4854	0.3657	729.7217	55.1299	1195.5289	2.5022
78.7353	0.2372	737.3628	11.407	1200.1667	0.3517
96.404	0.1506	774.1217	0.6322	1207.4214	5.5391
104.8875	1.4093	792.855	18.8629	1293.5142	0.5507
170.2895	0.9491	810.8918	22.5026	1352.5781	12.489
246.836	3.2075	878.8917	0.1303	1353.8533	3.5706
311.0516	0.9859	878.9261	15.6043	1457.4193	2.2596
323.7123	3.8795	933.5707	15.1306	1457.6437	0.1042
370.7202	4.9876	935.175	16.0672	1816.3667	99.4952
377.8962	3.1663	954.3218	27.0704	3120.7692	0.445
400.1671	5.3169	960.1032	76.0678	3130.1381	8.3162
420.5068	3.3966	979.1851	14.3627	3194.9114	2.4985
501.7406	7.921	989.6504	12.9452	3195.3682	1.1066
520.9035	7.6989	1006.2867	5.0433	3214.3934	1.7832
646.7333	10.8793	1024.2772	1.3907	3214.7508	4.242
660.1167	88.5355	1025.5134	0.0509	3230.8338	2.2361
670.2904	27.2922	1041.4271	4.8286	3233.1701	5.9416
704.5198	0.0056	1150.4099	4.4312	3251.3029	88.5442
727.1593	13.465	1157.1183	1.2176	3335.9621	72.2992

Table S81. Cartesian coordinates for the optimized geometry of isomer 5j-doublet $Ti^{+}(C_2H_2)_5$ followed by its predicted frequencies (cm^{-1}) and IR intensities (km/mol).

Z	x	y	z
6	-1.262430000	-0.869313000	1.065424000
6	-2.130789000	0.091948000	0.201720000
6	-1.779533000	-0.909491000	-0.930112000
6	-1.291869000	-1.814703000	0.013826000
1	-1.167803000	-0.963834000	2.136044000
1	-3.179105000	0.013443000	0.477390000
1	-2.121094000	-0.992505000	-1.952246000
1	-0.987572000	-2.851427000	-0.050610000
22	0.122565000	0.143721000	-0.297228000
6	-1.750402000	1.590678000	0.067375000
1	-2.603485000	2.256647000	-0.045328000
6	-0.481697000	2.017119000	0.091624000
1	-0.199285000	3.061335000	0.056074000
1	1.739467000	-0.977339000	1.995439000
6	1.808708000	-0.540390000	1.014594000
6	2.226103000	0.748484000	0.521032000
1	2.496186000	1.682666000	0.989086000
6	2.132703000	-1.073057000	-0.298195000
6	2.257812000	0.261893000	-0.848714000
1	2.655820000	0.657124000	-1.770142000
1	2.298800000	-2.066989000	-0.688133000

Frequency	Intensity	Frequency	Intensity	Frequency	Intensity
59.5436	0.1673	756.669	8.3854	1204.6875	4.3962
78.5156	0.6151	780.9679	21.1382	1241.3415	1.9429
132.6041	1.0388	793.9049	2.8911	1254.3194	2.0554
158.3326	1.1129	843.84	18.8645	1292.5346	18.8104
169.293	7.3722	869.5504	4.0578	1296.7732	22.1616
238.9199	6.5123	911.2242	3.7343	1361.2422	1.9651
241.0794	6.9627	918.9764	12.498	1380.1744	4.5598
330.1299	3.0613	930.5057	12.8098	1425.9058	3.403
361.2127	5.9239	956.3411	28.9424	1555.113	3.588
389.865	0.9432	958.8001	25.4906	3113.2976	2.8789
426.0041	18.843	964.171	31.8484	3126.3275	5.2244
463.437	2.5015	966.3394	14.2247	3194.2201	3.8811
563.7362	37.721	993.1894	9.1964	3198.3231	1.9984
596.5312	23.6832	997.504	2.3213	3219.721	7.003
658.1371	6.5715	1015.5493	0.6395	3221.7869	3.0818
675.6648	11.4621	1067.717	0.6167	3235.7376	4.9451
693.3768	2.6109	1080.3752	2.0736	3236.06	6.932
709.8339	14.4153	1172.6208	4.1608	3247.7794	16.4626
737.4313	63.8775	1194.4271	0.0935	3272.2689	8.5726

Table S82. Cartesian coordinates for the optimized geometry of isomer 5k-doublet $Ti^{+}(C_2H_2)_5$ followed by its predicted frequencies (cm^{-1}) and IR intensities (km/mol).

Z	x	y	z
6	-1.959014000	-0.765108000	-0.849473000
6	-1.951728000	-0.951133000	0.612702000
6	-2.407249000	0.426892000	0.690569000
6	-2.135396000	0.646309000	-0.712177000
1	-1.956068000	-1.443761000	-1.689046000
1	-2.128659000	-1.812664000	1.236519000
1	-2.848470000	1.033674000	1.468958000
1	-2.296579000	1.462898000	-1.393850000
22	-0.112602000	0.168771000	0.254739000
1	3.102981000	0.006379000	-0.702222000
6	2.109570000	0.075545000	-0.267129000
6	1.141615000	-0.891240000	-1.057416000
1	1.050060000	-1.031758000	-2.125145000
6	1.272758000	-1.827305000	0.022905000
1	0.960012000	-2.855672000	0.143252000
6	1.883545000	-0.925955000	0.872379000
1	2.241914000	-0.961851000	1.891421000
6	1.767722000	1.583886000	-0.049485000
6	0.513784000	2.056523000	-0.022154000
1	2.651938000	2.207113000	0.069060000
1	0.286479000	3.112191000	0.052480000

Frequency	Intensity	Frequency	Intensity	Frequency	Intensity
62.2527	0.5812	764.647	7.0389	1194.9257	3.2382
70.535	0.0245	772.6165	36.3124	1230.7905	1.6232
113.7169	1.2925	786.8047	31.8125	1250.1519	2.5251
137.1401	7.6806	842.2995	18.9946	1292.3714	6.4799
163.0863	1.4609	867.012	4.032	1295.6688	31.8517
243.2485	1.4482	909.1372	11.6178	1344.3711	6.4803
252.7201	9.2313	916.9582	4.8536	1368.1583	3.873
321.9103	0.9795	935.1778	6.4505	1467.574	1.9729
351.2545	3.2062	949.5868	24.0689	1547.1153	2.4571
398.6117	6.9869	959.33	30.7357	3111.6003	3.5674
439.2578	15.5533	965.2943	12.3949	3126.788	8.2441
471.3739	4.1197	969.8391	9.3695	3193.4792	1.3322
515.9904	21.5889	998.0785	14.9269	3201.9754	2.3856
603.3171	22.2886	1000.8666	6.0271	3212.8256	5.453
664.5732	4.2542	1023.5832	2.2354	3217.6921	4.6926
692.4402	11.5499	1069.9066	0.928	3228.1472	2.9448
714.3471	8.5516	1083.3641	2.5216	3232.2772	5.4625
733.495	16.0745	1165.836	4.308	3246.4453	13.5232
755.9186	24.3866	1194.6715	0.4737	3278.1189	11.5025

Table S83. Cartesian coordinates for the optimized geometry of isomer 5l-doublet $Ti^{+}(C_2H_2)_5$ followed by its predicted frequencies (cm^{-1}) and IR intensities (km/mol).

Z	x	y	z
6	1.667319000	0.960046000	1.023068000
6	2.261998000	-0.034272000	0.000051000
6	1.667447000	0.960324000	-1.022755000
6	1.238996000	1.820690000	0.000245000
1	1.714425000	1.021647000	2.100416000
1	3.350934000	0.010020000	0.000134000
1	1.714646000	1.022198000	-2.100084000
1	0.787724000	2.803748000	0.000348000
22	-0.280125000	-0.179745000	-0.000007000
6	1.847686000	-1.531406000	-0.000213000
1	2.702545000	-2.203683000	-0.000306000
6	0.591415000	-1.982924000	-0.000333000
1	0.342577000	-3.036077000	-0.000526000
6	-2.616188000	0.166975000	-0.748469000
6	-1.522514000	0.049782000	-1.541155000
1	-1.603694000	0.033781000	-2.620445000
1	-3.613136000	0.260343000	-1.174438000
1	-3.613216000	0.259827000	1.174392000
6	-2.616239000	0.166648000	0.748446000
6	-1.522616000	0.049127000	1.541153000
1	-1.603869000	0.032644000	2.620431000

Frequency	Intensity	Frequency	Intensity	Frequency	Intensity
53.1135	0.5324	730.0222	1.9287	1269.5541	0.8234
53.4861	1.0765	756.6468	26.2012	1300.7137	20.9486
83.4978	0.3364	821.0907	0.0171	1318.0895	11.7127
113.3268	2.8351	849.8447	27.0931	1330.279	14.8849
124.8489	0.5647	865.3107	9.8501	1339.7845	42.5747
150.5135	4.3194	910.6721	19.726	1423.818	7.2026
195.9091	0.7949	913.0303	4.0182	1440.8327	74.1829
218.9648	14.7891	958.8519	7.3788	1563.8287	5.8617
274.9631	0.0325	970.1552	24.5629	1569.1561	0.8491
276.9302	3.2236	976.3573	1.5693	3088.3376	4.9068
348.5386	2.7926	979.2242	0.9353	3109.7058	0.3887
353.5221	20.5939	990.6283	1.6118	3121.1485	0.2728
472.8895	5.5533	1014.8029	0.0119	3123.5894	1.7667
544.5596	19.3682	1088.8132	10.1617	3191.695	5.6268
615.7801	87.2801	1094.4136	10.9851	3191.7413	1.4288
659.6744	34.6529	1099.3099	10.1633	3199.1835	9.1418
673.8644	60.9578	1117.4556	2.8918	3210.0852	3.6459
683.908	28.0002	1185.1921	2.3659	3232.2296	14.2109
694.1452	21.6682	1208.7149	0.1115	3240.0883	5.4306

Table S84. Cartesian coordinates for the optimized geometry of isomer 5m-doublet $Ti^{+}(C_2H_2)_5$ followed by its predicted frequencies (cm^{-1}) and IR intensities (km/mol).

Z	x	y	z
6	-1.773070000	0.824987000	0.145241000
6	-1.773058000	-0.824851000	0.145376000
6	-1.154935000	-0.673405000	1.529292000
6	-1.154986000	0.673784000	1.529192000
1	-2.802011000	1.178050000	0.209463000
1	-2.801997000	-1.177897000	0.209713000
1	-0.906778000	-1.416650000	2.277506000
1	-0.906872000	1.417161000	2.277288000
22	0.692718000	-0.000114000	-0.067700000
6	-1.050737000	-1.660076000	-0.913977000
1	-1.712316000	-2.342332000	-1.444201000
6	0.259539000	-1.610867000	-1.188407000
1	0.710572000	-2.259567000	-1.928781000
6	2.909480000	0.000265000	-0.001567000
6	2.540491000	-0.000032000	1.174932000
1	2.597228000	-0.000233000	2.247614000
1	3.513633000	0.000535000	-0.889053000
1	0.710736000	2.259064000	-1.929109000
6	0.259604000	1.610501000	-1.188674000
6	-1.050646000	1.660019000	-0.914185000
1	-1.712097000	2.342418000	-1.444384000

Frequency	Intensity	Frequency	Intensity	Frequency	Intensity
61.2162	0.0798	681.8221	37.5218	1285.4573	2.8542
77.9041	0.1423	709.5987	0.4991	1292.9227	15.4298
91.5102	1.6002	728.8723	9.4718	1305.6766	0.8963
99.3888	15.0258	732.566	103.9166	1321.2903	15.2015
105.0673	0.0657	737.0872	24.0603	1321.727	1.3824
176.3534	0.6183	831.3242	57.0417	1555.2892	0.0003
193.3347	6.2797	864.6137	28.1024	1560.1286	6.9387
260.0892	0.2468	885.2972	12.3769	1587.4739	4.6953
269.5838	26.7003	901.0549	2.4059	1856.9697	75.1151
341.632	5.2154	977.2334	4.6776	3084.2882	0.1094
364.5929	1.4208	979.8503	2.1092	3088.0351	0.0966
385.4181	0.6668	985.7591	0.2977	3112.9644	0.0844
400.5571	0.8177	993.3902	7.4616	3113.8219	0.1464
477.5973	9.7984	999.7274	14.8524	3180.8559	1.2265
577.3308	13.9298	1083.5928	2.2925	3188.7162	3.1434
614.2222	15.7594	1113.1789	0.1164	3188.7601	4.2898
641.9379	33.6872	1123.9292	19.0371	3205.6345	0.6825
675.0629	0.2915	1159.0252	0.4817	3280.7122	101.4657
680.0501	10.3785	1176.7748	5.8138	3356.4622	89.8094

Table S85. Cartesian coordinates for the optimized geometry of isomer 5n-doublet $Ti^+(C_2H_2)_5$ followed by its predicted frequencies (cm^{-1}) and IR intensities (km/mol).

Z	x	y	z
6	-2.948913000	0.000114000	-0.684304000
6	-1.863452000	0.000228000	-1.607835000
6	-0.533732000	0.000266000	-1.329191000
6	-2.948911000	-0.000001000	0.684308000
6	-1.863446000	-0.000043000	1.607833000
6	-0.533730000	0.000023000	1.329170000
1	-2.166114000	-0.000129000	2.653209000
1	-3.929236000	0.000122000	-1.147050000
1	-2.166132000	0.000303000	-2.653208000
1	0.158739000	0.000353000	-2.208324000
1	-3.929233000	-0.000067000	1.147059000
1	0.158762000	0.000004000	2.208290000
22	0.887166000	-0.000010000	-0.000005000
6	2.009334000	2.066670000	0.605466000
6	2.009284000	2.066796000	-0.605124000
1	2.072837000	2.214854000	-1.662240000
1	2.073035000	2.214483000	1.662606000
1	2.072698000	-2.214640000	-1.662596000
6	2.008871000	-2.066952000	-0.605446000
6	2.008842000	-2.067083000	0.605140000
1	2.072121000	-2.215158000	1.662268000

Frequency	Intensity	Frequency	Intensity	Frequency	Intensity
35.7542	0.215	616.21	0	1301.7224	0.7088
49.6593	0	620.9538	0.0178	1355.1673	6.981
73.4316	0.1753	621.1168	0.5231	1362.2391	5.076
76.5455	0.6852	660.1885	0	1468.9294	9.6881
94.9831	0	673.4356	16.6368	1497.0058	8.4476
120.4125	0.0212	702.4697	0	1570.1707	1.1953
146.0844	3.7184	722.7626	0.3352	1633.6689	0.0102
207.4261	3.2077	755.1057	73.4916	1976.4663	27.2541
222.5191	15.43	759.3369	47.2272	1988.3798	16.0443
238.7869	20.5365	779.0083	53.5718	2802.3719	11.5294
266.4089	0	817.1065	1.2272	2803.9041	60.5793
271.9015	19.2109	834.9687	1.6185	3114.4892	5.8067
287.0766	0	886.7683	15.9679	3115.0961	0.119
330.5929	0.0233	964.277	0	3161.3789	1.1225
340.6989	10.5702	996.6691	0.053	3178.047	5.5104
435.1915	41.5271	1009.0408	0.0649	3345.6511	0.0168
435.6457	1.9806	1033.9264	0	3346.6695	308.2975
514.0242	0	1115.4034	27.1721	3434.855	116.8859
568.4418	105.4498	1183.821	1.4046	3439.0182	67.9722

Table S86. Cartesian coordinates for the optimized geometry of isomer 5o-doublet $Ti^{+}(C_2H_2)_5$ followed by its predicted frequencies (cm^{-1}) and IR intensities (km/mol).

Z	x	y	z
6	-2.298110000	-0.581110000	0.753655000
6	-2.298084000	-0.581164000	-0.753663000
6	-1.257779000	-0.269888000	1.559650000
1	-3.257968000	-0.886623000	-1.165262000
1	-1.345729000	-0.314984000	2.637763000
6	-1.257724000	-0.270007000	-1.559645000
1	-1.345634000	-0.315169000	-2.637759000
1	-3.257999000	-0.886571000	1.165242000
22	-0.072224000	0.121505000	0.000006000
6	1.816380000	-0.935416000	-1.007459000
6	1.176942000	-1.744593000	0.000194000
1	0.692784000	-2.707882000	0.000383000
1	1.958115000	-1.025843000	-2.073479000
1	2.999306000	0.781562000	-0.000351000
6	2.240723000	0.018551000	-0.000159000
1	1.958450000	-1.025206000	2.073523000
6	1.816552000	-0.935108000	1.007496000
6	0.859335000	2.540824000	-0.000052000
6	-0.348932000	2.476892000	-0.000037000
1	1.899567000	2.766868000	0.000067000
1	-1.407776000	2.626855000	-0.000141000

Frequency	Intensity	Frequency	Intensity	Frequency	Intensity
24.9479	0.0137	673.2092	15.9911	1198.847	0.0005
76.138	0.2787	746.2792	3.6875	1268.4835	0.2856
90.7804	1.1978	750.93	23.4771	1302.7733	14.402
115.6439	0.4842	755.2381	56.7052	1307.6702	24.8591
124.9708	3.8694	764.5757	61.3327	1344.6272	15.5426
125.6838	1.1131	772.1153	44.1243	1367.6541	1.1477
146.68	6.7145	785.0578	14.4526	1444.1726	25.7961
207.1542	6.2015	795.2021	0.2451	1574.3255	4.3437
223.3928	1.0782	839.0655	0.1695	1994.9268	5.0821
253.6942	0.2389	845.8959	5.4932	3106.1265	0.5164
263.7095	2.2568	885.6381	2.2302	3120.7157	2.5116
306.2998	9.2317	912.674	5.9659	3188.5957	1.1147
313.7695	3.4254	958.0345	28.1255	3188.7515	2.4857
406.3362	0.7896	974.2473	13.9785	3233.2148	3.349
469.2201	4.5032	993.9095	0.3854	3241.4961	11.1723
568.9261	33.2777	1015.5668	0.0047	3256.2361	12.3744
628.4968	74.4297	1092.5054	0.3845	3269.5692	4.8054
632.3019	0.0475	1092.8408	15.0725	3365.4181	126.2847
652.8662	18.835	1116.6594	2.9825	3469.6286	52.4521

Table S87. Cartesian coordinates for the optimized geometry of isomer 5p-doublet $Ti^+(C_2H_2)_5$ followed by its predicted frequencies (cm^{-1}) and IR intensities (km/mol).

Z	x	y	z
6	2.585192000	0.000008000	0.344529000
6	1.625407000	1.112123000	0.680104000
6	0.634882000	0.000003000	1.190941000
6	1.625415000	-1.112113000	0.680115000
1	3.598840000	0.000008000	-0.031689000
1	1.972655000	1.806126000	1.453402000
1	0.419167000	0.000010000	2.258178000
1	1.972665000	-1.806116000	1.453412000
22	-0.830565000	-0.000011000	-0.215637000
6	1.125733000	1.890209000	-0.522093000
1	1.790472000	2.671549000	-0.891303000
6	-0.039248000	1.607705000	-1.135455000
1	-0.340887000	2.142095000	-2.033169000
6	-3.166134000	-0.602311000	0.567089000
6	-3.166135000	0.602294000	0.567070000
1	-3.230075000	1.670968000	0.585639000
1	-3.230077000	-1.670984000	0.585690000
1	-0.340817000	-2.142070000	-2.033210000
6	-0.039213000	-1.607698000	-1.135473000
6	1.125763000	-1.890190000	-0.522097000
1	1.790525000	-2.671510000	-0.891312000

Frequency	Intensity	Frequency	Intensity	Frequency	Intensity
31.6224	0.0163	677.2645	12.5236	1224.6923	15.8297
53.7956	0.5737	704.6518	2.3333	1257.0206	0.6278
61.9183	0.1726	715.5721	58.1834	1264.2115	2.2167
125.4843	6.3653	787.9122	62.0152	1300.9809	5.5854
133.124	0.7134	794.343	2.9248	1313.9234	57.6906
136.2214	6.8881	800.3748	50.821	1314.2184	0.0195
161.5008	8.2533	836.9322	17.3713	1543.3532	10.9567
196.4336	4.3497	838.0854	97.3766	1554.7119	59.2156
200.9866	2.7753	927.0651	13.8941	2031.3763	20.6612
249.6011	19.1276	942.5843	1.2436	3010.5092	7.0033
283.0793	10.0442	970.8609	1.1743	3011.333	4.1075
291.9746	3.7832	997.1024	0.6568	3097.8366	9.9841
418.7613	27.0758	1000.0204	0.2098	3097.9247	5.2903
463.5997	12.9488	1016.2695	14.7102	3104.1457	2.0964
517.8894	29.0159	1039.2469	2.4398	3131.8169	1.07
610.7647	10.7648	1065.6389	0.0762	3132.1311	0.5308
637.9624	0.0633	1128.5882	4.3525	3205.4862	0.8621
654.4183	65.7073	1134.4565	2.5111	3335.838	202.839
657.3289	42.2487	1188.184	1.6575	3438.8621	22.8795

Table S88. Cartesian coordinates for the optimized geometry of isomer 5q-doublet $Ti^+(C_2H_2)_5$ followed by its predicted frequencies (cm^{-1}) and IR intensities (km/mol).

Z	x	y	z
22	1.135549000	0.000012000	-0.129317000
6	2.396620000	1.532816000	0.009480000
6	3.494733000	0.745131000	0.163527000
1	4.484203000	1.178260000	0.295265000
1	2.466021000	2.613629000	0.012125000
6	3.494685000	-0.745249000	0.163558000
6	2.396519000	-1.532869000	0.009544000
1	2.465849000	-2.613685000	0.012234000
1	4.484126000	-1.178438000	0.295315000
6	-1.728008000	0.000063000	-0.748727000
6	-0.536654000	0.000024000	-1.468599000
1	-0.614076000	0.000004000	-2.554421000
1	-2.703791000	0.000075000	-1.229174000
1	-2.613345000	0.000109000	1.226233000
6	-1.675958000	0.000086000	0.674503000
6	-0.435400000	0.000081000	1.305250000
1	-0.423952000	0.000110000	2.393324000
6	-5.491692000	-0.599036000	0.124225000
6	-5.491698000	0.598917000	0.124298000
1	-5.533005000	1.662198000	0.125963000
1	-5.532993000	-1.662317000	0.125760000

Frequency	Intensity	Frequency	Intensity	Frequency	Intensity
7.774	0.214	632.4424	0.0001	1291.5122	2.8446
13.4495	0.263	653.0333	2.2328	1300.6169	26.2294
21.6882	0.4918	665.1613	26.8769	1330.9568	138.4035
34.2049	0.0008	723.4952	0.7607	1342.8668	1.1607
53.2974	2.7172	724.6379	7.7771	1431.1427	99.5359
57.9444	1.4364	752.894	61.5141	1474.2957	66.8703
86.6104	0.0056	771.1648	93.8063	1504.9016	2.6393
123.4646	0.0004	783.3217	154.0063	1554.3617	5.2707
153.805	10.4975	821.175	0.018	2065.9099	7.0586
162.9628	0.0154	874.6155	11.0028	3112.7669	0.4266
172.426	0.0449	958.4281	0.0023	3125.0237	0.7038
194.4019	3.7921	991.3668	0.1951	3126.1778	1.5527
271.1986	2.4621	997.4138	0.0552	3131.1028	0.1148
285.4025	0.0088	1021.7162	0.0099	3143.6997	0.0175
449.4825	1.62	1028.7699	0.0009	3150.6448	3.587
482.5229	1.4891	1095.5096	23.4016	3187.4681	1.0171
548.4278	8.0769	1109.3903	14.2923	3187.4803	9.6222
614.5189	85.5005	1115.0514	3.499	3404.0147	105.7013
621.4916	30.6863	1140.1809	0.7414	3503.0743	1.5969

Table S89. Cartesian coordinates for the optimized geometry of isomer 5r-doublet $Ti^{+}(C_2H_2)_5$ followed by its predicted frequencies (cm^{-1}) and IR intensities (km/mol).

Z	x	y	z
22	-0.963730000	0.056111000	0.000002000
6	1.157372000	-0.230299000	0.000028000
6	1.809175000	1.000380000	0.000013000
1	2.894890000	1.068137000	0.000026000
1	1.792404000	-1.114850000	0.000052000
6	1.032857000	2.193991000	-0.000021000
6	-0.354478000	2.097213000	-0.000041000
1	-0.923862000	3.024479000	-0.000072000
1	1.544913000	3.153729000	-0.000031000
1	-2.167510000	-0.528491000	-2.613367000
6	-2.107058000	-0.494934000	-1.532524000
6	-3.117524000	-0.952041000	-0.745130000
1	-4.024286000	-1.369137000	-1.178512000
6	-3.117541000	-0.952002000	0.745136000
1	-4.024313000	-1.369075000	1.178519000
6	-2.107092000	-0.494857000	1.532530000
1	-2.167569000	-0.528359000	2.613373000
6	4.930596000	-1.181605000	0.598967000
6	4.930573000	-1.181593000	-0.598962000
1	4.968023000	-1.198191000	-1.662213000
1	4.968087000	-1.198221000	1.662216000

Frequency	Intensity	Frequency	Intensity	Frequency	Intensity
5.7887	0.3641	632.6588	0.0012	1297.1626	1.7767
15.9609	0.129	651.8269	2.2584	1300.6531	26.2206
18.3849	0.1697	665.1059	24.9734	1331.8074	111.2369
31.8846	0.0365	724.3423	10.6798	1344.5276	0.9073
55.948	2.7074	730.7627	2.9275	1431.2671	104.3646
57.3202	1.5546	753.1021	59.1314	1470.4731	96.0252
81.5965	0.0704	771.1218	90.726	1505.561	9.7529
125.6469	0.0441	782.4221	149.5726	1554.4546	5.2032
155.7707	10.0922	820.8519	0.0036	2066.0365	6.2853
158.878	0.0491	874.7909	10.9638	3112.776	0.4291
188.5694	0.1428	954.925	0.0734	3123.4396	2.5187
193.1078	3.5741	991.3762	0.2201	3126.1863	1.49
271.8509	3.114	997.226	0.0016	3129.4848	5.6148
286.6576	0.0062	1021.6325	0.0336	3140.862	3.934
449.7936	1.0088	1024.1237	0.0777	3148.816	1.2384
482.6066	1.5417	1095.5048	24.991	3187.2953	1.0035
548.2124	7.6142	1111.9116	8.2712	3187.302	9.5218
614.1517	85.6771	1115.0548	3.4334	3404.4787	104.2771
622.6713	30.1172	1137.7825	7.9721	3503.4913	1.4798

Table S90. Cartesian coordinates for the optimized geometry of isomer 5s-doublet $Ti^{+}(C_2H_2)_5$ followed by its predicted frequencies (cm^{-1}) and IR intensities (km/mol).

Z	x	y	z
6	-1.441616000	0.029746000	0.750811000
6	-0.355879000	-0.011495000	1.565148000
1	-0.429609000	0.065842000	2.642049000
1	-2.453205000	0.060500000	1.151891000
22	0.786301000	-0.432294000	0.000052000
6	3.702681000	-0.212532000	0.000077000
6	2.778885000	-1.248274000	0.000222000
1	3.178382000	-2.260994000	0.000397000
1	4.775872000	-0.389692000	0.000135000
6	1.861431000	1.365020000	-0.000230000
6	3.231368000	1.133423000	-0.000156000
1	3.953581000	1.946771000	-0.000270000
1	1.510057000	2.392920000	-0.000411000
1	-2.453200000	0.060121000	-1.151969000
6	-1.441612000	0.029495000	-0.750874000
6	-0.355873000	-0.012029000	-1.565194000
1	-0.429600000	0.064944000	-2.642121000
6	-5.209482000	-0.518309000	0.000103000
6	-5.182711000	0.679452000	-0.000057000
1	-5.199287000	1.743462000	-0.000197000
1	-5.274749000	-1.580393000	0.000244000

Frequency	Intensity	Frequency	Intensity	Frequency	Intensity
16.9222	0.387	644.868	52.5184	1276.8556	38.6103
19.67	0.0139	657.87	17.8489	1295.8694	9.3754
31.9374	0.0006	660.3414	3.1398	1324.6489	28.2675
33.6132	11.7784	735.2708	12.9558	1344.7576	29.786
56.2096	2.8472	741.1652	20.0799	1418.7022	75.1514
60.7106	0.5782	761.6067	17.763	1479.0534	70.6548
76.2763	2.1111	771.1628	104.4409	1500.5788	15.6262
107.1236	3.008	784.157	159.5475	1552.554	3.8081
123.6461	0.1472	841.0971	0.5745	2065.3098	9.6507
173.0517	0.0023	847.387	12.1475	3107.4155	0.009
237.5358	4.7262	962.1274	0.7614	3115.9377	44.8039
239.2002	0.5086	992.6485	43.0544	3121.357	1.1538
272.1992	0.8572	997.2821	1.595	3133.258	0.8169
307.9188	1.2544	1013.7563	0.2058	3145.6933	2.5177
455.9514	4.6002	1026.2721	6.5523	3160.6788	0.1411
490.4808	2.0984	1078.775	4.0943	3198.9246	1.3086
557.1377	5.2046	1099.2451	8.3553	3199.453	12.094
623.9505	37.4961	1114.1023	11.6687	3403.832	106.2716
632.3762	0.0094	1138.2227	7.6472	3502.8947	1.5315

Table S91. Cartesian coordinates for the optimized geometry of isomer 5t-doublet $Ti^{+}(C_2H_2)_5$ followed by its predicted frequencies (cm^{-1}) and IR intensities (km/mol).

Z	x	y	z
22	1.087225000	-0.000018000	0.000003000
6	-0.155670000	-0.000145000	-1.553944000
6	-1.246287000	-0.000030000	-0.752683000
1	-2.256162000	-0.000038000	-1.159129000
1	-0.240693000	-0.000262000	-2.633486000
6	-1.246204000	0.000121000	0.752942000
6	-0.155501000	0.000145000	1.554085000
1	-0.240409000	0.000260000	2.633637000
1	-2.256034000	0.000209000	1.159502000
6	2.972751000	1.552576000	-0.000182000
6	1.928946000	2.173037000	-0.000206000
1	1.120991000	2.875877000	-0.000254000
1	3.990880000	1.230885000	-0.000168000
1	1.121065000	-2.875912000	0.000276000
6	1.928993000	-2.173040000	0.000128000
6	2.972780000	-1.552547000	-0.000009000
1	3.990903000	-1.230835000	-0.000140000
6	-5.076560000	-0.598955000	-0.000229000
6	-5.076620000	0.598886000	0.000074000
1	-5.114720000	1.662071000	0.000340000
1	-5.114552000	-1.662144000	-0.000498000

Frequency	Intensity	Frequency	Intensity	Frequency	Intensity
17.1987	0.0195	624.8684	0	1095.5773	7.7611
20.3382	0.0059	629.5703	0	1115.2186	4.5273
24.8531	0	631.6818	35.2324	1299.7223	31.7112
40.6242	0	639.8544	0.2809	1343.7725	50.4411
50.7239	3.3742	644.6277	31.0357	1449.1396	14.6522
66.3056	0.8473	652.0365	13.8549	1576.9507	1.5298
88.8056	0.0854	656.289	5.5402	1953.4697	36.7436
90.0261	0.1214	698.419	60.1734	1969.0012	10.5214
103.5124	0	703.6245	3.0168	2066.5872	6.377
123.3401	1.7147	738.2252	24.4313	3106.8056	0.4819
140.3222	9.4625	740.5112	0	3116.7597	6.6948
142.6202	0.0346	765.9336	58.4489	3186.396	3.1629
192.6663	0.1447	770.1177	190.7084	3186.8287	3.5628
222.9464	10.0358	781.2671	145.3618	3337.9438	111.7831
256.4141	0	794.7323	0.6166	3341.5032	144.7712
296.6484	11.2777	845.0778	0	3406.3458	100.9385
330.3936	2.5408	849.411	4.7308	3429.0422	83.5031
337.8645	0.1352	1008.0321	1.1277	3435.1139	64.1684
475.4999	5.5014	1036.1865	0	3505.2353	1.0852

Table S92. Cartesian coordinates for the optimized geometry of isomer 5a-quartet $Ti^{+}(C_2H_2)_5$ followed by its predicted frequencies (cm^{-1}) and IR intensities (km/mol).

Z	x	y	z
22	-0.388416000	-0.000058000	-0.060948000
6	-1.847627000	1.405290000	-0.507592000
6	-2.712204000	0.707475000	0.341276000
1	-3.361368000	1.226907000	1.046309000
1	-2.053609000	2.420672000	-0.829556000
6	-2.712259000	-0.707366000	0.341366000
6	-1.847751000	-1.405355000	-0.507428000
1	-2.053832000	-2.420737000	-0.829326000
1	-3.361454000	-1.226659000	1.046473000
6	1.743196000	1.212888000	0.767733000
6	1.690112000	-0.000242000	1.470537000
6	1.800552000	1.213794000	-0.628665000
1	1.650199000	-0.000449000	2.551421000
1	1.826800000	2.150860000	-1.168270000
6	1.743285000	-1.213112000	0.767272000
6	1.803979000	0.000282000	-1.333269000
1	1.728914000	-2.150987000	1.306731000
1	1.841845000	0.000489000	-2.414046000
6	1.800691000	-1.213490000	-0.629122000
1	1.827067000	-2.150352000	-1.169073000
1	1.728757000	2.150561000	1.307542000

Frequency	Intensity	Frequency	Intensity	Frequency	Intensity
12.4762	0.5126	759.299	65.5839	1202.8648	2.0113
63.2776	1.0152	900.1976	48.5442	1334.2551	0.6379
69.7296	0.7114	906.6294	33.8023	1389.3101	0.0009
157.5996	0.0596	916.583	0.8152	1434.8514	20.602
196.8862	3.7008	923.8598	0.3933	1435.6582	12.7002
208.3177	0.5546	963.3404	16.1558	1505.0285	20.4259
250.181	0.0951	996.908	0.0743	1507.1446	23.0774
279.7996	0.0252	1012.632	3.8456	1589.697	0.216
301.3576	50.9056	1026.6199	0.0285	1601.8226	13.8666
412.4814	1.3441	1027.2973	0.0231	3092.3458	0.0372
414.5609	0.8246	1046.0331	1.075	3105.7495	3.4505
444.6528	6.0432	1047.8844	0.7297	3167.1122	1.873
523.1898	10.2258	1053.4136	0.3978	3167.2182	0.3536
608.4383	4.8886	1063.0634	3.0719	3193.1383	0.0584
615.102	0.0565	1072.2323	8.3067	3198.1981	0.0853
630.5351	43.9267	1142.3732	44.8045	3201.3274	0.3666
667.1948	47.7164	1172.0504	21.6865	3209.4648	5.411
699.8991	0.5331	1189.9671	0.0172	3210.3537	5.3983
727.4413	4.3652	1195.6462	0.0142	3216.1035	1.7976

Table S93. Cartesian coordinates for the optimized geometry of isomer 5b-quartet $Ti^+(C_2H_2)_5$ followed by its predicted frequencies (cm^{-1}) and IR intensities (km/mol).

Z	x	y	z
6	-1.458030000	-0.832204000	-1.219677000
6	-1.826759000	0.531782000	-1.222586000
6	-1.305577000	-1.514884000	0.000768000
1	-1.975922000	1.052683000	-2.157963000
1	-1.038444000	-2.562201000	0.001074000
6	-2.023804000	1.198645000	0.000086000
6	-1.456450000	-0.831074000	1.220833000
1	-2.297242000	2.246112000	-0.000208000
1	-1.317312000	-1.353824000	2.156733000
6	-1.825451000	0.532851000	1.223031000
1	-1.973634000	1.054523000	2.158134000
1	-1.320179000	-1.355792000	-2.155303000
22	0.236502000	0.337011000	-0.000924000
6	2.353830000	1.004615000	-0.002785000
6	2.335918000	-0.046386000	-1.004176000
1	2.578441000	-0.088995000	-2.055746000
1	2.695843000	2.028125000	-0.005639000
1	2.578402000	-0.077544000	2.056241000
6	2.335903000	-0.040788000	1.004445000
1	2.020440000	-2.119359000	0.005915000
6	2.011513000	-1.042219000	0.002910000

Frequency	Intensity	Frequency	Intensity	Frequency	Intensity
22.7166	0.0531	762.5226	41.8791	1242.0522	4.4445
75.5752	0.2323	846.2456	3.0958	1292.3452	22.5107
94.5971	0.827	880.8233	0.3085	1333.7766	1.1334
210.7238	4.9947	895.0377	0.1488	1352.7067	0.8853
257.6568	1.3309	901.0233	1.212	1376.2383	0
264.6173	1.1068	945.218	29.939	1486.0453	12.7875
302.9906	10.4224	955.8227	5.049	1486.6572	12.5615
356.1146	0.001	958.6873	1.4491	1538.2493	2.7135
410.9285	0.0965	981.9423	8.8554	1542.5462	0.0032
419.7873	0.2929	991.9128	0.5595	3190.0932	0.5248
426.5051	0.0621	1002.2257	0.627	3202.5664	0.2705
572.5196	18.3049	1029.2751	4.6219	3204.8702	0.3993
610.8258	1.4362	1034.6283	3.3344	3212.7181	3.7344
612.6438	0.1948	1041.1682	0.087	3215.1257	6.2638
657.6008	40.557	1062.3195	0.0231	3219.5161	1.2596
667.1642	0.5489	1172.9622	0.862	3220.3956	2.8492
673.8302	0.0115	1176.4817	0.6268	3229.609	4.5986
728.3222	0.0484	1181.9039	0.0388	3239.6799	13.8734
749.2891	43.0177	1188.7723	0.0051	3258.6573	6.9061

Table S94. Cartesian coordinates for the optimized geometry of isomer 5d-quartet $Ti^+(C_2H_2)_5$ followed by its predicted frequencies (cm^{-1}) and IR intensities (km/mol).

Z	x	y	z
1	-1.217581000	-2.508434000	0.435904000
6	-1.193386000	-1.426288000	0.437335000
6	-2.187789000	-0.722696000	-0.174286000
6	-2.187790000	0.722690000	-0.174284000
6	-1.193394000	1.426285000	0.437344000
6	0.000005000	0.801730000	1.167028000
6	0.000009000	-0.801725000	1.167025000
6	1.193410000	-1.426282000	0.437345000
6	2.187797000	-0.722688000	-0.174292000
6	2.187786000	0.722700000	-0.174304000
6	1.193392000	1.426291000	0.437330000
1	-2.992561000	-1.247459000	-0.672013000
1	-2.992562000	1.247452000	-0.672013000
1	-1.217595000	2.508431000	0.435911000
1	1.217585000	2.508437000	0.435890000
1	2.992550000	1.247463000	-0.672045000
1	2.992572000	-1.247445000	-0.672020000
1	1.217613000	-2.508429000	0.435921000
1	0.000011000	1.122170000	2.210756000
1	0.000004000	-1.122168000	2.210752000
22	-0.000013000	-0.000005000	-1.081568000

Frequency	Intensity	Frequency	Intensity	Frequency	Intensity
109.2774	1.6847	885.1112	0	1312.2331	18.5091
130.25	0	894.9235	0.38	1376.8682	0
211.1885	4.7987	898.4274	4.5363	1385.5931	0.8945
213.9314	1.1067	958.8601	0	1426.9269	10.6402
246.9012	0.4325	960.2722	0.3539	1458.5227	1.7498
286.2091	2.1571	966.1115	0.2778	1511.914	72.8453
315.9398	0.1099	977.3495	3.9536	1554.9597	2.8574
441.1284	0	980.1801	1.8179	1604.495	0
460.9137	0.1144	993.2753	0	1606.6856	2.4722
488.452	0.4421	1014.4678	3.8199	3019.8891	4.2325
519.2386	0	1015.9206	0.1067	3053.0075	0.0253
538.0343	8.2593	1083.2745	0.38	3180.6337	0
582.602	0.2424	1179.2288	9.2706	3182.5545	0.3904
624.536	6.3613	1197.3252	0	3183.8387	0.6715
699.1924	35.3719	1199.6706	0.2404	3187.3368	0.0047
749.2362	23.4239	1209.9926	21.5273	3195.2526	0
818.0382	0	1215.257	4.0621	3196.5625	1.5733
822.1096	63.2549	1218.582	0	3203.5224	4.6877
822.3078	4.1307	1264.6924	2.8624	3204.3834	0.6569

Table S95. Cartesian coordinates for the optimized geometry of isomer 5e-quartet $Ti^{+}(C_2H_2)_5$ followed by its predicted frequencies (cm^{-1}) and IR intensities (km/mol).

Z	x	y	z
6	-1.612300000	-0.699516000	1.128981000
6	-1.534784000	-1.398696000	-0.089207000
6	-1.612307000	0.699945000	1.128721000
1	-1.531476000	-2.479834000	-0.089983000
1	-1.687899000	1.242488000	2.061091000
6	-1.444459000	-0.702155000	-1.303502000
6	-1.534778000	1.398682000	-0.089716000
1	-1.379548000	-1.244151000	-2.236784000
1	-1.531452000	2.479820000	-0.090883000
6	-1.444444000	0.701701000	-1.303760000
1	-1.379533000	1.243356000	-2.237239000
1	-1.687897000	-1.241718000	2.061548000
22	0.512429000	0.000007000	0.035056000
6	2.168329000	-1.555599000	-0.391554000
6	1.555470000	-1.961008000	0.593664000
1	1.265044000	-2.579025000	1.419865000
1	2.903544000	-1.470883000	-1.166527000
1	2.903608000	1.470844000	-1.166471000
6	2.168353000	1.555585000	-0.391539000
1	1.264983000	2.579091000	1.419794000
6	1.555452000	1.961038000	0.593636000

Frequency	Intensity	Frequency	Intensity	Frequency	Intensity
7.2697	0.0104	646.3619	48.4133	1189.9346	0.2976
58.3427	0.3453	663.5694	3.8623	1345.2637	0.2154
104.2292	1.6071	665.8016	9.1506	1383.2344	0
120.9773	2.7206	683.3197	0.6584	1494.6786	15.4912
136.0846	0.039	697.0713	27.539	1501.727	14.9201
142.1712	0.9908	706.4188	38.7411	1564.7972	1.4766
217.337	3.6902	730.5985	65.3172	1574.874	0.8932
219.1277	0.8363	768.054	80.7186	1866.7484	104.9754
250.8366	1.9584	901.3535	0.0176	1877.5889	102.3536
284.557	19.1942	906.7062	0.3833	3196.1554	0.0004
309.0566	4.487	991.6837	0.0241	3202.1641	0.0283
349.5913	10.1917	991.9681	3.7288	3202.2719	0.2008
389.2541	17.3459	997.651	0.2561	3212.3977	3.151
414.1952	0.5159	1011.9471	0.6643	3212.4655	2.6723
415.9085	0.1073	1038.0909	1.8088	3218.5758	2.5624
605.0313	2.5043	1042.1755	0.3465	3300.9446	16.7418
611.9334	0.0993	1048.9336	2.2702	3301.9446	129.4179
618.2519	1.2766	1187.3088	0.0932	3375.4372	72.4366
642.1258	6.4036	1187.873	0.1827	3376.9279	65.6514

Table S96. Cartesian coordinates for the optimized geometry of isomer 5f-quartet $Ti^{+}(C_2H_2)_5$ followed by its predicted frequencies (cm^{-1}) and IR intensities (km/mol).

Z	x	y	z
6	1.194959000	1.571281000	-0.448040000
6	1.802949000	0.906459000	0.678670000
6	1.390618000	-0.229993000	1.374688000
6	0.467623000	-1.328367000	1.167318000
6	-0.586479000	-1.748483000	0.325450000
6	-1.737637000	-1.258862000	-0.414278000
6	-2.515165000	-0.160409000	-0.263557000
6	-2.218404000	0.959728000	0.610026000
6	-1.125066000	1.709555000	0.480750000
6	-0.161443000	1.691589000	-0.633963000
1	-0.511985000	2.085716000	-1.584542000
1	-0.932902000	2.490177000	1.213510000
1	-2.933766000	1.196005000	1.392254000
1	-3.465731000	-0.152389000	-0.788339000
1	-2.134034000	-2.022170000	-1.076691000
1	-0.615256000	-2.836188000	0.328611000
1	0.846156000	-2.183323000	1.719284000
1	2.112811000	-0.510381000	2.133309000
1	2.788795000	1.258387000	0.960824000
1	1.869255000	2.040680000	-1.164443000
22	1.086588000	-0.638250000	-0.927097000

Frequency	Intensity	Frequency	Intensity	Frequency	Intensity
74.9513	4.4843	817.1188	22.2277	1417.1959	2.0828
104.762	1.9755	826.4132	9.1865	1440.3675	9.5622
145.8557	0.7147	850.8306	1.7741	1454.6221	2.721
174.1538	1.0177	892.0006	3.4393	1472.2312	1.6447
245.5463	0.817	925.8388	3.9121	1486.1366	0.1602
279.6308	0.7508	940.9707	12.314	1507.8191	2.105
289.9089	3.802	946.1511	4.5294	1546.2919	1.3003
327.8875	3.2562	955.6422	0.2475	1597.4448	7.5616
358.2328	1.2586	989.0159	2.1974	1686.7394	0.7096
426.5734	1.1349	998.9474	1.5094	3096.483	0.6258
437.6582	1.3111	1009.8554	0.4876	3101.6807	0.3568
480.4192	2.1498	1040.509	0.4778	3122.9939	0.3865
519.1553	7.8473	1080.044	1.3085	3129.9131	0.2356
637.2396	24.5504	1206.6015	5.7394	3133.6227	1.1121
659.6063	31.2417	1236.1091	2.0057	3139.5635	1.6987
693.2766	4.1938	1251.7847	1.6175	3150.6948	0.7059
727.5134	8.1946	1308.0247	1.5363	3152.8706	0.4667
743.8025	78.4398	1371.9713	1.6999	3157.7776	1.0663
797.7444	15.7518	1398.7982	7.6942	3167.9143	0.3179

Table S97. Cartesian coordinates for the optimized geometry of isomer 5g-quartet $Ti^+(C_2H_2)_5$ followed by its predicted frequencies (cm^{-1}) and IR intensities (km/mol).

Z	x	y	z
6	2.425362000	-0.397935000	0.668402000
6	2.594126000	-0.125668000	-0.730743000
6	1.617383000	0.321840000	-1.596492000
6	-2.801827000	-0.583546000	-0.380018000
6	-2.737825000	0.703118000	0.213925000
6	-1.594438000	1.445049000	0.416962000
1	-3.706603000	1.137873000	0.452402000
1	3.344545000	-0.334358000	1.241259000
1	3.634335000	-0.168866000	-1.055260000
1	1.853049000	0.407873000	-2.652410000
1	-3.785933000	-0.923239000	-0.684349000
1	-1.791190000	2.386743000	0.942989000
22	0.443296000	0.986024000	-0.053180000
1	1.455913000	-0.626190000	2.531009000
6	1.286175000	-0.675916000	1.461786000
6	-1.734823000	-1.412737000	-0.723698000
1	-1.938550000	-2.151584000	-1.493490000
1	-0.782213000	-0.992055000	1.843098000
6	-0.025741000	-0.985501000	1.066995000
6	-0.421733000	-1.377019000	-0.249736000
1	0.324184000	-1.898843000	-0.839597000

Frequency	Intensity	Frequency	Intensity	Frequency	Intensity
90.5529	3.6654	801.727	5.5331	1317.858	22.1176
102.0878	1.537	808.1953	4.089	1355.1787	27.2649
139.2838	2.5383	833.2658	30.6956	1384.3537	40.406
163.1322	2.7657	891.0568	36.1535	1435.2143	10.7913
189.8106	4.1916	899.3406	24.608	1459.0249	15.5336
217.7978	4.631	928.2645	7.9034	1490.1563	25.061
277.9375	0.8852	941.5537	2.0595	1495.1102	7.5796
322.7581	5.4355	948.4068	13.3541	1531.6084	36.0111
331.5436	2.9368	970.1863	19.115	1542.426	13.0143
368.2711	1.2537	992.4289	10.1599	3027.3103	8.1072
417.7089	14.9272	1011.744	4.6807	3086.8418	0.2929
430.6503	9.017	1026.1201	2.8457	3117.5853	6.447
477.3061	7.1685	1088.6999	5.3436	3141.9214	0.5361
487.332	2.1896	1139.1094	8.0835	3154.3725	1.0261
579.5009	7.1545	1187.7064	8.5664	3157.5585	0.2296
630.6587	13.3809	1215.6875	9.1327	3162.7822	0.0766
650.6689	51.5022	1251.2883	2.1355	3167.4283	2.038
677.4923	56.7736	1269.4197	7.9091	3171.4658	0.3468
766.994	11.4311	1278.1374	49.9825	3177.8665	2.5001

Table S98. Cartesian coordinates for the optimized geometry of isomer 5h-quartet $Ti^{+}(C_2H_2)_5$ followed by its predicted frequencies (cm^{-1}) and IR intensities (km/mol).

Z	x	y	z
6	0.020862000	1.863811000	-0.583416000
6	-0.427804000	1.927901000	0.797670000
6	-1.413860000	1.000111000	0.997177000
6	-2.313465000	-0.998829000	-0.413673000
6	-1.514512000	-2.053880000	-0.049775000
6	-0.203256000	-1.852550000	0.395584000
1	-1.937870000	-3.053160000	-0.111547000
1	0.775191000	2.504249000	-1.018108000
1	-0.033790000	2.603319000	1.545663000
1	-1.953074000	0.825864000	1.916089000
1	-3.324956000	-1.173686000	-0.756835000
1	0.328888000	-2.759058000	0.700755000
22	0.849805000	-0.090019000	0.393338000
1	-0.619489000	0.630521000	-2.285249000
6	-0.686826000	0.874183000	-1.235662000
6	-1.840439000	0.425777000	-0.348272000
1	-2.689995000	1.064943000	-0.655741000
6	2.714540000	-0.296578000	-0.944747000
6	3.064929000	-0.513330000	0.211567000
1	3.676278000	-0.758162000	1.060362000
1	2.682096000	-0.164112000	-2.007542000

Frequency	Intensity	Frequency	Intensity	Frequency	Intensity
49.8652	0.0689	686.1933	3.3052	1263.4168	24.8246
85.898	0.7348	727.8545	68.662	1286.1508	10.9502
101.782	0.9444	771.3377	36.0386	1296.6616	0.6845
117.5303	1.4696	779.6206	0.5978	1388.0727	29.5224
166.3072	3.8679	792.2657	1.2926	1433.4534	25.1677
188.7843	1.1682	831.1367	0.5909	1436.5266	5.8038
243.321	10.7969	841.9253	1.8044	1505.4905	4.6944
281.0709	2.4843	852.6181	2.4509	1525.0859	2.9722
292.3795	14.0665	927.7818	8.8453	1879.6097	146.1677
337.0527	11.9098	968.3191	7.416	2903.9945	2.9989
378.7923	7.8332	999.8413	6.5935	3053.5212	4.6011
449.9712	3.1198	1003.4832	2.2692	3135.396	6.737
478.5812	3.2248	1024.5102	17.1588	3194.0953	0.1887
531.6244	32.2276	1034.6964	0.9871	3199.4168	1.0425
556.3681	5.6446	1091.4188	4.5082	3214.4272	3.6507
616.2101	41.9919	1096.9809	1.8335	3232.5604	5.9899
637.7023	45.6867	1128.5507	15.0587	3236.5677	3.4179
660.6514	9.1827	1169.6183	7.6969	3289.765	119.5075
672.8388	95.7447	1201.4156	4.488	3374.8526	116.7239

Table S99. Cartesian coordinates for the optimized geometry of isomer 5i-quartet $Ti^{+}(C_2H_2)_5$ followed by its predicted frequencies (cm^{-1}) and IR intensities (km/mol).

Z	x	y	z
6	-0.348048000	-1.578282000	1.078797000
6	-1.426231000	-1.144801000	0.040893000
6	-0.396278000	-1.651780000	-1.001062000
6	0.321815000	-2.272564000	0.038915000
1	-0.432069000	-1.764109000	2.139162000
1	-2.273162000	-1.823841000	0.093601000
1	-0.494104000	-1.863387000	-2.054876000
1	1.116690000	-3.006863000	0.044371000
22	0.586309000	0.086854000	0.002187000
6	-1.923074000	0.339316000	-0.009781000
1	-3.012710000	0.377961000	-0.020803000
6	-1.299919000	1.339891000	0.983318000
6	-1.281595000	1.265011000	-1.062974000
1	-1.395447000	1.345400000	-2.134340000
1	-1.420099000	1.489806000	2.046502000
6	-0.795538000	2.134982000	-0.064229000
1	-0.318790000	3.104658000	-0.095672000
6	2.528025000	1.248691000	0.165897000
6	2.827880000	0.102416000	-0.179530000
1	3.428478000	-0.744534000	-0.452889000
1	2.660186000	2.276834000	0.445373000

Frequency	Intensity	Frequency	Intensity	Frequency	Intensity
55.5414	0.1944	710.0767	89.6006	1204.4096	2.1668
76.7358	0.0122	739.1349	3.3636	1213.0535	0.1252
90.7855	0.2332	748.485	7.9947	1214.1545	0.8382
140.5395	0.0917	777.2705	2.3324	1319.1832	0.5508
185.3112	0.7539	799.9847	9.1494	1321.5314	21.012
195.3167	11.9918	872.7386	1.2596	1368.2682	9.7172
223.0535	6.8924	897.8559	0.2671	1388.4351	7.0399
305.0332	2.2771	916.3795	0.6868	1410.3364	5.2478
329.1334	2.8688	934.5151	11.0415	1854.7624	106.3904
343.6362	3.1653	940.2255	4.4679	3082.3297	0.0026
380.0347	0.3933	948.6826	27.2487	3121.8141	4.449
415.3609	5.0503	951.1443	56.1223	3198.6485	0.9977
468.8568	1.2693	971.0164	13.7519	3209.7113	0.467
486.1542	2.7578	972.3783	3.3688	3222.7117	2.517
620.3403	28.5957	1012.4731	0.5678	3226.452	7.9815
646.7353	26.5568	1035.5507	4.7505	3231.4089	2.2523
673.4492	12.8459	1122.3587	7.0486	3235.6237	6.2812
677.4899	2.0711	1151.0752	12.0406	3280.121	80.1941
694.3429	27.3046	1173.0516	0.1846	3352.7309	76.0821

Table S100. Cartesian coordinates for the optimized geometry of isomer 5j-quartet $Ti^+(C_2H_2)_5$ followed by its predicted frequencies (cm^{-1}) and IR intensities (km/mol).

Z	x	y	z
6	-1.741015000	-0.838200000	1.051889000
6	-2.284122000	0.162689000	0.005944000
6	-1.817132000	-0.918628000	-0.998609000
6	-1.411321000	-1.768784000	0.046560000
1	-1.804242000	-0.877504000	2.129871000
1	-3.372811000	0.204780000	0.048205000
1	-1.954166000	-1.037418000	-2.063725000
1	-1.064441000	-2.792860000	0.072343000
22	0.214912000	0.095272000	-0.044756000
6	-1.764314000	1.625840000	-0.070820000
1	-2.565213000	2.335286000	-0.266889000
6	-0.494803000	2.001702000	0.111199000
1	-0.220015000	3.048652000	0.079471000
1	2.280375000	-0.180593000	2.111604000
6	2.133269000	-0.157240000	1.044742000
6	2.423726000	0.820639000	0.013739000
1	2.770054000	1.843123000	0.031097000
6	2.193780000	-1.159343000	-0.002805000
6	2.179154000	-0.143029000	-1.043477000
1	2.392962000	-0.158963000	-2.099453000
1	2.306092000	-2.234374000	-0.008065000

Frequency	Intensity	Frequency	Intensity	Frequency	Intensity
41.2916	5.2368	738.8756	12.9643	1204.406	0.3018
70.9521	0.5637	746.7189	12.2175	1243.7575	4.4556
81.674	0.4534	760.7761	6.0609	1262.1173	0.5902
98.0319	3.2566	836.059	17.981	1288.4284	20.3449
137.9218	7.445	854.4496	14.2558	1316.7876	16.3523
163.1772	3.6444	904.3434	10.0913	1320.7417	14.4831
236.2212	5.4346	905.5788	5.9678	1355.8719	2.5921
273.33	9.4631	911.514	9.404	1409.2738	2.4532
330.5855	5.6241	951.5213	22.4038	1562.6598	0.5616
341.9963	11.5826	952.0965	10.0132	3082.1226	6.2548
369.0699	7.8539	959.2369	9.7527	3115.4828	0.8014
404.5919	6.885	962.0239	28.6217	3185.5455	1.1191
532.1304	15.9605	969.9911	1.1217	3207.4948	1.5666
567.6385	14.8484	988.1009	0.585	3216.9172	1.9995
643.739	32.8706	1061.0054	0.0776	3227.0642	9.0051
655.3391	0.3345	1093.0587	4.5433	3231.9202	4.1121
677.4415	41.4432	1097.336	8.0232	3234.8708	6.0646
680.1099	9.9929	1177.2272	4.5416	3252.5398	14.0585
695.5844	10.6663	1192.5291	0.0068	3260.5889	14.4564

Table S101. Cartesian coordinates for the optimized geometry of isomer 5k-quartet $Ti^{+}(C_2H_2)_5$ followed by its predicted frequencies (cm^{-1}) and IR intensities (km/mol).

Z	x	y	z
6	-1.866768000	-0.888588000	-1.025327000
6	-1.328935000	-1.631934000	0.104173000
6	-1.966372000	-0.658207000	0.971792000
6	-2.260150000	0.201003000	-0.156366000
1	-2.024702000	-1.117515000	-2.068669000
1	-1.078413000	-2.669508000	0.239011000
1	-2.221339000	-0.643968000	2.021046000
1	-2.919191000	1.040553000	-0.299262000
22	0.014746000	0.162670000	-0.056075000
1	3.079529000	0.987575000	-0.043037000
6	2.368420000	0.174923000	0.002303000
6	1.824494000	-0.734509000	-0.985373000
1	2.046899000	-0.921385000	-2.022663000
6	1.507466000	-1.603785000	0.135235000
1	1.358156000	-2.668532000	0.222668000
6	1.767451000	-0.555640000	1.103376000
1	1.931904000	-0.558217000	2.167800000
6	0.483472000	2.624031000	-0.265356000
6	-0.594230000	2.636358000	0.280863000
1	1.423962000	2.792952000	-0.739245000
1	-1.530302000	2.797389000	0.764084000

Frequency	Intensity	Frequency	Intensity	Frequency	Intensity
32.4751	0.056	658.3247	41.82	1189.4804	0.0035
78.0324	0.9386	716.6082	14.8713	1196.9026	0.0741
88.4967	0.2473	728.3857	0.4968	1245.3281	7.4062
119.0248	0.2729	741.3897	3.2643	1247.8096	1.0792
126.688	0.6914	751.4731	37.9283	1294.0305	24.8129
142.7305	3.2509	759.5027	46.7804	1298.2317	19.1072
198.2705	0.3667	766.2525	23.8828	1357.5429	1.5797
257.9793	3.5786	772.7346	43.0038	1358.7966	0.6396
270.3265	10.7888	782.2855	3.0873	2001.6087	3.979
296.6906	7.8936	854.5714	0.2674	3214.7351	1.5442
311.6969	0.1769	865.0342	1.2267	3226.0214	0.1746
351.5925	3.4716	906.4471	1.2436	3231.8609	6.4042
381.2398	0.7482	917.3824	1.5608	3236.297	1.2913
435.0538	0.8924	948.8779	28.4558	3253.2612	11.8827
559.3521	7.9198	950.5906	3.8844	3257.8449	4.4767
580.6295	68.0712	953.709	27.6604	3261.3168	10.1031
602.7953	10.901	960.9273	12.7151	3269.6861	7.232
630.9417	2.3438	1051.0091	0.0396	3380.1712	122.1628
646.4891	1.751	1077.6738	0.2275	3471.7938	49.8413

Table S102. Cartesian coordinates for the optimized geometry of isomer 5l-quartet $Ti^{+}(C_2H_2)_5$ followed by its predicted frequencies (cm^{-1}) and IR intensities (km/mol).

Z	x	y	z
6	1.669181000	0.373685000	-1.284867000
6	2.274649000	0.327126000	0.138836000
6	1.911575000	-1.159049000	0.042618000
6	1.428610000	-1.010237000	-1.273734000
1	1.625747000	1.144845000	-2.041202000
1	3.351328000	0.507647000	0.120380000
1	2.101401000	-2.000068000	0.693535000
1	1.096186000	-1.711001000	-2.030152000
22	-0.531104000	-0.146393000	-0.020144000
6	1.595167000	1.087041000	1.265427000
1	2.206734000	1.826464000	1.778893000
6	0.320502000	0.831339000	1.600675000
1	-0.104650000	1.350084000	2.456875000
6	-2.408450000	-1.359450000	0.286232000
6	-1.469329000	-2.087913000	0.607353000
1	-0.928929000	-2.940005000	0.972595000
1	-3.392858000	-0.983437000	0.088154000
1	-2.267959000	2.245946000	0.824138000
6	-1.984561000	1.856121000	-0.128553000
6	-1.727664000	1.529727000	-1.268452000
1	-1.660784000	1.449826000	-2.333275000

Frequency	Intensity	Frequency	Intensity	Frequency	Intensity
54.4318	0.1785	644.0645	15.6956	1177.3051	4.2575
77.1451	1.4121	648.8406	14.4408	1208.4295	0.0236
90.3457	0.2585	674.2485	2.4464	1303.178	0.5645
98.6447	0.4871	677.4288	6.0949	1318.9888	9.2109
111.9231	3.0348	692.2737	21.0601	1322.8053	10.6435
117.4239	0.4234	707.4592	16.5356	1409.0335	2.7295
140.1635	0.588	717.7751	19.0147	1557.0417	2.4171
151.4102	4.4322	725.3345	65.3349	1865.1001	89.526
179.7149	14.5672	730.5059	12.1781	1960.1752	31.6971
223.406	0.8489	758.7936	75.2473	3070.3879	0.3028
246.6476	5.4742	871.8644	18.5801	3107.9786	4.9585
288.5805	5.8013	897.5981	11.7835	3131.3984	4.8856
323.8796	11.5402	922.0642	12.0187	3185.3448	0.4489
355.6338	0.6004	941.045	6.6241	3220.2224	2.3746
375.5824	7.5895	965.4596	0.422	3230.5239	4.3446
402.2558	2.2584	973.0912	15.2355	3292.6589	76.0091
535.8628	18.0896	1004.0644	2.7288	3344.0278	122.4184
622.0873	22.1435	1111.0278	1.2315	3368.2126	73.7608
639.6181	3.5618	1130.0554	7.9474	3436.9573	72.8999

Table S103. Cartesian coordinates for the optimized geometry of isomer 5m-quartet $Ti^+(C_2H_2)_5$ followed by its predicted frequencies (cm^{-1}) and IR intensities (km/mol).

Z	x	y	z
6	-1.744758000	0.973173000	0.377297000
6	-1.859864000	-0.645220000	0.615528000
6	-0.723761000	-0.447181000	1.607973000
6	-0.647804000	0.893941000	1.423242000
1	-2.654236000	1.455768000	0.738104000
1	-2.822749000	-0.912925000	1.055798000
1	-0.296701000	-1.121715000	2.340314000
1	-0.109171000	1.688428000	1.924554000
22	0.849674000	-0.085943000	-0.299142000
6	-1.564316000	-1.516912000	-0.558261000
1	-2.351878000	-1.626476000	-1.312461000
6	-0.384013000	-2.049687000	-0.815995000
1	0.012849000	-2.848924000	-1.427320000
6	3.082772000	-0.116100000	-0.191479000
6	2.725682000	0.185737000	0.948016000
1	2.745901000	0.461319000	1.984369000
1	3.719885000	-0.320891000	-1.032348000
1	0.197465000	1.860088000	-2.361971000
6	-0.061065000	1.414525000	-1.403393000
6	-1.326524000	1.517779000	-0.965921000
1	-2.112275000	1.995746000	-1.549956000

Frequency	Intensity	Frequency	Intensity	Frequency	Intensity
75.1883	0.0586	675.777	18.9572	1215.8073	8.6404
91.1912	0.395	680.1457	30.0532	1291.1032	7.1717
116.9031	0.7174	687.9533	58.6603	1309.5994	6.5231
134.2736	2.0901	712.1112	77.2947	1326.4182	12.4596
159.0634	2.1446	756.8664	20.412	1334.7125	1.5166
181.9374	1.664	786.5863	46.1265	1549.1427	5.3267
205.5542	1.8862	832.928	17.2624	1567.1038	4.6532
249.4857	5.2894	891.0819	2.524	1576.4467	3.7149
269.6546	6.0081	895.971	4.6108	1862.4573	119.8755
299.2753	5.3855	907.4219	11.7705	3036.1095	5.545
337.2795	7.842	916.215	2.2281	3067.7279	0.5846
372.6137	1.6291	945.2676	7.4721	3077.1841	0.8874
379.4263	2.6496	997.8407	7.0974	3098.1527	8.9134
426.2545	8.4375	1011.5201	1.0563	3125.5485	2.162
524.7564	32.4408	1040.4456	12.882	3177.7021	0.285
571.3285	6.9453	1101.9147	1.136	3202.5197	2.0824
635.7722	34.5262	1114.1219	2.7869	3215.6403	42.7554
654.232	7.746	1140.105	6.2381	3281.3412	101.1377
664.699	1.1095	1185.7557	13.9206	3359.8955	94.3144

Table S104. Cartesian coordinates for the optimized geometry of isomer 5n-quartet $Ti^{+}(C_2H_2)_5$ followed by its predicted frequencies (cm^{-1}) and IR intensities (km/mol).

Z	x	y	z
6	-2.797729000	-0.714198000	-0.049732000
6	-1.844581000	-1.732869000	-0.144666000
6	-0.455125000	-1.660264000	-0.207913000
6	-2.797728000	0.714200000	-0.049741000
6	-1.844580000	1.732868000	-0.144694000
6	-0.455125000	1.660261000	-0.207948000
1	-2.294762000	2.723055000	-0.171089000
1	-3.810256000	-1.100076000	0.012673000
1	-2.294764000	-2.723056000	-0.171046000
1	0.009787000	-2.645218000	-0.315664000
1	-3.810255000	1.100079000	0.012663000
1	0.009787000	2.645213000	-0.315723000
22	0.784974000	-0.000001000	-0.154623000
6	2.876754000	-0.000010000	-1.003677000
6	2.102096000	-0.000026000	-1.963841000
1	1.752032000	-0.000045000	-2.979636000
1	3.782385000	0.000001000	-0.427531000
1	1.521566000	-1.660451000	2.304533000
6	1.469944000	-0.605249000	2.148454000
6	1.469984000	0.605289000	2.148429000
1	1.521588000	1.660495000	2.304491000

Frequency	Intensity	Frequency	Intensity	Frequency	Intensity
50.6953	0.3733	608.55	35.2916	1300.0821	20.4514
53.2908	0.0002	623.9045	0.3284	1329.5498	50.5626
62.8943	0.0637	639.0719	0.0065	1382.1914	63.3451
91.2275	0.2671	643.9707	78.5422	1470.0935	60.9741
101.0524	0.2903	657.2109	1.6899	1471.3844	129.9942
132.1794	1.6031	683.6228	1.1036	1530.3662	0.7474
134.5468	0.2518	686.6275	33.3574	1546.4771	8.4215
172.114	0.0915	716.1374	68.9709	1851.8468	122.0082
172.2545	0.3608	731.8201	1.0192	1981.4275	14.1066
213.3815	9.5186	767.3426	95.4579	3051.5885	2.2056
265.2548	1.0639	815.5287	0.1873	3051.6333	10.3587
287.0764	6.1106	826.2764	1.2796	3114.236	6.5689
322.8483	3.3641	835.7823	1.8864	3115.2894	1.8941
360.722	7.0836	945.5512	0.7032	3147.5941	1.2374
367.412	2.1912	1018.3778	2.1154	3165.3825	1.9959
458.3915	1.9872	1031.1349	0.2202	3277.77	80.902
487.5846	0.0339	1054.8349	1.8192	3351.982	93.8976
523.7844	2.1295	1156.6451	10.3725	3360.0633	137.4278
592.3641	7.891	1249.5331	3.1794	3449.7573	67.1476

Table S105. Cartesian coordinates for the optimized geometry of isomer 5o-quartet $Ti^{+}(C_2H_2)_5$ followed by its predicted frequencies (cm^{-1}) and IR intensities (km/mol).

Z	x	y	z
6	0.457725000	1.818680000	-0.720647000
6	0.457682000	1.818682000	0.720644000
6	0.927599000	0.742212000	-1.443597000
1	-0.003074000	2.650812000	1.236935000
1	0.872859000	0.750497000	-2.525012000
6	0.927501000	0.742211000	1.443625000
1	0.872698000	0.750498000	2.525036000
1	-0.003005000	2.650804000	-1.236968000
22	-0.751541000	-0.082775000	-0.000029000
6	1.718843000	-0.376181000	0.794268000
6	1.718904000	-0.376176000	-0.794192000
1	2.719019000	-0.447778000	-1.229207000
1	2.718925000	-0.447792000	1.229357000
1	0.696863000	-2.447402000	1.425638000
6	0.982517000	-1.715980000	0.680034000
1	0.696977000	-2.447393000	-1.425655000
6	0.982573000	-1.715977000	-0.680023000
6	-2.794708000	-0.952052000	-0.000023000
6	-2.954315000	0.279245000	0.000000000
1	-3.086972000	-1.987467000	-0.000052000
1	-3.496297000	1.208292000	0.000037000

Frequency	Intensity	Frequency	Intensity	Frequency	Intensity
36.0995	0.6588	735.6285	10.5844	1264.6153	17.7425
61.0262	0.1173	757.5381	37.5936	1284.7583	0.2645
78.6388	0.2896	795.9456	1.5227	1288.2937	24.3568
170.6453	1.617	830.0336	33.5949	1378.3401	7.4544
175.385	0.3093	841.6504	8.0707	1433.2778	1.5119
238.6131	0.7861	893.9275	14.1805	1486.0018	44.0958
274.9942	0.1895	937.715	3.2202	1531.5963	4.148
288.3579	1.0993	945.426	12.0411	1555.9779	0.0243
342.9406	0.5617	961.1545	1.2792	1822.6341	110.0057
417.7566	0.3032	973.5693	0.7038	3044.8871	0.0367
431.4392	6.2737	985.0693	1.2393	3051.0959	0.4462
449.2254	3.6031	985.163	0.575	3180.2852	0.9903
525.0685	0.5315	999.5479	0.5169	3182.4313	0.5014
542.554	6.8419	1047.1973	4.3316	3183.9721	0.0689
565.0878	0.149	1086.2781	1.6311	3192.6653	1.4013
650.6309	67.7065	1137.8379	16.7329	3201.6001	2.5397
675.0933	36.8267	1174.6096	0.2311	3206.2941	2.603
700.2259	41.9369	1199.3222	5.5994	3253.4153	73.7956
703.4561	26.1849	1206.4708	2.684	3321.703	75.5317

Table S106. Cartesian coordinates for the optimized geometry of isomer 5p-quartet $Ti^{+}(C_2H_2)_5$ followed by its predicted frequencies (cm^{-1}) and IR intensities (km/mol).

Z	x	y	z
6	2.640005000	0.000001000	0.187795000
6	1.707277000	1.115021000	0.609129000
6	0.866940000	0.000032000	1.220786000
6	1.707255000	-1.114992000	0.609158000
1	3.621177000	-0.000016000	-0.265423000
1	2.095906000	1.811623000	1.365277000
1	0.469867000	0.000049000	2.232705000
1	2.095869000	-1.811577000	1.365331000
22	-1.004749000	-0.000020000	-0.328344000
6	1.096664000	1.934839000	-0.519140000
1	1.742461000	2.732383000	-0.883948000
6	-0.119494000	1.703007000	-1.026670000
1	-0.483578000	2.350913000	-1.824187000
6	-3.235900000	0.000037000	0.202393000
6	-2.687246000	0.000034000	1.294391000
1	-2.451939000	0.000041000	2.339448000
1	-3.960006000	0.000047000	-0.590389000
1	-0.483574000	-2.351008000	-1.824128000
6	-0.119515000	-1.703071000	-1.026625000
6	1.096652000	-1.934847000	-0.519091000
1	1.742477000	-2.732377000	-0.883882000

Frequency	Intensity	Frequency	Intensity	Frequency	Intensity
76.529	1.133	650.0548	36.2654	1215.8462	0.9706
81.7046	0.0384	690.284	33.3095	1223.4795	5.3843
82.2217	0.5635	703.7071	13.4259	1267.8124	1.9696
110.9511	13.4776	710.2061	19.7029	1280.9281	6.1758
136.3732	6.9459	736.8346	110.8089	1334.613	0.3153
147.5177	2.6218	751.1455	25.009	1337.1733	19.7038
172.5728	0.4165	791.8689	5.9643	1564.4088	3.845
242.7805	7.4804	852.9224	11.885	1582.4397	62.309
273.5877	19.933	921.6675	12.9146	1910.7063	74.8712
296.8007	0.0506	929.5994	8.4907	2969.4769	0.7713
325.3133	19.3023	936.8871	2.3501	2973.1411	1.0604
343.0522	2.856	956.3232	10.5285	3093.3764	5.3216
376.8454	2.3757	995.105	0.3794	3093.5805	4.8468
417.1024	10.808	1001.5194	0.5911	3117.3427	2.5201
489.8984	2.212	1053.7598	4.3318	3117.5404	1.314
512.5635	27.0979	1075.8312	0.2278	3140.1814	2.0557
588.829	27.3363	1134.5399	0.1001	3214.0119	2.5299
618.7083	58.4595	1136.8264	1.246	3301.2946	143.0589
646.1917	1.2303	1179.6029	1.6391	3388.3987	112.5759

Table S107. Cartesian coordinates for the optimized geometry of isomer 5q-quartet $Ti^{+}(C_2H_2)_5$ followed by its predicted frequencies (cm^{-1}) and IR intensities (km/mol).

Z	x	y	z
22	0.884267000	0.110393000	0.000016000
6	2.626341000	1.057906000	0.000690000
6	3.308846000	-0.112430000	0.000448000
1	4.396736000	-0.124206000	0.000719000
1	3.131930000	2.014566000	0.001161000
6	2.727144000	-1.498683000	-0.000194000
6	1.413744000	-1.819759000	-0.000620000
1	1.067683000	-2.845910000	-0.001074000
1	3.494916000	-2.269622000	-0.000292000
6	-1.406478000	0.707568000	-0.722220000
6	-0.380020000	1.159235000	-1.476865000
1	-0.184280000	1.499525000	-2.481864000
1	-2.206619000	0.129221000	-1.195471000
1	-2.207294000	0.128458000	1.193853000
6	-1.406862000	0.707093000	0.721433000
6	-0.380813000	1.158255000	1.476928000
1	-0.185617000	1.497845000	2.482270000
6	-4.503256000	-1.325044000	0.000081000
6	-4.880117000	-0.187030000	0.000310000
1	-5.257243000	0.808538000	0.000516000
1	-4.215248000	-2.349741000	-0.000123000

Frequency	Intensity	Frequency	Intensity	Frequency	Intensity
18.5939	0.7381	614.9698	55.5675	1200.0386	35.3466
20.8419	0.3499	635.4025	0.0605	1218.5196	43.069
35.9091	0.0173	660.5999	42.2003	1292.9572	34.9038
42.0764	0.0544	663.8567	36.0249	1335.74	2.2605
66.374	1.5224	664.4027	82.9044	1420.2587	120.0122
74.6872	3.4122	738.4242	14.5585	1441.5535	63.328
93.1511	1.7717	771.5203	54.843	1508.6136	0.0761
94.2158	0.1746	773.0853	95.3574	1570.8137	2.5812
97.908	9.8938	787.773	144.9055	2059.7638	36.872
187.5242	15.5285	819.1799	45.9847	3032.9936	0.7832
246.4016	0.0641	825.7689	14.9906	3045.622	144.1383
257.5108	0.1975	835.2803	0.7317	3109.8277	0.2311
291.0347	11.2929	847.7397	3.9248	3123.9599	0.3977
316.7133	5.725	921.105	7.305	3186.0043	6.7512
349.6047	1.5159	980.4972	1.4675	3196.8994	4.9549
384.0674	1.0132	1016.2094	0.0701	3247.7591	33.5342
469.0769	5.3046	1027.4036	0.9689	3247.7902	113.8578
524.6844	3.1785	1078.3211	3.3781	3400.0533	108.9171
613.4111	64.3323	1109.8641	9.5886	3499.1943	0.3885

Table S108. Cartesian coordinates for the optimized geometry of isomer 5r-quartet $Ti^{+}(C_2H_2)_5$ followed by its predicted frequencies (cm^{-1}) and IR intensities (km/mol).

Z	x	y	z
22	-0.811014000	0.149466000	0.005282000
6	1.349334000	0.632155000	0.007855000
6	0.945837000	1.809060000	0.535889000
1	1.392597000	2.139632000	1.477562000
1	2.226183000	-0.006221000	-0.050702000
6	-0.209486000	2.544760000	0.079312000
6	-1.017610000	2.132598000	-0.925402000
1	-1.732807000	2.542380000	-1.621530000
1	-0.548804000	3.363617000	0.719583000
1	-0.913841000	-1.406027000	-2.494310000
6	-1.131767000	-1.137906000	-1.468934000
6	-1.951408000	-1.886582000	-0.692238000
1	-2.419526000	-2.788190000	-1.081639000
6	-2.331455000	-1.622680000	0.737949000
1	-3.007143000	-2.378343000	1.133287000
6	-1.918751000	-0.585553000	1.501014000
1	-2.232754000	-0.459879000	2.529813000
6	4.323685000	-1.410649000	0.542730000
6	4.618457000	-0.814402000	-0.454426000
1	4.915876000	-0.302390000	-1.338973000
1	4.101511000	-1.957639000	1.428208000

Frequency	Intensity	Frequency	Intensity	Frequency	Intensity
17.7764	0.1502	632.3742	7.6252	1206.7259	19.9044
21.9633	0.1535	634.9979	27.7018	1225.8396	16.4774
28.9788	0.1571	655.8533	15.2927	1292.9131	40.4306
46.1807	0.0812	666.5784	92.6996	1335.5705	3.5412
68.6825	2.0557	697.4634	75.939	1417.3637	89.3497
75.9912	4.4638	739.0962	23.8143	1441.5677	53.8882
89.4517	0.2896	769.1225	64.8608	1509.7878	1.6677
99.7146	11.5678	783.957	43.1486	1570.571	2.0973
105.8319	2.1079	789.2967	150.4312	2061.0028	34.5067
182.1522	11.5457	807.5863	46.557	3050.5759	0.429
243.2959	1.815	832.9904	3.9841	3063.8542	14.8259
258.3541	0.2322	840.0154	11.8041	3109.6292	0.2157
296.8556	14.1126	848.3572	2.8602	3123.7578	0.6447
322.6549	3.8344	905.6807	12.4466	3136.1422	710.4099
358.7143	1.3891	980.4484	1.8733	3185.8838	7.2298
383.271	0.0819	1016.1717	0.1457	3197.0977	4.4875
469.1578	6.1529	1026.3391	5.8939	3248.0432	77.7029
539.4942	7.6196	1078.0543	5.4178	3401.7404	108.8106
614.1441	70.4661	1109.798	10.046	3500.8989	0.1508

Table S109. Cartesian coordinates for the optimized geometry of isomer 5s-quartet $Ti^{+}(C_2H_2)_5$ followed by its predicted frequencies (cm^{-1}) and IR intensities (km/mol).

Z	x	y	z
6	-1.406664000	0.707380000	0.721826000
6	-0.380407000	1.158747000	1.476919000
1	-0.184941000	1.498635000	2.482108000
1	-2.206965000	0.128891000	1.194642000
22	0.884259000	0.110405000	-0.000011000
6	2.727083000	-1.498732000	-0.000028000
6	1.413670000	-1.819764000	-0.000059000
1	1.067582000	-2.845906000	-0.000092000
1	3.494827000	-2.269699000	-0.000039000
6	2.626365000	1.057860000	0.000055000
6	3.308832000	-0.112500000	0.000021000
1	4.396722000	-0.124310000	0.000027000
1	3.131992000	2.014500000	0.000088000
1	-2.206951000	0.128972000	-1.194690000
6	-1.406657000	0.707431000	-0.721827000
6	-0.380389000	1.158848000	-1.476876000
1	-0.184921000	1.498825000	-2.482035000
6	-4.503196000	-1.325097000	0.000029000
6	-4.880078000	-0.187091000	-0.000025000
1	-5.257224000	0.808469000	-0.000072000
1	-4.215176000	-2.349791000	0.000077000

Frequency	Intensity	Frequency	Intensity	Frequency	Intensity
18.5914	0.7383	614.9717	55.5665	1200.0401	35.3458
20.8401	0.35	635.4014	0.0605	1218.5212	43.0715
35.8961	0.0173	660.6005	42.1689	1292.9569	34.9021
42.0734	0.0546	663.8574	36.0575	1335.739	2.2614
66.3727	1.5221	664.4027	82.9062	1420.2603	120.0129
74.6865	3.4126	738.4249	14.5607	1441.5495	63.3325
93.1456	1.7718	771.5227	54.8433	1508.6146	0.0761
94.2161	0.1732	773.0839	95.3574	1570.8093	2.5814
97.9126	9.8948	787.7717	144.9027	2059.7657	36.869
187.5244	15.5277	819.1841	45.9826	3032.9881	0.7793
246.4006	0.0641	825.773	14.9903	3045.6161	144.1429
257.5155	0.1975	835.2812	0.7326	3109.8287	0.231
291.0286	11.2928	847.742	3.9252	3123.9609	0.3977
316.7133	5.725	921.1081	7.3057	3186.0011	6.7508
349.6005	1.5159	980.4983	1.4676	3196.8969	4.9547
384.0681	1.013	1016.2104	0.0701	3247.7596	33.5498
469.0772	5.3046	1027.4029	0.9685	3247.7907	113.8414
524.6841	3.1784	1078.3238	3.3775	3400.0555	108.9162
613.4113	64.3315	1109.867	9.5879	3499.1965	0.3886

Table S110. Cartesian coordinates for the optimized geometry of isomer 5t-quartet $Ti^{+}(C_2H_2)_5$ followed by its predicted frequencies (cm^{-1}) and IR intensities (km/mol).

Z	x	y	z
22	1.332307000	0.067347000	0.200386000
6	0.295669000	-0.244770000	-1.609147000
6	-1.078092000	-0.224985000	-1.392673000
1	-1.797766000	-0.540453000	-2.143374000
1	0.626960000	-0.529532000	-2.607920000
6	-1.545032000	0.194257000	-0.122756000
6	-0.634552000	0.768828000	0.757261000
1	-0.992561000	1.434270000	1.540947000
1	-2.597457000	0.073483000	0.137353000
6	3.145273000	1.356890000	0.175645000
6	2.202921000	2.134848000	0.000165000
1	1.637934000	3.030045000	-0.173088000
1	4.159883000	1.016222000	0.271360000
1	1.677536000	-2.750151000	-1.025243000
6	1.953376000	-2.243848000	-0.125460000
6	2.361344000	-1.875113000	0.963351000
1	2.821283000	-1.843554000	1.931543000
6	-5.310312000	-0.583087000	0.661764000
6	-5.401211000	0.502189000	0.163067000
1	-5.517378000	1.464370000	-0.275638000
1	-5.265498000	-1.547594000	1.108264000

Frequency	Intensity	Frequency	Intensity	Frequency	Intensity
9.5424	0.1197	507.6542	1.6925	1088.1519	9.5502
15.3074	0.2174	622.3543	7.3533	1126.8843	5.508
20.3015	0.0248	626.5379	8.7432	1174.9917	34.685
41.8398	2.7525	629.5397	0.0484	1294.2194	11.9005
51.0491	3.4005	646.5628	9.5301	1452.7437	51.6038
53.0177	1.1712	649.2645	25.222	1467.1354	49.1287
75.11	0.3666	657.4966	4.0382	1849.2451	90.1953
89.8249	0.2319	681.6258	11.1205	1921.3706	63.1632
92.5508	1.135	685.711	13.0462	2066.4797	7.8628
106.7293	0.357	703.6686	5.5836	3092.204	68.6958
117.4342	0.0209	710.3718	33.0873	3105.9352	1.5105
162.6351	7.1487	720.986	32.3123	3128.6634	7.8581
232.8518	8.4896	748.9732	38.8819	3145.7268	1.5055
276.1756	2.3529	752.0379	124.2161	3279.2999	86.2693
326.6754	22.443	768.5708	94.909	3318.8848	125.3128
343.1615	12.0612	781.5357	140.2381	3357.1258	72.7969
360.0152	3.7324	900.6664	54.3198	3406.9071	100.2383
391.5173	5.816	963.7678	25.0544	3413.3195	81.5844
438.9938	0.3611	1026.3184	3.2961	3505.7669	0.7893

Table S111. Cartesian coordinates for the optimized geometry of isomer 5u-quartet $Ti^{+}(C_2H_2)_5$ followed by its predicted frequencies (cm^{-1}) and IR intensities (km/mol).

Z	x	y	z
6	-0.532837000	-1.808744000	-0.583799000
6	0.688696000	-1.322465000	-0.989720000
6	1.362368000	-0.000440000	-1.160718000
6	-1.808589000	1.280986000	-0.219816000
6	-0.533158000	1.808099000	-0.585190000
6	0.688595000	1.321638000	-0.990194000
1	-0.525128000	2.889007000	-0.498469000
1	-0.524692000	-2.889592000	-0.496359000
1	1.412333000	-2.115819000	-1.145356000
1	1.863589000	-0.000567000	-2.135384000
1	-2.511082000	2.066022000	0.033792000
1	1.412295000	2.114932000	-1.145814000
22	-0.123666000	0.000646000	1.144057000
1	-2.510448000	-2.066451000	0.036187000
6	-1.808204000	-1.281517000	-0.218428000
6	-2.346508000	-0.000245000	-0.075318000
1	-3.378554000	-0.000227000	0.254961000
6	2.452233000	-0.000323000	-0.039467000
6	2.034218000	0.001096000	1.244714000
1	3.489381000	-0.001505000	-0.370654000
1	2.812080000	0.001480000	2.005462000

Frequency	Intensity	Frequency	Intensity	Frequency	Intensity
30.8136	0.6194	802.5034	4.3596	1385.8998	5.9764
60.0689	15.1477	856.7461	0.4482	1418.2192	1.6596
168.9772	0.3077	879.0328	3.9509	1452.3658	0.975
187.1836	4.7415	903.419	16.593	1485.2412	29.9793
198.7587	0.2707	906.8265	4.692	1492.5253	6.0443
205.8615	2.0463	941.355	0.188	1511.2596	41.4163
294.2129	5.3354	971.446	3.9277	1532.3234	6.8119
312.387	4.0043	982.4343	0.4079	1566.5194	31.5801
327.4393	0.3758	998.3586	0.8651	1580.4397	0.3591
340.4243	0.3797	1020.0232	0.3439	3015.1725	0.1399
488.5845	3.0426	1021.2565	0.6774	3106.3462	5.9218
497.9249	25.3821	1110.7828	13.792	3130.3664	9.8276
565.368	12.4071	1123.1048	0.158	3144.6981	0.6595
635.4172	1.6264	1184.5371	6.6591	3144.786	0.1783
700.7279	23.8022	1217.9038	0.1075	3157.5676	0.1881
726.4941	30.2186	1304.6348	0.0019	3159.7748	0.1903
728.0937	20.4455	1314.1988	12.9062	3165.4498	0.0623
740.1124	2.8291	1330.6634	3.5186	3173.2806	0.6495
795.9586	36.9834	1380.0201	1.6276	3181.3048	0.4216

Table S112. Cartesian coordinates for the optimized geometry of isomer 5v-quartet $Ti^{+}(C_2H_2)_5$ followed by its predicted frequencies (cm^{-1}) and IR intensities (km/mol).

Z	x	y	z
6	1.966665000	-0.660884000	-1.022707000
6	2.285482000	0.452900000	0.000075000
6	1.966559000	-0.661064000	1.022621000
6	1.753490000	-1.600868000	-0.000136000
1	2.043309000	-0.714923000	-2.099457000
1	3.351185000	0.685202000	0.000152000
1	2.043078000	-0.715291000	2.099370000
1	1.562129000	-2.665758000	-0.000239000
22	-0.271498000	-0.109950000	-0.000078000
6	1.496513000	1.781464000	0.000153000
6	0.162708000	1.876546000	0.000228000
1	-0.330738000	2.840631000	0.000294000
1	2.139900000	2.658832000	0.000130000
1	-3.342123000	0.779972000	1.243612000
6	-2.652598000	0.131254000	0.706161000
6	-1.704715000	-0.658455000	1.360937000
1	-1.825354000	-1.019015000	2.376839000
1	-3.342234000	0.780476000	-1.243099000
6	-2.652667000	0.131535000	-0.705970000
6	-1.704868000	-0.657940000	-1.361151000
1	-1.825623000	-1.018153000	-2.377162000

Frequency	Intensity	Frequency	Intensity	Frequency	Intensity
66.3677	0.0045	702.9193	11.0352	1185.7021	0.3621
81.125	0.055	723.0739	0.0205	1207.2785	0.2022
84.9029	1.07	741.9318	2.737	1279.7854	0.769
117.4307	1.3641	859.136	29.749	1321.141	20.4044
141.6076	7.2154	902.1655	40.2419	1322.9926	5.7633
149.4109	6.0632	908.7241	6.4935	1419.4302	3.4126
193.3338	4.7643	910.727	2.8907	1427.0466	23.1234
266.6967	1.0771	918.5374	43.4319	1439.292	14.7556
287.6995	2.6842	963.1038	15.719	1568.4304	6.2355
346.6206	6.4009	969.1471	0.0638	3079.9183	2.5973
351.5464	14.6261	974.5386	4.0422	3112.248	0.017
353.6403	13.7953	979.7431	18.9917	3115.6408	0.4653
456.5935	1.8353	986.2507	0.5931	3125.3761	1.4338
513.6638	5.9496	1059.7015	2.1741	3171.4045	4.5404
550.7067	36.4731	1084.6533	6.6796	3171.6269	6.3991
636.7607	26.0541	1102.3306	1.7272	3186.0837	0.5478
647.4459	25.4264	1112.1192	9.1	3205.6824	3.1013
661.2725	71.183	1154.147	36.3042	3224.8467	11.4311
693.3291	28.8081	1179.5094	29.0038	3233.3177	4.8947

Table S113. Cartesian coordinates for the optimized geometry of isomer 5w-quartet $Ti^{+}(C_2H_2)_5$ followed by its predicted frequencies (cm^{-1}) and IR intensities (km/mol).

Z	x	y	z
6	2.628148000	-0.831777000	0.060564000
6	2.069253000	-0.708610000	1.329681000
6	0.843814000	-0.086867000	1.622463000
6	2.148991000	-0.264915000	-1.154749000
6	1.380736000	0.908495000	-1.322695000
6	1.201136000	1.864282000	-0.340978000
1	0.992451000	1.111489000	-2.320691000
1	3.488177000	-1.484682000	-0.037817000
1	2.618036000	-1.189632000	2.136686000
1	0.637136000	0.112795000	2.673511000
1	2.446764000	-0.769762000	-2.068616000
1	1.749779000	2.284701000	0.486397000
22	-0.479002000	0.238776000	0.074371000
6	-2.194139000	1.175028000	0.460624000
6	-2.953376000	0.111782000	0.098907000
1	-4.037679000	0.133934000	0.187146000
1	-2.632281000	2.087205000	0.845433000
1	-3.226143000	-1.903757000	-0.644652000
6	-2.435508000	-1.187212000	-0.431030000
6	-1.130422000	-1.505507000	-0.637018000
1	-0.849983000	-2.483562000	-1.008161000

Frequency	Intensity	Frequency	Intensity	Frequency	Intensity
36.1267	0.1279	665.046	111.8345	1275.1355	3.668
51.0485	0.1884	745.6874	35.8001	1300.6407	21.1639
74.6181	0.5573	754.2217	12.2167	1338.9065	26.9319
94.7068	2.3207	789.0529	26.4237	1386.2993	33.0389
144.0201	8.7925	826.0511	1.7823	1432.814	12.1834
151.2016	1.9129	842.8259	16.3429	1437.0225	52.8138
174.6725	1.6754	865.3145	6.1569	1477.8767	20.5125
212.9864	3.5345	895.7955	18.2183	1493.7387	27.014
269.0377	5.1765	922.1301	5.7456	1562.4014	3.8977
280.0905	0.4813	928.1668	3.7141	3097.2673	7.3646
294.1411	11.0217	993.2656	0.5639	3109.6212	0.4929
352.8169	5.9675	997.1426	0.4447	3111.1235	2.4023
462.585	3.3158	1020.3743	0.2313	3123.6007	2.363
477.7834	4.2641	1042.0785	3.6028	3128.5172	0.3931
484.2111	5.2154	1094.1873	12.9517	3157.593	0.9998
564.672	15.3116	1118.5313	1.2471	3175.9201	2.1878
598.3145	3.9926	1145.2089	8.3468	3183.6531	1.5991
619.4911	72.1556	1202.5905	9.588	3190.5847	5.4626
632.8624	45.6096	1209.3521	1.9428	3245.8073	27.9696

Table S114. Cartesian coordinates for the optimized geometry of isomer 5x-quartet $Ti^+(C_2H_2)_5$ followed by its predicted frequencies (cm^{-1}) and IR intensities (km/mol).

Z	x	y	z
6	-2.675432000	-0.295341000	0.786911000
6	-2.735396000	0.076685000	-0.673422000
6	-1.565733000	-0.463498000	1.543776000
1	-3.760907000	0.131274000	-1.033046000
1	-1.643593000	-0.758190000	2.583171000
6	-1.691334000	0.315921000	-1.501737000
1	-1.856040000	0.553738000	-2.545248000
1	-3.668849000	-0.432338000	1.209163000
22	-0.404580000	0.093875000	0.011936000
6	1.767068000	-0.515230000	-1.218169000
6	1.301920000	-1.645801000	-0.520041000
1	0.803704000	-2.555275000	-0.826790000
1	1.789479000	-0.227247000	-2.259299000
1	3.538785000	0.068836000	0.008214000
6	2.445011000	0.050474000	0.036531000
1	1.952240000	-1.650104000	1.682776000
6	1.851423000	-1.217311000	0.698389000
6	1.885847000	1.328405000	0.608799000
6	0.920744000	2.061496000	0.086972000
1	2.261905000	1.601629000	1.602608000
1	0.459331000	3.027622000	0.227795000

Frequency	Intensity	Frequency	Intensity	Frequency	Intensity
52.9155	0.1912	712.1685	0.3999	1228.7138	6.3279
66.2499	0.2502	745.5916	42.5202	1301.0177	0.4369
76.8571	0.3151	815.8586	15.4918	1305.191	26.6703
111.6819	6.0716	823.3219	14.2184	1327.2749	15.0335
123.1603	6.2143	838.7559	0.1627	1346.948	10.3506
141.1327	4.2962	840.7384	5.3698	1416.9312	4.3386
178.3967	0.6698	890.0558	1.7218	1436.8251	26.8533
241.5569	0.3442	907.6024	1.9512	1572.3668	1.4144
251.3628	0.0237	918.8563	2.6402	1591.5982	3.0368
275.0289	6.1432	944.1212	9.6023	3016.7756	3.5489
289.6944	5.2769	972.8813	2.3733	3044.1765	1.1476
300.3506	12.1928	989.9229	1.9491	3104.8485	0.8562
449.4426	0.2398	1018.6933	0.2591	3120.0102	5.1338
471.644	7.636	1022.9694	4.7853	3180.3151	1.8778
535.3731	17.7281	1093.5199	9.4659	3184.038	1.0337
623.5042	101.1486	1118.824	3.8671	3209.4915	3.5601
658.6986	25.565	1136.0996	15.437	3228.3358	11.1584
661.5795	51.6595	1168.699	7.2073	3237.9175	47.3235
700.6232	69.6798	1212.0928	0.2585	3238.2884	7.1654
